# Second cancers in 475 000 women with early invasive breast cancer diagnosed in England during 1993-2016: population based observational cohort study

**DOI:** 10.1136/bmj-2024-083975

**Published:** 2025-08-27

**Authors:** Paul McGale, David Dodwell, Andrew Challenger, David Cutter, Alexander Williams, John Broggio, Sarah Darby, Gurdeep Mannu, Carolyn Taylor

**Affiliations:** 1 Nuffield Department of Population Health, University of Oxford, Oxford, UK; 2 Oxford University Hospitals NHS Trust, Oxford, UK; 3 University of Exeter Medical School, Exeter, UK; 4 National Disease Registration Service (NDRS), NHS England, Birmingham, UK

## Abstract

**Objective:**

To describe long term risks of second non-breast primary cancers and contralateral breast cancers among women with early invasive breast cancer after primary surgery.

**Design:**

Population based observational cohort study.

**Setting:**

Routinely collected data from the National Cancer Registration and Analysis Service for England.

**Participants:**

All 476 373 women with breast cancer as their first invasive (index) cancer registered in England from January 1993 to December 2016 with follow-up until October 2021.

**Main outcome measures:**

Rates and cumulative risks of subsequent primary cancers, compared with those occurring in the general population; associations with characteristics of patients, index tumours, and adjuvant treatments.

**Results:**

Although 64 747 women developed a second primary cancer, the absolute excess risks compared with risks in the general population were small. By 20 years, 13.6% (95% confidence interval 13.5% to 13.7%) of women had developed a non-breast cancer, 2.1% (2.0% to 2.3%) more than expected in the general population, and 5.6% (5.5% to 5.6%) had developed a contralateral breast cancer, 3.1% (3.0% to 3.2%) more than expected. The absolute excess risk of contralateral breast cancer was greater in younger than in older women. Among specific types of non-breast cancer, the largest 20 year absolute excess risks were for uterine and lung cancers. Although for cancers of the uterus, soft tissue, bones and joints, and salivary glands, as well as acute leukaemias, standardised incidence ratios exceeded those of the general population by a factor of at least 1.5, absolute excess risks at 20 years were <1% for every individual non-breast cancer type. When patients were categorised according to adjuvant treatment, radiotherapy was associated with increased contralateral breast and lung cancer, endocrine therapy with increased uterine cancer (but reduced contralateral breast cancer), and chemotherapy with increased acute leukaemia. These were consistent with effects reported in randomised trials, but positive associations for soft tissue, head and neck, ovarian, and stomach cancers were also identified, and these have not previously been observed in trials. This suggested that approximately 2% of all the 64 747 second cancers and 7% of the 15 813 excess second cancers in the cohort may be attributable to adjuvant therapies.

**Conclusions:**

The risk of a second primary cancer in women treated for early invasive breast cancer is slightly higher than for women in the general population. Contralateral breast cancer accounts for around 60% of the overall increase, with higher risks in younger women. The risk associated with adjuvant therapies is small.

## Introduction

Breast cancer is the most common cancer in women worldwide. It is also increasing in incidence, making it a substantial and growing global health problem.^1^ At the same time, large reductions have occurred in breast cancer mortality in women with a diagnosis of early invasive breast cancer in many countries. For example, in our recent study of 500 000 women in England, we reported a two thirds reduction in the five year risk of breast cancer mortality in women with a diagnosis made during 2010-15 compared with women with a diagnosis during 1993-99.^2^

The landscape of management of early breast cancer has also evolved over this time with the introduction of many new adjuvant therapies and an increased use of these therapies, even in patients with a low risk of mortality and recurrence.^3^ Substantial increases have been seen in the numbers of survivors of breast cancer as well as a greater intensity of adjuvant treatment, creating a need to improve understanding of survivorship considerations and of the risks of long term serious health problems.

Women with breast cancer are recognised to be at higher risk of second primary cancers than women in the general population. These include new primary breast cancers, as well as cancers of other types. The risk and type of second cancer may be influenced by the treatment used for the index (that is, initial) breast cancer, as well as demographic, lifestyle, and genetic factors, including those that may have contributed to the development of the index breast cancer. Many previous studies examining second cancers have arisen from single institutional experiences, whereas others are population based. However, most of these studies are not of sufficient size or duration to characterise these long term risks or to assess associations between characteristics of patients and index breast cancers and the risk of a second cancer. Breast cancer survivors and clinicians need information on the long term absolute magnitude of the risk of developing a second cancer, taking into account patient, tumour, and treatment related factors.

We report a population based observational study of more than 475 000 women with early invasive breast cancer diagnosed at ages 20-75 years in England during 1993-2016 and followed to 2021. We consider the risks of second primary cancers and compare them with the risks in the general population. We also consider associations between these risks and multiple patient and treatment related factors. As our priority is relevance to current care, we focus on the absolute magnitude of the excess risk of a second cancer, allowing for the competing risk of death or of any other second cancer occurring after the index cancer but before the second cancer of interest.

## Methods

### Study population

The National Disease Registration Service, together with its preceding organisations, has registered all patients with a diagnosis of cancer in England for many decades (supplementary text S1). These registrations are routinely linked with other information on each individual patient. For this study, we identified all women registered in England during the period January 1993 to December 2016 with breast cancer as their first invasive cancer. Data items received included calendar year of diagnosis, age at diagnosis, and index of multiple deprivation. For many women, details of pathological stage, grade, oestrogen receptor status, screening status, laterality, region of residence, and adjuvant treatments received were also available (although not recurrence of the index breast cancer or its management). Follow-up information was available up to 31 October 2021 and included the date of any subsequent primary invasive or non-invasive cancers; their type, morphology, and laterality; and, if the woman had died, her date and cause of death.

The study focused on women with early invasive breast cancer who received either breast conserving surgery or mastectomy as their first treatment. We defined early invasive breast cancer as disease detected in the breast. The disease could have spread to the axillary lymph nodes, but with no evidence of metastatic disease. Of the 823 369 women for whom we received data, we excluded those who were aged <20 or >75 years at diagnosis (n=180 296), whose registrations were based on a death certificate only or who had less than three months of follow-up (n=14 937), whose morphology was not invasive breast cancer (n=9444), who had a second invasive cancer of any type within three months of their index breast cancer (n=14 537), or who had likely metastatic disease (n=88 927) (supplementary figure S5). We also excluded women who had received neoadjuvant therapy (n=38 855), as the characteristics of their tumours were recorded before the neoadjuvant therapy was given and were therefore based on clinical staging. This is in contrast to other women, for whom pathological staging was recorded. Data were collated and checked before analysis. In addition, we compiled cancer incidence rates for the whole female population of England (supplementary text S1).

### Statistical analysis

We did four main types of analyses in this study (supplementary text S2). The first was estimation of the cumulative risk of a second cancer by type, starting from three months after the date of diagnosis of the index breast cancer and taking account of the competing risks of any other incident second cancer or of death but ignoring breast cancer recurrence, for which information was not available. The second and third types of analysis estimated the standardised incidence ratios and absolute excess rates (per 10 000 woman years), and the fourth comprised Poisson regressions to examine variation in cancer rates. Analyses of the first three types related rates and risks in the cohort to those of the general population (external comparisons), whereas type 4 compared rates between different subgroups within the cohort (internal comparisons). Rather than considering each individual type of second cancer, we grouped cancer types by ICD (international classification of diseases) code (see supplementary table S1).

We calculated woman years at risk from three months after diagnosis of the index breast cancer to the earliest of any second cancer event, 85th birthday, death, or 31 October 2021. For analyses of invasive cancers, we ignored non-invasive cancers. For analyses of non-invasive cancers, we censored women at the time of any invasive cancer. Some women had more than one cancer occurring after the index breast cancer. Therefore, we also did standardised incidence ratio and absolute excess rate analyses considering cancer at any time more than three months after the index breast cancer as a check that interpretation did not depend on considering just the second cancer.

We stratified the study cohort by attained age, calendar year, and index of multiple deprivation at diagnosis of the index breast cancer, to give observed (o) cancer counts and woman years per stratum. We estimated expected (e) cancer counts by multiplying the general population rates for the same stratum by the woman years. We calculated the standardised incidence ratio (O/E) by summing both the observed (O=Σo) and expected (E=Σe) cancer counts over the strata. We defined the absolute excess rate as (O−E)/woman years. Confidence intervals for standardised incidence ratios and absolute excess rates assumed that the observed counts had a Poisson distribution and the expected counts were fixed (supplementary text S2).

We estimated cumulative risk for each second cancer group by using a two step approach. Firstly, we subdivided the period of follow-up into annual time windows and estimated the probability of a diagnosis of a second cancer in each time window. Secondly, for each time window, we calculated the probability of survival without experiencing a second cancer or death from the start of follow-up to the beginning of that time window. For each time window, we then multiplied these two quantities together and summed the results over all the time windows up to the end of the period under consideration. The outcome was the estimated cumulative risk. This competing risks approach is of clinical relevance as it provides the probability of experiencing the specific second cancer conditional on not having received a diagnosis of any other second cancer or of dying. To derive expected cumulative risks, we considered the general population cancer rates in conjunction with the same competing risks as the study cohort. This maintained consistency between the standardised incidence ratios and any differences between the “observed” and “expected” cumulative risks (supplementary text S2).

We used Poisson regression to investigate variation in cancer rates between subgroups, defined by characteristics of patients and tumours, in the study cohort (table 1). We adjusted the rate ratio for each factor for the other factors.

**Table 1:** Characteristics of study population

	No (%) women in study population		Type of second invasive cancer																					
			Non-breast								Contralateral breast									Unspecified				
			Per cent		Rate*		P value†				Per cent			Rate		P value				Per cent		Rate		P value
**Patients’ characteristics and length of follow-up**																								
Year index breast cancer diagnosed:																								
1993-99	102 105 (22)		29		89.6		<0.001				31			38.0		<0.001				31		2.4		<0.001
2000-04	102 072 (21)		27		87.8						29			38.1						28		2.4		
2005-09	113 521 (24)		24		85.2						24			33.9						25		2.3		
2010-16	158 675 (33)		20		74.2						16			23.3						16		1.5		
Age at index breast cancer, years:																								
20-39	29 300 (7)		2		29.0		<0.001				9		44.2			<0.001				3		1.1		<0.001
40-49	95 844 (20)		13		48.9						23		35.1							11		1.1		
50-59	148 458 (31)		32		79.2						35		34.8							30		1.9		
60-69	145 004 (30)		39		116.0						26		30.8							40		3.0		
70-75	57 767 (12)		14		138.5						7		27.7							16		4.0		
Index of multiple deprivation:																								
<20% (least deprived)	111 780 (23)		22		76.1		<0.001				24			33.0		0.14				20		1.8		<0.001
20-39%	109 012 (23)		22		81.2						23		34.2							21		2.0		
40-59%	98 973 (21)		21		84.7						20		32.8							20		2.1		
60-79%	85 877 (18)		19		88.8						18		35.2							20		2.5		
≥80% (most deprived)	70 731 (15)		16		99.6						15			33.8						19		2.8		
Duration of follow-up, years:																								
3 m-1	7926 (1)		4		51.9		<0.001				3		14.9			<0.001				2		0.6		<0.001
1-4	60 171 (13)		25		65.7						25		25.5							24		1.6		
5-9	152 885 (32)		32		86.9						31		34.0							31		2.1		
10-14	124 115 (26)		22		107.4						24		46.6							25		3.1		
15-19	77 600 (16)		12		120.7						12		50.5							13		3.4		
20-24	41 358 (9)		4		128.2						4		49.8							5		3.4		
25-29	12 318 (3)		1		127.9						1		48.7							0		4.1		
**Characteristics of index breast cancer**																								
Cancer screen detected:																								
Screen detected	135 903 (28)		31	92.6		0.05				26			29.9		<0.001				28		2.2		0.19	
Not screen detected	141 096 (30)		34	94.7						32			36.0						34		2.4			
Not eligible for screening	199 374 (42)		35	71.8						42			34.8						38		2.0			
Tumour size, mm:																								
1-20	240 040 (51)		53	86.1		0.005				49		31.4			<0.001				52		2.1		0.17	
21-50	134 078 (28)		25	84.3						26		34.7							26		2.2			
>50	15 054 (3)		3	79.5						3		44.8							3		2.8			
Unknown	87 201 (18)		19	81.8						22			37.2						19		2.0			
Positive nodes:																								
0	171 886 (36)		33	84.8		<0.001				30		31.3			<0.001				30		2.0		0.82	
1- 3	80 497 (17)		14	75.4						14		30.7							13		1.8			
4- 9	23 031 (5)		4	76.8						4		37.9							4		2.4			
≥10	10 098 (2)		0	80.6						3			47.1						2		1.9			
Unknown	190 861 (40)		49	88.4						49			35.8						51		2.4			
Tumour grade:																								
Low	84 782 (18)		20	87.6		<0.001				19		33.0			<0.001				19		2.1		0.07	
Medium	211 845 (44)		44	85.3						42		32.0							41		2.0			
High	145 446 (31)		27	80.8						30		36.1							31		2.4			
Unknown	34 300 (7)		9	86.8						9			36.7						9		2.3			
Focality:																								
Unifocal	82 926 (17)		17	84.3		<0.001				18		35.8			0.12				21		2.6		0.04	
Multiple	21 527 (5)		3	71.5						4		33.5							3		1.8			
Unknown	371 920 (78)		80	85.3						78			33.3						76		2.1			
Breast quadrant:																								
Lateral	153 462 (32)		32	84.1		0.06				30		32.4			0.04				33		2.2		0.19	
Central	24 199 (5)		5	87.0						6		35.3							6		2.6			
Medial	57 049 (12)		11	85.6						11		33.8							10		1.9			
Overlapping	26 740 (6)		5	81.0						6			34.4						5		1.9			
Unknown	214 923 (45)		47	84.9						47			34.4						46		2.1			
Cancer morphology:																								
Carcinoma NST	382 077 (81)		78	83.6		<0.001				78			33.2		<0.001				80		2.2		0.27	
Lobular	52 903 (11)		12	89.5						12		36.6							11		2.1			
Medullary	2385 (1)		1	81.5						1			46.2						1		1.8			
Metaplastic	1225 (0)		0	120.1						1			48.2						0		4.7			
Mucinous	7789 (2)		2	100.2						1			27.6						1		1.8			
Papillary	1750 (0)		0	102.4						0		36.0							0		1.5			
Tubular	11 853 (2)		3	82.5						3		34.1							2		1.6			
Other/unspecified	16 391 (3)		4	83.3						4			35.6						5		2.6			
**Treatments of index breast cancer**																								
Surgery and radiotherapy:																								
BCS or BCS+RT	297 122 (63)		65		85.3		<0.001				61			32.0		<0.001				57		1.9		<0.001
Mastectomy+RT	57 980 (12)		9		75.8						12		38.2							11		2.3		
Mastectomy	121 271 (25)		26		86.5						27		36.3							32		2.7		
ER status and endocrine therapy use:																								
ER positive (or endocrine therapy)	261 142 (54)		53		85.4		<0.001				47			30.2		<0.001				53		2.2		0.34
ER negative	22 175 (5)		3		77.2						4			43.9						4		2.5		
Unknown	193 056 (41)		44		84.3						49			37.2						43		2.1		
Chemotherapy:																								
None recorded	308 253 (65)		73		92.9		<0.001				65			32.8		<0.001				71		2.3		<0.001
Chemotherapy recorded	168 120 (35)		27		68.3						35			35.5						29		1.8		
**Total**																								
Women, %	100		100		-		-				100			-		-				100		-		-
No of women	476 373		45 467		-		-				18 127			-		-				1153		-		

BCS=breast conserving surgery; ER=oestrogen receptor; NST=no special type; RT=radiotherapy.

* Annual incidence rates per 10 000 woman years.

† Tests for trend, except for breast quadrant, cancer morphology, and surgery and radiotherapy, for which tests are for heterogeneity. Tests exclude unknown categories and for cancer screen detected excludes “not eligible” category.

Assessing the effect of treatment on risk of second cancer in observational data is problematic owing to confounding by indication. On the basis of results from meta-analyses of randomised trials, some cancers (and other diseases) are known to be caused by cancer treatments, and many years may elapse before the risk becomes evident (supplementary table S2). Therefore, we assessed several regression methods (including increasingly complex Poisson models and propensity score matching techniques) and applied them to causes of mortality known to be and known not to be associated with adjuvant breast cancer treatments to provide some guidance before analysing any correlation between second cancer risk and breast cancer treatments in the study cohort.

On the basis of these assessments, we decided on an analysis plan with four components: logistic regression models to examine treatment use by patient and tumour related factors; a Poisson model with second cancer as the outcome and the treatments as the exposure variables, stratified by age in 5 year groups, year of follow-up, year of diagnosis of index cancer, and index of multiple deprivation; comparison of unadjusted and fully adjusted rate ratios, both overall and separately in the follow-up period 0-9 years and ≥10 years after diagnosis of the index cancer; and consideration only of rate ratios >1 (or <1 for contralateral breast cancer and endocrine therapy) that had P values <0.01 in our analyses.

We estimated the fraction of second cancers in the cohort that were associated with the use of adjuvant treatments by estimating the attributable fraction in the exposed ((rate ratio−1)/rate ratio) and hence the numbers of excess cancers (supplementary text S2). We summed these numbers across the second cancer types if their rate ratios followed the fourth guideline described above and according to whether they were consistent with effects found in randomised trials (supplementary table S2).

We used Stata version 17 for all analyses. Where regression models were used, tests for heterogeneity and trend were based on either the likelihood ratio or a variance weighted least squares approach using the effect measures as the dependent variables (Stata vwls command).

### Patient and public involvement

Two patient advocates, Mairead Mackenzie and Hilary Stobart, on behalf of Independent Cancer Patients’ Voice, highlighted the need for information on risks of second cancer to help to inform and reassure survivors of breast cancer and guide the clinicians who care for them. They provided input to the study questions, analyses, results, and interpretation. They emphasised the need to focus on absolute cumulative risks and absolute excess risks, which are directly relevant to patients. They also asked us to provide clinical examples, and these are in the Discussion section. They have agreed to help us with dissemination of our findings.

This work uses data collected by the NHS as part of patient care and support. The data were collated, maintained, and quality assured by the National Cancer Registration and Analysis Service, which is part of the National Disease Registration Service.

## Results

### Characteristics of study population

We included 476 373 women in the study. Of these, 22% received a diagnosis during 1993-99, 21% during 2000-04, 24% during 2005-09, and 33% during 2010-16. For age at diagnosis of the index breast cancer, 7% of women were 20-39 years at diagnosis, 20% were aged 40-49 years, 31% were aged 50-59, 30% were aged 60-69, and 12% were aged 70-75 (table 1). For index of multiple deprivation, 67% lived in areas with index of multiple deprivation classified as within the 60% least deprived areas in England. For follow-up duration, 86% (408 276) of women were followed up for at least five years, 54% (255 391) for ≥10 years, and 11% (53 676) for ≥20 years.

The index cancer was screen detected for 28% of women, and for a further 30% it was not (although these women were in age groups eligible for screening); 42% were in age groups not eligible for routine screening. Where tumour characteristics were known, the most common categories were tumour size ≤20 mm (62% of known), negative axillary nodes (60% of known), medium grade (48% of known), unifocal (79% of known), and lateral breast quadrant (59% of known). For 81% of women, the morphology was breast cancer of no special type (previously known as invasive ductal carcinoma, not otherwise specified), 11% had lobular cancer, and 8% had another or unspecified morphology.

Breast conserving surgery was the surgical treatment for 63% of women, and 37% had mastectomy. Fifty four per cent of women either had a record of receiving endocrine therapy or else had oestrogen receptor positive disease, 5% were recorded as having oestrogen receptor negative disease, and for 41% neither the oestrogen receptor status nor the use of endocrine therapy was recorded. Thirty five per cent of women were recorded as receiving chemotherapy.

### Second invasive primary cancers

A total of 67 064 women went on to develop a second invasive primary cancer; 68% of these were new primary non-breast cancers, 30% were new primary breast cancers, and 2% were cancers of unspecified type. Of the new primary breast cancers, 86% were contralateral to the index breast cancer, 11% (2317) were ipsilateral, and 3% (514) had unknown laterality (supplementary text S1). Distinguishing whether an ipsilateral breast cancer is a new primary cancer or a recurrence of the index cancer is difficult, so these are excluded from all tables and analyses but retained for censoring, leaving 64 747 cancers available for analysis. Second breast cancers of unknown laterality were grouped with contralateral cancers.

**Fig 1:**
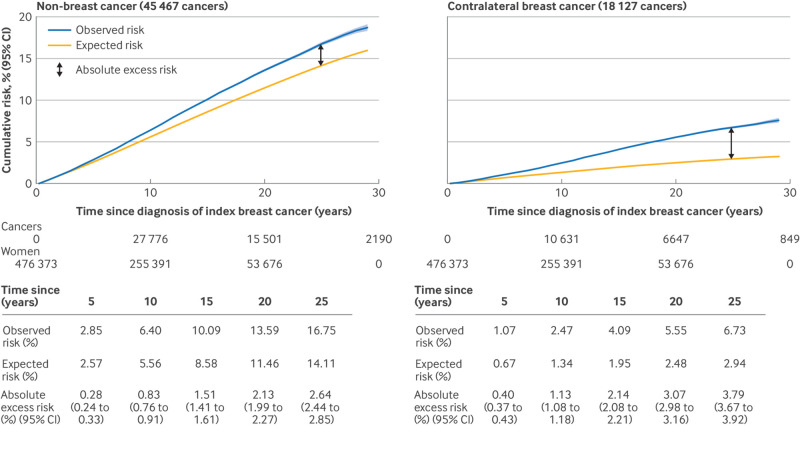
Cumulative risks (and 95% confidence bands) of diagnosis of non-breast cancer as second cancer and of contralateral breast cancer by time since diagnosis of index breast cancer. For each cancer, cumulative risk takes into account competing risk of any other cancer and of death. Also shown are cumulative risks that would be expected if study population had same cancer incidence rates as national rates for all women in England in each calendar year, attained age category, and fifth of index of multiple deprivation. To calculate expected risk for contralateral breast cancer, national incidence rates for breast cancer have been halved. For non-breast cancer, standardised incidence ratio is 1.17 (95% confidence interval (CI) 1.16 to 1.18) and absolute excess rate per 10 000 is 12.63 (11.64 to 13.09). For contralateral breast cancer, these two quantities are 2.10 (2.07 to 2.13) and 17.79 (17.38 to 18.35), respectively

By 20 years after diagnosis of the index cancer, 13.6% of the women in the study population had developed a second non-breast cancer, which was 2.1% (95% confidence interval (CI) 2.0% to 2.3%) more than expected from English national rates, and 5.6% had developed contralateral breast cancer, which was 3.1% (3.0% to 3.2%) more than expected (fig 1). The corresponding overall standardised incidence ratios and absolute excess rates were 1.17 and 12.63 (per 10 000), respectively, for second non-breast cancers and 2.10 and 17.79 (per 10 000) for contralateral breast cancers (fig 1). Thus, 58% (17.79/(12.63+17.79)) of the excess of second cancers was attributable to contralateral breast cancer and the remaining 42% to non-breast primary cancers of different types.

Assessment of the relation between second non-breast cancer incidence and characteristics of patients and tumours via regression found no significant association with the adjusted rate of non-breast cancer for whether the cancer was screen detected, number of positive nodes, quadrant within the breast, and oestrogen receptor status of the index breast cancer, whereas for calendar year of diagnosis, tumour size, tumour grade, and focality, the association was statistically significant but the magnitude of the variation in the adjusted rate ratio was small. By contrast, the adjusted rate of non-breast cancer was significantly and substantially higher for women who were older when the index breast cancer was diagnosed, women whose index breast cancer had metaplastic morphology compared with other morphological types, and women living in more deprived areas (supplementary figure S10). We also observed a positive association between index of multiple deprivation and the adjusted rate for several smoking related cancers (supplementary figure S11).

For contralateral breast cancer, higher adjusted rates were associated with earlier calendar year of diagnosis, younger age at diagnosis, non-screen detected tumours, larger tumours, more positive nodes, lobular morphology, and oestrogen receptor negative disease but not with tumour grade or focality. The association with quadrant and index of multiple deprivation was statistically significant but the magnitude of the variation in adjusted rate ratio was small (supplementary figure S10).

**Fig 2:**
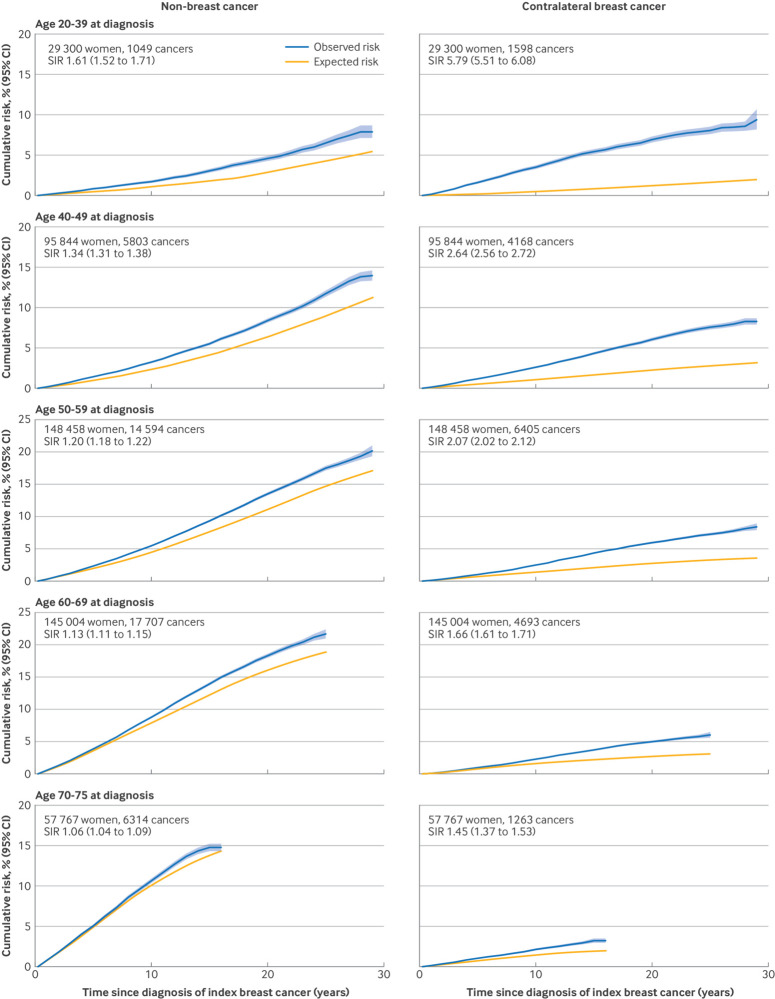
Cumulative risks (and 95% confidence bands) of diagnosis of non-breast cancer as second cancer and of contralateral breast cancer by age at index breast cancer diagnosis and time since that diagnosis. For each cancer, cumulative risk takes into account competing risk of any other cancer and of death. Also shown are cumulative risks expected if study population had same cancer incidence rates as national rates for all women in England in each calendar year, attained age category, and fifth of index of multiple deprivation. To calculate expected risks for contralateral breast cancer, national incidence rates for breast cancer have been halved. See supplementary tables S3 and S4 for cumulative risks, standardised incidence ratios, and absolute excess rates, according to attained age on basis of narrower groupings for age at index breast cancer diagnosis. CI=confidence interval; SIR=standardised incidence ratio

The factor most closely associated with the rates of both second non-breast cancer and contralateral cancer was age at diagnosis of the index breast cancer, but the associations were in opposite directions. Therefore, we plotted cumulative risks separately for women at different ages at diagnosis of their index breast cancer (fig 2). The 20 year absolute excess risk of non-breast cancer varied little across age groups (the risks were 4.6% *v* 2.8% for women aged 20-39 years at diagnosis, 8.4% *v* 6.3% for those aged 40-49, 13.5% *v* 11.2% for those aged 50-59, and 18.3% *v* 16.1% for those aged 60-69) (fig 2). By contrast, the 20 year absolute excess risk for contralateral breast cancer was higher in younger women (6.9% *v* 1.3% in women aged 20-39, 6.0% *v* 2.2% in women aged 40-49, 5.9% *v* 2.7% in women aged 50-59, and 5.0% *v* 2.7% in women aged 60-69). Absolute excess rates for non-breast second cancer for a given attained age did not vary significantly with age at diagnosis of index breast cancer, but absolute excess rates for contralateral breast cancer for a given attained age were greater in patients with younger ages at diagnosis. Supplementary tables S3 and S4 provide greater detail, with values and confidence intervals for standardised incidence ratios, absolute excess rates, and cumulative incidence by age at index breast cancer diagnosis, attained ages, and follow-up period.

### Types of second invasive non-breast primary cancers

**Fig 3:**
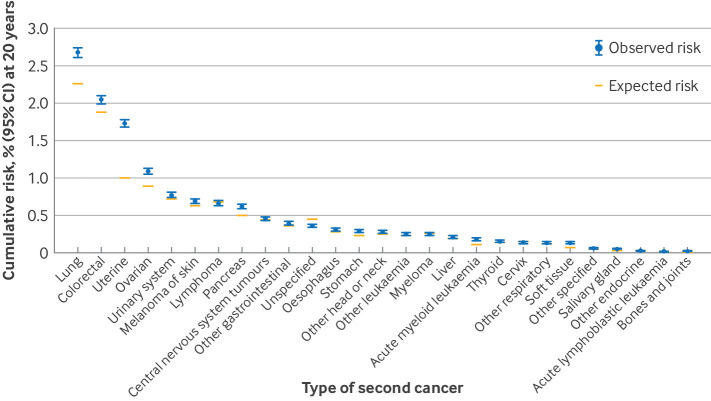
Cumulative risks (and 95% confidence intervals (CIs)) of different types of non-breast cancer at 20 years after diagnosis of index breast cancer. Cancer types are ordered from highest to lowest observed 20 year risk. For each cancer, cumulative risk takes into account competing risks of any other cancer and of death. Also shown are cumulative risks expected if study population had same cancer incidence rates as national rates for all women in England in each calendar year, attained age category, and fifth of index of multiple deprivation. Risks for uterine cancer exclude cervix. See supplementary tables S5-S7 and figures S6-S8 for results based on estimates of standardised incidence ratios and absolute excess rates

The absolute differences between the observed 20 year risks of different types of second non-breast cancer and the corresponding 20 year expected risks in the general population were small (fig 3). The largest 20 year absolute excesses were 0.74% for uterine cancer (non-cervix) and 0.41% for lung cancer, which together accounted for more than 50% of the total 20 year excess. For all other cancer types, the 20 year cumulative absolute excesses were ≤0.2% (supplementary table S5).

Although the absolute excess risks were small for most second non-breast cancers, we observed elevated standardised incidence ratios (>1.20) in several tumour types. The largest values were for soft tissue (2.01), salivary gland (1.95), acute lymphoblastic leukaemia (1.80), uterine non-cervix (1.70), acute myeloid leukaemia (1.69), bone and joints (1.54), stomach (1.27), ovary (1.22), and pancreas (1.21) (supplementary tables S5-7 and figure S6). Rate ratios, excess rates, risks, expected risks, and excess risks, with 95% confidence intervals, for any non-breast, lung, uterine, and contralateral breast cancers by age at diagnosis of index breast cancer and attained age are provided in a look-up tool available at https://livedataoxford.shinyapps.io/BC_SecondCancerRisk/.

Investigation of secular trends in incidence of second cancer is complicated by confounding due to length of follow-up (supplementary figure S7). Limiting the index breast cancer diagnosis period to 1993-2011 and follow-up to 10 years removes this effect. For several cancer types, a significant trend by calendar period of diagnosis remained (supplementary figure S12). In most cases, this is likely to be due to trends in the study cohort following trends in the national rates (supplementary figure S3). The only second cancers with secular trends that differed significantly (P≤0.05) from national rates were contralateral breast, uterine (non-cervix), and ovarian cancers (negative trends) and lung cancer (positive trend) (supplementary figure S8).

### Associations between adjuvant treatments and risk of second invasive cancer

Most women with early invasive breast cancer treated by primary surgery receive some form of adjuvant postoperative therapy.^3^ Breast radiotherapy is recommended for the great majority of women after breast conserving surgery and is commonly given following mastectomy in women with large tumours or nodal involvement. Endocrine therapy is prescribed for most women with oestrogen receptor positive disease, and chemotherapy is advised if poor prognostic features are present, such as large tumour size, positive lymph nodes, or high tumour grade, and for women with triple negative or human epidermal growth factor receptor 2 positive disease. The recording of adjuvant radiotherapy and chemotherapy in the cohort reflected these patterns, but for endocrine therapy, which is often prescribed in primary care and not in hospital, under-recording was clearly apparent for the women in the cohort (supplementary figures S13-S15 and text S1).

#### Radiotherapy

**Fig 4:**
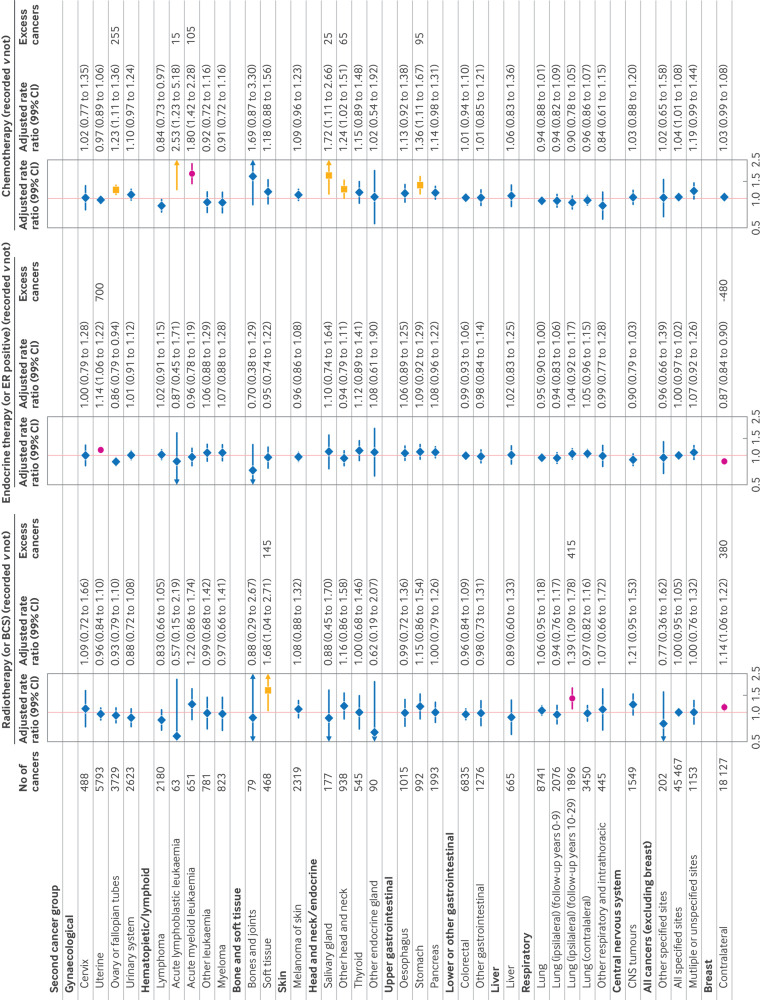
Adjusted rate ratios (and 99% confidence intervals (CIs)) for recording of use of treatments for index breast cancer by type of incident second cancer. Rate ratios (RRs) are stratified for age at index breast cancer diagnosis (5 year age bands), calendar year of diagnosis, time since diagnosis (5 year intervals), and fifth of deprivation. Each treatment category is adjusted for other two and also for surgery (breast conserving surgery (BCS) or mastectomy). All patients treated with BCS are included in radiotherapy recorded group and all women with oestrogen receptor positive disease are included in endocrine recorded group. See supplementary figure S16 for rate ratios before stratification and figures S17 and S18 for rate ratios in follow-up periods 0-9 and 10-29 years. Results in yellow and red indicate results with RR >1 and P value <0.01 (or RR <1 and P value <0.01 if contralateral after endocrine therapy). Results in yellow are not supported by randomised evidence; results in red are supported by randomised evidence. Estimate of number of excess cancers is reported if box is yellow or red. (See supplementary table S2 for summary of randomised trial evidence on adjuvant treatments and second cancer risks and supplementary text S2 for details of excess cancers calculation). CNS=central nervous system; ER=oestrogen receptor

Considering the entire follow-up period, the unadjusted rate ratio for radiotherapy recorded versus not was significantly raised for cancer of soft tissue (1.76, 99% CI 1.27 to 2.43). The increase persisted after adjustment (rate ratio 1.68, 1.04 to 2.71), and the rate ratio was also significantly raised (1.14, 1.06 to 1.22) for contralateral breast cancer (fig 4; supplementary figure S16). When we considered the periods 0-9 years after diagnosis and ≥10 years after diagnosis separately, the adjusted rate ratio for years 0-9 was significantly raised only for contralateral breast cancer (1.16, 1.06 to 1.26) (supplementary figure S17). Beyond 10 years of follow-up, the association between contralateral breast cancer and recording of radiotherapy was no longer significant (rate ratio 1.08, 0.96 to 1.21) (supplementary figure S18). By contrast, we found a strong association between the recording of radiotherapy and lung cancer (rate ratio 1.25, 1.05 to 1.48). This increase was due to lung cancer ipsilateral to the index breast cancer (rate ratio 1.39, 1.09 to 1.78) but not for lung cancer contralateral to the index breast cancer (rate ratio 1.10, 0.83 to 1.45) (fig 4; supplementary figure S18). Considering just the period ≥10 years after the index breast cancer diagnosis, the rate ratio for ipsilateral versus contralateral lung cancer did not vary significantly between the main morphological lung cancer types (supplementary figure S19).

#### Endocrine therapy

For recording of endocrine therapy or oestrogen receptor positive disease versus not, the unadjusted rate ratio was increased for cancer of the uterus (excluding the cervix) (1.14, 99% CI 1.07 to 1.22) and decreased for contralateral breast cancer (0.80, 0.77 to 0.84) (supplementary figure S16). After adjustment, the rate ratios were 1.14 (1.06 to 1.22) for uterine cancer and 0.87 (0.84 to 0.90) for contralateral breast cancer. When we considered the periods 0-9 and ≥10 years after breast cancer diagnosis separately, the adjusted rate ratios were largely unchanged (fig 4; supplementary figures S17 and S18).

#### Chemotherapy

The unadjusted rate ratio for use of chemotherapy versus not was increased for acute lymphoblastic leukaemia, acute myeloid leukaemia, salivary gland cancer, thyroid cancer, and contralateral breast cancer, whereas for many other types of cancer the unadjusted rate ratio was significantly decreased (supplementary figure S16). Most of these decreases probably reflect the age distribution of cancer incidence, as increased age at index breast cancer diagnosis was strongly associated with a lower use of chemotherapy (supplementary figure S15). After adjustment for age at diagnosis and other factors, all these significant decreases disappeared but significant increases remained between chemotherapy use and cancer of the ovary or fallopian tubes (rate ratio 1.23, 99% CI 1.11 to 1.36), acute lymphoblastic leukaemia (2.53, 1.23 to 5.18), acute myeloid leukaemia (1.80, 1.42 to 2.28), and cancer of the salivary gland (1.72, 1.11 to 2.66), other head and neck sites (1.24, 1.02 to 1.51), and stomach (1.36, 1.11 to 1.67) (fig 4). The raised rate ratio for cancer of the ovary or fallopian tubes was present in both the 0-9 year and the 10-29 year periods of follow-up, whereas the raised rate ratios for acute lymphoblastic leukaemia, acute myeloid leukaemia, and stomach cancer were present in the 0-9 year but not the 10-29 year period of follow-up. In the 10-29 year period of follow-up, the rate ratios were raised for uterine cancer (non- cervix), cancers of the head and neck (apart from salivary gland), and cancer of the pancreas (supplementary figures S17 and S18).

### Interpretation of associations between adjuvant treatments and risk of second invasive cancer

Figure 4 shows absolute numbers of excess second cancers of different types associated with the three modalities of adjuvant therapy. However, we show these only for second cancers for which rate ratios and P values follow the description in the methods section (rate ratio >1.0 (or <1.0 for contralateral breast cancer in women prescribed endocrine therapy) and P<0.01). In figure 4, we also indicate, using yellow and red colours, which of these associations are consistent with effects found from meta-analyses of randomised trials (red) or not (yellow) (supplementary table S2).

Summing these numbers and allowing for the reduction in contralateral breast cancer associated with the use of endocrine therapy suggests that 1120 (1.7%) to 1720 (2.7%) second cancers compared with an overall total of 64 747 may be due to the use of adjuvant therapies, depending on whether the associations that have not been shown in meta-analyses of randomised trials are included. Supplementary table S5 gives the excess numbers of second cancers in the cohort compared with the general population (64 747−48 934=15 813), allowing an estimation that 7.1% (10.9% if including associations not found in randomised trials) of the excess number of cancers may be due to the use of adjuvant therapies.

### Non-invasive cancers

The most common second non-invasive cancer was contralateral carcinoma in situ, with 3485 cases registered, of which only 147 were recorded as ipsilateral. For the remaining 3338 cases, 84.1% were of intraductal morphology (ductal carcinoma in situ) (supplementary table S8). The standardised incidence ratio was 2.81 (95% CI 2.72 to 2.91) with an absolute excess rate of 4.10 (3.90 to 4.33) per 10 000 woman years.

Within the cohort, women who were younger when their index breast cancer was diagnosed had a higher rate of contralateral ductal carcinoma in situ (P<0.001), as did those with a larger index breast cancer (P=0.01). The distribution, absolute numbers, standardised incidence ratios, and absolute excess rates and the associations between incidence rates and characteristics of patients and tumours for all recorded non-invasive cancers are provided in supplementary figure S1, table S8, and figure S20.

### Subsequent (second and beyond) invasive cancer

As a sensitivity analysis, we considered the patterns of development of further cancers beyond the first second cancer. The distribution of third cancer types closely matched that of both invasive and non-invasive second cancers (supplementary figure S1). The standardised incidence ratios and absolute excess rates were similar to those seen when we considered only the second cancer (supplementary figure S9).

## Discussion

### Principal findings

In this study of 476 373 women in England with a diagnosis of early invasive breast cancer from January 1993 to December 2016, survivors of breast cancer had a higher risk of some second cancers than did women in the general English population. Overall, the absolute 20 year risks for breast cancer survivors were 14% for non-breast cancer, compared with 12% in the general population, and 6% for contralateral invasive breast cancer, compared with 3% in the general population. Thus, most of the excess cancers were contralateral breast cancers, especially in women who were younger at diagnosis of the index breast cancer. Larger tumour size and degree of nodal involvement of the index breast cancer were also associated with a greater risk of developing contralateral breast cancer.

For non-breast second cancers, the excess risk was mostly from common cancers including uterine (non-cervix), lung, and colorectal. When 20 year risks of these individual cancer types were compared with those expected in the general population, the absolute excess risk was less than 1% for any cancer type. For rarer cancers, risks of acute leukaemias, soft tissue cancer, and salivary gland tumours were higher than in the general population, but they contributed little to the overall increase in absolute risk.

We observed associations between types of adjuvant therapy and some types of second cancer. Use of radiotherapy was associated with higher rates of contralateral breast, lung, and soft tissue cancer. Endocrine therapy was associated with an increased rate of uterine cancer but with a reduced rate of contralateral breast cancer. Rates of uterine and contralateral breast cancer during the first 10 years after diagnosis were lower in more recent calendar periods of index breast cancer diagnosis. Chemotherapy use was associated with salivary gland, stomach, head and neck, and ovarian cancer, as well as acute leukaemia.

### Strengths and weaknesses of study

This is a large population based study with very long term follow-up describing the development of second cancers according to multiple characteristics of patients and tumours and modes of treatment. Many of these characteristics were unavailable in previously published studies. The women included in this study were chiefly those included in our previous study of breast cancer mortality.^2^ Before analysis, the data were extensively checked by statisticians, oncologists, a breast surgeon, and a pathologist, and inconsistencies and misclassifications were corrected. The analyses and manuscript development reflected ongoing input from our two patient representatives. We focused on absolute risks, which are most relevant for survivors of breast cancer, as well as for clinical management and public health policy. We also included risks of non-invasive second cancers, subsequent (third, fourth, and so on) cancers and used a competing risks approach in analyses to provide estimates of second cancer risks that are relevant to breast cancer survivors. We assessed possible misreporting of breast cancer metastases as new primary cancers by considering the rates of second cancers of the lung, central nervous system, liver, and bones according to degree of nodal involvement of the index breast cancer (supplementary figure S2). Secular incidence patterns in the general population—for example, the reduction in recorded incidence of most cancer types during the covid pandemic—are mirrored in the cohort and provide confidence in the plausibility of our findings.

A limitation of our study was that the cancer registry data were incomplete for some variables, such as adjuvant treatments delivered. The possibility of greater ascertainment of cancer diagnoses in a population of breast cancer survivors than in the general population also exists. Source data verification is not possible when using large scale cancer registry data. We did not have information on family history, genetic predisposition, and lifestyle choices including smoking. We had insufficient data on comorbidities, performance status, and body mass index to include these variables. As <5% of women were recorded as non-white, ethnicity data could not reasonably be studied; nor did we study men.

Finally, causality cannot be easily proved when analysing observational data, and the associations we identified should be considered in the light of the totality of evidence from this and other studies. The rate ratios we identified that relate to the associations with adjuvant therapies were in general closer to unity than those identified in relevant EBCTCG meta-analyses (supplementary table S2). This may be due to variation in the type and duration of chemotherapy and endocrine therapy given, lower treatment adherence than in randomised trials, and under-ascertainment of adjuvant therapies within the cohort.

### Strengths and limitations compared with other studies

A recent publication from Allen and colleagues reported on a large cohort of patients with a diagnosis of breast cancer in England.^4^ Their lack of a competing risks approach led to estimates of second cancer risks that are higher than those experienced by breast cancer survivors, and adjustment for deprivation in the general population was not made when standardised incidence ratios were estimated. The analyses focused on incidence rates, whereas our main focus was to provide patients and clinicians with estimates of absolute risks.

Our findings are consistent with those of Allen and colleagues in some respects, including the higher risks of lung, endometrial, and contralateral breast cancer, but we found novel findings in that higher tumour and nodal stage were associated with the development of contralateral breast cancer and that the rate of contralateral breast cancer fell in more recent calendar year periods of index breast cancer diagnosis. We also observed falling rates of endometrial cancer from around 2005, consistent with the introduction of aromatase inhibitors in preference to tamoxifen (supplementary figures S3 and S8).^5^

Previous studies of the risks of second primary cancers following breast cancer have reported qualitatively similar findings to ours, in terms of incidence rates for second cancers in totality, as well as for specific cancer types. However, few quantify the small absolute differences in cumulative risk, which allow a better appreciation of the risk of second cancer in the real world, especially in the context of the risk of mortality from the index breast cancer itself (supplementary table S9).

### Implications for patients, clinicians, and policy makers

The risks of invasive cancers are higher for breast cancer survivors than for other women in the general population. These additional risks are small in comparison with the risks of recurrence and death from breast cancer in the great majority of women with a diagnosis of early invasive breast cancer despite the substantial falls in breast cancer mortality recently reported.^2^ This is an important consideration in efforts to reduce morbidity by de-escalating treatments, as it may compromise breast cancer cure. Lifestyle factors including smoking and obesity have a greater impact on second cancer risk than does a previous breast cancer diagnosis.^6^

The risks in this study can inform patients with breast cancer and the clinicians who treat and support them. For example, for a woman whose index breast cancer was diagnosed when she was aged 60, her risks of developing a new cancer by the age of 80 are 17% for non-breast cancer and 5% for contralateral breast cancer compared with risks of 15% and 3%, respectively, for women of the same age in the general population. For a woman with breast cancer diagnosed when she was aged 40, her risks of developing a second invasive cancer by the age of 60 are 6% for both non-breast and contralateral breast cancer compared with 4% and 2%, respectively, for women in the general population. As described in the accompanying patient perspective,^7^ many breast cancer survivors believe their risks of a second cancer are much higher than those we estimated. The information in our study can reassure them and help them to plan their future.

Our findings are also relevant to clinical practice and policy. The associations between radiotherapy and excess lung and contralateral breast cancers are consistent with those in randomised trials and suggest that irradiation of the lung and contralateral breast should be minimised and smoking cessation advice strengthened. Current post-treatment surveillance imaging of the contralateral breast involves annual mammography for only five years in most cases, but the higher risks in younger women warrant reassessment of this practice.

The associations between endocrine therapy and reductions in contralateral breast cancer and increases in uterine cancer are also consistent with randomised clinical trials and can inform the use of these treatments in the care of patients with breast cancer and in chemoprevention. The higher contralateral breast cancer rates associated with younger age are consistent with a genetic aetiology, and those associated with higher stage of the index breast cancer may suggest that some contralateral cancers are metastases from the index breast cancer. Similar findings have been reported in other recent studies.^8^ ^9^ ^10^ ^11^ Our findings indicate that a need may exist to investigate the possible presence of distant metastatic disease to guide management in patients who develop contralateral disease.

### Unanswered questions and future research

Breast cancer mortality after a diagnosis of early breast cancer is likely to continue to fall, as treatment for breast cancer continues to evolve. The improving survival prospects for patients with early breast cancer and the uncertainty about the long term effects of recently introduced interventions including new drugs and modifications to radiotherapy (for example, regional nodal radiotherapy^12^) mean that the risks of second cancers need ongoing study.

Beyond these considerations, our findings may inform the development of algorithms to assess cancer risk,^13^ ^14^ as well as the design of future studies to determine the frequency and duration of post-treatment surveillance imaging according to the characteristics of patients, tumours, and treatment.^15^ Absolute excess risks of non-breast cancer for survivors of breast cancer are small relative to the cancer risks in the general population, and for contralateral breast and uterine cancers they seem to be falling. Therefore, future research directed at overall cancer prevention rather than at trying specifically to reduce the small excess in survivors of breast cancer may be of greater overall public health benefit.^6^

### Conclusion

For patients with a diagnosis of early breast cancer, the long term risks of developing a second cancer depend chiefly on age at diagnosis of breast cancer. The excess risk of non-breast cancer, over that experienced by the general population, is small compared with the risk of mortality from breast cancer for most women. Adjuvant treatments contribute around 7% of this excess risk, and trial data show that their benefits outweigh this risk in almost all circumstances. This information is of value to patients with early breast cancer and to the clinicians who care for them.

## What is already known on this topic

Breast cancer survivors are at increased risk of developing second primary cancers
The methods that have been used to estimate this risk are inconsistent and varying associations have been reported with different cancer types
The relations between second primary cancer risk and characteristics of patients, index breast cancer, and treatment are not clearly defined

## What this study adds

The 20 year absolute excess risk of contralateral breast cancer was ~3% overall, but larger for younger women and those with lobular phenotype or higher stage disease at index breast cancer diagnosis
The 20 year absolute excess risk of non-breast primary cancer was smaller (~2%), with lung and uterine cancer contributing most of the risk
Adjuvant radiotherapy and systemic therapies were associated with around 7% of the excess number of cancers

## Data Availability

De-personalised study data may be made available on request to accredited researchers who submit a proposal that is approved by NHS England’s Data Access Request Service (DARS). An interactive look-up application to accompany supplementary tables S3-S7 is available at https://livedataoxford.shinyapps.io/BC_SecondCancerRisk/. Analytical code used to derive results is available in supplementary appendix 2.

## Web extra

Extra material supplied by authors

Web appendix: Supplementary materials

Web appendix: Analytical code

## Notes

Contributors: PM, JB, DD, CT, GM, and SD developed the study concept and design. PM, AC, JB, GM, and SD contributed to the data collection and collation. PM, DD, AC, and SD contributed to the data analysis. DD did the literature review. All authors participated in data interpretation and manuscript preparation. All authors read and approved the final version. PM and DD contributed equally (joint first authors) and are the guarantors. GM and CT contributed equally (joint last authors). The corresponding author attests that all listed authors meet authorship criteria, and that no others meeting the criteria have been omitted.

Transparency: The lead authors (the manuscript’s guarantors) affirm that this manuscript is an honest, accurate, and transparent account of the study being reported; that no important aspects of the study have been omitted; and that any discrepancies from the study as planned (and, if relevant, registered) have been explained.

Dissemination to participants and related patient and public communities: The results have already been presented to representatives of Independent Cancer Patients’ Voice. After publication, they will be freely available both to stakeholders and to the broader public.

Provenance and peer review: Not commissioned; externally peer reviewed.
