# Supplementary material for: Second cancers in 475 000 women with early invasive breast cancer diagnosed in England during 1993-2016: population based observational cohort study
Source: BMJ. 2025 Aug 27;390:e083975. doi: 10.1136/bmj-2024-083975 (PMC12381949; doi:10.1136/bmj-2024-083975)
Supplement: Supplementary file 1 — Web appendix: Supplementary materials [file mcgp083975.ww1.pdf]

Second cancers in 475 000 women with early invasive breast cancer diagnosed in England during 1993-2016: Population based observational cohort study

Supplementary Appendix 1

**Statistical Methods and Basic Tabulations**		**Page**
Text S1	Data	2
Figure S1	Distribution of second and of third cancers for invasive and non-invasive cancers following breast cancer as the first cancer in 476,373 women	5
Figure S2	Adjusted rates ratios of second cancers known to be common sites of metastatic breast cancer by number of nodes to which the index breast cancer had spread	6
Figure S3	Study population and national average adjusted second cancer rates (per 10,000) by current calendar year	7
Figure S4	Study population and national average adjusted second cancer rates (per 10,000) by attained age	10
Figure S5	Composition of study population	13
Text S2	Statistical methods	14
Table S1	Definitions of cancer categories by ICD 9 and 10 codes	17
Table S2	Rate ratios from randomised trials for the effects of breast cancer treatments on causes of second cancers	18

Analysis of Invasive Cancers

Comparisons with the general population (i.e. national incidence rates)

Table S3*	Non-breast cancer standardised incidence ratios, absolute excess rates (per 10,000), and cumulative risk by age at index breast cancer and attained age	20
Table S4*	Contralateral breast cancer standardised incidence ratios, absolute excess rates (per 10,000), and cumulative risk by age at index breast cancer and attained age	21
Table S5*	Standardised incidence ratios, absolute excess rates (per 10,000), and 10- and 20-year cumulative risk by type of second cancer	22
Table S6*	Standardised incidence ratios, absolute excess rates (per 10,000), and 10- and 20-year cumulative risk by type of second cancer for women aged 20-59 years at diagnosis of index breast cancer	23
Table S7*	Standardised incidence ratios, absolute excess rates (per 10,000), and 10- and 20-year cumulative risk by type of second cancer for women aged 60-75 years at diagnosis of index breast cancer	24
Figure S6	Standardised incidence ratios for different second cancer groups by age at breast cancer diagnosis	25
Figure S7	Standardised incidence ratios for different second cancer groups by time since breast cancer diagnosis	26
Figure S8	Standardised incidence ratios for different second cancer groups by year breast cancer diagnosed	27
Figure S9	Standardised incidence ratios and absolute excess rates by type of non-breast second cancer occurring at any time	28

**Results also available - https://livedataoxford.shinyapps.io/BC_SecondCancerRisk/*

Comparisons between subgroups within the study population cohort

Figure S10	Adjusted rate ratios for non-breast cancer and contralateral breast cancer by patient factors and characteristics of the index breast cancer	30
Figure S11	Adjusted rate ratio for type of second cancer per quintile increase in the index of multiple deprivation	31
Figure S12	Adjusted rate ratio for type of second cancer per 5-year increase in the calendar year the index breast cancer was diagnosed	32
Figure S13	Recording of use of radiotherapy, to treat the index breast cancer, by patient and tumour characteristics.	33
Figure S14	Recording of use of endocrine therapy, to treat the index breast cancer, by patient and tumour characteristics	34
Figure S15	Recording of use of chemotherapy, to treat the index breast cancer, by patient and tumour characteristics	35
Figure S16	Unadjusted rate ratios for the recording of the use of treatments for the index breast cancer by type of second cancer	36
Figure S17	Adjusted rate ratios, in the 0-9 year period of follow-up, for the recording of the use of treatments for the index breast cancer by type of second cancer	37
Figure S18	Adjusted rate ratios, in the 10-29 year period of follow-up, for the recording of the use of treatments for the index breast cancer by type of second cancer	38
Figure S19	Relative risks for lung cancer, ipsilateral vs. contralateral, by recording of use of radiotherapy, year index breast cancer diagnosed, and time since diagnosis	39

Analysis of Non-invasive Cancers

Table S8	Standardised incidence ratios and absolute excess rates by type of non-invasive second cancer	41
Figure S20	Adjusted rate ratios for non-invasive non-breast cancer and contralateral CIS breast cancer by patient factors and characteristics of the index invasive breast cancer	42

Comparable studies

Table S9	Other studies and reviews of second cancer after breast cancer	44

Text S1: Data

1. Breast cancer cohort

a) Data

Cancer Registration

In England, data have been collected and registered for all patients treated within the National Health Service (NHS) for many decades. The process is currently managed by the National Disease Registration Service (NDRS) which collects information on patient details, and on type and stage of cancer and its treatment. Data are obtained by the NDRS from a range of sources, including histopathology services, hospital treatment records and patient administration systems. NDRS endeavours to register everyone with a cancer before they die of it, the few who are not registered are added to the cancer registrations but flagged as 'death certificate only'. If a patient has been registered with more than one cancer then, provided they have different site codes, they are registered as different events, even if they occur on the same day. Cancers with the same site code are coded only once (on the earliest date) unless they are recorded as having a different laterality or a different morphology, in which case they are regarded as separate cancers. To avoid registering metastatic disease, a new primary tumour needs to be at a different anatomical site and of a different histological type from the first tumour, or to be stated explicitly as being a new tumour. For patients who die, the date and cause of death is provided by the Office for National Statistics. Further details are given in Henson et al 2020.

Data received

The central dataset received from the NDRS contained one record for every woman registered with invasive breast cancer during the period 1 January 1993 to 31 December 2016 (see Taylor et al 2023 for a full description). Women who had previously been registered with an invasive cancer were excluded. The file contained a pseudonymised patient identifier, a pseudonymised tumour identifier for each woman's first ('index') breast cancer, and details about the woman and of that cancer, including the quintile of the index of multiple deprivation (IMD) for the area in which the woman lived. IMD is the official measure of relative deprivation for small areas (neighbourhoods) in England. It was introduced in 2000 and is updated every few years (Smith et al 2015). For both the study cohort and the national data (see below), IMD for women diagnosed with breast cancer in the pre-2000 period was based on the year 2000 assessment of small areas.

A cancer history file was also provided, this contained any previous non-invasive breast cancers and any subsequent non-invasive and invasive cancers up to 31st October 2021. The cancers were coded using either the 9th revision or 10th revision of the International Classification of Diseases (ICD) or the 2nd or 3rd edition of the International Classification of Diseases for Oncology. After standardising to ICD code, the individual 4-digit site ICD codes were grouped into categories corresponding to cancer sites for use in the present study (Table S1).

Underlying cause of death was available up to March 2023. Deaths from cancer as the underlying cause were considered, except deaths certified as due to either breast cancer (ICD-9 174; ICD-10 C50) or cancer of an ill-defined/unspecified site (ICD-9 195-199; ICD-10 C76-C80). The at-risk period was taken to be 3 months from diagnosis of the index cancer, to the earliest of 85th birthday, death, or 31st October 2021. For each woman, any subsequent invasive or non-invasive cancers (ignoring non-melanoma skin cancer: ICD-9 173 & 232, ICD-10 C44 & D04) were organised into temporal order. If the cancer was in the lung or the breast the laterality was noted, enabling it to be classified as either ipsilateral or contralateral to the index breast cancer.

Invasive cancers

Among the 476,373 women in the cohort, a total of 74,557 subsequent invasive cancers were registered; 60,168 women had only one subsequent invasive cancer, 6,337 had two, 522 had three, 36 had four, and 1 had five. This gave 67,064 cancers available for analysis of time to first subsequent invasive cancer (ie the second invasive cancer). Ties on dates were handled by giving preference to any breast event, but otherwise the selection was random. The number of ties (277) was very small compared to the total number of cancers. If there was a registration in the cancer history file less than a month before death and the underlying cause of death was given as a different non-breast cancer then the entry in the cancer history file was taken as the second cancer. Figure S1a shows that the distributions of second and third invasive cancers, by type, look similar.

Considering the 67,064 second invasive cancers; 66,456 were registered prior to death, only 608 cancers were named on the death certificate with no previous entry in the cancer history file. Of the second cancers identified as a new invasive breast primary, 17,613/20,444 (86%) were contralateral to the index breast cancer, 2317 (11%) were ipsilateral, and for 514 (3%) insufficient laterality information was available to classify them. Given the overwhelming majority of the new breast primaries were contralateral, the 514 cancers lacking laterality information were grouped with the contralateral breast cancers.

Non-invasive cancers

Non-invasive cancers were censored by any prior invasive cancer. After the censoring process was carried out, a total of 9,462 subsequent non-invasive cancers remained; 8,981 women had only one, 225 had two, 9 had three, and 1 had four. Figure S1b shows that the distributions of second and third non-invasive cancers, by type, look similar. Among non-invasive cancers identified as carcinoma in situ of the breast (CIS), 3,296/3,485 (95%) were contralateral to the index breast cancer, 147 (4%) were ipsilateral, and for 42 (1%) insufficient laterality information was available to classify them. The 42 cancers lacking laterality information were grouped with the contralateral breast cancers.

Adjuvant breast cancer treatments

Information was available for some women on the type of breast cancer treatments received. Restricting to the 2010-2016 period recording of radiotherapy aligned well with expectation in its use after breast-conserving surgery (~95%) and after mastectomy (~45%) and there was very little variation between geographical regions. However, for earlier periods (1993-2009) there was substantial variation between geographical regions and its recorded use after breast-conserving surgery was only 52%. This is likely to reflect a deficit in recording in the earlier period rather than any change in use. Therefore, it was assumed that if breast-conserving surgery was given, radiotherapy was also given irrespective of calendar year of registration. Use of endocrine therapy was poorly recorded throughout the whole 1993-2016 period. However, several publications that considered the primary care prescription database have demonstrated that a very high proportion of women with ER+ breast cancer in England during 1995-2016 were prescribed endocrine therapy (Emanuel et al 2019, Gannon et al 2023). Therefore, to make best use of the available data, a variable was created with the following three categories; "endocrine therapy recorded and/or ER-positive", "ER-negative" and "ER-status unknown and endocrine therapy not recorded" (however, in the analyses presented in figures 4 and S16-S18 the last two groups are combined). The recording of the use of chemotherapy varied little by geographical region over the study period. Therefore, it was assumed that the use of chemotherapy was well documented in the data.

b) Plausibility of true new primary cancers

Breast cancer has several common metastatic sites. It is therefore possible in principle that some of the primary cancers recorded (liver, bone, central nervous system, and lung) might actually be metastatic cancers of the original breast cancer. The rate of metastatic cancer increases with number of lymph nodes involved with cancer at the time of breast cancer diagnosis. Therefore, the variation in the rates of these cancers by the number of positive lymph nodes was examined (figure S2). The lack of any large trends gives indicates that the registered second invasive cancers for these sites is not materially contaminated by metastatic breast cancers.

2. Cancer incidence rates and population count data for the whole of England

The overall numbers of cancer registrations for the whole of England were received from the NDRS for the period January 1993 to December 2021. The data comprised cancer counts for both non-invasive and invasive disease, by 4-character ICD code for each individual calendar year, sex, age-group and quintile of the index of multiple deprivation. Age-groups were in five-year categories up to age 80-84 but 85+ years was given only as a single group. The study cohort was therefore limited to women aged less than 75 years, to allow all cohort members to have the potential of at least 10-years of follow-up. The Office for National Statistics provided population counts subdivided by the same factors for which the cancer data were made available. The numbers of cancer registrations and the population counts were combined to produce national cancer incidence rates according to the categories of interest (Table S1).

3. Temporal patterns in cancer rates in the study cohort and national datasets

If there are large temporal changes in cancer incidence rates in the cohort and/or general population these should be present in both datasets if cancer registrations are being equally recorded. An example of a systematic temporal change occurred during the recent COVID epidemic (starting 2020) when cancer screening services were suspended (Armitage and Morling 2021). Plotting cancer rates by current calendar year (and also by attained age) should reveal if patterns in one dataset are being mirrored in the other. In figure S3, the rates of many common cancers can be seen to drop in the year 2020 in both the cohort and the general population. Thereafter the rates recover to those of previous years. For cancers showing strong secular trends in the general population eg stomach cancer (figure S3), these are also occurring in the study cohort. Patterns by attained age in the study cohort and general population (figure S4) appear to be commensurate. These comparisons suggest the record-linkage of cancer registrations has been successful.

References

Katherine E Henson, Lucy Elliss-Brookes, Victoria H Coupland, Elsita Payne, Sally Vernon, Brian Rous, Jem Rashbass, Data Resource Profile: National Cancer Registration Dataset in England, *International Journal of Epidemiology*, Volume 49, Issue 1, February 2020, Pages 16–16h, https://doi.org/10.1093/ije/dyz076

Taylor C, McGale P, Probert J, Broggio J, Charman J, Darby S C et al. Breast cancer mortality in 500 000 women with early invasive breast cancer diagnosed in England, 1993-2015: population based observational cohort study *BMJ* 2023; 381 :e074684 doi:10.1136/bmj-2022-074684

Tom Smith, Michael Noble, Stefan Noble, Gemma Wright, David Mclennan, Emma Plunkett. (2015). The English Indices of Deprivation 2015: Research Report. (https://assets.publishing.service.gov.uk/media/5a80528840f0b62305b8a78f/English_Indices_of_Deprivation_2015_-_Research_Report.pdf accessed 26th Sept 2024)

ICD: World Health Organization. International Statistical Classification of Diseases and Related Health Problems 10th Revision. 2019 [cited 2024 23/07/2024]; Available from: https://icd.who.int/browse10/2019/en. 9th Revision. 1975. World Health Organisation: Geneva

Gabrielle Emanuel, Katherine E. Henson, John Broggio, Jackie Charman, Kieran Horgan, David Dodwell, Sarah C. Darby. Endocrine therapy in the years following a diagnosis of breast cancer: A proof of concept study using the primary care prescription database linked to cancer registration data. Cancer Epidemiology, Volume 61,2019,Pages 185-189,https://doi.org/10.1016/j.canep.2019.04.012.

Gannon MR, Dodwell D, Miller K, et al. Completeness of endocrine therapy information in the Primary Care Prescription Database (PCPD) and secondary care treatment datasets: A national population-based cohort study using routine healthcare data. Cancer Epidemiology. 2023 Oct 1;86:102423.

Armitage RC, Morling JR. The impact of COVID-19 on national screening programmes in England. Public Health. 2021 Sep;198:174-176. doi: 10.1016/j.puhe.2021.07.022. Epub 2021 Jul 29. PMID: 34461451; PMCID: PMC8318685.

a) Invasive cancers

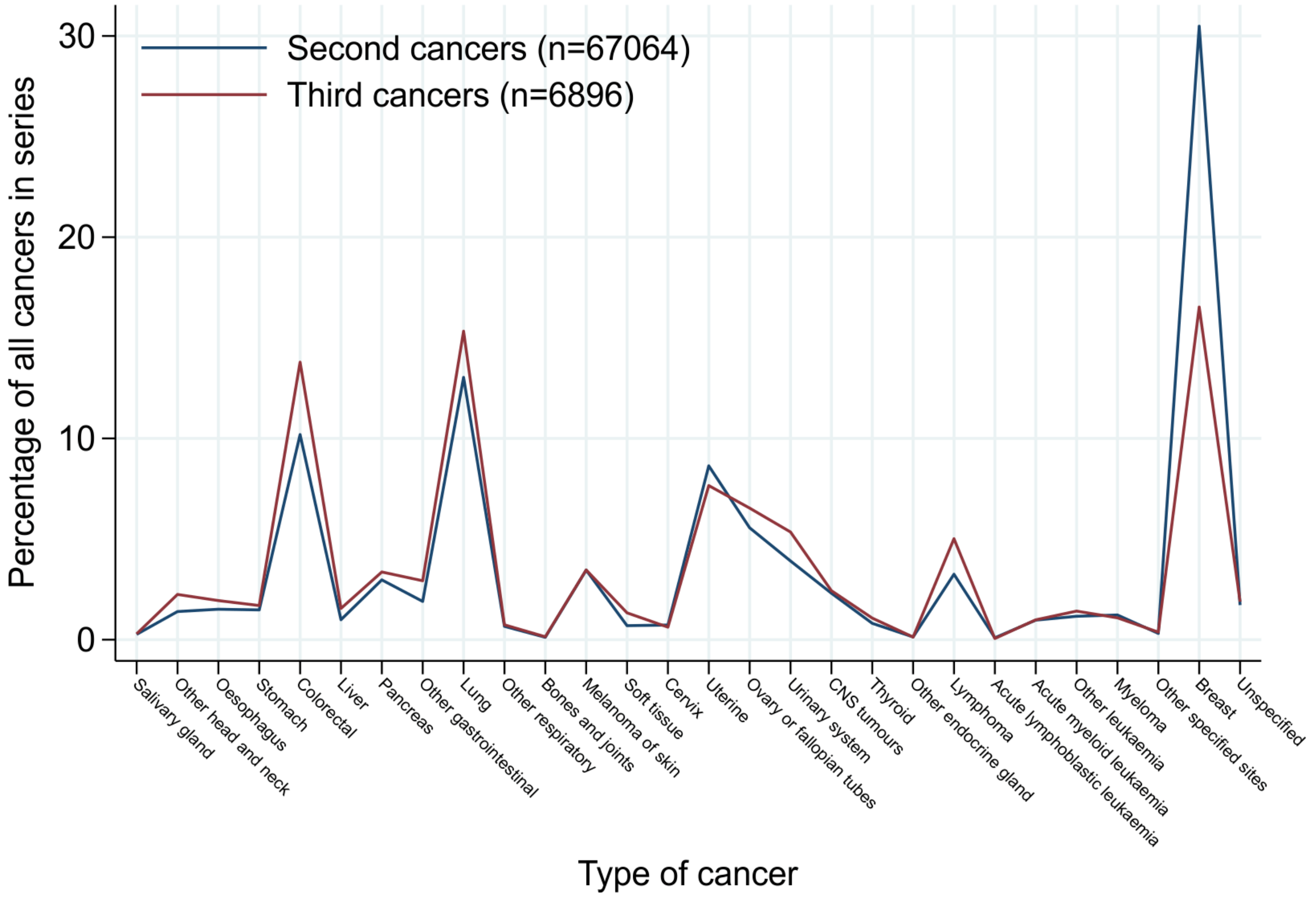

b) Non-invasive cancers

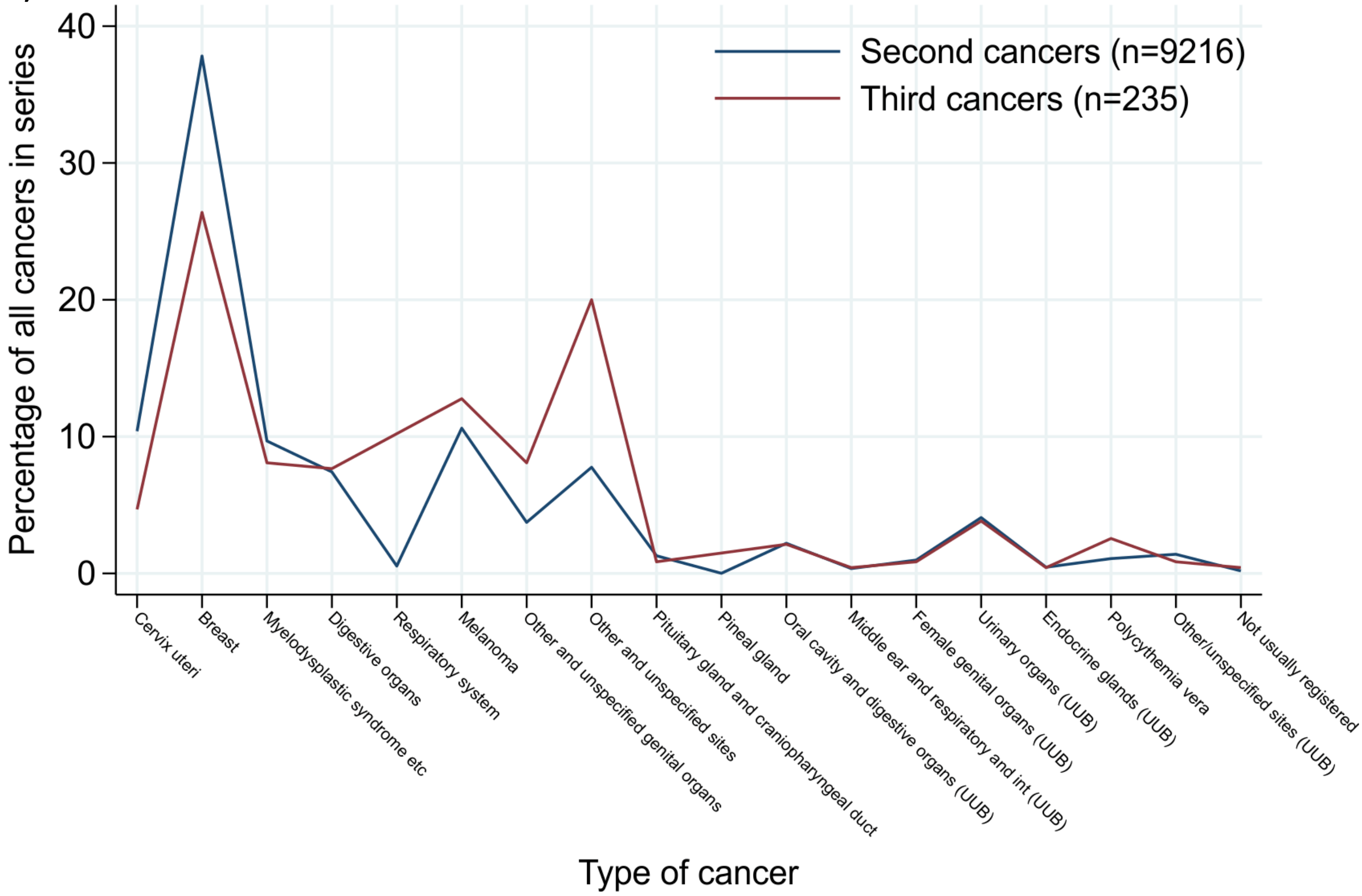

Figure S1: Distribution of second and of third cancers for a) invasive, b) non-invasive cancers following breast cancer as the first cancer in 476,373 women. Abbreviations: UUB=Neoplasm of uncertain or unknown behaviour. See Table S1 for definitions of cancer types.

Cancer and number of positive nodes	Number of cancers/women	Rate ratio (95% CI)
**Lung (p=0.05)**		
0	2803/171886	1.00 (1.00, 1.00)
1 to 3	1107/80497	0.91 (0.85, 0.98)
4 to 9	265/23031	0.91 (0.80, 1.04)
10 or more	99/10098	0.98 (0.80, 1.20)
Unknown	4467/190861	1.04 (0.97, 1.11)
**CNS tumours (p=0.06)**		
0	520/171886	1.00 (1.00, 1.00)
1 to 3	235/80497	1.03 (0.87, 1.20)
4 to 9	66/23031	1.17 (0.90, 1.53)
10 or more	29/10098	1.49 (1.01, 2.18)
Unknown	699/190861	0.99 (0.85, 1.15)
**Liver (p=0.95)**		
0	239/171886	1.00 (1.00, 1.00)
1 to 3	85/80497	0.85 (0.65, 1.10)
4 to 9	19/23031	0.81 (0.50, 1.31)
10 or more	14/10098	1.71 (0.98, 2.98)
Unknown	308/190861	0.83 (0.66, 1.04)
**Bones and joints (p=0.20)**		
0	24/171886	1.00 (1.00, 1.00)
1 to 3	16/80497	1.44 (0.74, 2.79)
4 to 9	6/23031	2.21 (0.86, 5.69)
10 or more	1/10098	1.04 (0.14, 7.99)
Unknown	32/190861	1.01 (0.51, 2.00)

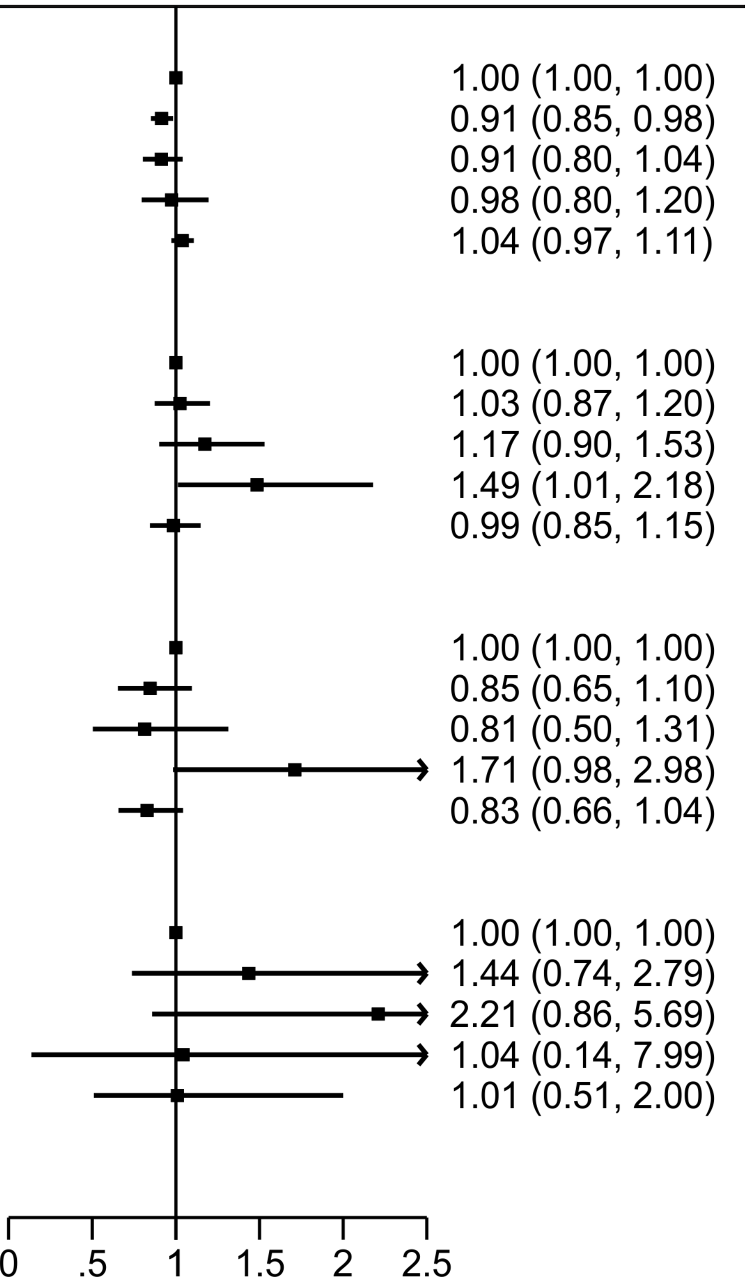

Figure S2: Adjusted rate ratios (and p for trend) of second cancers known to be common sites of metastatic breast cancer by number of nodes to which the index breast cancer had spread. A strong trend may indicate the cancer is largely composed of misclassified metastatic breast cancer rather than a new primary at that site. Rate ratios are adjusted for age at breast cancer diagnosis, whether screen detected, tumour size, grade, focality and quadrant, ER status, type of breast cancer, IMD, geographical region, year of breast cancer diagnosis and years since. See Table S1 for definition of cancer types.

Abbreviations: CNS=central nervous system, ER=oestrogen-receptor, IMD=index of multiple deprivation

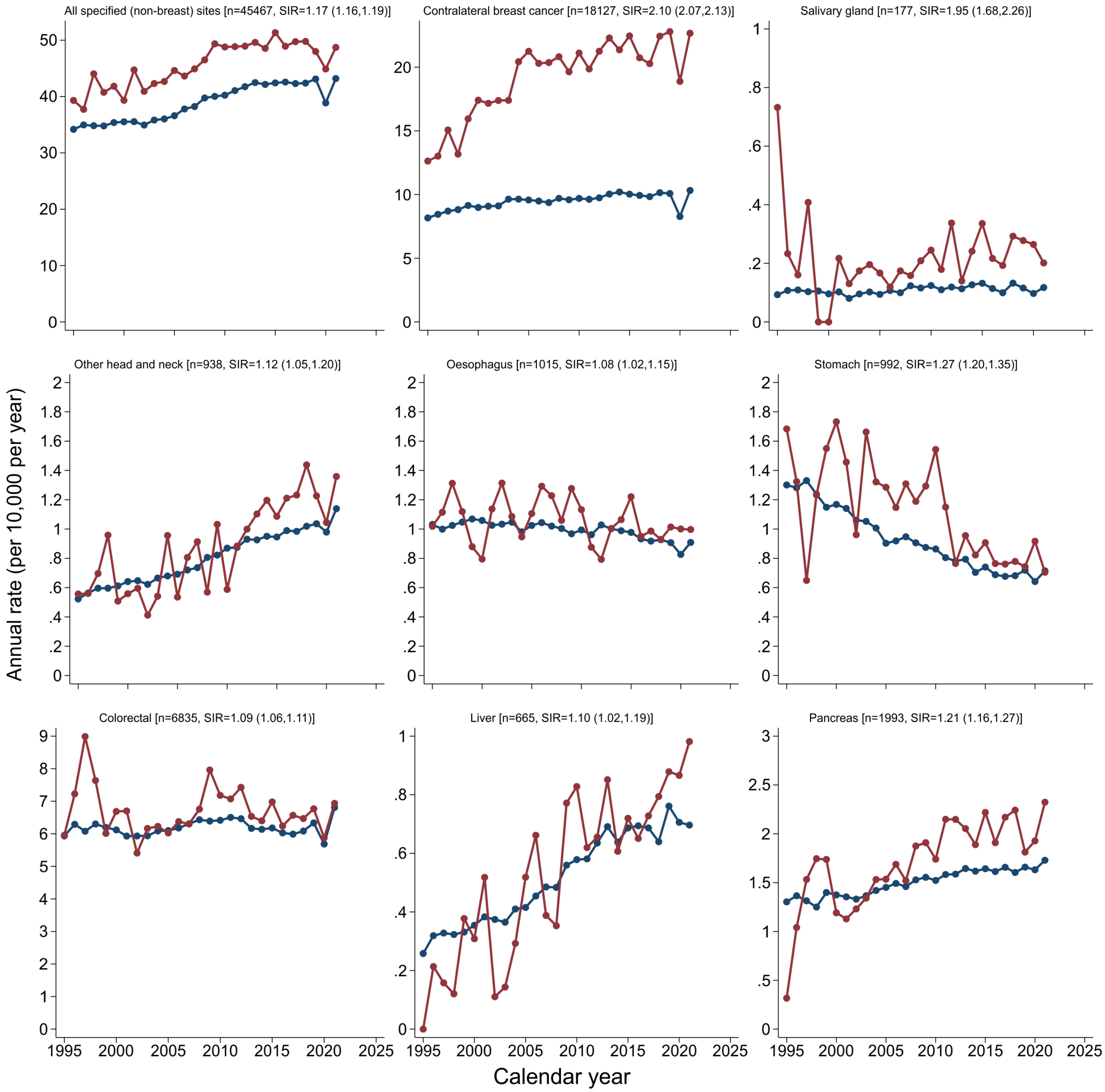

Figure S3: Study population (red lines) and national (blue lines) average age and quintile of deprivation index adjusted second invasive cancer incidence rates (per 10,000 per year) by current calendar year. Both the 1993 and 1994 years are omitted from the plots to a allow events to accrue in the cohort (but for continuity with other analyses, are included in the estimate of the SIR). Confidence intervals are omitted for readability. In order to compare the incidence of contralateral breast cancer, national rates are halved. See Table S1 for definitions of cancer types.

(continued on next page)

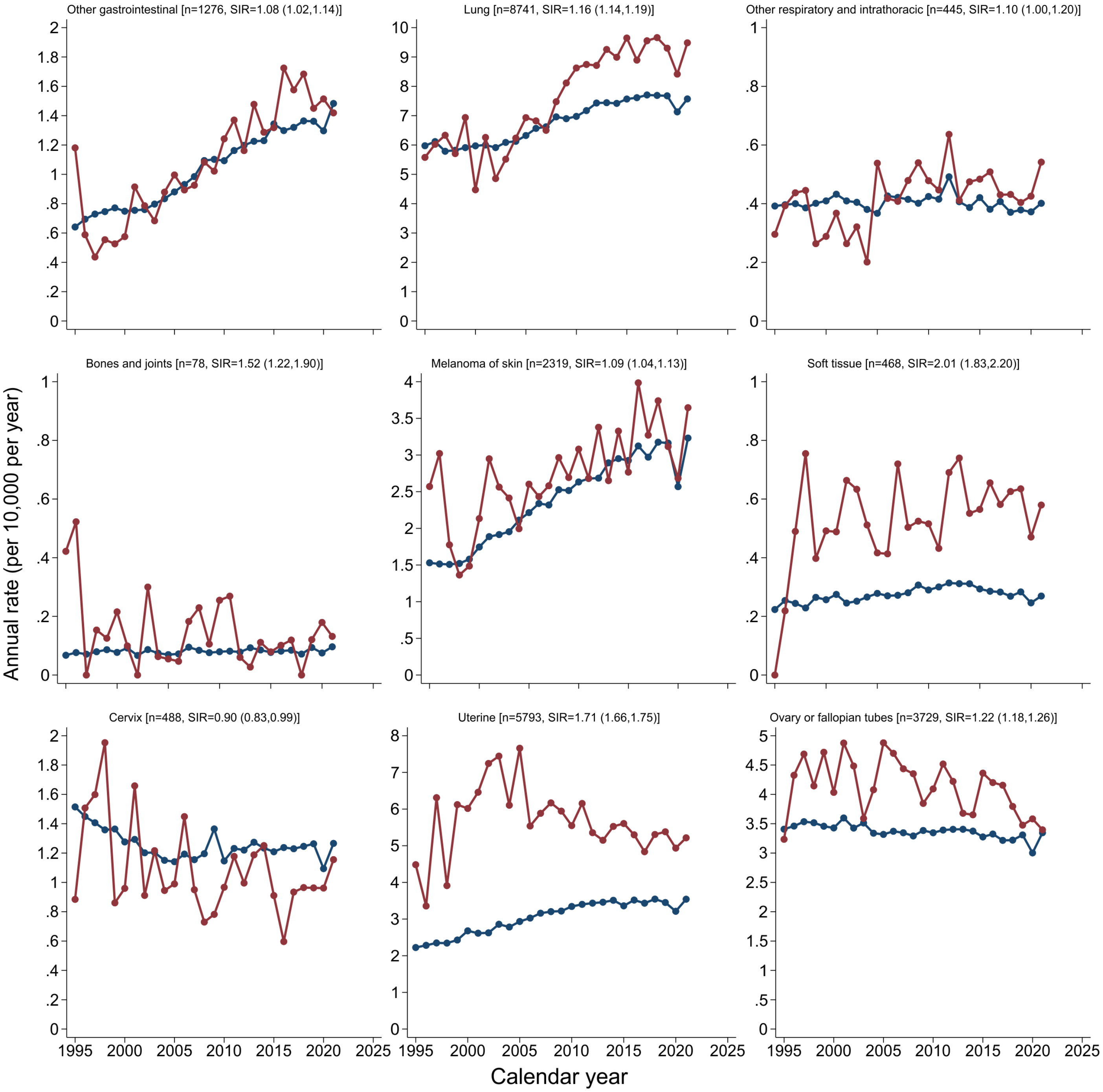

Figure S3 (continued)

(continued on next page)

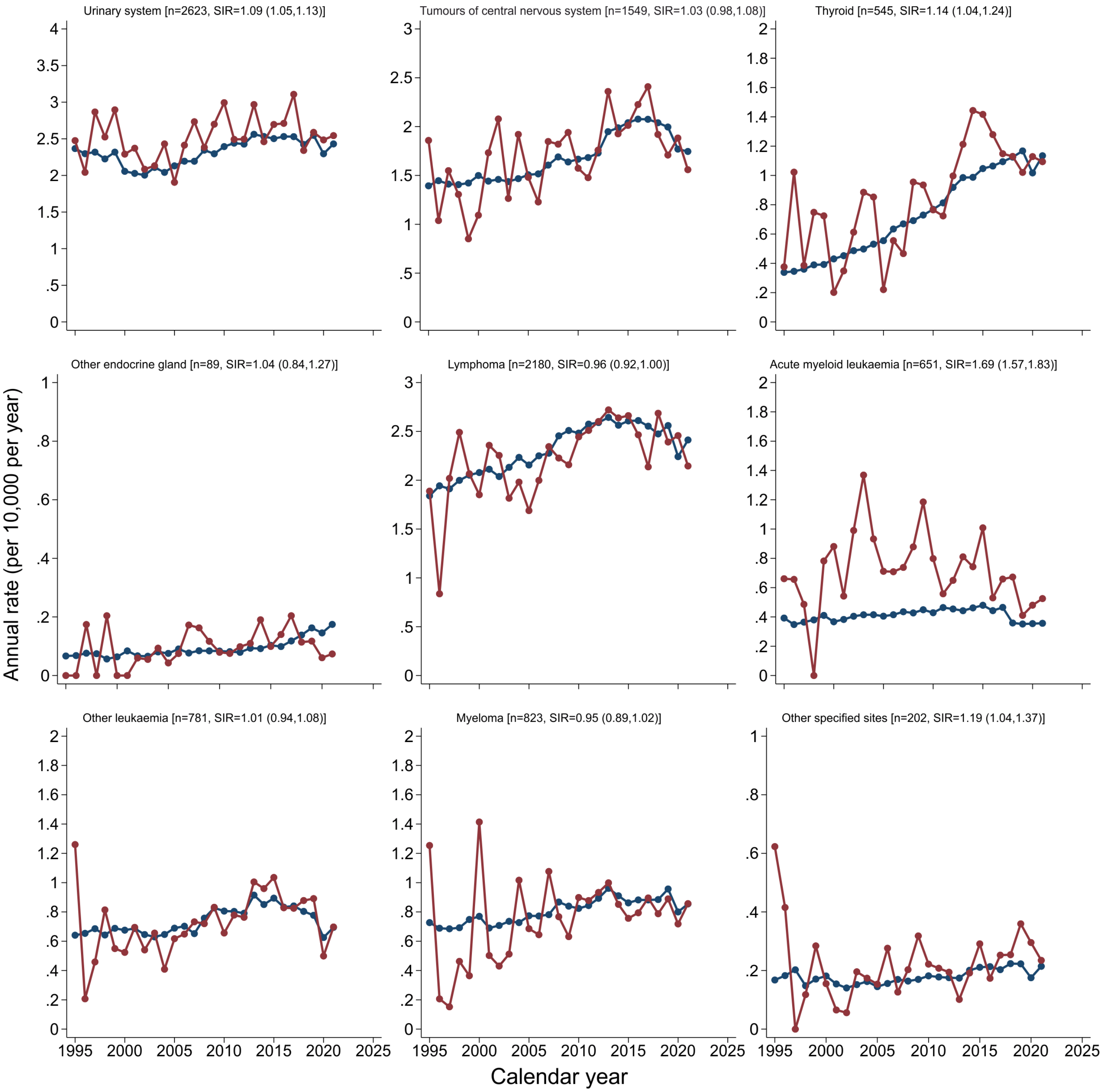

Figure S3 (continued)

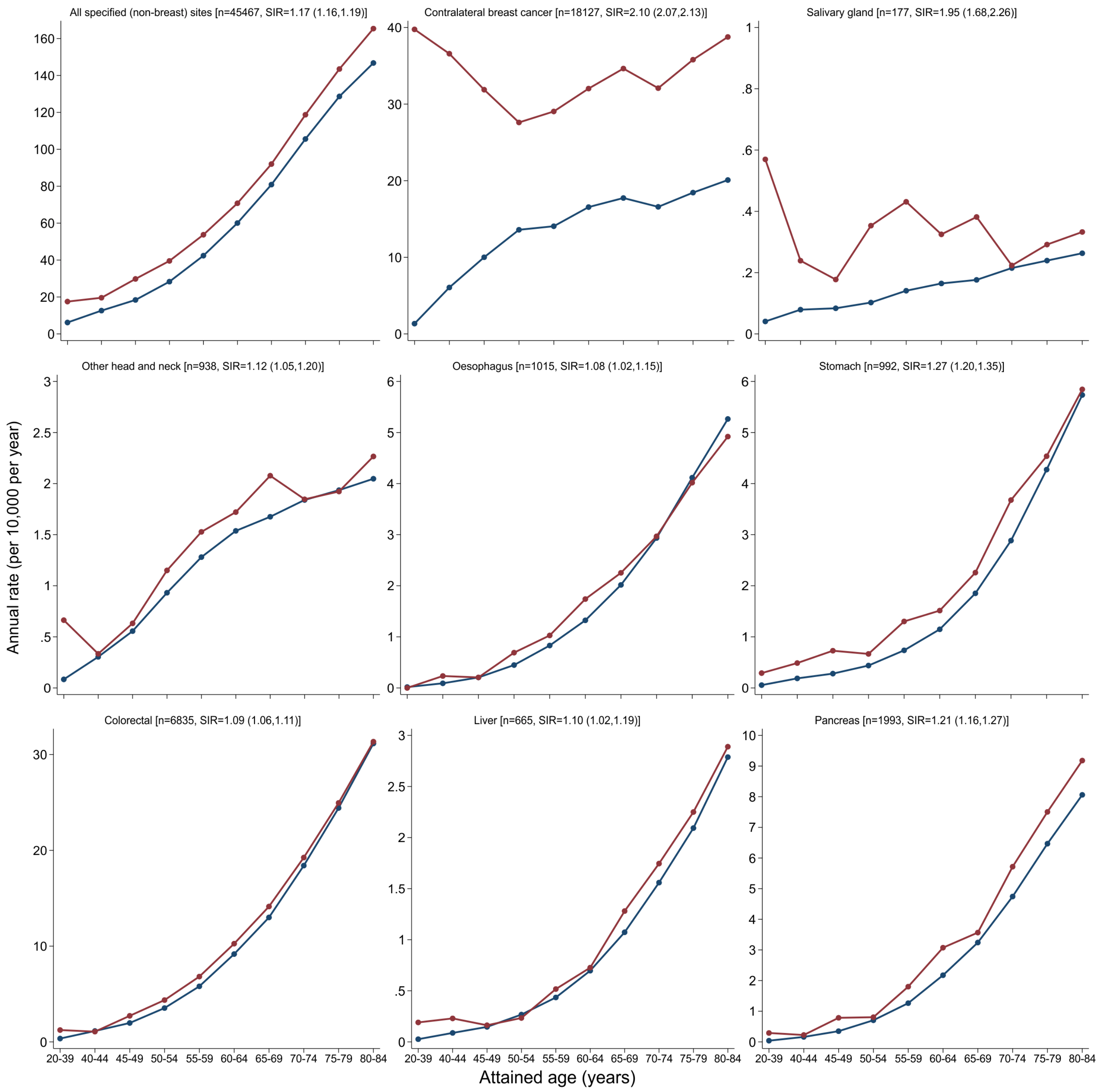

Figure S4: Study population (red lines) and national (blue lines) average current calendar year and quintile of deprivation index adjusted invasive second cancer incidence rates (per 10,000 per year) by attained age. Confidence intervals are omitted for readability. In order to compare the incidence of contralateral breast cancer, national rates are halved. See Table S1 for definition of cancer types.

(continued on next page)

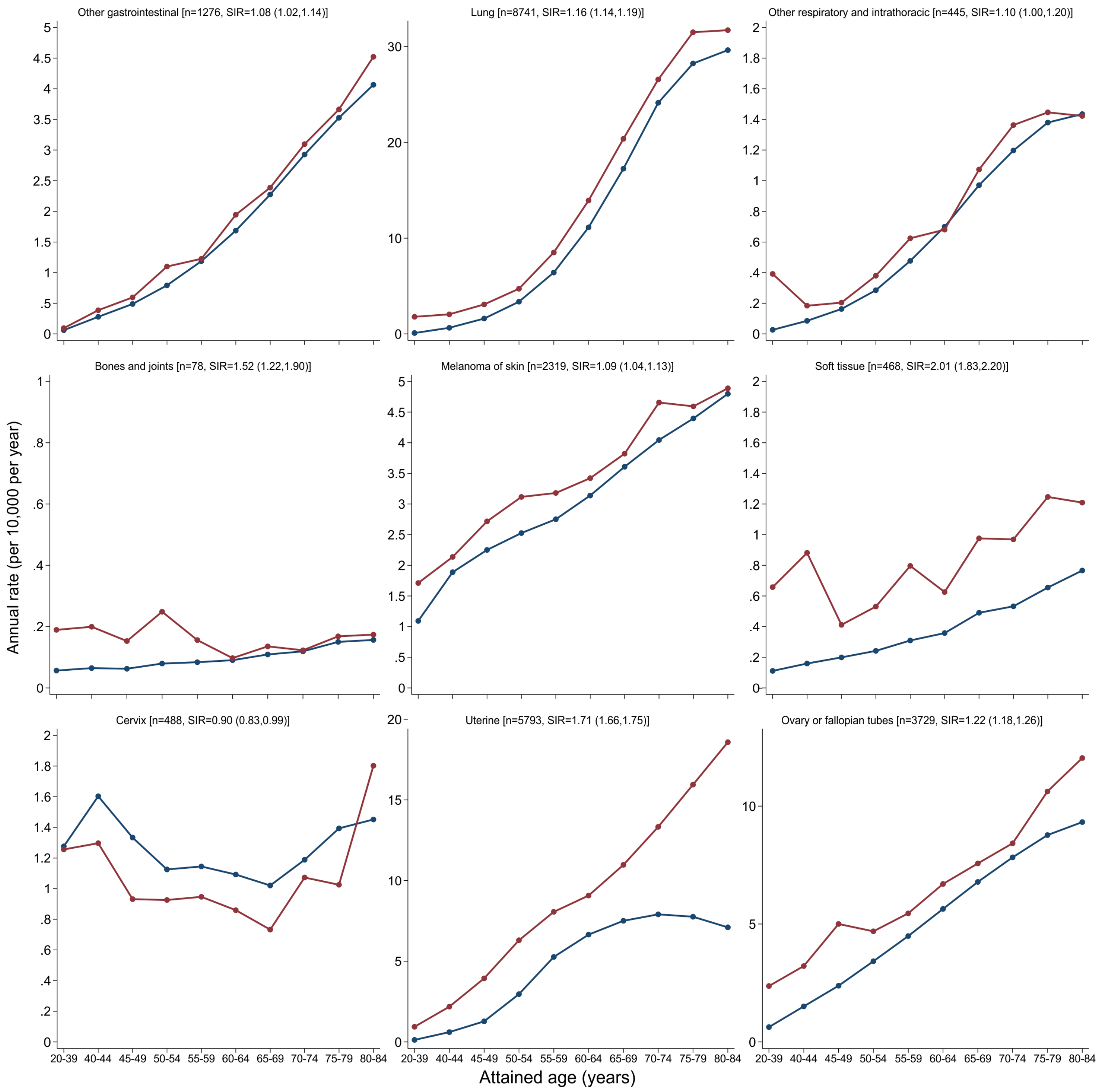

Figure S4 (continued)

(continued on next page)

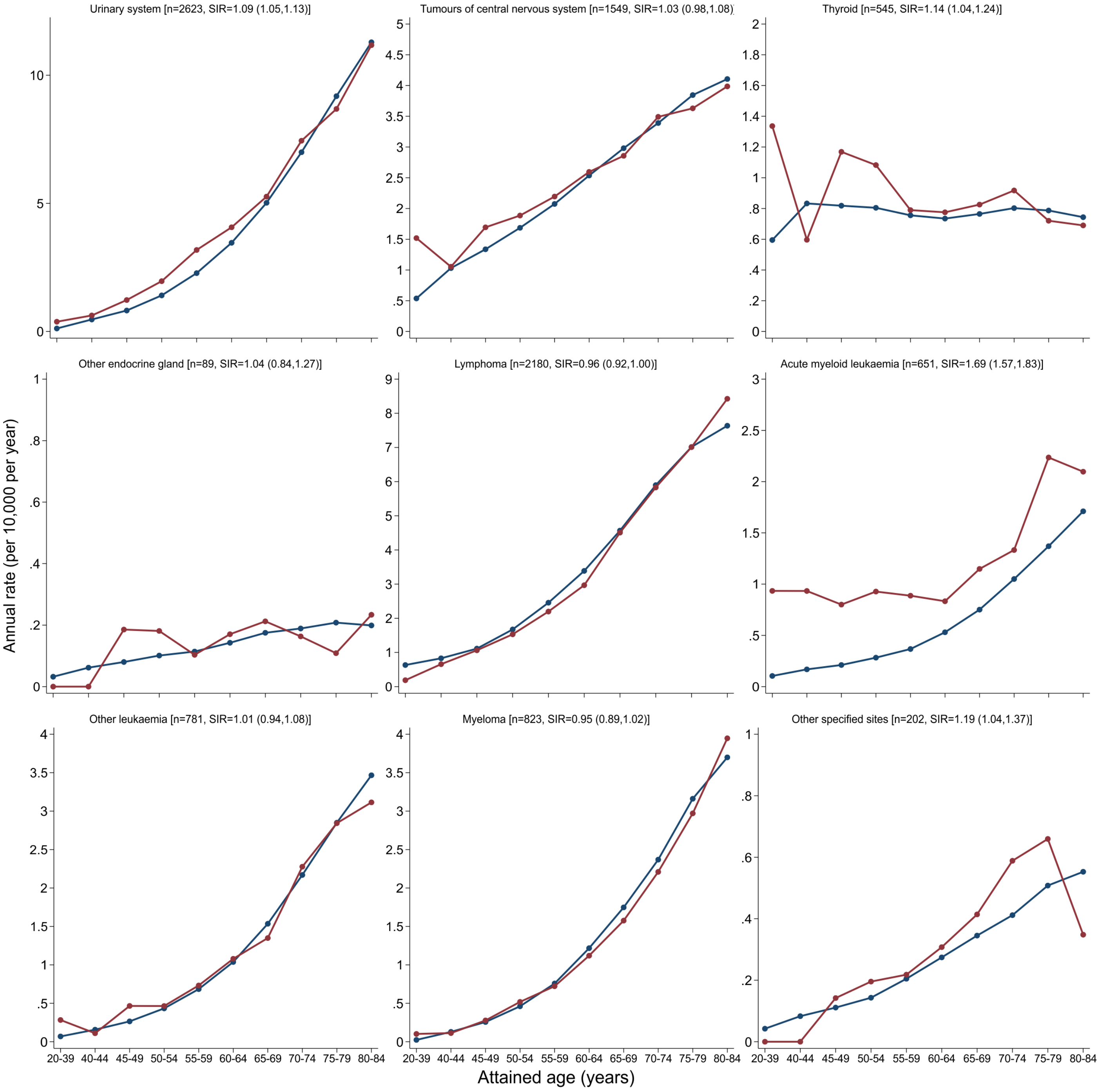

Figure S4 (continued)

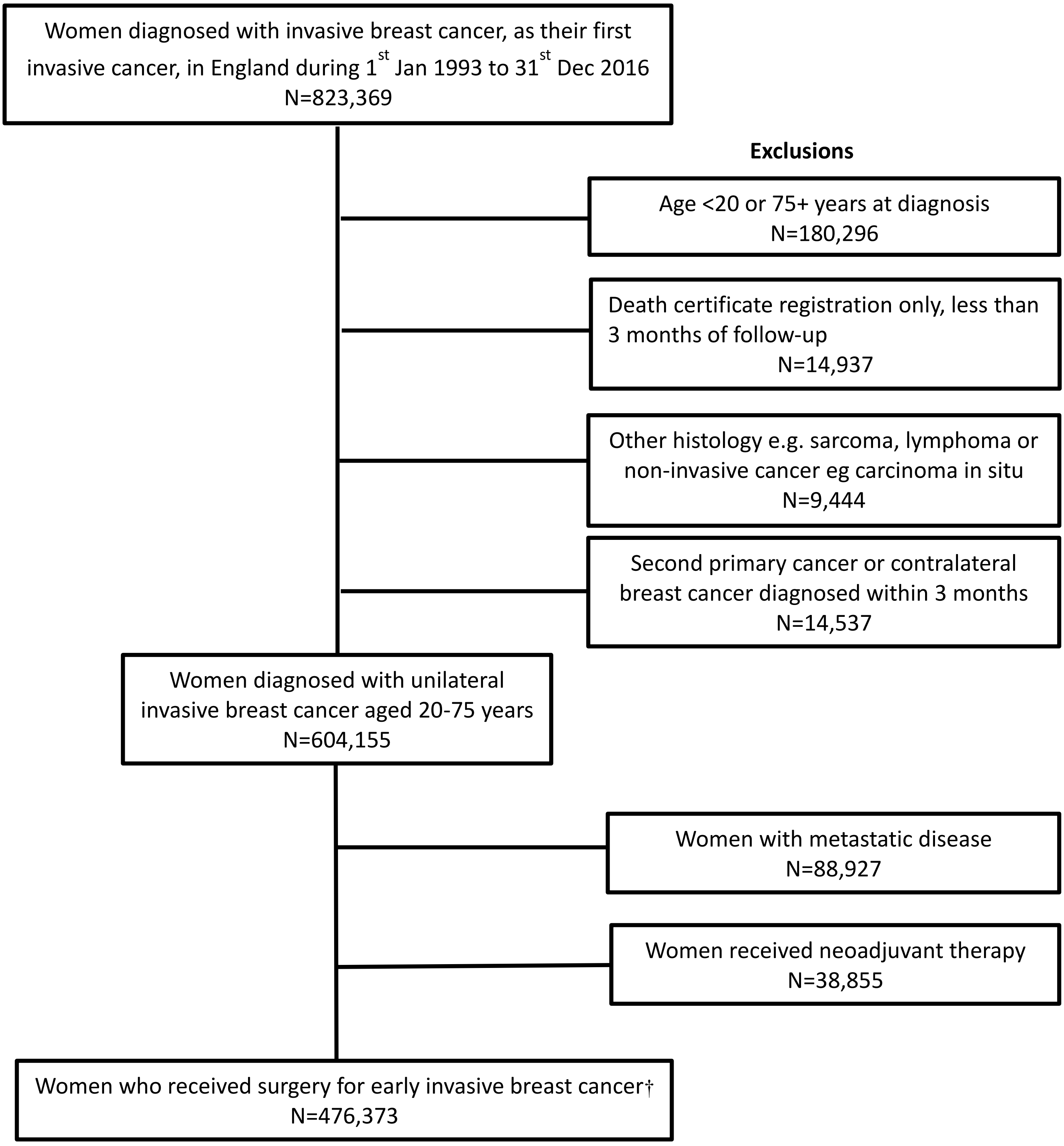

Figure S5: Composition of study population.

Text S2: Statistical methods

1. Standardised incidence ratios (SIR) and absolute excess rates (AER) (Figures 1,2, S3, S4, S6-S9, S20. Tables S3-S8.)

The number of registered cancers (by 4 character ICD9/10 code) in the population of England was available by sex, individual calendar year, age in 5 year groups, and index of multiple of deprivation (IMD). Population counts were also available broken down by these same factors. This enabled national cancer rates to be calculated. Applying the woman-years in the study population stratified by calendar year, attained age, and IMD quintile to the national rates gives the expected number of cancers in each stratum, e, which can be compared to the observed number of cancers, o. Summing o and e over all strata and then dividing gives the standardised incidence ratio SIR=O/E for each cancer category (see Table S1) under consideration. Poisson regression was used to derive the 95% confidence intervals.

The absolute excess was expressed via the absolute excess rate, AER per 10,000 women; AER=10,000*(O-E)/woman-years. Since the variance of the SIR is derived on the log scale, and that for the AER on the linear scale, the confidence limits of the SIR were used to derive the confidence intervals for the AER as this ensures the AER CI will cross 0 only if the CI for the SIR crosses 1. Therefore, the confidence limits for the AER were defined as 10,000*E*(SIR_[lower or upper CI]-1)/woman-years.

The primary analysis was time to second cancer. However, as outlined in Text S1, some women also had third, fourth cancers etc. Therefore, SIRs and AERs for subsequent cancers at any time were also calculated and compared to those derived when considering just the second cancer as check on whether interpretation depended on restricting to just the second cancer or not.

A simple comparison of the at-risk time for these two types of analyses is as follows:

i) Second cancer only

Time of BCa (plus 3 months) ----------> time of 2nd cancer

ii) Second and further cancers

Time of BCa (plus 3 months) ----------> time of 2nd cancer--------> time of 3rd cancer------> end of follow-up

In i), each woman contributes woman-years from 3 months after breast cancer diagnosis until the time of second cancer. In ii), each woman contributes woman-years from 3 months after breast cancer diagnosis until the end of follow-up. Follow-up time is divided amongst the subsequent cancers with the division being at the dates of the cancers.

For non-invasive cancers, censoring occurs at any prior invasive cancer. Non-invasive cancer events did not censor subsequent invasive cancer events. For all situations, censoring occurs due to death or reaching 85th birthday.

2. Cumulative risk (Figures 1, 2, 3. Tables S3-S7.)

This was estimated for each category of second cancer, for each year since diagnosis of the index breast cancer (plus 3 months) to the end of follow-up. Account was taken of the competing risks of any invasive cancer event and death.

Follow-up was split into individual follow-up years and for each of these years the rate of the cancer of interest calculated. This gave the probability of suffering that cancer just in that follow-up year. The cumulative probability of not suffering any cancer event or death up to the start of each follow-up year (taken as 1 for the first follow-up year) was also calculated. These two probabilities were then multiplied together and summed over follow-up year to give the cumulative risk by years since index breast cancer diagnosis. Confidence intervals were estimated following the method outlined in Ramroth et al 2023.

To derive expected cumulative risk curves, the national cancer rates were subjected to the same competing risks as the study cohort. This maintained agreement between the SIRs and any differences between the "observed" and "expected" cumulative risks ie the ratio of these two cumulative risks will be approximately equal to the SIR.

3. Regression models (Figures 4, S2-S4, S10-S20.)

Variation in cancer rates by patient and tumour factors

Poisson regression was used to compare temporal patterns in cancer rates in the study cohort with those in the general population (see figures S3, S4). This was mainly for the purposes of checking data consistency. Here average adjusted rates were derived by considering current calendar year, attained age, and index of deprivation in the regression. The offset in the model was the woman-years (see next paragraph) for the cohort and the population counts for the national data. Coefficients from the model were combined with the offset values (weighted by the sum of the offsets) to derive average rates (using the Stata *margins* command).

Poisson regression was also employed to investigate variation of cancer rates between patient and between tumour subgroups within the cohort i.e. without reference to the general population (see figures S10-S12, S20). All factors (see Table 1) were adjusted for each other. Woman-years were taken to be the time from 3 months after diagnosis of the index breast cancer to the earliest of any cancer event, 85th birthday, death or 31st October 2021. Adjusted rate ratios varied little by whether adjustment included treatment or not.

Variation in cancer rates by recorded anti-cancer treatment

Assessment of the effect of treatment on outcome outside the setting of a randomised trial or available instrumental variables can be severely confounded by indication bias eg where patient health determines whether they receive treatment or not. Meta-analyses of randomised trials of breast cancer treatments have revealed serious side-effects which appear to be causal eg an increase in heart disease due to chemotherapy (EBCTCG 2012) and an increase in lung cancer due to radiotherapy (EBCTCG 2017).

To derive guidelines for the interpretation of results from regressions applied to the present cohort, mortality outcomes from randomised trials shown to be related to adjuvant treatment effects, and shown not to be related, were modelled using increasingly complex Poisson regressions and also various propensity matching techniques. The outcome suggested the following approach to modelling and interpreting treatment associated risks:

- Examine how the use of treatments varies by patient and tumour factors (via logistic regressions with treatment use as the outcome, see figures S13-S15)
- Execute a Poisson model with cancer as the outcome and the treatments as the exposure variables stratified by age (in 5- rather than 10-year groups), year of follow-up, year of index breast cancer diagnosis, and IMD quintile
- Examine the unadjusted and fully adjusted rate ratios, both overall and separately in the follow-up period 0-9 years and 10+ years after diagnosis of the index cancer
- Consider rate ratios (RR) greater than 1, and p values <0.01 (exception is contralateral after endocrine therapy as RR to <1 is to be expected)

However, any excesses (see figures 4, S16-S18) could still be due to indication bias, common susceptibility to genetic causes, common susceptibility to environmental exposure, or just chance. The one exception to this is a case-only analysis of lung cancer by concordance with the index breast cancer since each woman acts as her own control, here identified associations are more likely to be causal. A logistic model was used to estimate the relative risk of concordance by the calendar year in which the index cancer was diagnosed and follow-up year, separately for those for whom use of radiotherapy (or BCS) was recorded versus not (see figure S19).

Calculation of the number of excess second cancers associated with recorded anti-cancer treatment

We estimated the fraction of second cancers in the study cohort that were associated with the use of adjuvant treatments by applying the familiar formula for the attributable fraction in the exposed (AFE), (RR-1)/RR where RR is the rate ratio for the second cancer and adjuvant treatment, recorded versus not, under study. To derive the numbers of excess second cancers of each type associated with each adjuvant treatment the AFE needs to be multiplied by the total number of second cancers of that type and the proportion of use (ie the prevalence) of the adjuvant treatment associated with the excess.

Figures S13-S15 show the reporting of adjuvant treatments in the cohort. The prevalence of both radiotherapy (75%) and chemotherapy (35%) are in line with clinical expectation, but the reporting of endocrine therapy (55%) is below this. However, there are 3 cancer registries (Eastern, Northern & Yorkshire, and West Midlands) who appear to have reported endocrine use at the levels expected (70+%), so these are utilised to supply the prevalences of each of the adjuvant treatments. Given the misclassification of endocrine use, these 3 registries can also be used to estimate the likely dilution of the RRs for endocrine reported versus not and so the correction factor (CF) which needs to be applied to the RRs for endocrine therapy.

Hence, the excess number of second cancers (X) of type c, associated with adjuvant treatment t can be written as:

$$X_{ct} = N_c \text{ x prevalence(t) x } (CF_{ct} \text{ x } RR_{ct} -1)/(CF_{ct} \text{x} RR_{ct})$$

Since contralateral cancer is affected by both radiotherapy and endocrine therapy the relevant prevalences are those that had radiotherapy recorded but not endocrine therapy and those that didn't have radiotherapy recorded but had endocrine therapy recorded. The prevalence for the recording of both is not required as their RR was found to be 1.

CF is 1 when calculating excess second cancers associated with radiotherapy and with chemotherapy

If the RR<1 then the reciprocal is taken in the formula above

These excess numbers were summed across the second cancers and adjuvant treatments with significant RRs depending on whether they were consistent with effects found in randomised trials or not (Table S2). This sum can be expressed as either a fraction of the total number of second cancers or as a fraction of the excess from the SIR analyses ie the number O-E from section 1 above. (Analytic code is provided in Appendix 2)

References

Ramroth J, Shakir R, Darby SC, Cutter DJ, Kuan V. Cardiovascular disease incidence rates: a study using routinely collected health data. *Cardiooncology*. 2023;9(1):41. Published 2023 Nov 15. doi:10.1186/s40959-023-00189-8

EBCTCG. Comparisons between different polychemotherapy regimens for early breast cancer: meta-analyses of long-term outcome among 100,000 women in 123 randomised trials. Lancet 2012; 379: 432-44, DOI:10.1016/S0140-6736(11)61625-5

EBCTCG. Estimating the risks of breast cancer radiotherapy: evidence from modern radiation doses to the lungs and heart and from previous randomized trials. J Clin Oncol 2017; 35: 1641-49, DOI: 10.1200/JCO.2016.72.0722

Table S1: Definitions of cancer categories by ICD 9 and 10 codes.

ICD-9	ICD-10	Description of cancer
140, 141, 143-149	C00-C06, C09-C14	Other head and neck
142	C07-C08	Salivary gland
150	C15	Oesophagus
151	C16	Stomach
152, 154.2, 154.3, 154.8, 156, 158, 159.8, 159.9	C17,C21, C23, C24, C26.8-C26.9, C45.1, C48	Other gastrointestinal
153, 154.0, 154.1, 159.0	C18-C20, C26.0	Colorectal
155	C22	Liver
157	C25	Pancreas
160, 161, 162.0, 163, 164.2-164.3, 164.8-164.9, 165	C30-C32, C33, C38.1-C38.9, C39, C45.0	Other respiratory and intrathoracic
162.2-162.5, 162.8-162.9	C34	Lung
170	C40, C41	Bones and joints
172	C43	Melanoma of skin
164.1, 171	C47, C49, C38.0, C45.2, C45.7, C45.9	Soft tissue
174	C50	Invasive breast
180	C53	Cervix
179, 182	C54, C55	Uterine
181, 183, 184	C51, C52, C56-C58	Ovary or fallopian tubes
185-189	C60-C68	Urinary system
191-192, 225, 237.5, 237.6, 237.9, 239.6	C70-C72, D32, D33, D42, D43	Central nervous system
193	C73	Thyroid
164.0,194	C37, C74-C75	Other endocrine gland
200,201, 202.0-202.2, 202.8-202.9	C81-C86, C96.3	Lymphoma
204.0	C91.0	Acute lymphoblastic leukaemia
205.0	C92.0, C92.4, C92.5, C92.6, C92.8	Acute myeloid leukaemia
202.4, 204.1-204.9, 205.1-205.9, 206-208	C91.1-C91.9, C92.1, C92.2, C92.3, C92.7, C92.9, C93, C94, C95	Other leukaemia
203.0,203.1, 238.6	C90	Myeloma
159.1, 176, 190, 202.3, 202.5, 202.6, 203.8	C26.1,C46, C69, C88, C94.6, C96.0-C96.2, C96.4-C96.9,	Other specified sites
195-199	C76-C80, C97	Malignant neoplasm of unspecified/multiple sites
230	D00, D01	CIS of digestive organs
231	D02	CIS of respiratory system
232	D03	Melanoma in situ
233.0	D05	CIS of breast
233.1	D06	CIS of cervix uteri
233.3,233.7, 233.9	D07	CIS of other/unspecified genital organs
234	D09	CIS of other/unspecified sites
227.3	D35.2, D35.3	CIS of pituitary gland and craniopharyngeal duct
227.4	D35.4	CIS of pineal gland
235.0-235.5	D37	UUB of oral cavity and digestive organs
235.6-235.9	D38	UUB of middle ear, respiratory and intrathoracic organs
236.0-236.3	D39	UUB of female genital organs
236.7, 236.9	D41	UUB of urinary organs
237.4	D44	UUB of endocrine glands
238.4	D45	Polycythemia vera
238.7	D46, D47	Myelodysplastic syndrome etc
237.0-237.4, 237.7, 238.0-238.3, 238.5, 238.7-238.9, 239.0-239.5, 239.7-239.9	D48	UUB of other/unspecified sites
210-224,226-229	D10-D31, D34, D35.0, D35.1, D35.5-D35.9, D36	Benign cancers not fully covered in registries

* Any occurrence of C44 or D04 was ignored.

Abbreviations: CIS=Carcinoma in situ, UUB=Neoplasm of uncertain or unknown behaviour

Table S2: Rate ratios from randomised trials for the effects of adjuvant breast cancer treatments on individual causes of second cancers

Treatment type and reference	Second cancer type	Rate ratio (95% CI)
**Radiotherapy vs not (any surgery)**		
EBCTCG 2017[1]	Contralateral breast	1.20 (1.08, 1.33)
EBCTCG 2017[1]	Lung (10 years post treatment)	2.10 (1.48, 2.98)
EBCTCG 2017[1]	Oesophageal	2.42 (1.19, 4.92)
EBCTCG 2017[1]	Leukaemia	1.71 (1.05, 2.79)
**Endocrine therapy (5 years of tamoxifen vs not) in ER+ disease**		
EBCTCG 2011[2]	Contralateral breast*	0.62 (0.52, 0.73)
EBCTCG 2011[2]	Endometrial	2.40 (1.62, 3.54)
**Chemotherapy (anthracycline vs not, both with taxanes)**		
EBCTCG 2023[3]	Acute myeloid leukaemia	3.90 (1.34, 11.36)

Note: *Protective effect

References

1. EBCTCG. Estimating the risks of breast cancer radiotherapy: evidence from modern radiation doses to the lungs and heart and from previous randomized trials. Journal of Clinical Oncology. 2017 May 20;35(15):1641-9
2. EBCTCG. Relevance of breast cancer hormone receptors and other factors to the efficacy of adjuvant tamoxifen: patient-level meta-analysis of randomised trials. Lancet 2011a;378:771-84.
3. EBCTCG. Anthracycline-containing and taxane-containing chemotherapy for early-stage operable breast cancer: a patient-level meta-analysis of 100 000 women from 86 randomised trials. The Lancet. 2023 Apr 15;401 (10384):1277-92.

ANALYSIS OF INVASIVE CANCERS

Part a: Comparisons with the general population (i.e. national rates)

Table S3: Non-breast cancer standardised incidence ratios (SIR), absolute excess rates (per 10,000) (AER), and cumulative risks (%) by age (years) at index breast cancer (BCa) and attained age. All measures are calculated for the intervals from age at BCa diagnosis to attained age. Cohort rates are compared to all England rates for the same calendar year, attained age and quintile of deprivation.

Age at index BCa	Measure	Attained age (years)											
		30	35	40	45	50	55	60	65	70	75	80	85
<30	SIR (95% CI)	3.27 (1.70,6.29)	2.88 (2.03,4.10)	2.34 (1.73,3.17)	2.22 (1.69,2.90)	1.98 (1.53,2.56)	1.98 (1.54,2.53)	1.95 (1.52,2.49)					
	AER (95% CI)	12.20 (3.77,28.39)	12.86 (7.01,21.17)	10.57 (5.75,17.09)	10.79 (6.15,16.87)	9.61 (5.16,15.37)	10.27 (5.72,16.09)	10.06 (5.53,15.86)					
	Risk obs (95% CI)	0.87 (0.44,1.61)	1.71 (1.14,2.48)	2.24 (1.59,3.06)	3.00 (2.22,3.94)	3.44 (2.57,4.49)	4.92 (3.57,6.58)	4.92 (3.56,6.59)					
	Risk exp	0.27	0.57	0.92	1.33	1.87	2.63	3.61					
30	SIR (95% CI)		2.14 (1.40,3.29)	1.87 (1.49,2.34)	1.68 (1.40,2.01)	1.73 (1.49,2.01)	1.71 (1.50,1.96)	1.72 (1.51,1.95)	1.70 (1.50,1.93)				
	AER (95% CI)		8.04 (2.80,16.09)	7.60 (4.31,11.73)	6.99 (4.15,10.39)	8.79 (5.92,12.12)	9.74 (6.81,13.08)	10.49 (7.50,13.89)	10.40 (7.40,13.79)				
	Risk obs (95% CI)		0.75 (0.48,1.13)	1.45 (1.12,1.86)	2.11 (1.73,2.56)	3.23 (2.75,3.77)	4.57 (3.95,5.25)	6.47 (5.53,7.50)	7.32 (5.56,9.39)				
	Risk exp		0.35	0.75	1.21	1.81	2.63	3.73	5.13				
35	SIR (95% CI)			2.12 (1.67,2.69)	1.68 (1.47,1.91)	1.69 (1.54,1.87)	1.66 (1.53,1.80)	1.58 (1.46,1.70)	1.57 (1.46,1.68)	1.56 (1.46,1.68)			
	AER (95% CI)			10.02 (5.97,15.16)	7.89 (5.48,10.64)	9.82 (7.61,12.26)	11.24 (9.05,13.62)	11.12 (8.94,13.48)	11.55 (9.33,13.93)	11.57 (9.35,13.96)			
	Risk obs (95% CI)			0.95 (0.74,1.19)	1.79 (1.54,2.06)	2.98 (2.68,3.30)	4.50 (4.13,4.89)	6.06 (5.59,6.56)	8.39 (7.65,9.18)	10.89 (8.77,13.26)			
	Risk exp			0.45	1.00	1.69	2.65	3.93	5.57	7.52			
40	SIR (95% CI)				1.59 (1.35,1.88)	1.61 (1.49,1.75)	1.52 (1.43,1.61)	1.43 (1.36,1.51)	1.39 (1.33,1.46)	1.39 (1.33,1.45)	1.39 (1.32,1.45)		
	AER (95% CI)				7.34 (4.33,10.90)	10.24 (8.19,12.46)	10.76 (8.93,12.71)	10.72 (8.94,12.59)	10.85 (9.06,12.72)	11.26 (9.46,13.16)	11.26 (9.45,13.15)		
	Risk obs (95% CI)				0.98 (0.83,1.15)	2.31 (2.11,2.51)	3.88 (3.64,4.13)	5.76 (5.46,6.07)	8.08 (7.67,8.50)	11.12 (10.46,11.79)	13.93 (12.25,15.72)		
	Risk exp				0.62	1.44	2.56	4.08	6.03	8.31	10.91		
45	SIR (95% CI)					1.59 (1.43,1.76)	1.43 (1.36,1.51)	1.40 (1.35,1.46)	1.35 (1.31,1.40)	1.33 (1.29,1.38)	1.32 (1.28,1.36)	1.32 (1.28,1.36)	
	AER (95% CI)					10.68 (7.78,13.90)	10.89 (9.02,12.87)	12.45 (10.74,14.22)	12.69 (11.03,14.42)	13.17 (11.50,14.90)	13.32 (11.64,15.05)	13.41 (11.73,15.15)	
	Risk obs (95% CI)					1.43 (1.29,1.59)	3.19 (3.01,3.38)	5.50 (5.28,5.73)	8.17 (7.89,8.47)	11.35 (10.97,11.74)	14.78 (14.22,15.36)	19.37 (17.67,21.13)	
	Risk exp					0.91	2.18	3.88	6.07	8.67	11.62	14.75	
50	SIR (95% CI)						1.39 (1.29,1.49)	1.29 (1.24,1.34)	1.27 (1.23,1.31)	1.26 (1.22,1.29)	1.24 (1.21,1.27)	1.23 (1.20,1.26)	1.23 (1.20,1.26)
	AER (95% CI)						10.88 (8.12,13.85)	10.99 (9.12,12.94)	12.11 (10.41,13.86)	13.09 (11.44,14.79)	13.19 (11.54,14.88)	13.00 (11.35,14.69)	13.00 (11.35,14.69)
	Risk obs (95% CI)						1.93 (1.79,2.07)	4.33 (4.16,4.51)	7.36 (7.14,7.58)	10.95 (10.67,11.24)	14.75 (14.38,15.13)	18.41 (17.85,18.98)	21.99 (20.30,23.73)
	Risk exp						1.39	3.31	5.77	8.73	12.11	15.69	19.00
55	SIR (95% CI)							1.23 (1.15,1.32)	1.17 (1.13,1.21)	1.19 (1.16,1.23)	1.19 (1.16,1.22)	1.19 (1.16,1.21)	1.18 (1.16,1.21)
	AER (95% CI)							9.78 (6.28,13.54)	9.35 (7.07,11.72)	12.28 (10.21,14.40)	13.63 (11.62,15.68)	14.04 (12.04,16.08)	13.92 (11.92,15.95)
	Risk obs (95% CI)							2.56 (2.39,2.74)	5.60 (5.39,5.82)	9.44 (9.18,9.71)	13.70 (13.37,14.03)	18.02 (17.59,18.44)	21.54 (20.94,22.15)
	Risk exp							2.08	4.73	7.89	11.49	15.28	18.68
60	SIR (95% CI)								1.14 (1.07,1.21)	1.11 (1.08,1.15)	1.14 (1.12,1.17)	1.15 (1.12,1.17)	1.14 (1.12,1.17)
	AER (95% CI)								8.22 (4.37,12.31)	8.25 (5.73,10.84)	12.21 (9.92,14.54)	13.44 (11.22,15.70)	13.83 (11.63,16.07)
	Risk obs (95% CI)								3.35 (3.16,3.54)	7.22 (7.00,7.46)	12.01 (11.73,12.30)	16.85 (16.50,17.21)	21.14 (20.69,21.60)
	Risk exp								2.95	6.48	10.51	14.72	18.52
65	SIR (95% CI)									1.04 (0.98,1.10)	1.08 (1.05,1.11)	1.11 (1.08,1.13)	1.11 (1.09,1.14)
	AER (95% CI)									3.19 (-1.35,7.99)	7.84 (4.79,10.98)	11.54 (8.77,14.37)	12.77 (10.08,15.51)
	Risk obs (95% CI)									4.14 (3.92,4.37)	9.07 (8.81,9.35)	14.47 (14.14,14.80)	19.29 (18.88,19.70)
	Risk exp									3.99	8.50	13.19	17.42
70	SIR (95% CI)										1.04 (0.98,1.10)	1.04 (1.01,1.07)	1.06 (1.04,1.09)
	AER (95% CI)										4.21 (-1.81,10.59)	5.12 (1.30,9.05)	8.24 (4.85,11.72)
	Risk obs (95% CI)										5.30 (5.01,5.60)	10.59 (10.26,10.92)	15.53 (15.14,15.92)
	Risk exp										5.10	10.18	14.67

Table S4: Contralateral breast cancer standardised incidence ratios (SIR), absolute excess rates (per 10,000) (AER), and cumulative risks (%) by age (years) at index breast cancer (BCa) and attained age. All measures are calculated for the intervals from age at BCa diagnosis to attained age. Cohort rates are compared to all England rates for the same calendar year, attained age and quintile of deprivation, however, in order to compare incidence of contralateral breast cancer, national rates are halved.

Age at index BCa	Measure	Attained age (years)											
		30	35	40	45	50	55	60	65	70	75	80	85
<30	SIR (95% CI)	93.98 (61.27,144.14)	35.67 (27.86,45.66)	25.70 (21.21,31.15)	18.95 (15.91,22.56)	15.13 (12.81,17.86)	13.81 (11.73,16.25)	13.68 (11.63,16.09)					
	AER (95% CI)	40.54 (26.28,62.42)	38.91 (30.15,50.13)	43.92 (35.93,53.61)	44.30 (36.81,53.22)	44.26 (37.00,52.83)	44.37 (37.18,52.82)	44.47 (37.29,52.92)					
	Risk obs (95% CI)	2.03 (1.30,3.03)	3.62 (2.74,4.69)	5.55 (4.50,6.75)	7.06 (5.84,8.42)	8.49 (7.07,10.06)	9.97 (8.18,11.96)	11.84 (8.22,16.17)					
	Risk exp	0.02	0.08	0.19	0.37	0.65	0.98	1.30					
30	SIR (95% CI)		29.83 (23.11,38.50)	16.86 (14.73,19.31)	13.19 (11.88,14.64)	10.38 (9.45,11.40)	8.89 (8.12,9.73)	8.50 (7.78,9.28)	8.45 (7.73,9.23)				
	AER (95% CI)		40.94 (31.40,53.26)	42.51 (36.80,49.06)	46.83 (41.80,52.42)	47.05 (42.38,52.17)	45.65 (41.22,50.50)	45.39 (41.03,50.16)	45.33 (40.97,50.09)				
	Risk obs (95% CI)		2.10 (1.61,2.68)	4.01 (3.44,4.64)	6.11 (5.44,6.82)	7.86 (7.10,8.66)	9.07 (8.22,9.96)	10.34 (9.31,11.44)	11.20 (9.33,13.25)				
	Risk exp		0.07	0.21	0.42	0.74	1.11	1.46	1.83				
35	SIR (95% CI)			9.48 (7.84,11.46)	6.91 (6.27,7.61)	5.85 (5.43,6.30)	5.02 (4.70,5.37)	4.78 (4.49,5.10)	4.65 (4.36,4.95)	4.64 (4.36,4.94)			
	AER (95% CI)			27.14 (21.90,33.47)	31.07 (27.73,34.75)	34.05 (31.13,37.21)	32.99 (30.32,35.85)	32.90 (30.32,35.64)	32.47 (29.93,35.17)	32.45 (29.92,35.15)			
	Risk obs (95% CI)			1.51 (1.24,1.81)	3.16 (2.84,3.51)	4.98 (4.60,5.38)	6.31 (5.88,6.77)	7.63 (7.12,8.17)	8.49 (7.86,9.15)	9.51 (8.05,11.10)			
	Risk exp			0.16	0.42	0.81	1.25	1.66	2.09	2.54			
40	SIR (95% CI)				4.43 (3.85,5.10)	3.36 (3.12,3.62)	3.10 (2.92,3.28)	3.15 (2.99,3.32)	3.13 (2.98,3.29)	3.10 (2.95,3.25)	3.10 (2.95,3.25)		
	AER (95% CI)				21.07 (17.49,25.19)	21.37 (19.18,23.72)	22.34 (20.47,24.33)	24.10 (22.33,25.97)	24.77 (23.03,26.59)	24.78 (23.06,26.59)	24.83 (23.11,26.63)		
	Risk obs (95% CI)				1.35 (1.17,1.55)	2.75 (2.53,2.98)	4.21 (3.96,4.48)	5.83 (5.52,6.15)	7.36 (6.98,7.76)	8.60 (8.09,9.13)	10.15 (8.88,11.53)		
	Risk exp				0.31	0.76	1.30	1.79	2.31	2.85	3.26		
45	SIR (95% CI)					2.17 (1.93,2.45)	2.07 (1.94,2.20)	2.22 (2.12,2.34)	2.37 (2.27,2.47)	2.40 (2.30,2.49)	2.41 (2.31,2.50)	2.40 (2.31,2.50)	
	AER (95% CI)					12.09 (9.56,14.95)	13.63 (12.03,15.32)	16.24 (14.84,17.71)	18.96 (17.60,20.38)	20.01 (18.67,21.41)	20.26 (18.92,21.64)	20.25 (18.92,21.64)	
	Risk obs (95% CI)					1.11 (0.99,1.25)	2.37 (2.22,2.54)	3.79 (3.60,3.99)	5.57 (5.33,5.82)	7.23 (6.92,7.54)	8.51 (8.11,8.93)	9.40 (8.55,10.29)	
	Risk exp					0.51	1.14	1.70	2.30	2.94	3.42	3.88	
50	SIR (95% CI)						1.42 (1.28,1.57)	1.71 (1.62,1.81)	1.92 (1.84,2.00)	2.07 (1.99,2.14)	2.09 (2.02,2.16)	2.10 (2.02,2.17)	2.10 (2.03,2.17)
	AER (95% CI)						5.86 (3.91,8.01)	10.18 (8.86,11.57)	13.95 (12.74,15.20)	17.06 (15.88,18.28)	17.53 (16.38,18.72)	17.74 (16.60,18.93)	17.75 (16.61,18.94)
	Risk obs (95% CI)						0.99 (0.89,1.09)	2.20 (2.07,2.33)	3.78 (3.62,3.95)	5.68 (5.48,5.90)	6.97 (6.71,7.23)	8.25 (7.89,8.62)	9.34 (8.36,10.37)
	Risk exp						0.70	1.35	2.04	2.76	3.32	3.84	4.32
55	SIR (95% CI)							1.44 (1.28,1.60)	1.56 (1.47,1.65)	1.83 (1.75,1.91)	1.97 (1.90,2.04)	2.03 (1.96,2.10)	2.05 (1.97,2.12)
	AER (95% CI)							6.28 (4.11,8.71)	9.19 (7.76,10.71)	14.53 (13.20,15.92)	17.00 (15.73,18.32)	18.27 (17.01,19.57)	18.59 (17.33,19.89)
	Risk obs (95% CI)							1.03 (0.92,1.15)	2.26 (2.12,2.40)	4.01 (3.84,4.19)	5.59 (5.38,5.81)	7.19 (6.91,7.47)	8.50 (8.11,8.90)
	Risk exp							0.72	1.48	2.25	2.84	3.40	3.89
60	SIR (95% CI)								1.22 (1.10,1.36)	1.34 (1.27,1.42)	1.61 (1.54,1.68)	1.75 (1.68,1.82)	1.79 (1.73,1.86)
	AER (95% CI)								3.79 (1.70,6.11)	6.31 (4.95,7.76)	11.11 (9.87,12.41)	13.86 (12.64,15.12)	14.73 (13.52,15.98)
	Risk obs (95% CI)								1.04 (0.94,1.16)	2.22 (2.09,2.36)	3.70 (3.54,3.87)	5.31 (5.10,5.52)	6.62 (6.35,6.89)
	Risk exp								0.86	1.70	2.36	2.99	3.53
65	SIR (95% CI)									1.05 (0.94,1.18)	1.27 (1.19,1.35)	1.42 (1.35,1.50)	1.49 (1.42,1.56)
	AER (95% CI)									1.04 (-1.12,3.45)	4.75 (3.37,6.23)	7.81 (6.53,9.15)	9.18 (7.93,10.48)
	Risk obs (95% CI)									1.00 (0.89,1.12)	2.03 (1.90,2.17)	3.29 (3.12,3.46)	4.45 (4.24,4.67)
	Risk exp									0.95	1.69	2.39	2.99
70	SIR (95% CI)										1.36 (1.20,1.54)	1.38 (1.29,1.47)	1.45 (1.37,1.53)
	AER (95% CI)										6.15 (3.46,9.18)	6.93 (5.26,8.72)	8.63 (7.13,10.21)
	Risk obs (95% CI)										1.15 (1.01,1.30)	2.20 (2.04,2.37)	3.23 (3.05,3.43)
	Risk exp										0.84	1.61	2.25

Table S5: Standardised incidence ratios (SIR), absolute excess rates (per 10,000) (AER), and 10- and 20-year cumulative risks (%) by type of second cancer. Cohort rates are compared to all England rates for the same calendar year, attained age and quintile of deprivation. To calculate the expected risk for contralateral breast cancer, the national incidence rates for breast cancer have been halved.

					10-year cumulative risk % (95% CI)			20-year cumulative risk % (95% CI)		
**Second cancer group**	**Observed**	**Expected**	**SIR (95% CI)**	**AER (95% CI)**	**Observed**	**Expected**	**Difference**	**Observed**	**Expected**	**Difference**
**Gynaecological**										
Cervix	488	545	0.90 (0.82,0.98)	-0.11 (-0.18,-0.02)	0.08 (0.07,0.08)	0.09	-0.01 (-0.02,-0.01)	0.14 (0.12,0.15)	0.15	-0.01 (-0.03,0.00)
Uterine	5793	3398	1.70 (1.66,1.75)	4.50 (4.21,4.78)	0.85 (0.82,0.88)	0.50	0.34 (0.32,0.37)	1.73 (1.68,1.78)	1.00	0.74 (0.69,0.79)
Ovary or fallopian tubes	3729	3067	1.22 (1.18,1.26)	1.24 (1.04,1.50)	0.56 (0.53,0.58)	0.47	0.09 (0.07,0.11)	1.09 (1.05,1.13)	0.89	0.20 (0.16,0.24)
Urinary system	2623	2412	1.09 (1.05,1.13)	0.40 (0.23,0.59)	0.37 (0.36,0.39)	0.34	0.03 (0.02,0.05)	0.77 (0.74,0.81)	0.72	0.05 (0.02,0.08)
**Hematopietic/lymphoid**										
Lymphoma	2180	2278	0.96 (0.92,1.00)	-0.18 (-0.34,0.00)	0.30 (0.28,0.32)	0.33	-0.03 (-0.04,-0.01)	0.66 (0.63,0.70)	0.68	-0.01 (-0.04,0.02)
Acute lymphoblastic leukaemia	63	35	1.80 (1.41,2.30)	0.05 (0.03,0.09)	0.01 (0.01,0.02)	0.01	0.01 (0.00,0.01)	0.02 (0.01,0.02)	0.01	0.01 (0.00,0.01)
Acute myeloid leukaemia	651	385	1.69 (1.57,1.83)	0.50 (0.41,0.60)	0.11 (0.10,0.12)	0.06	0.05 (0.04,0.06)	0.18 (0.16,0.20)	0.11	0.07 (0.05,0.08)
Other leukaemia	781	776	1.01 (0.94,1.08)	0.01 (-0.09,0.12)	0.11 (0.10,0.12)	0.11	-0.00 (-0.01,0.01)	0.25 (0.23,0.27)	0.23	0.01 (-0.01,0.03)
Myeloma	823	862	0.95 (0.89,1.02)	-0.07 (-0.18,0.03)	0.11 (0.10,0.13)	0.12	-0.01 (-0.02,0.00)	0.25 (0.23,0.27)	0.26	-0.01 (-0.03,0.01)
**Bone and soft tissue**										
Bones and joints	79	51	1.54 (1.24,1.92)	0.05 (0.02,0.09)	0.01 (0.01,0.02)	0.01	0.00 (0.00,0.01)	0.02 (0.02,0.03)	0.01	0.01 (0.00,0.01)
Soft tissue	468	233	2.01 (1.83,2.20)	0.44 (0.36,0.53)	0.08 (0.07,0.08)	0.03	0.04 (0.03,0.05)	0.13 (0.12,0.15)	0.07	0.06 (0.05,0.08)
**Skin**										
Melanoma of skin	2319	2137	1.08 (1.04,1.13)	0.34 (0.16,0.52)	0.34 (0.33,0.36)	0.31	0.03 (0.01,0.05)	0.69 (0.66,0.72)	0.63	0.06 (0.03,0.09)
**Head and neck/endocrine**										
Salivary gland	177	91	1.95 (1.68,2.26)	0.16 (0.12,0.22)	0.03 (0.02,0.03)	0.01	0.01 (0.01,0.02)	0.05 (0.05,0.06)	0.03	0.03 (0.02,0.04)
Other head and neck	938	835	1.12 (1.05,1.20)	0.19 (0.08,0.31)	0.13 (0.12,0.15)	0.12	0.01 (0.00,0.02)	0.28 (0.26,0.30)	0.25	0.04 (0.02,0.06)
Thyroid	545	482	1.13 (1.04,1.23)	0.12 (0.04,0.21)	0.08 (0.07,0.09)	0.07	0.01 (-0.00,0.02)	0.15 (0.14,0.17)	0.14	0.02 (0.00,0.03)
Other endocrine gland	90	86	1.05 (0.85,1.29)	0.01 (-0.02,0.05)	0.01 (0.01,0.02)	0.01	0.00 (-0.00,0.00)	0.03 (0.02,0.03)	0.03	0.00 (-0.01,0.01)
**Upper gastrointestinal**										
Oesophagus	1015	939	1.08 (1.02,1.15)	0.14 (0.04,0.26)	0.14 (0.13,0.15)	0.13	0.00 (-0.01,0.01)	0.31 (0.29,0.33)	0.28	0.03 (0.01,0.05)
Stomach	992	780	1.27 (1.20,1.35)	0.40 (0.29,0.51)	0.15 (0.14,0.16)	0.12	0.03 (0.02,0.04)	0.29 (0.27,0.31)	0.23	0.06 (0.04,0.08)
Pancreas	1993	1643	1.21 (1.16,1.27)	0.66 (0.49,0.83)	0.26 (0.24,0.28)	0.23	0.03 (0.02,0.05)	0.62 (0.59,0.65)	0.50	0.12 (0.09,0.15)
**Lower or other gastrointestinal**										
Colorectal	6835	6302	1.08 (1.06,1.11)	1.00 (0.71,1.30)	0.96 (0.94,0.99)	0.90	0.07 (0.04,0.10)	2.05 (1.99,2.10)	1.88	0.17 (0.11,0.22)
Other GI	1276	1179	1.08 (1.02,1.14)	0.18 (0.04,0.31)	0.17 (0.16,0.18)	0.16	0.00 (-0.01,0.02)	0.39 (0.37,0.42)	0.36	0.03 (0.01,0.06)
**Liver**										
Liver	665	602	1.10 (1.02,1.19)	0.12 (0.02,0.21)	0.09 (0.08,0.10)	0.08	0.01 (-0.00,0.02)	0.21 (0.19,0.23)	0.19	0.03 (0.01,0.05)
**Respiratory**										
Lung	8741	7533	1.16 (1.14,1.18)	2.27 (1.98,2.55)	1.15 (1.12,1.19)	1.05	0.10 (0.07,0.13)	2.68 (2.61,2.74)	2.26	0.41 (0.35,0.48)
Other respiratory and intrathoracic	445	406	1.10 (1.00,1.20)	0.07 (0.00,0.15)	0.07 (0.06,0.07)	0.06	0.01 (-0.00,0.01)	0.13 (0.12,0.15)	0.12	0.01 (-0.00,0.03)
**Central nervous system**										
CNS tumours	1549	1510	1.03 (0.98,1.08)	0.07 (-0.06,0.23)	0.23 (0.21,0.24)	0.22	0.00 (-0.01,0.02)	0.46 (0.43,0.48)	0.44	0.01 (-0.01,0.04)
**All cancers (excluding breast)**										
Other specified sites	202	170	1.19 (1.04,1.37)	0.06 (0.01,0.12)	0.03 (0.02,0.03)	0.02	0.00 (-0.00,0.01)	0.06 (0.05,0.07)	0.05	0.01 (-0.00,0.02)
All specified sites	45467	38740	1.17 (1.16,1.18)	12.63 (11.64,13.09)	6.40 (6.32,6.47)	5.56	0.83 (0.76,0.91)	13.59 (13.45,13.73)	11.46	2.13 (1.99,2.27)
Mutliple or unspecified sites	1153	1544	0.75 (0.71,0.79)	-0.73 (-0.84,-0.61)	0.15 (0.14,0.17)	0.24	-0.08 (-0.09,-0.07)	0.36 (0.34,0.38)	0.45	-0.09 (-0.11,-0.06)
**Breast**										
Contralateral	18127	8650	2.10 (2.07,2.13)	17.79 (17.38,18.35)	2.47 (2.42,2.51)	1.34	1.13 (1.08,1.18)	5.55 (5.46,5.64)	2.48	3.07 (2.98,3.16)

Table S6: Standardised incidence ratios (SIR), absolute excess rates (per 10,000) (AER), and 10- and 20-year cumulative risks (%) by type of second cancer for women aged 20-59 years at diagnosis of index breast cancer. Cohort rates are compared to all England rates for the same calendar year, attained age and quintile of deprivation. To calculate the expected risk for contralateral breast cancer, the national incidence rates for breast cancer have been halved. See figures S6 and S7 for SIRs estimated for narrower categories of age and at various years of follow-up.

					10-year cumulative risk % (95% CI)			20-year cumulative risk % (95% CI)		
**Second cancer group**	**Observed**	**Expected**	**SIR (95% CI)**	**AER (95% CI)**	**Observed**	**Expected**	**Difference**	**Observed**	**Expected**	**Difference**
**Gynaecological**										
Cervix	286	348	0.82 (0.73,0.92)	-0.19 (-0.28,-0.08)	0.08 (0.07,0.09)	0.10	-0.02 (-0.03,-0.01)	0.13 (0.11,0.15)	0.16	-0.03 (-0.05,-0.01)
Uterine	2931	1755	1.67 (1.61,1.73)	3.50 (3.19,3.81)	0.66 (0.63,0.69)	0.37	0.29 (0.25,0.32)	1.46 (1.40,1.52)	0.88	0.58 (0.53,0.64)
Ovary or fallopian tubes	1973	1517	1.30 (1.24,1.36)	1.36 (1.08,1.63)	0.45 (0.42,0.48)	0.34	0.11 (0.08,0.14)	0.97 (0.92,1.02)	0.74	0.22 (0.18,0.27)
Urinary system	1125	962	1.17 (1.10,1.24)	0.49 (0.29,0.69)	0.22 (0.20,0.24)	0.18	0.04 (0.03,0.06)	0.56 (0.53,0.60)	0.49	0.07 (0.04,0.11)
**Hematopietic/lymphoid**										
Lymphoma	984	1016	0.97 (0.91,1.03)	-0.09 (-0.27,0.09)	0.18 (0.16,0.20)	0.20	-0.02 (-0.04,-0.01)	0.50 (0.47,0.54)	0.51	-0.01 (-0.04,0.03)
Acute lymphoblastic leukaemia	42	19	2.18 (1.61,2.95)	0.07 (0.03,0.11)	0.01 (0.01,0.02)	0.00	0.01 (0.00,0.01)	0.02 (0.01,0.02)	0.01	0.01 (0.00,0.01)
Acute myeloid leukaemia	311	158	1.96 (1.76,2.19)	0.45 (0.36,0.56)	0.09 (0.07,0.10)	0.03	0.05 (0.04,0.07)	0.14 (0.12,0.16)	0.08	0.06 (0.04,0.08)
Other leukaemia	328	313	1.05 (0.94,1.17)	0.04 (-0.06,0.16)	0.06 (0.05,0.07)	0.06	0.00 (-0.01,0.01)	0.18 (0.16,0.20)	0.16	0.02 (-0.00,0.04)
Myeloma	323	345	0.94 (0.84,1.04)	-0.07 (-0.16,0.04)	0.06 (0.05,0.07)	0.06	-0.00 (-0.01,0.01)	0.17 (0.15,0.19)	0.18	-0.01 (-0.03,0.02)
**Bone and soft tissue**										
Bones and joints	54	28	1.92 (1.47,2.50)	0.08 (0.04,0.13)	0.01 (0.01,0.02)	0.01	0.01 (0.00,0.01)	0.02 (0.02,0.03)	0.01	0.01 (0.00,0.02)
Soft tissue	254	115	2.20 (1.95,2.49)	0.41 (0.33,0.51)	0.07 (0.06,0.08)	0.03	0.04 (0.03,0.05)	0.12 (0.10,0.13)	0.06	0.06 (0.04,0.08)
**Skin**										
Melanoma of skin	1286	1172	1.10 (1.04,1.16)	0.34 (0.14,0.56)	0.30 (0.28,0.32)	0.26	0.04 (0.02,0.06)	0.64 (0.60,0.68)	0.57	0.06 (0.02,0.10)
**Head and neck/endocrine**										
Salivary gland	124	48	2.59 (2.17,3.09)	0.23 (0.17,0.30)	0.03 (0.02,0.04)	0.01	0.02 (0.01,0.02)	0.06 (0.05,0.08)	0.02	0.04 (0.03,0.05)
Other head and neck	531	452	1.18 (1.08,1.28)	0.24 (0.11,0.38)	0.12 (0.10,0.13)	0.10	0.02 (0.01,0.03)	0.27 (0.24,0.29)	0.22	0.04 (0.02,0.07)
Thyroid	352	309	1.14 (1.03,1.27)	0.13 (0.03,0.25)	0.08 (0.07,0.09)	0.08	0.01 (-0.01,0.02)	0.17 (0.15,0.19)	0.15	0.02 (0.00,0.04)
Other endocrine gland	53	46	1.15 (0.88,1.50)	0.02 (-0.02,0.07)	0.01 (0.01,0.02)	0.01	0.00 (-0.00,0.01)	0.03 (0.02,0.03)	0.02	0.00 (-0.00,0.01)
**Upper gastrointestinal**										
Oesophagus	423	351	1.21 (1.10,1.33)	0.21 (0.10,0.34)	0.07 (0.06,0.08)	0.06	0.01 (-0.00,0.02)	0.22 (0.20,0.25)	0.18	0.04 (0.02,0.07)
Stomach	387	270	1.43 (1.30,1.58)	0.35 (0.24,0.47)	0.08 (0.07,0.09)	0.05	0.03 (0.02,0.04)	0.20 (0.17,0.22)	0.14	0.06 (0.04,0.08)
Pancreas	816	617	1.32 (1.23,1.42)	0.59 (0.42,0.77)	0.14 (0.12,0.15)	0.10	0.03 (0.02,0.05)	0.43 (0.40,0.47)	0.32	0.12 (0.08,0.15)
**Lower or other gastrointestinal**										
Colorectal	2779	2485	1.12 (1.08,1.16)	0.87 (0.59,1.18)	0.51 (0.48,0.54)	0.47	0.05 (0.02,0.08)	1.42 (1.36,1.48)	1.25	0.16 (0.10,0.22)
Other GI	616	530	1.16 (1.07,1.26)	0.26 (0.11,0.41)	0.11 (0.10,0.12)	0.10	0.01 (-0.00,0.02)	0.31 (0.28,0.34)	0.27	0.04 (0.01,0.07)
**Liver**										
Liver	266	232	1.15 (1.02,1.29)	0.10 (0.01,0.20)	0.04 (0.03,0.05)	0.04	0.00 (-0.00,0.01)	0.14 (0.12,0.16)	0.12	0.02 (0.00,0.04)
**Respiratory**										
Lung	4035	2986	1.35 (1.31,1.39)	3.12 (2.76,3.47)	0.66 (0.63,0.69)	0.51	0.15 (0.12,0.18)	2.08 (2.01,2.16)	1.54	0.54 (0.47,0.62)
Other respiratory and intrathoracic	217	176	1.24 (1.08,1.41)	0.12 (0.04,0.21)	0.05 (0.04,0.05)	0.03	0.01 (0.00,0.02)	0.11 (0.09,0.13)	0.09	0.02 (0.01,0.04)
**Central nervous system**										
CNS tumours	841	783	1.07 (1.00,1.15)	0.17 (0.00,0.35)	0.19 (0.17,0.21)	0.17	0.02 (-0.00,0.03)	0.41 (0.38,0.44)	0.39	0.02 (-0.01,0.05)
**All cancers (excluding breast)**										
Other specified sites	106	82	1.29 (1.07,1.56)	0.07 (0.02,0.14)	0.02 (0.01,0.03)	0.02	0.00 (-0.00,0.01)	0.05 (0.04,0.06)	0.04	0.01 (-0.00,0.02)
All specified sites	21446	17116	1.25 (1.24,1.27)	12.89 (12.23,13.76)	4.28 (4.20,4.36)	3.38	0.90 (0.82,0.98)	10.76 (10.60,10.92)	8.57	2.19 (2.03,2.35)
Mutliple or unspecified sites	513	512	1.00 (0.92,1.09)	0.00 (-0.12,0.14)	0.11 (0.10,0.12)	0.11	0.00 (-0.01,0.01)	0.26 (0.23,0.28)	0.25	0.01 (-0.02,0.03)
**Breast**										
Contralateral	12171	4948	2.46 (2.42,2.50)	21.50 (20.91,22.09)	2.64 (2.57,2.70)	1.19	1.45 (1.39,1.51)	6.09 (5.97,6.21)	2.39	3.70 (3.58,3.82)

Table S7: Standardised incidence ratios (SIR), absolute excess rates (per 10,000) (AER), and 10- and 20-year cumulative risks (%) by type of second cancer for women aged 60-75 years at diagnosis of index breast cancer. Cohort rates are compared to all England rates for the same calendar year, attained age and quintile of deprivation. To calculate the expected risk for contralateral breast cancer, the national incidence rates for breast cancer have been halved. See figures S6 and S7 for SIRs estimated for narrower categories of age and at various years of follow-up.

					10-year cumulative risk % (95% CI)			20-year cumulative risk % (95% CI)		
**Second cancer group**	**Observed**	**Expected**	**SIR (95% CI)**	**AER (95% CI)**	**Observed**	**Expected**	**Difference**	**Observed**	**Expected**	**Difference**
**Gynaecological**										
Cervix	202	197	1.03 (0.89,1.18)	0.03 (-0.11,0.18)	0.07 (0.06,0.09)	0.08	-0.01 (-0.02,0.00)	0.16 (0.13,0.19)	0.14	0.02 (-0.01,0.05)
Uterine	2862	1643	1.74 (1.68,1.81)	6.20 (5.68,6.77)	1.11 (1.06,1.16)	0.69	0.42 (0.37,0.47)	2.19 (2.10,2.29)	1.17	1.02 (0.93,1.12)
Ovary or fallopian tubes	1756	1550	1.13 (1.08,1.19)	1.05 (0.63,1.50)	0.70 (0.67,0.74)	0.64	0.07 (0.03,0.10)	1.28 (1.21,1.36)	1.12	0.16 (0.09,0.23)
Urinary system	1498	1450	1.03 (0.98,1.09)	0.24 (-0.15,0.66)	0.58 (0.55,0.62)	0.57	0.02 (-0.02,0.05)	1.12 (1.05,1.19)	1.12	0.00 (-0.07,0.07)
**Hematopietic/lymphoid**										
Lymphoma	1196	1262	0.95 (0.90,1.00)	-0.34 (-0.64,0.00)	0.47 (0.44,0.50)	0.50	-0.04 (-0.07,-0.00)	0.94 (0.87,1.00)	0.95	-0.01 (-0.08,0.06)
Acute lymphoblastic leukaemia	21	16	1.33 (0.87,2.04)	0.03 (-0.01,0.08)	0.01 (0.01,0.01)	0.01	0.00 (-0.00,0.01)	0.01 (0.01,0.03)	0.01	0.00 (-0.00,0.01)
Acute myeloid leukaemia	340	226	1.50 (1.35,1.67)	0.58 (0.40,0.77)	0.13 (0.12,0.15)	0.09	0.05 (0.03,0.06)	0.25 (0.22,0.29)	0.17	0.08 (0.05,0.11)
Other leukaemia	453	463	0.98 (0.89,1.07)	-0.05 (-0.26,0.16)	0.17 (0.16,0.19)	0.18	-0.01 (-0.03,0.01)	0.35 (0.31,0.39)	0.35	-0.00 (-0.04,0.04)
Myeloma	500	517	0.97 (0.89,1.06)	-0.09 (-0.29,0.16)	0.19 (0.17,0.21)	0.20	-0.01 (-0.03,0.01)	0.39 (0.35,0.43)	0.40	-0.01 (-0.05,0.03)
**Bone and soft tissue**										
Bones and joints	25	23	1.08 (0.73,1.60)	0.01 (-0.03,0.07)	0.01 (0.01,0.02)	0.01	0.00 (-0.00,0.01)	0.01 (0.01,0.02)	0.02	-0.00 (-0.01,0.00)
Soft tissue	214	118	1.82 (1.59,2.08)	0.49 (0.35,0.65)	0.09 (0.08,0.10)	0.05	0.04 (0.03,0.05)	0.17 (0.14,0.19)	0.09	0.08 (0.05,0.11)
**Skin**										
Melanoma of skin	1033	965	1.07 (1.01,1.14)	0.34 (0.05,0.69)	0.41 (0.38,0.44)	0.39	0.02 (-0.01,0.05)	0.78 (0.72,0.84)	0.72	0.05 (-0.00,0.11)
**Head and neck/endocrine**										
Salivary gland	53	43	1.23 (0.94,1.61)	0.05 (-0.01,0.13)	0.02 (0.02,0.03)	0.02	0.00 (-0.00,0.01)	0.04 (0.03,0.05)	0.03	0.01 (-0.01,0.02)
Other head and neck	407	383	1.06 (0.96,1.17)	0.12 (-0.08,0.33)	0.16 (0.14,0.18)	0.16	0.00 (-0.02,0.02)	0.32 (0.28,0.36)	0.28	0.04 (0.00,0.08)
Thyroid	193	173	1.12 (0.97,1.28)	0.10 (-0.03,0.25)	0.08 (0.07,0.09)	0.07	0.01 (-0.00,0.02)	0.13 (0.11,0.15)	0.12	0.00 (-0.02,0.02)
Other endocrine gland	37	40	0.93 (0.67,1.28)	-0.01 (-0.07,0.06)	0.01 (0.01,0.02)	0.02	-0.00 (-0.01,0.00)	0.03 (0.02,0.05)	0.03	0.00 (-0.01,0.02)
**Upper gastrointestinal**										
Oesophagus	592	588	1.01 (0.93,1.09)	0.02 (-0.21,0.27)	0.23 (0.21,0.25)	0.23	-0.00 (-0.03,0.02)	0.47 (0.43,0.52)	0.45	0.02 (-0.02,0.07)
Stomach	605	510	1.19 (1.10,1.28)	0.48 (0.26,0.73)	0.24 (0.22,0.27)	0.20	0.04 (0.02,0.06)	0.45 (0.41,0.50)	0.38	0.07 (0.03,0.11)
Pancreas	1177	1026	1.15 (1.08,1.21)	0.77 (0.42,1.10)	0.43 (0.40,0.46)	0.40	0.03 (0.00,0.06)	0.93 (0.87,1.00)	0.80	0.13 (0.07,0.20)
**Lower or other gastrointestinal**										
Colorectal	4056	3817	1.06 (1.03,1.10)	1.22 (0.58,1.94)	1.59 (1.53,1.65)	1.50	0.09 (0.03,0.15)	3.09 (2.97,3.20)	2.92	0.17 (0.05,0.29)
Other GI	660	649	1.02 (0.94,1.10)	0.05 (-0.20,0.33)	0.25 (0.22,0.27)	0.25	-0.01 (-0.03,0.02)	0.52 (0.48,0.58)	0.50	0.03 (-0.02,0.08)
**Liver**										
Liver	399	370	1.08 (0.98,1.19)	0.15 (-0.04,0.36)	0.15 (0.13,0.17)	0.14	0.01 (-0.01,0.03)	0.34 (0.30,0.38)	0.30	0.04 (-0.00,0.08)
**Respiratory**										
Lung	4706	4547	1.03 (1.01,1.06)	0.81 (0.23,1.39)	1.84 (1.78,1.90)	1.80	0.03 (-0.03,0.10)	3.61 (3.48,3.74)	3.42	0.19 (0.06,0.31)
Other respiratory and intrathoracic	228	231	0.99 (0.87,1.12)	-0.01 (-0.15,0.14)	0.09 (0.08,0.11)	0.09	-0.00 (-0.01,0.01)	0.17 (0.15,0.21)	0.17	0.00 (-0.03,0.03)
**Central nervous system**										
CNS tumours	708	727	0.97 (0.90,1.05)	-0.10 (-0.37,0.18)	0.28 (0.25,0.30)	0.29	-0.02 (-0.04,0.01)	0.55 (0.50,0.60)	0.54	0.01 (-0.04,0.06)
**All cancers (excluding breast)**										
Other specified sites	96	88	1.09 (0.90,1.34)	0.04 (-0.04,0.15)	0.04 (0.03,0.05)	0.04	0.01 (-0.00,0.01)	0.07 (0.05,0.08)	0.07	0.00 (-0.01,0.02)
All specified sites	24021	21624	1.11 (1.10,1.12)	12.18 (10.99,13.19)	9.31 (9.17,9.45)	8.57	0.74 (0.60,0.88)	18.25 (17.97,18.53)	16.16	2.09 (1.81,2.37)
Mutliple or unspecified sites	640	1032	0.62 (0.57,0.67)	-1.99 (-2.26,-1.73)	0.22 (0.19,0.24)	0.42	-0.20 (-0.22,-0.18)	0.54 (0.49,0.60)	0.77	-0.23 (-0.28,-0.17)
**Breast**										
Contralateral	5956	3703	1.61 (1.57,1.65)	11.46 (10.73,12.23)	2.24 (2.17,2.31)	1.54	0.69 (0.62,0.76)	4.73 (4.59,4.88)	2.64	2.09 (1.95,2.24)

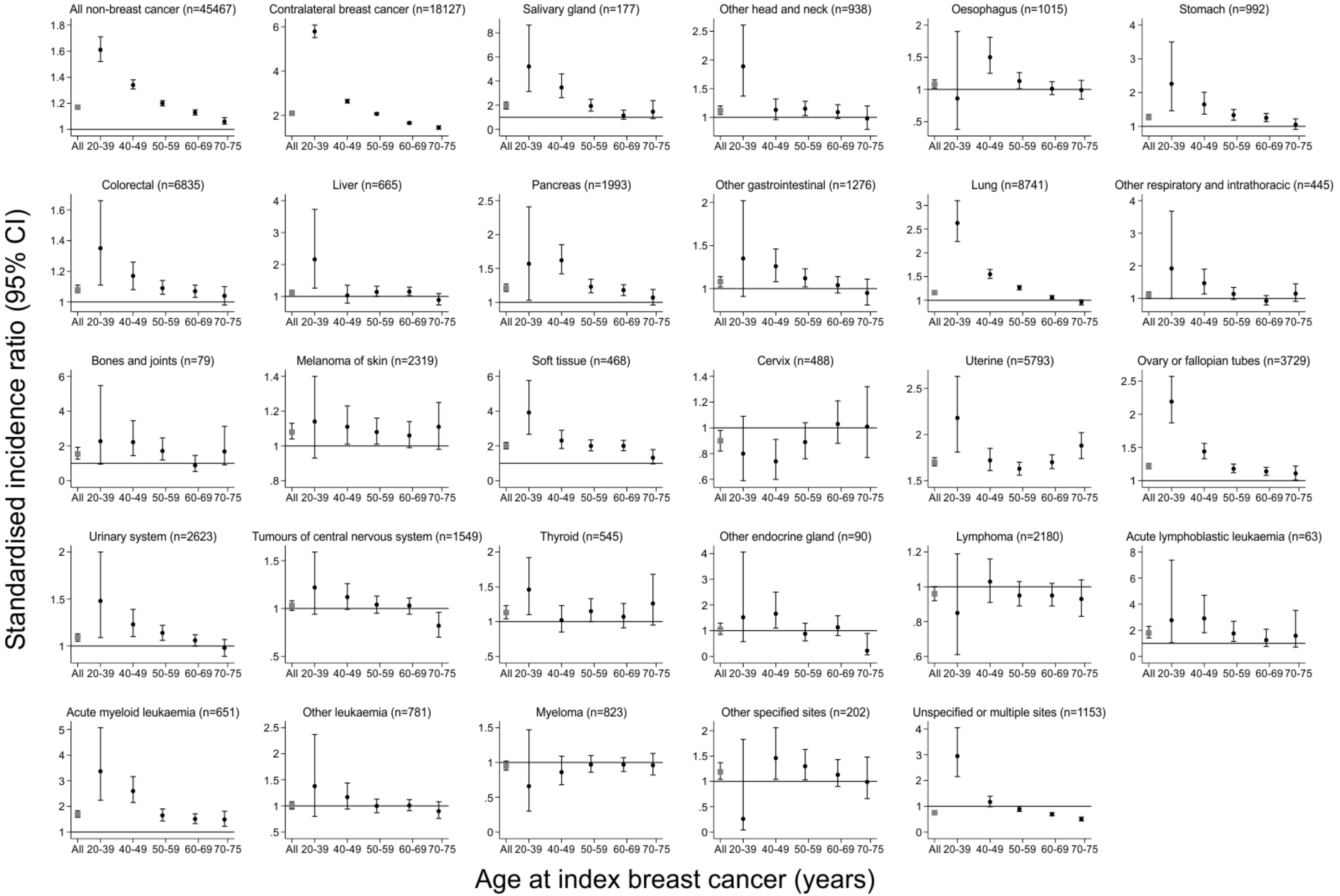

Figure S6: Standardised incidence ratios for different second cancer groups by age at index breast cancer diagnosis. Cohort rates are compared to all England rates for the same calendar year, attained age and quintile of deprivation (in order to compare incidence of contralateral breast cancer, national rates are halved).

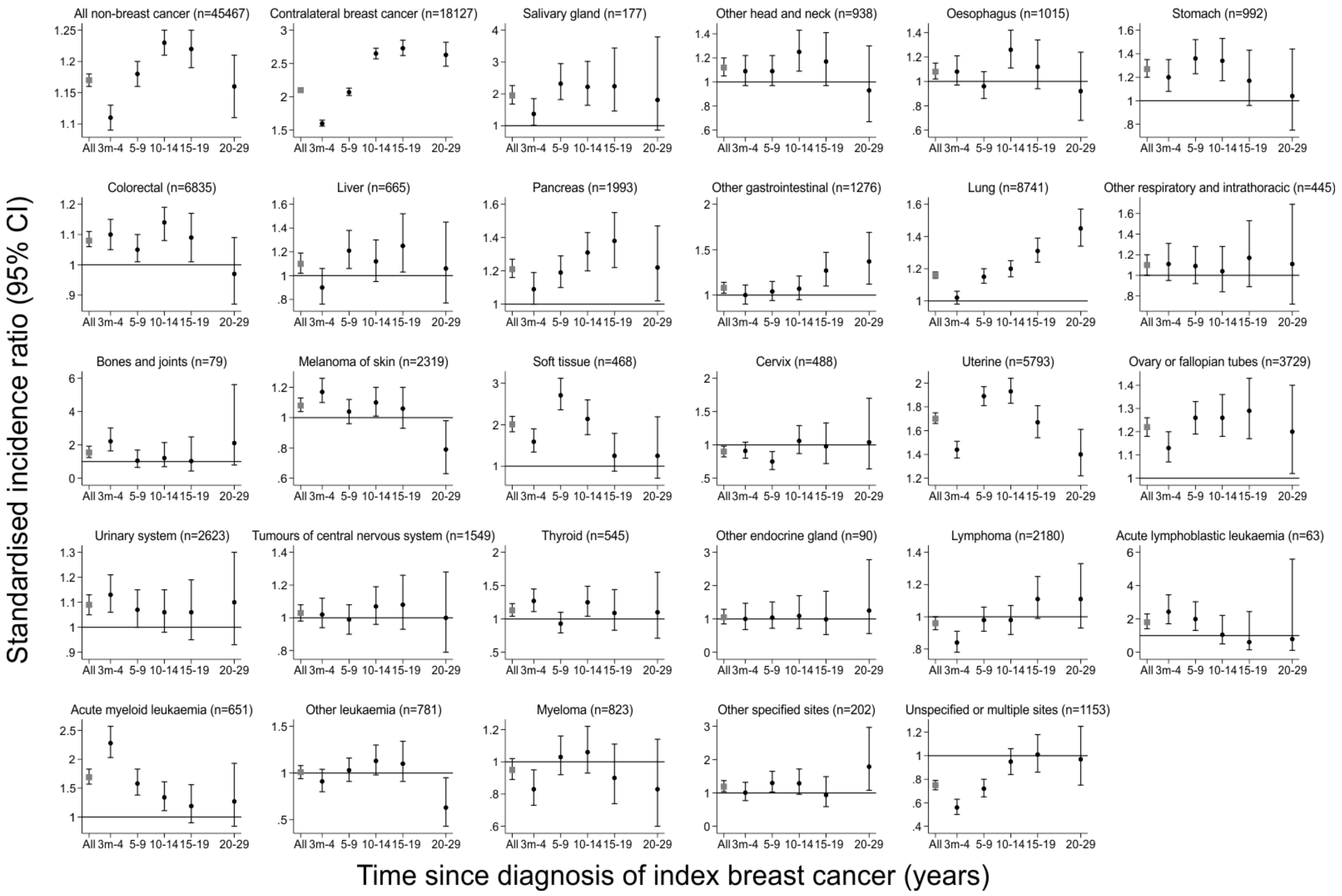

Figure S7: Standardised incidence ratios for different second cancer groups by time since index breast cancer diagnosis. Cohort rates are compared to all England rates for the same calendar year, attained age and quintile of deprivation (in order to compare incidence of contralateral breast cancer, national rates are halved).

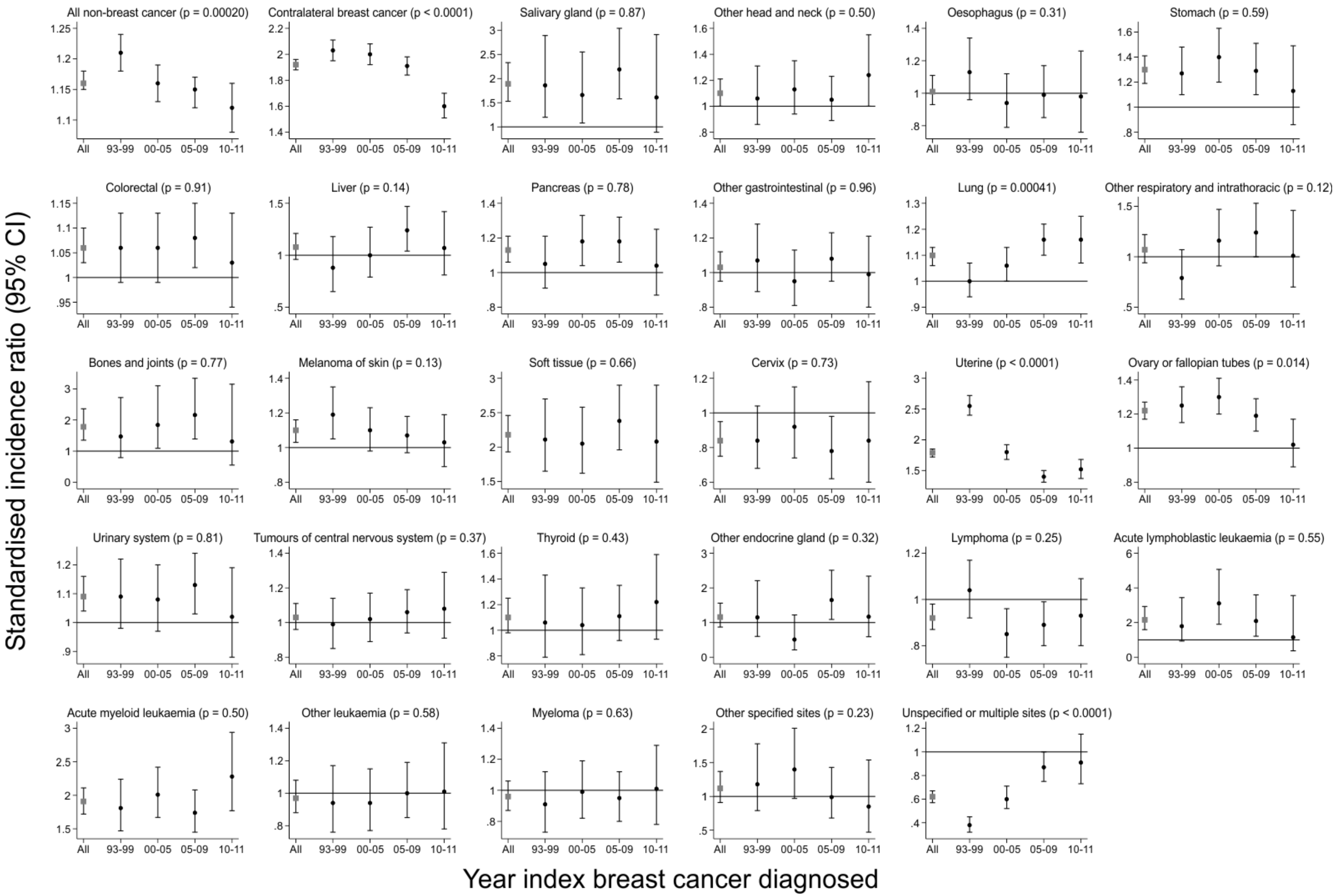

Figure S8: Standardised incidence ratios (SIR) for different second cancer groups by year index breast cancer diagnosed. To reduce confounding by length of follow-up (see Figure S7) analysis was restricted to the first 10-years and breast cancer diagnosis period 1993-2011. Cohort rates are compared to all England rates for the same calendar year, attained age and quintile of deprivation (in order to compare incidence of contralateral breast cancer, national rates are halved). P-value is a test for trend. Uterine excludes cervix.

a) Standardised incidence ratios (SIR)

Type of second cancer	Observed	Expected	SIR (95% CI)
Soft tissue	569	248.2	2.29 (2.11, 2.49)
Salivary gland	198	96.7	2.05 (1.78, 2.35)
Acute lymphoblastic leukaemia	68	37.1	1.83 (1.45, 2.33)
Acute myeloid leukaemia	724	411.3	1.76 (1.64, 1.89)
Uterine	6343	3618.9	1.75 (1.71, 1.80)
Bones and joints	92	54.3	1.69 (1.38, 2.08)
Stomach	1118	833.8	1.34 (1.26, 1.42)
Ovary or fallopian tubes	4207	3262.4	1.29 (1.25, 1.33)
Pancreas	2238	1767	1.27 (1.22, 1.32)
Other specified sites	229	181.4	1.26 (1.11, 1.44)
Other head and neck	1112	890.4	1.25 (1.18, 1.32)
Lung	9884	8090.5	1.22 (1.20, 1.25)
Thyroid	622	510.1	1.22 (1.13, 1.32)
Liver	776	649.7	1.19 (1.11, 1.28)
Other gastrointestinal	1492	1265.9	1.18 (1.12, 1.24)
Urinary system	3022	2587.7	1.17 (1.13, 1.21)
Colorectal	7865	6753.2	1.16 (1.14, 1.19)
Other respiratory and intrathoracic	502	434.3	1.16 (1.06, 1.26)
Oesophagus	1158	1007.3	1.15 (1.09, 1.22)
Melanoma of skin	2573	2277.7	1.13 (1.09, 1.17)
Other endocrine gland	103	92.1	1.12 (0.92, 1.36)
Tumours of central nervous system	1737	1608.4	1.08 (1.03, 1.13)
Other leukaemia	889	832.1	1.07 (1.00, 1.14)
Lymphoma	2559	2436.2	1.05 (1.01, 1.09)
Myeloma	901	924.9	0.97 (0.91, 1.04)
Cervix	535	573.2	0.93 (0.86, 1.02)
Malignant neoplasm of unspecified sites or multiple sites	1289	1645.5	0.78 (0.74, 0.83)

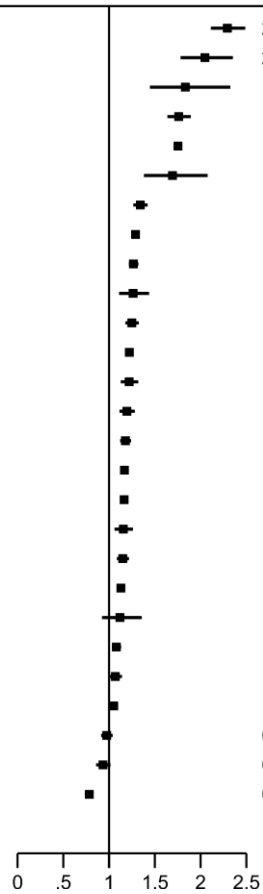

b) Absolute excess rates (AER per 10,000 person–years)

Type of second cancer	Observed	Expected	AER (95% CI)
Uterine	6343	3618.9	4.85 (4.57, 5.15)
Lung	9884	8090.5	3.19 (2.88, 3.60)
Colorectal	7865	6753.2	1.98 (1.68, 2.28)
Ovary or fallopian tubes	4207	3262.4	1.68 (1.45, 1.92)
Pancreas	2238	1767	0.84 (0.69, 1.01)
Urinary system	3022	2587.7	0.77 (0.60, 0.97)
Soft tissue	569	248.2	0.57 (0.49, 0.66)
Acute myeloid leukaemia	724	411.3	0.56 (0.47, 0.65)
Melanoma of skin	2573	2277.7	0.53 (0.36, 0.69)
Stomach	1118	833.8	0.51 (0.39, 0.62)
Other gastrointestinal	1492	1265.9	0.40 (0.27, 0.54)
Other head and neck	1112	890.4	0.39 (0.29, 0.51)
Oesophagus	1158	1007.3	0.27 (0.16, 0.39)
Tumours of central nervous system	1737	1608.4	0.23 (0.09, 0.37)
Lymphoma	2559	2436.2	0.22 (0.04, 0.39)
Liver	776	649.7	0.22 (0.13, 0.32)
Thyroid	622	510.1	0.20 (0.12, 0.29)
Salivary gland	198	96.7	0.18 (0.13, 0.23)
Other respiratory and intrathoracic	502	434.3	0.12 (0.05, 0.20)
Other leukaemia	889	832.1	0.10 (0.00, 0.21)
Other specified sites	229	181.4	0.08 (0.04, 0.14)
Bones and joints	92	54.3	0.07 (0.04, 0.10)
Acute lymphoblastic leukaemia	68	37.1	0.06 (0.03, 0.09)
Other endocrine gland	103	92.1	0.02 (-0.01, 0.06)
Myeloma	901	924.9	-0.04 (-0.15, 0.07)
Cervix	535	573.2	-0.07 (-0.14, 0.02)
Malignant neoplasm of unspecified sites or multiple sites	1289	1645.5	-0.63 (-0.76, -0.50)

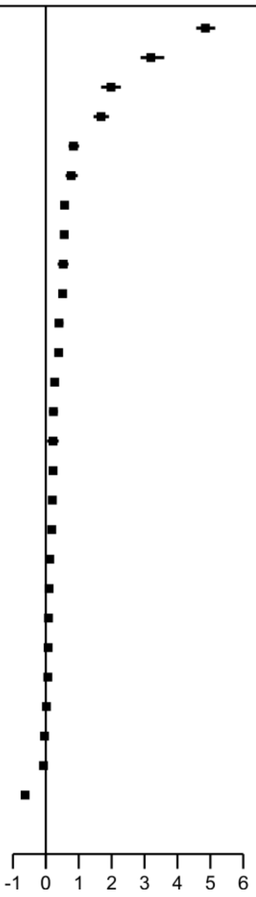

Figure S9: Standardised incidence ratios (a) and absolute excess rates (b) by type of non-breast invasive second cancer occurring at any time after diagnosis of the index breast cancer (ie including second, third, and later new invasive primaries). Cohort rates, from combining second and all subsequent cancers, are compared to all England rates for the same calendar year, attained age and quintile of deprivation.

ANALYSIS OF INVASIVE CANCERS

Part b: Comparisons between subgroups within the cohort

Non-breast cancer

Characteristic	Number of cancers /women	Rate ratio (95% CI)
**Year breast cancer diagnosed (p=0.00017)**		
1993-1999	13304/102105	0.94 (0.91, 0.97)
2000-2004	12136/102072	0.95 (0.92, 0.98)
2005-2009	11124/113521	1.00 (1.00, 1.00)
2010-2016	8903/158675	1.00 (0.97, 1.03)
**Age at breast cancer diagnosis (years) (p=<0.00001)**		
20-39	1049/29300	0.20 (0.19, 0.22)
40-49	5803/95844	0.35 (0.34, 0.37)
50-59	14594/148458	0.65 (0.63, 0.66)
60-69	17707/145004	1.00 (1.00, 1.00)
70-75	6314/57767	1.24 (1.20, 1.29)
**Cancer screen detected (p=0.27)**		
Screen detected	14193/135903	1.00 (1.00, 1.00)
Not screen detetected	15382/141096	1.01 (0.99, 1.04)
Not eligible	15892/199374	1.12 (1.08, 1.17)
**Tumour size (p=0.05)**		
1-20 mm	24197/240040	1.00 (1.00, 1.00)
21-50 mm	11546/134078	1.02 (1.00, 1.05)
>50 mm	1046/15054	1.03 (0.97, 1.10)
Unknown	8678/87201	0.98 (0.95, 1.00)
**Positive nodes (p=0.98)**		
0	14880/171886	1.00 (1.00, 1.00)
1 to 3	6322/80497	0.97 (0.94, 1.00)
4 to 9	1618/23031	1.02 (0.96, 1.07)
10 or more	589/10098	1.05 (0.96, 1.14)
Unknown	22058/190861	1.01 (0.98, 1.04)
**Tumour grade (p=0.00007)**		
Low grade	9266/84782	1.00 (1.00, 1.00)
Medium grade	20083/211845	1.01 (0.98, 1.04)
High grade	12134/145446	1.06 (1.03, 1.09)
Unknown	3984/34300	1.02 (0.98, 1.06)
**Focality (p=0.03)**		
Single	7836/82926	1.00 (1.00, 1.00)
Multiple	1438/21527	0.94 (0.88, 1.00)
Unknown	36193/371920	0.97 (0.93, 1.01)
**Breast quadrant (p=0.84)**		
Lateral	14328/153462	1.00 (1.00, 1.00)
Central	2280/24199	1.01 (0.97, 1.06)
Medial	5225/57049	1.00 (0.97, 1.03)
Overlapping	2421/26740	0.98 (0.94, 1.03)
Unknown	21213/214923	0.99 (0.97, 1.01)
**Cancer type (p=0.009)**		
Carcinoma NST	35685/382077	1.00 (1.00, 1.00)
Lobular	5306/52903	1.01 (0.98, 1.04)
Medullary	275/2385	1.05 (0.93, 1.18)
Metaplastic	127/1225	1.37 (1.15, 1.63)
Mucinous	890/7789	1.04 (0.97, 1.11)
Papillary	199/1750	1.03 (0.89, 1.18)
Tubular	1319/11853	0.95 (0.89, 1.00)
Other/unspecified	1666/16391	0.98 (0.93, 1.03)
**ER status and endocrine use (p=0.90)**		
ER-positive (or endocrine)	23993/261142	1.00 (1.00, 1.00)
ER-negative	1408/22175	1.00 (0.95, 1.06)
Unknown	20066/193056	0.99 (0.97, 1.01)
**Deprivation index (p=<0.00001)**		
<20%, least deprived	9883/111780	1.00 (1.00, 1.00)
20-39%	10104/109012	1.06 (1.03, 1.09)
40-59%	9470/98973	1.11 (1.08, 1.14)
60-79%	8440/85877	1.18 (1.15, 1.22)
80+%, most deprived	7570/70731	1.35 (1.31, 1.40)
**Time since breast cancer diagnosis (years) (p=<0.00001)**		
3m-4	13400/2113101	0.53 (0.51, 0.54)
5-9	14376/1654503	0.74 (0.72, 0.76)
10-14	10154/945119	1.00 (1.00, 1.00)
15-19	5347/443013	1.28 (1.24, 1.33)
20-24	1938/151131	1.57 (1.49, 1.65)
25-29	252/19702	1.80 (1.58, 2.04)

Contralateral breast cancer

Characteristic	Number of cancers /women	Rate ratio (95% CI)
**Year breast cancer diagnosed (p=<0.00001)**		
1993-1999	5642/102105	1.03 (0.98, 1.08)
2000-2004	5266/102072	1.05 (1.01, 1.10)
2005-2009	4421/113521	1.00 (1.00, 1.00)
2010-2016	2798/158675	0.82 (0.78, 0.86)
**Age at breast cancer diagnosis (years) (p=<0.00001)**		
20-39	1598/29300	1.42 (1.31, 1.54)
40-49	4168/95844	1.16 (1.08, 1.25)
50-59	6405/148458	1.02 (0.98, 1.07)
60-69	4693/145004	1.00 (1.00, 1.00)
70-75	1263/57767	1.07 (0.99, 1.16)
**Cancer screen detected (p=0.00005)**		
Screen detected	4580/135903	1.00 (1.00, 1.00)
Not screen detetected	5853/141096	1.09 (1.04, 1.13)
Not eligible	7694/199374	0.93 (0.86, 1.00)
**Tumour size (p=<0.00001)**		
1-20 mm	8829/240040	1.00 (1.00, 1.00)
21-50 mm	4758/134078	1.09 (1.05, 1.13)
>50 mm	589/15054	1.40 (1.29, 1.53)
Unknown	3951/87201	1.06 (1.01, 1.11)
**Positive nodes (p=0.00002)**		
0	5488/171886	1.00 (1.00, 1.00)
1 to 3	2572/80497	0.95 (0.90, 1.00)
4 to 9	798/23031	1.13 (1.04, 1.22)
10 or more	344/10098	1.42 (1.27, 1.59)
Unknown	8925/190861	1.01 (0.96, 1.06)
**Tumour grade (p=0.93)**		
Low grade	3488/84782	1.00 (1.00, 1.00)
Medium grade	7539/211845	0.96 (0.92, 1.00)
High grade	5413/145446	0.99 (0.95, 1.04)
Unknown	1687/34300	0.96 (0.90, 1.03)
**Focality (p=0.61)**		
Single	3328/82926	1.00 (1.00, 1.00)
Multiple	674/21527	1.02 (0.94, 1.12)
Unknown	14125/371920	0.92 (0.86, 0.98)
**Breast quadrant (p=0.01)**		
Lateral	5520/153462	1.00 (1.00, 1.00)
Central	924/24199	1.06 (0.99, 1.14)
Medial	2064/57049	1.08 (1.03, 1.14)
Overlapping	1027/26740	1.04 (0.97, 1.11)
Unknown	8592/214923	1.03 (1.00, 1.07)
**Cancer type (p=0.00001)**		
Carcinoma NST	14176/382077	1.00 (1.00, 1.00)
Lobular	2171/52903	1.12 (1.06, 1.17)
Medullary	156/2385	1.15 (0.98, 1.34)
Metaplastic	51/1225	1.28 (0.97, 1.69)
Mucinous	245/7789	0.86 (0.76, 0.98)
Papillary	70/1750	1.11 (0.88, 1.41)
Tubular	545/11853	1.00 (0.91, 1.09)
Other/unspecified	713/16391	0.98 (0.91, 1.06)
**ER status and endocrine use (p=<0.00001)**		
ER-positive (or endocrine)	8486/261142	1.00 (1.00, 1.00)
ER-negative	801/22175	1.54 (1.42, 1.66)
Unknown	8840/193056	1.12 (1.09, 1.16)
**Deprivation index (p=0.04)**		
<20%, least deprived	4286/111780	1.00 (1.00, 1.00)
20-39%	4258/109012	1.04 (1.00, 1.09)
40-59%	3673/98973	1.00 (0.96, 1.05)
60-79%	3346/85877	1.08 (1.03, 1.13)
80+%, most deprived	2564/70731	1.03 (0.98, 1.09)
**Time since breast cancer diagnosis (years) (p=<0.00001)**		
3m-4	5009/2113101	0.54 (0.52, 0.56)
5-9	5622/1654503	0.77 (0.74, 0.80)
10-14	4408/945119	1.00 (1.00, 1.00)
15-19	2239/443013	1.06 (1.01, 1.12)
20-24	753/151131	1.05 (0.97, 1.14)
25-29	96/19702	1.03 (0.84, 1.26)

Figure S10: Adjusted rate ratios for the incidence of non-breast cancer and contralateral breast cancer by patient factors and characteristics of the index breast cancer. All factors are adjusted for each other as well as for geographical region. The p-values exclude unknown levels and where factors are ordinal they are tests for trend, otherwise they are tests for heterogeneity. (All ER+ve patients are included in the endocrine recorded group.)
Abbreviations: ER=oestrogen-receptor, NST=no special type**.**

Type of second cancer	Number of cancers	Average adjusted rate per 10,000 woman-years IMD quintile 1	2	3	4	5	Adjusted rate ratio per 1 IMD category (95% CI)
**Second cancers where p-value for trend ≤ 0.05**							
Lung	8741	10.5	13.3	15.9	19.5	27.7	1.26 (1.24, 1.28)
Cervix	488	0.64	0.85	0.74	1.09	1.50	1.22 (1.14, 1.30)
Stomach	992	1.43	1.53	1.80	2.15	2.73	1.18 (1.13, 1.23)
Oesophagus	1015	1.55	1.56	1.89	2.27	2.53	1.15 (1.10, 1.20)
Other head and neck	938	1.43	1.63	1.84	1.74	2.36	1.11 (1.06, 1.17)
Urinary system	2623	4.13	4.51	4.66	5.81	6.02	1.11 (1.08, 1.14)
Malignant neoplasm of unspecified sites	1153	1.85	1.95	2.06	2.47	2.66	1.10 (1.05, 1.15)
Other respiratory and intrathoracic	445	0.75	0.73	0.79	0.98	1.01	1.09 (1.02, 1.17)
Liver	665	1.17	1.07	1.31	1.18	1.61	1.08 (1.02, 1.14)
All non-breast cancer	45467	75.7	80.3	84.1	89.5	102.4	1.07 (1.06, 1.08)
Pancreas	1993	3.53	3.51	3.65	3.79	4.38	1.05 (1.01, 1.08)
Uterine	5793	10.0	10.5	10.8	11.3	11.9	1.04 (1.02, 1.06)
Ovary or fallopian tubes	3729	6.46	6.99	6.74	7.27	7.59	1.04 (1.01, 1.06)
Contralateral breast cancer	18127	32.8	34.1	32.9	35.5	33.8	1.01 (1.00, 1.02)
Melanoma of skin	2319	5.31	4.70	4.40	3.69	2.57	0.86 (0.83, 0.89)
**Second cancers where p-value for trend > 0.05**							
Other gastrointestinal	1276	2.21	2.40	2.38	2.31	2.69	1.04 (0.99, 1.08)
Other specified sites	202	0.36	0.30	0.50	0.35	0.38	1.03 (0.93, 1.14)
Lymphoma	2180	3.92	4.02	3.92	4.17	4.46	1.03 (1.00, 1.06)
Tumours of central nervous system	1549	2.79	2.81	3.01	2.98	2.85	1.01 (0.98, 1.05)
Colorectal	6835	12.7	12.5	13.0	12.4	13.1	1.00 (0.99, 1.02)
Other leukaemia	781	1.28	1.65	1.47	1.51	1.31	1.00 (0.95, 1.06)
Thyroid	545	1.10	0.96	0.94	0.91	1.19	1.00 (0.94, 1.07)
Soft tissue	468	0.89	0.84	0.89	0.88	0.86	1.00 (0.93, 1.07)
Myeloma	823	1.49	1.62	1.55	1.43	1.56	1.00 (0.95, 1.05)
Salivary gland	177	0.30	0.43	0.25	0.33	0.33	0.98 (0.88, 1.10)
Acute myeloid leukaemia	651	1.22	1.28	1.23	1.17	1.12	0.98 (0.92, 1.03)
Bones and joints	79	0.15	0.16	0.16	0.13	0.11	0.93 (0.79, 1.10)
Other endocrine gland	90	0.19	0.20	0.16	0.13	0.13	0.88 (0.75, 1.04)
Acute lymphoblastic leukaemia	63	0.10	0.19	0.12	0.07	0.07	0.86 (0.71, 1.05)

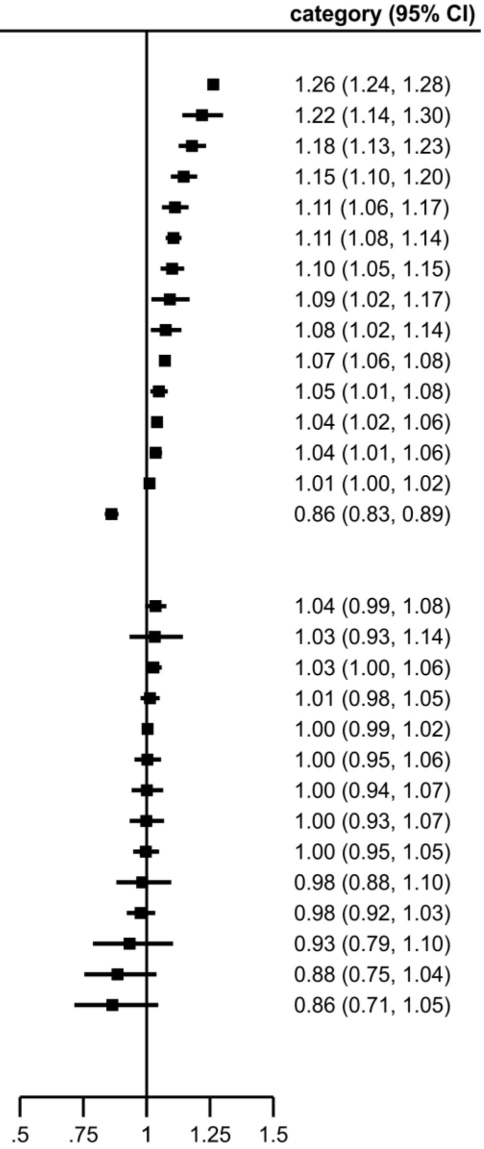

Figure S11: Adjusted rate ratios (RR) for the incidence of different second cancer groups per quintile increase in the index of multiple deprivation (IMD). Cancers are grouped according to whether the p-value for trend in the RR by IMD quintile reached conventional significance (p≤0.05, top panel) or not (bottom panel). Rate ratios are adjusted for the same factors as in Figure S10. Average adjusted rates per 10,000 woman-years are given for each IMD quintile (1=least deprived, 5=most deprived).

Type of second cancer	Number of cancers	Average adjusted rate per 10,000 woman years<br>Year index breast cancer diagnosed<br>1993-99	2000-04	2005-09	2010-11	Adjusted rate ratio per 5-year index breast cancer diagnosis period (95% CI)
**Second cancers where p-value for trend ≤ 0.05**						
Other endocrine gland	44	0.10	0.06	0.23	0.23	1.47 (1.04, 2.08)
Thyroid	261	0.55	0.72	1.05	1.34	1.36 (1.18, 1.57)
Other head and neck	444	1.04	1.35	1.64	2.38	1.30 (1.16, 1.46)
Liver	282	0.58	0.82	1.22	1.07	1.26 (1.09, 1.46)
Other gastrointestinal	554	1.38	1.62	2.23	2.27	1.21 (1.09, 1.34)
Other leukaemia	370	0.96	1.09	1.48	1.52	1.19 (1.05, 1.35)
Lung	3932	10.3	12.0	15.0	16.0	1.17 (1.13, 1.22)
Melanoma of skin	1135	3.07	3.63	4.21	4.26	1.13 (1.05, 1.21)
Tumours of central nervous system	785	2.25	2.45	2.78	3.17	1.12 (1.03, 1.22)
All non-breast cancer	22275	69.4	71.7	77.9	77.0	1.04 (1.03, 1.06)
Contralateral breast cancer	8955	30.0	31.0	29.9	25.5	0.96 (0.94, 0.99)
Ovary or fallopian tubes	2001	7.05	7.12	6.38	5.39	0.92 (0.88, 0.97)
Uterine	3089	12.4	10.1	8.89	9.42	0.89 (0.85, 0.93)
Stomach	548	2.15	1.96	1.63	1.27	0.85 (0.77, 0.94)
**Second cancers where p-value for trend > 0.05**						
Salivary gland	88	0.25	0.24	0.38	0.27	1.12 (0.87, 1.43)
Bones and joints	49	0.12	0.16	0.21	0.14	1.11 (0.79, 1.56)
Malignant neoplasm of unspecified sites	553	1.54	1.79	2.06	1.97	1.11 (1.00, 1.23)
Acute myeloid leukaemia	376	1.08	1.28	1.25	1.51	1.10 (0.97, 1.24)
Myeloma	398	1.15	1.32	1.37	1.50	1.09 (0.97, 1.22)
Other respiratory and intrathoracic	225	0.55	0.84	0.88	0.61	1.07 (0.92, 1.25)
Urinary system	1281	3.88	4.03	4.71	4.28	1.06 (0.99, 1.13)
Pancreas	874	2.47	3.02	3.17	2.77	1.06 (0.97, 1.15)
Other specified sites	91	0.26	0.33	0.32	0.30	1.06 (0.82, 1.35)
Soft tissue	268	0.83	0.82	1.00	0.86	1.05 (0.91, 1.21)
Lymphoma	1043	3.40	3.11	3.69	3.72	1.04 (0.97, 1.12)
Colorectal	3339	10.7	11.0	11.6	10.6	1.01 (0.97, 1.05)
Cervix	273	0.95	0.92	0.84	0.90	0.96 (0.84, 1.11)
Oesophagus	478	1.77	1.47	1.60	1.43	0.94 (0.85, 1.05)
Acute lymphoblastic leukaemia	41	0.12	0.19	0.13	0.07	0.89 (0.61, 1.28)

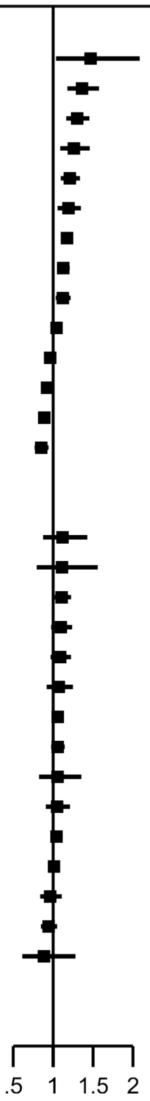

Figure S12: Adjusted rate ratios (RR) for the incidence of different second cancer groups per 5-year increase in the calendar year the index breast cancer was diagnosed. To reduce confounding by length of follow-up (see Figure S7) analysis was restricted to the first 10-years after diagnosis and breast cancer diagnosis periods 1993-2011. Cancers are grouped according to whether the p-value for trend in the RR by 5-year calendar year period reached conventional significance (p≤0.05, top panel) or not (bottom panel). Rate ratios are adjusted for the same factors as in Figure S10. Average adjusted rates per 10,000 woman-years are given for each calendar period of index breast cancer diagnosis.

Unadjusted percentages

Characteristic	Recorded /women	Unadjusted percent (95% CI)
**Year breast cancer diagnosed (p=<0.00001)**		
1993-1999	70634/102105	69.2 (68.9, 69.5)
2000-2004	69142/102072	67.7 (67.5, 68.0)
2005-2009	84091/113521	74.1 (73.8, 74.3)
2010-2016	131235/158675	82.7 (82.5, 82.9)
**Age at breast cancer diagnosis (years) (p=<0.00001)**		
20-39	20960/29300	71.5 (71.0, 72.1)
40-49	69941/95844	73.0 (72.7, 73.3)
50-59	113804/148458	76.7 (76.4, 76.9)
60-69	111288/145004	76.7 (76.5, 77.0)
70-75	39109/57767	67.7 (67.3, 68.1)
**Cancer screen detected (p=<0.00001)**		
Screen-detected	113431/135903	83.5 (83.3, 83.7)
Not screen-detetected	101882/141096	72.2 (72.0, 72.4)
Not eligible	139789/199374	70.1 (69.9, 70.3)
**Tumour size (mm) (p=<0.00001)**		
1-20	193335/240040	80.5 (80.4, 80.7)
21-50	93614/134078	69.8 (69.6, 70.1)
>50	10250/15054	68.1 (67.3, 68.8)
Unknown	57903/87201	66.4 (66.1, 66.7)
**Positive nodes (p=<0.00001)**		
0	135837/171886	79.0 (78.8, 79.2)
1 to 3	61746/80497	76.7 (76.4, 77.0)
4 to 9	17809/23031	77.3 (76.8, 77.9)
10 or more	7485/10098	74.1 (73.3, 75.0)
Unknown	132225/190861	69.3 (69.1, 69.5)
**Tumour grade (p=<0.00001)**		
Low	68621/84782	80.9 (80.7, 81.2)
Medium	156629/211845	73.9 (73.7, 74.1)
High	107171/145446	73.7 (73.5, 73.9)
Unknown	22681/34300	66.1 (65.6, 66.6)
**Focality (p=<0.00001)**		
Single	66505/82926	80.2 (79.9, 80.5)
Multiple	13179/21527	61.2 (60.6, 61.9)
Unknown	275418/371920	74.1 (73.9, 74.2)
**Breast quadrant (p=<0.00001)**		
Lateral	118987/153462	77.5 (77.3, 77.7)
Central	14920/24199	61.7 (61.0, 62.3)
Medial	43954/57049	77.0 (76.7, 77.4)
Overlapping	19180/26740	71.7 (71.2, 72.3)
Unknown	158061/214923	73.5 (73.4, 73.7)
**Cancer type (p=<0.00001)**		
Carcinoma NST	288214/382077	75.4 (75.3, 75.6)
Lobular	35490/52903	67.1 (66.7, 67.5)
Medullary	1950/2385	81.8 (80.2, 83.3)
Metaplastic	914/1225	74.6 (72.2, 77.0)
Mucinous	5738/7789	73.7 (72.7, 74.6)
Papillary	1299/1750	74.2 (72.2, 76.3)
Tubular	10184/11853	85.9 (85.3, 86.5)
Other/unspecified	11313/16391	69.0 (68.3, 69.7)
**Deprivation index (p=<0.00001)**		
<20%, least deprived	85355/111780	76.4 (76.1, 76.6)
20-39%	82010/109012	75.2 (75.0, 75.5)
40-59%	74263/98973	75.0 (74.8, 75.3)
60-79%	63227/85877	73.6 (73.3, 73.9)
80+%, most deprived	50247/70731	71.0 (70.7, 71.4)
**Geograhical region (p=<0.00001)**		
Eastern	43027/54322	79.2 (78.9, 79.5)
North West	42537/64383	66.1 (65.7, 66.4)
Northern & Yorkshire	47259/63058	74.9 (74.6, 75.3)
Oxford	25008/29621	84.4 (84.0, 84.8)
South West	56616/73731	76.8 (76.5, 77.1)
Thames	73757/98920	74.6 (74.3, 74.8)
Trent	25978/39555	65.7 (65.2, 66.1)
West Midlands	40920/52783	77.5 (77.2, 77.9)
**Endocrine therapy (p=<0.00001)**		
Not recorded	150758/215231	70.0 (69.9, 70.2)
Recorded or ER-positive	204344/261142	78.3 (78.1, 78.4)
**Chemotherapy (p=<0.00001)**		
Not recorded	228844/308253	74.2 (74.1, 74.4)
Recorded	126258/168120	75.1 (74.9, 75.3)
**All women (p=<0.00001)**		
Overall average	355102/476373	74.5 (74.4, 74.7)

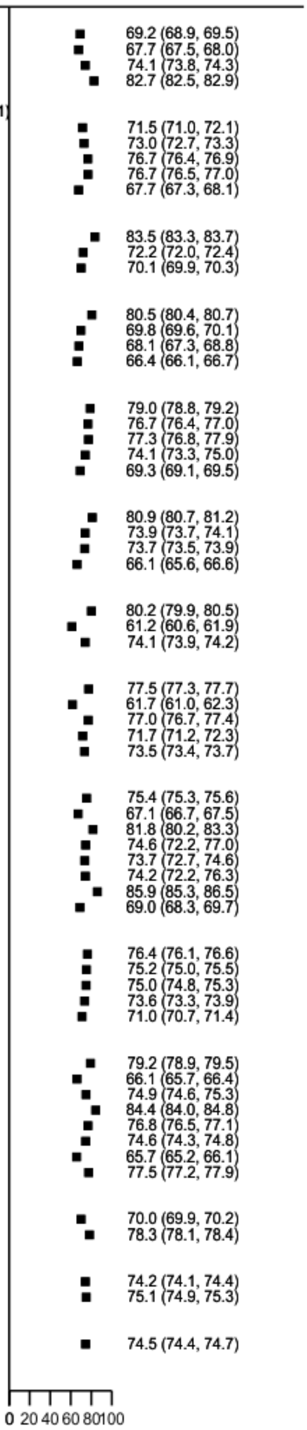

Adjusted percentages

Characteristic	Recorded /women	Adjusted percent (95% CI)
**Year breast cancer diagnosed (p=<0.00001)**		
1993-1999	70634/102105	70.4 (70.1, 70.7)
2000-2004	69142/102072	68.8 (68.5, 69.1)
2005-2009	84091/113521	73.5 (73.2, 73.7)
2010-2016	131235/158675	82.1 (81.9, 82.3)
**Age at breast cancer diagnosis (years) (p=<0.00001)**		
20-39	20960/29300	75.4 (74.8, 75.9)
40-49	69941/95844	75.9 (75.5, 76.3)
50-59	113804/148458	74.4 (74.1, 74.7)
60-69	111288/145004	74.6 (74.3, 74.8)
70-75	39109/57767	72.1 (71.7, 72.6)
**Cancer screen detected (p=<0.00001)**		
Screen-detected	113431/135903	80.4 (80.1, 80.7)
Not screen-detetected	101882/141096	73.8 (73.5, 74.1)
Not eligible	139789/199374	71.6 (71.3, 71.9)
**Tumour size (mm) (p=<0.00001)**		
1-20	193335/240040	78.8 (78.7, 79.0)
21-50	93614/134078	70.0 (69.8, 70.3)
>50	10250/15054	68.8 (68.1, 69.5)
Unknown	57903/87201	71.7 (71.3, 72.0)
**Positive nodes (p=<0.00001)**		
0	135837/171886	72.4 (72.1, 72.7)
1 to 3	61746/80497	75.3 (75.0, 75.6)
4 to 9	17809/23031	79.4 (78.9, 79.9)
10 or more	7485/10098	78.1 (77.3, 78.8)
Unknown	132225/190861	74.9 (74.7, 75.1)
**Tumour grade (p=<0.00001)**		
Low	68621/84782	78.1 (77.8, 78.4)
Medium	156629/211845	73.5 (73.3, 73.7)
High	107171/145446	74.3 (74.1, 74.5)
Unknown	22681/34300	73.8 (73.3, 74.2)
**Focality (p=<0.00001)**		
Single	66505/82926	77.2 (76.8, 77.6)
Multiple	13179/21527	55.3 (54.7, 56.0)
Unknown	275418/371920	75.0 (74.9, 75.2)
**Breast quadrant (p=<0.00001)**		
Lateral	118987/153462	76.8 (76.5, 77.0)
Central	14920/24199	61.4 (60.8, 62.0)
Medial	43954/57049	75.6 (75.3, 76.0)
Overlapping	19180/26740	71.1 (70.6, 71.6)
Unknown	158061/214923	74.6 (74.4, 74.8)
**Cancer type (p=<0.00001)**		
Carcinoma NST	288214/382077	75.1 (75.0, 75.2)
Lobular	35490/52903	69.1 (68.7, 69.5)
Medullary	1950/2385	84.4 (83.0, 85.7)
Metaplastic	914/1225	78.0 (75.9, 80.1)
Mucinous	5738/7789	73.9 (73.0, 74.9)
Papillary	1299/1750	75.4 (73.5, 77.4)
Tubular	10184/11853	81.7 (81.0, 82.5)
Other/unspecified	11313/16391	74.0 (73.3, 74.6)
**Deprivation index (p=<0.00001)**		
<20%, least deprived	85355/111780	75.3 (75.1, 75.6)
20-39%	82010/109012	74.7 (74.5, 75.0)
40-59%	74263/98973	74.9 (74.6, 75.2)
60-79%	63227/85877	74.2 (73.9, 74.5)
80+%, most deprived	50247/70731	73.1 (72.8, 73.4)
**Geograhical region (p=<0.00001)**		
Eastern	43027/54322	78.7 (78.4, 79.1)
North West	42537/64383	68.0 (67.6, 68.3)
Northern & Yorkshire	47259/63058	72.4 (72.0, 72.8)
Oxford	25008/29621	85.2 (84.8, 85.6)
South West	56616/73731	78.5 (78.2, 78.8)
Thames	73757/98920	73.7 (73.4, 74.0)
Trent	25978/39555	67.3 (66.8, 67.7)
West Midlands	40920/52783	76.5 (76.0, 76.9)
**Endocrine therapy (p=<0.00001)**		
Not recorded	150758/215231	72.3 (72.1, 72.5)
Recorded or ER-positive	204344/261142	76.6 (76.5, 76.8)
**Chemotherapy (p=<0.00001)**		
Not recorded	228844/308253	73.4 (73.3, 73.6)
Recorded	126258/168120	76.5 (76.2, 76.7)
**All women (p=<0.00001)**		
Overall average	355102/476373	74.5 (74.4, 74.7)

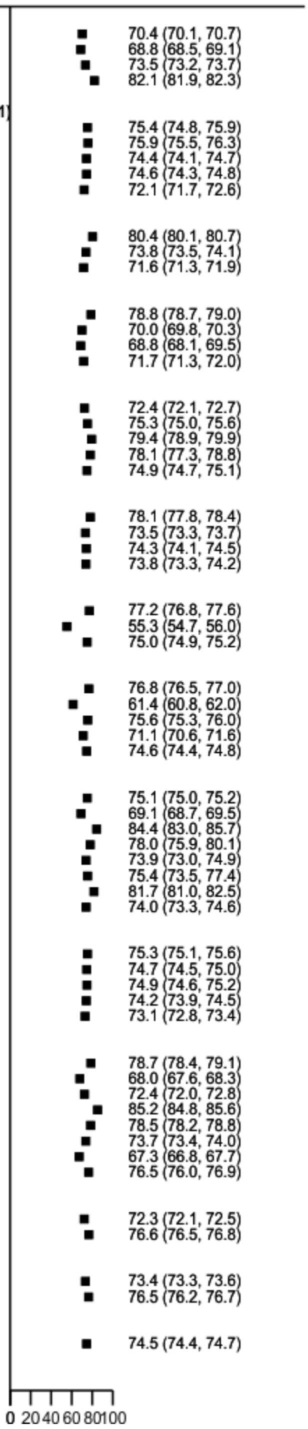

Figure S13: Recording of the use of radiotherapy (or BCS) to treat the index breast cancer, by patient and tumour characteristics. In the adjusted percent panel all factors are adjusted for each other. The p-values exclude unknown levels and where factors are ordinal it's a test for trend, otherwise it's a test for heterogeneity. (All patients treated with BCS are included in the radiotherapy recorded group and all ER+ve patients are included in the endocrine recorded group.)

Abbreviations: BCS=breast-conserving surgery, ER=oestrogen-receptor, NST=no special type

Unadjusted percentages

Characteristic	Recorded /women	Unadjusted percent (95% CI)
**Year breast cancer diagnosed (p=<0.00001)**		
1993-1999	49794/102105	48.8 (48.5, 49.1)
2000-2004	44501/102072	43.6 (43.3, 43.9)
2005-2009	50872/113521	44.8 (44.5, 45.1)
2010-2016	115975/158675	73.1 (72.9, 73.3)
**Age at breast cancer diagnosis (years) (p=<0.00001)**		
20-39	12296/29300	42.0 (41.4, 42.5)
40-49	49324/95844	51.5 (51.1, 51.8)
50-59	81688/148458	55.0 (54.8, 55.3)
60-69	85161/145004	58.7 (58.5, 59.0)
70-75	32673/57767	56.6 (56.2, 57.0)
**Cancer screen detected (p=<0.00001)**		
Screen-detected	88175/135903	64.9 (64.6, 65.1)
Not screen-detetected	71520/141096	50.7 (50.4, 50.9)
Not eligible	101447/199374	50.9 (50.7, 51.1)
**Tumour size (mm) (p=<0.00001)**		
1-20	144920/240040	60.4 (60.2, 60.6)
21-50	72963/134078	54.4 (54.2, 54.7)
>50	7966/15054	52.9 (52.1, 53.7)
Unknown	35293/87201	40.5 (40.1, 40.8)
**Positive nodes (p=<0.00001)**		
0	108410/171886	63.1 (62.8, 63.3)
1 to 3	46930/80497	58.3 (58.0, 58.6)
4 to 9	12266/23031	53.3 (52.6, 53.9)
10 or more	5191/10098	51.4 (50.4, 52.4)
Unknown	88345/190861	46.3 (46.1, 46.5)
**Tumour grade (p=<0.00001)**		
Low	53959/84782	63.6 (63.3, 64.0)
Medium	132057/211845	62.3 (62.1, 62.5)
High	61754/145446	42.5 (42.2, 42.7)
Unknown	13372/34300	39.0 (38.5, 39.5)
**Focality (p=<0.00001)**		
Single	61198/82926	73.8 (73.5, 74.1)
Multiple	14765/21527	68.6 (68.0, 69.2)
Unknown	185179/371920	49.8 (49.6, 50.0)
**Breast quadrant (p=<0.00001)**		
Lateral	90709/153462	59.1 (58.9, 59.4)
Central	14342/24199	59.3 (58.6, 59.9)
Medial	34523/57049	60.5 (60.1, 60.9)
Overlapping	15930/26740	59.6 (59.0, 60.2)
Unknown	105638/214923	49.2 (48.9, 49.4)
**Cancer type (p=<0.00001)**		
Carcinoma NST	208246/382077	54.5 (54.3, 54.7)
Lobular	32686/52903	61.8 (61.4, 62.2)
Medullary	714/2385	29.9 (28.1, 31.8)
Metaplastic	201/1225	16.4 (14.3, 18.5)
Mucinous	4905/7789	63.0 (61.9, 64.0)
Papillary	959/1750	54.8 (52.5, 57.1)
Tubular	7192/11853	60.7 (59.8, 61.6)
Other/unspecified	6239/16391	38.1 (37.3, 38.8)
**Deprivation index (p=0.009)**		
<20%, least deprived	60511/111780	54.1 (53.8, 54.4)
20-39%	60928/109012	55.9 (55.6, 56.2)
40-59%	54660/98973	55.2 (54.9, 55.5)
60-79%	47056/85877	54.8 (54.5, 55.1)
80+%, most deprived	37987/70731	53.7 (53.3, 54.1)
**Geograhical region (p=<0.00001)**		
Eastern	41108/54322	75.7 (75.3, 76.0)
North West	23698/64383	36.8 (36.4, 37.2)
Northern & Yorkshire	49453/63058	78.4 (78.1, 78.7)
Oxford	10847/29621	36.6 (36.1, 37.2)
South West	27265/73731	37.0 (36.6, 37.3)
Thames	54034/98920	54.6 (54.3, 54.9)
Trent	16459/39555	41.6 (41.1, 42.1)
West Midlands	38278/52783	72.5 (72.1, 72.9)
**Surgery and radiotherapy (p=<0.00001)**		
BCS	170141/297122	57.3 (57.1, 57.4)
Mast+RT	34203/57980	59.0 (58.6, 59.4)
Mast-RT	56798/121271	46.8 (46.6, 47.1)
**Chemotherapy (p=<0.00001)**		
Not recorded	179669/308253	58.3 (58.1, 58.5)
Recorded	81473/168120	48.5 (48.2, 48.7)
**All women (p=<0.00001)**		
Overall average	261142/476373	54.8 (54.7, 55.0)

0 20 40 60 80 100

Adjusted percentages

Characteristic	Recorded /women	Adjusted percent (95% CI)
**Year breast cancer diagnosed (p=<0.00001)**		
1993-1999	49794/102105	51.4 (51.1, 51.7)
2000-2004	44501/102072	45.0 (44.7, 45.3)
2005-2009	50872/113521	44.8 (44.5, 45.0)
2010-2016	115975/158675	70.6 (70.4, 70.8)
**Age at breast cancer diagnosis (years) (p=<0.00001)**		
20-39	12296/29300	49.7 (49.1, 50.3)
40-49	49324/95844	53.6 (53.2, 54.0)
50-59	81688/148458	55.4 (55.1, 55.7)
60-69	85161/145004	56.0 (55.7, 56.3)
70-75	32673/57767	55.0 (54.6, 55.5)
**Cancer screen detected (p=<0.00001)**		
Screen-detected	88175/135903	56.5 (56.2, 56.9)
Not screen-detetected	71520/141096	52.9 (52.6, 53.2)
Not eligible	101447/199374	55.0 (54.7, 55.4)
**Tumour size (mm) (p=0.001)**		
1-20	144920/240040	55.4 (55.2, 55.6)
21-50	72963/134078	55.2 (55.0, 55.5)
>50	7966/15054	53.7 (52.9, 54.4)
Unknown	35293/87201	52.8 (52.5, 53.2)
**Positive nodes (p=<0.00001)**		
0	108410/171886	54.8 (54.6, 55.1)
1 to 3	46930/80497	57.6 (57.3, 57.9)
4 to 9	12266/23031	57.1 (56.5, 57.7)
10 or more	5191/10098	56.0 (55.2, 56.9)
Unknown	88345/190861	53.3 (53.1, 53.5)
**Tumour grade (p=<0.00001)**		
Low	53959/84782	61.1 (60.8, 61.4)
Medium	132057/211845	59.8 (59.6, 60.0)
High	61754/145446	45.0 (44.8, 45.3)
Unknown	13372/34300	50.6 (50.1, 51.1)
**Focality (p=0.00092)**		
Single	61198/82926	62.1 (61.6, 62.5)
Multiple	14765/21527	60.8 (60.2, 61.5)
Unknown	185179/371920	52.9 (52.8, 53.1)
**Breast quadrant (p=<0.00001)**		
Lateral	90709/153462	57.2 (57.0, 57.4)
Central	14342/24199	59.1 (58.5, 59.6)
Medial	34523/57049	58.1 (57.7, 58.5)
Overlapping	15930/26740	58.4 (57.9, 58.9)
Unknown	105638/214923	51.3 (51.2, 51.5)
**Cancer type (p=<0.00001)**		
Carcinoma NST	208246/382077	54.9 (54.7, 55.0)
Lobular	32686/52903	58.6 (58.2, 59.0)
Medullary	714/2385	43.2 (41.4, 45.0)
Metaplastic	201/1225	18.3 (16.3, 20.3)
Mucinous	4905/7789	57.3 (56.4, 58.3)
Papillary	959/1750	53.1 (51.1, 55.1)
Tubular	7192/11853	54.0 (53.1, 54.8)
Other/unspecified	6239/16391	45.4 (44.8, 46.1)
**Deprivation index (p=<0.00001)**		
<20%, least deprived	60511/111780	54.9 (54.6, 55.1)
20-39%	60928/109012	55.4 (55.1, 55.6)
40-59%	54660/98973	55.0 (54.8, 55.3)
60-79%	47056/85877	54.8 (54.5, 55.1)
80+%, most deprived	37987/70731	53.6 (53.2, 53.9)
**Geograhical region (p=<0.00001)**		
Eastern	41108/54322	71.1 (70.7, 71.5)
North West	23698/64383	40.2 (39.9, 40.6)
Northern & Yorkshire	49453/63058	79.7 (79.4, 80.0)
Oxford	10847/29621	36.9 (36.3, 37.4)
South West	27265/73731	37.0 (36.7, 37.4)
Thames	54034/98920	57.1 (56.8, 57.4)
Trent	16459/39555	42.7 (42.2, 43.2)
West Midlands	38278/52783	65.9 (65.3, 66.4)
**Surgery and radiotherapy (p=<0.00001)**		
BCS	170141/297122	55.8 (55.6, 56.0)
Mast+RT	34203/57980	56.5 (56.1, 56.9)
Mast-RT	56798/121271	51.6 (51.3, 51.8)
**Chemotherapy (p=<0.00001)**		
Not recorded	179669/308253	57.3 (57.1, 57.4)
Recorded	81473/168120	50.4 (50.1, 50.6)
**All women (p=<0.00001)**		
Overall average	261142/476373	54.8 (54.7, 55.0)

0 20 40 60 80 100

Figure S14: Recording of the use of endocrine therapy to treat the index breast cancer, by patient and tumour characteristics. In the adjusted percent panel all factors are adjusted for each other. The p-values exclude unknown levels and where factors are ordinal it's a test for trend, otherwise it's a test for heterogeneity. (All patients treated with BCS are included in the radiotherapy recorded group and all ER+ve patients are included in the endocrine recorded group.)

Abbreviations: BCS=breast-conserving surgery, ER=oestrogen-receptor, NST=no special type

Unadjusted percentages

Characteristic	Recorded /women	Unadjusted percent (95% CI)
**Year breast cancer diagnosed (p=<0.00001)**		
1993-1999	25127/102105	24.6 (24.3, 24.9)
2000-2004	34382/102072	33.7 (33.4, 34.0)
2005-2009	45229/113521	39.8 (39.6, 40.1)
2010-2016	63382/158675	39.9 (39.7, 40.2)
**Age at breast cancer diagnosis (years) (p=<0.00001)**		
20-39	20394/29300	69.6 (69.1, 70.1)
40-49	54166/95844	56.5 (56.2, 56.8)
50-59	53906/148458	36.3 (36.1, 36.6)
60-69	33029/145004	22.8 (22.6, 23.0)
70-75	6625/57767	11.5 (11.2, 11.7)
**Cancer screen detected (p=<0.00001)**		
Screen-detected	30206/135903	22.2 (22.0, 22.4)
Not screen-detetected	55044/141096	39.0 (38.8, 39.3)
Not eligible	82870/199374	41.6 (41.3, 41.8)
**Tumour size (mm) (p=<0.00001)**		
1-20	58195/240040	24.2 (24.1, 24.4)
21-50	69873/134078	52.1 (51.8, 52.4)
>50	9650/15054	64.1 (63.3, 64.9)
Unknown	30402/87201	34.9 (34.5, 35.2)
**Positive nodes (p=<0.00001)**		
0	39982/171886	23.3 (23.1, 23.5)
1 to 3	48605/80497	60.4 (60.0, 60.7)
4 to 9	17089/23031	74.2 (73.6, 74.8)
10 or more	7777/10098	77.0 (76.2, 77.8)
Unknown	54667/190861	28.6 (28.4, 28.8)
**Tumour grade (p=<0.00001)**		
Low	8198/84782	9.7 (9.5, 9.9)
Medium	61816/211845	29.2 (29.0, 29.4)
High	89555/145446	61.6 (61.3, 61.8)
Unknown	8551/34300	24.9 (24.5, 25.4)
**Focality (p=<0.00001)**		
Single	28650/82926	34.5 (34.2, 34.9)
Multiple	10413/21527	48.4 (47.7, 49.0)
Unknown	129057/371920	34.7 (34.5, 34.9)
**Breast quadrant (p=<0.00001)**		
Lateral	58192/153462	37.9 (37.7, 38.2)
Central	8888/24199	36.7 (36.1, 37.3)
Medial	18927/57049	33.2 (32.8, 33.6)
Overlapping	10139/26740	37.9 (37.3, 38.5)
Unknown	71974/214923	33.5 (33.3, 33.7)
**Cancer type (p=<0.00001)**		
Carcinoma NST	143679/382077	37.6 (37.5, 37.8)
Lobular	15559/52903	29.4 (29.0, 29.8)
Medullary	1195/2385	50.1 (48.1, 52.1)
Metaplastic	639/1225	52.2 (49.4, 55.0)
Mucinous	929/7789	11.9 (11.2, 12.6)
Papillary	277/1750	15.8 (14.1, 17.5)
Tubular	561/11853	4.7 (4.4, 5.1)
Other/unspecified	5281/16391	32.2 (31.5, 32.9)
**Deprivation index (p=<0.00001)**		
<20%, least deprived	37691/111780	33.7 (33.4, 34.0)
20-39%	37966/109012	34.8 (34.5, 35.1)
40-59%	34601/98973	35.0 (34.7, 35.3)
60-79%	31304/85877	36.5 (36.1, 36.8)
80+%, most deprived	26558/70731	37.5 (37.2, 37.9)
**Geograhical region (p=<0.00001)**		
Eastern	18054/54322	33.2 (32.8, 33.6)
North West	20017/64383	31.1 (30.7, 31.4)
Northern & Yorkshire	24690/63058	39.2 (38.8, 39.5)
Oxford	9276/29621	31.3 (30.8, 31.8)
South West	24207/73731	32.8 (32.5, 33.2)
Thames	38173/98920	38.6 (38.3, 38.9)
Trent	14889/39555	37.6 (37.2, 38.1)
West Midlands	18814/52783	35.6 (35.2, 36.1)
**Surgery and radiotherapy (p=<0.00001)**		
BCS	86176/297122	29.0 (28.8, 29.2)
Mast+RT	40082/57980	69.1 (68.8, 69.5)
Mast-RT	41862/121271	34.5 (34.3, 34.8)
**Endocrine therapy (p=<0.00001)**		
Not recorded	86647/215231	40.3 (40.1, 40.5)
Recorded or ER-positive	81473/261142	31.2 (31.0, 31.4)
**All women (p=<0.00001)**		
Overall average	168120/476373	35.3 (35.2, 35.4)

Axis: 0 20 40 60 80 100

Adjusted percentages

Characteristic	Recorded /women	Adjusted percent (95% CI)
**Year breast cancer diagnosed (p=<0.00001)**		
1993-1999	25127/102105	26.0 (25.8, 26.2)
2000-2004	34382/102072	33.3 (33.1, 33.6)
2005-2009	45229/113521	37.6 (37.4, 37.8)
2010-2016	63382/158675	41.5 (41.2, 41.7)
**Age at breast cancer diagnosis (years) (p=<0.00001)**		
20-39	20394/29300	65.1 (64.5, 65.7)
40-49	54166/95844	57.6 (57.1, 58.0)
50-59	53906/148458	33.7 (33.4, 34.1)
60-69	33029/145004	23.3 (23.1, 23.6)
70-75	6625/57767	14.3 (14.0, 14.6)
**Cancer screen detected (p=<0.00001)**		
Screen-detected	30206/135903	36.5 (36.2, 36.8)
Not screen-detetected	55044/141096	41.4 (41.1, 41.6)
Not eligible	82870/199374	29.9 (29.6, 30.2)
**Tumour size (mm) (p=<0.00001)**		
1-20	58195/240040	30.7 (30.5, 30.8)
21-50	69873/134078	40.9 (40.7, 41.1)
>50	9650/15054	40.1 (39.4, 40.8)
Unknown	30402/87201	37.3 (37.0, 37.6)
**Positive nodes (p=<0.00001)**		
0	39982/171886	26.8 (26.6, 27.0)
1 to 3	48605/80497	51.4 (51.1, 51.7)
4 to 9	17089/23031	59.1 (58.5, 59.7)
10 or more	7777/10098	59.9 (59.0, 60.8)
Unknown	54667/190861	31.5 (31.3, 31.7)
**Tumour grade (p=<0.00001)**		
Low	8198/84782	17.1 (16.9, 17.4)
Medium	61816/211845	29.9 (29.7, 30.0)
High	89555/145446	52.3 (52.1, 52.6)
Unknown	8551/34300	31.3 (30.8, 31.7)
**Focality (p=0.00006)**		
Single	28650/82926	34.2 (33.8, 34.5)
Multiple	10413/21527	35.4 (34.9, 35.9)
Unknown	129057/371920	35.5 (35.4, 35.6)
**Breast quadrant (p=<0.00001)**		
Lateral	58192/153462	36.6 (36.4, 36.8)
Central	8888/24199	35.1 (34.6, 35.6)
Medial	18927/57049	35.5 (35.2, 35.8)
Overlapping	10139/26740	35.5 (35.0, 35.9)
Unknown	71974/214923	34.3 (34.1, 34.4)
**Cancer type (p=<0.00001)**		
Carcinoma NST	143679/382077	35.8 (35.6, 35.9)
Lobular	15559/52903	34.0 (33.7, 34.4)
Medullary	1195/2385	36.1 (34.8, 37.5)
Metaplastic	639/1225	40.6 (38.6, 42.7)
Mucinous	929/7789	27.7 (26.7, 28.7)
Papillary	277/1750	26.5 (24.6, 28.5)
Tubular	561/11853	23.3 (22.3, 24.4)
Other/unspecified	5281/16391	34.9 (34.3, 35.5)
**Deprivation index (p=0.18)**		
<20%, least deprived	37691/111780	34.7 (34.5, 34.9)
20-39%	37966/109012	35.7 (35.4, 35.9)
40-59%	34601/98973	35.7 (35.4, 35.9)
60-79%	31304/85877	35.5 (35.2, 35.7)
80+%, most deprived	26558/70731	34.9 (34.6, 35.2)
**Geograhical region (p=<0.00001)**		
Eastern	18054/54322	34.4 (34.0, 34.7)
North West	20017/64383	33.1 (32.8, 33.4)
Northern & Yorkshire	24690/63058	41.8 (41.5, 42.1)
Oxford	9276/29621	29.7 (29.3, 30.2)
South West	24207/73731	33.1 (32.9, 33.4)
Thames	38173/98920	35.1 (34.9, 35.4)
Trent	14889/39555	37.7 (37.3, 38.0)
West Midlands	18814/52783	35.8 (35.4, 36.2)
**Surgery and radiotherapy (p=<0.00001)**		
BCS	86176/297122	33.6 (33.4, 33.7)
Mast+RT	40082/57980	46.8 (46.4, 47.2)
Mast-RT	41862/121271	33.9 (33.7, 34.2)
**Endocrine therapy (p=<0.00001)**		
Not recorded	86647/215231	37.8 (37.6, 37.9)
Recorded or ER-positive	81473/261142	33.1 (33.0, 33.3)
**All women (p=<0.00001)**		
Overall average	168120/476373	35.3 (35.2, 35.4)

Axis: 0 20 40 60 80 100

Figure S15: Recording of the use of chemotherapy to treat the index breast cancer, by patient and tumour characteristics. In the adjusted percent panel all factors are adjusted for each other. The p-values exclude unknown levels and where factors are ordinal it's a test for trend, otherwise it's a test for heterogeneity. (All patients treated with BCS are included in the radiotherapy recorded group and all ER+ve patients are included in the endocrine recorded group.)

Abbreviations: BCS=breast-conserving surgery, ER=oestrogen-receptor, NST=no special type

Type of second cancer		Radiotherapy (or BCS) (recorded vs not)	Endocrine therapy (or ER+ve) (recorded vs not)	Chemotherapy (recorded vs not)
**Second cancer group**	**Number of cancers**	**Unadjusted rate ratio (99% CI)**	**Unadjusted rate ratio (99% CI)**	**Unadjusted rate ratio (99% CI)**
**Gynaecological**				
Cervix	488	0.87 (0.67, 1.13)	1.00 (0.79, 1.26)	0.99 (0.77, 1.27)
Uterine	5793	0.97 (0.90, 1.05)	1.14 (1.07, 1.22)	0.68 (0.62, 0.73)
Ovary or fallopian tubes	3729	1.00 (0.91, 1.10)	0.84 (0.78, 0.92)	0.94 (0.86, 1.03)
Urinary system	2623	0.94 (0.84, 1.06)	1.04 (0.94, 1.15)	0.72 (0.64, 0.80)
**Hematopietic/lymphoid**				
Lymphoma	2180	0.91 (0.81, 1.03)	1.06 (0.95, 1.19)	0.56 (0.49, 0.64)
Acute lymphoblastic leukaemia	63	1.00 (0.48, 2.11)	0.83 (0.43, 1.59)	2.19 (1.14, 4.19)
Acute myeloid leukaemia	651	1.06 (0.83, 1.34)	0.98 (0.80, 1.20)	1.31 (1.06, 1.61)
Other leukaemia	781	0.95 (0.77, 1.17)	1.10 (0.92, 1.33)	0.61 (0.49, 0.76)
Myeloma	823	1.06 (0.86, 1.31)	1.12 (0.93, 1.34)	0.55 (0.44, 0.68)
**Bone and soft tissue**				
Bones and joints	79	1.01 (0.52, 1.96)	0.66 (0.36, 1.18)	1.58 (0.88, 2.84)
Soft tissue	468	1.76 (1.27, 2.43)	0.97 (0.76, 1.23)	1.00 (0.77, 1.28)
**Skin**				
Melanoma of skin	2319	1.05 (0.93, 1.19)	1.01 (0.90, 1.12)	0.96 (0.85, 1.07)
**Head and neck/endocrine**				
Salivary gland	177	0.78 (0.51, 1.18)	0.99 (0.67, 1.46)	1.80 (1.22, 2.65)
Other head and neck	938	1.04 (0.85, 1.26)	0.97 (0.82, 1.14)	1.10 (0.92, 1.31)
Thyroid	545	0.89 (0.70, 1.14)	1.14 (0.91, 1.42)	1.28 (1.02, 1.60)
Other endocrine gland	90	0.94 (0.51, 1.73)	1.04 (0.61, 1.80)	0.95 (0.53, 1.69)
**Upper gastrointestinal**				
Oesophagus	1015	0.91 (0.76, 1.10)	1.08 (0.92, 1.27)	0.69 (0.57, 0.83)
Stomach	992	0.84 (0.70, 1.01)	1.07 (0.91, 1.26)	0.81 (0.67, 0.97)
Pancreas	1993	1.02 (0.89, 1.17)	1.10 (0.98, 1.24)	0.70 (0.62, 0.80)
**Lower or other gastrointestinal**				
Colorectal	6835	0.96 (0.90, 1.03)	1.03 (0.97, 1.10)	0.63 (0.59, 0.68)
Other GI	1276	1.00 (0.85, 1.18)	1.01 (0.88, 1.17)	0.74 (0.63, 0.87)
**Liver**				
Liver	665	0.91 (0.72, 1.13)	1.05 (0.86, 1.29)	0.68 (0.54, 0.86)
**Respiratory**				
Lung	8741	0.96 (0.90, 1.02)	0.99 (0.94, 1.05)	0.65 (0.61, 0.70)
Lung (ipsilateral)	3972	1.03 (0.93, 1.13)	1.04 (0.95, 1.12)	0.67 (0.61, 0.74)
Lung (contralaleral)	3450	0.94 (0.85, 1.04)	1.11 (1.02, 1.22)	0.66 (0.60, 0.73)
Other respiratory and intrathoracic	445	0.98 (0.74, 1.29)	1.02 (0.80, 1.31)	0.63 (0.47, 0.84)
**Central nervous system**				
CNS tumours	1549	0.99 (0.85, 1.15)	0.93 (0.82, 1.06)	0.90 (0.78, 1.03)
**All cancers (excluding breast)**				
Other specified sites	202	0.87 (0.58, 1.30)	0.99 (0.69, 1.42)	0.76 (0.51, 1.14)
All specified sites	45467	0.97 (0.95, 1.00)	1.02 (1.00, 1.05)	0.74 (0.72, 0.76)
Mutliple or unspecified sites	1153	0.74 (0.63, 0.87)	1.03 (0.88, 1.20)	0.80 (0.68, 0.95)
**Breast**				
Contralateral	18127	0.91 (0.87, 0.95)	0.80 (0.77, 0.84)	1.08 (1.04, 1.13)

Axis (each panel): .5, 1, 1.5, 2, 2.5

Figure S16: Unadjusted rate ratios (and 99% confidence intervals) for the recording of the use of treatments for the index breast cancer by type of second cancer incidence. All patients treated with BCS are included in the radiotherapy recorded group and all ER+ve patients are included in the endocrine recorded group. *Boxes in grey and black indicate results with RR>1 and p-value<0.01 (or RR<1 & p-value<0.01 if contralateral after endocrine therapy). If the box is grey the result is not supported by randomised evidence, if black it is supported by randomised evidence. (See Table S2 for a summary of the evidence from randomised trials on adjuvant treatments and second cancer risks.)*
Abbreviations: BCS=breast-conserving surgery, ER=oestrogen-receptor

Type of second cancer		Radiotherapy (or BCS) (recorded vs not)	Endocrine therapy (or ER+ve) (recorded vs not)	Chemotherapy (recorded vs not)
Second cancer group	Numberof cancers	Adjusted rate ratio (99% CI)	Adjusted rate ratio (99% CI)	Adjusted rate ratio (99% CI)
**Gynaecological**				
Cervix	336	1.27 (0.79, 2.04)	0.91 (0.68, 1.22)	0.93 (0.66, 1.31)
Uterine	3640	0.99 (0.84, 1.17)	1.13 (1.04, 1.24)	0.87 (0.78, 0.97)
Ovary or fallopian tubes	2416	0.87 (0.71, 1.06)	0.87 (0.78, 0.97)	1.20 (1.06, 1.36)
Urinary system	1623	0.85 (0.67, 1.10)	1.00 (0.87, 1.14)	1.11 (0.94, 1.30)
**Hematopietic/lymphoid**				
Lymphoma	1299	0.79 (0.59, 1.06)	1.05 (0.90, 1.22)	0.74 (0.61, 0.90)
Acute lymphoblastic leukaemia	53	0.66 (0.16, 2.68)	0.98 (0.47, 2.03)	3.39 (1.52, 7.53)
Acute myeloid leukaemia	468	1.26 (0.85, 1.89)	0.88 (0.69, 1.13)	2.06 (1.56, 2.71)
Other leukaemia	463	0.94 (0.59, 1.50)	1.04 (0.80, 1.34)	0.91 (0.67, 1.25)
Myeloma	491	1.06 (0.67, 1.67)	0.97 (0.76, 1.24)	0.86 (0.63, 1.17)
**Bone and soft tissue**				
Bones and joints	58	0.96 (0.29, 3.25)	0.64 (0.32, 1.31)	1.61 (0.73, 3.55)
Soft tissue	325	1.63 (0.93, 2.83)	0.95 (0.71, 1.29)	1.05 (0.75, 1.49)
**Skin**				
Melanoma of skin	1510	1.17 (0.92, 1.48)	0.94 (0.82, 1.08)	1.05 (0.89, 1.23)
**Head and neck/endocrine**				
Salivary gland	108	0.78 (0.34, 1.78)	1.14 (0.68, 1.92)	1.61 (0.91, 2.83)
Other head and neck	577	0.98 (0.66, 1.44)	0.90 (0.72, 1.13)	1.12 (0.87, 1.44)
Thyroid	357	0.93 (0.58, 1.50)	1.02 (0.76, 1.36)	1.08 (0.79, 1.49)
Other endocrine gland	54	0.39 (0.07, 2.09)	1.03 (0.49, 2.16)	1.31 (0.61, 2.82)
**Upper gastrointestinal**				
Oesophagus	593	1.00 (0.67, 1.49)	1.11 (0.89, 1.39)	1.08 (0.82, 1.43)
Stomach	643	1.26 (0.90, 1.76)	1.05 (0.85, 1.29)	1.39 (1.08, 1.80)
Pancreas	1120	0.88 (0.66, 1.19)	1.09 (0.92, 1.28)	1.04 (0.85, 1.27)
**Lower or other gastrointestinal**				
Colorectal	4186	0.93 (0.79, 1.08)	1.01 (0.93, 1.10)	1.02 (0.92, 1.13)
Other GI	722	0.91 (0.62, 1.33)	0.95 (0.78, 1.17)	1.01 (0.80, 1.28)
**Liver**				
Liver	368	0.84 (0.51, 1.40)	1.04 (0.78, 1.39)	1.00 (0.71, 1.41)
**Respiratory**				
Lung	4971	0.94 (0.82, 1.08)	0.88 (0.82, 0.96)	0.93 (0.84, 1.02)
Lung (ipsilateral)	2076	0.94 (0.76, 1.17)	0.94 (0.83, 1.06)	0.94 (0.82, 1.09)
Lung (contralateral)	2049	0.91 (0.73, 1.13)	0.97 (0.86, 1.10)	0.90 (0.78, 1.05)
Other respiratory and intrathoracic	284	0.99 (0.57, 1.72)	1.15 (0.83, 1.59)	0.83 (0.56, 1.23)
**Central nervous system**				
CNS tumours	982	1.29 (0.96, 1.73)	0.88 (0.74, 1.05)	1.09 (0.89, 1.32)
**All cancers (excluding breast)**				
Other specified sites	123	0.89 (0.38, 2.10)	0.81 (0.50, 1.32)	0.90 (0.50, 1.60)
All specified sites	27776	0.97 (0.91, 1.03)	0.98 (0.95, 1.01)	1.02 (0.98, 1.06)
Mutliple or unspecified sites	654	0.86 (0.60, 1.22)	0.87 (0.71, 1.08)	1.08 (0.84, 1.39)
**Breast**				
Contralateral	10631	1.16 (1.06, 1.26)	0.83 (0.79, 0.87)	1.01 (0.95, 1.07)

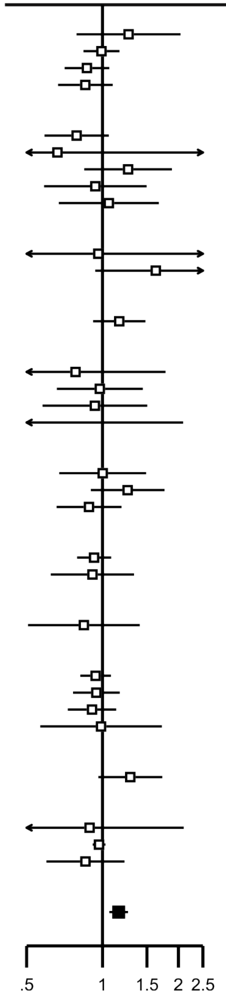

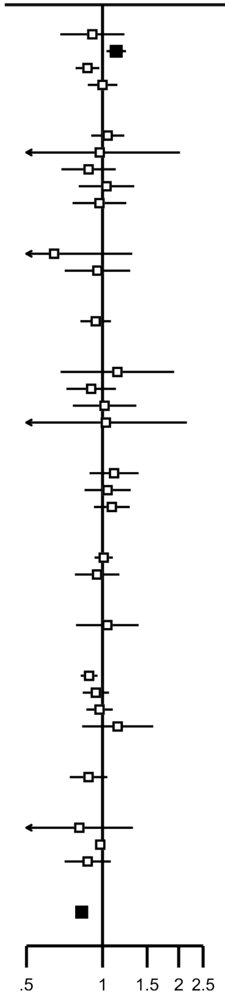

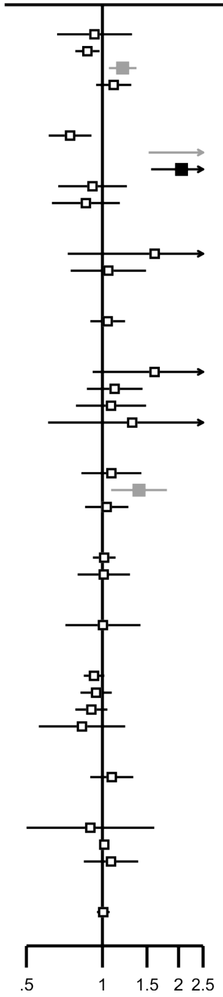

Figure S17: Adjusted rate ratios (and 99% confidence intervals), in the 0-9 year period of follow-up, for the recording of the use of treatments for the index breast cancer by type of second cancer incidence. Rate ratios (RR) are stratified for age at index breast cancer diagnosis (5-year age bands), calendar year of diagnosis, time since diagnosis (5-year intervals), and quintile of deprivation. Each treatment category is adjusted for the other two, and also for surgery (BCS or Mastectomy). All patients treated with BCS are included in the radiotherapy recorded group and all women with ER+ve disease are included in the endocrine recorded group. *Boxes in grey and black indicate results with RR>1 and p-value<0.01 (or RR<1 & p-value<0.01 if contralateral after endocrine therapy). If the box is grey the result is not supported by randomised evidence, if black it is supported by randomised evidence. (See Table S2 for a summary of the evidence from randomised trials on adjuvant treatments and second cancer risks.)*
Abbreviations: BCS=breast-conserving surgery, ER=oestrogen-receptor

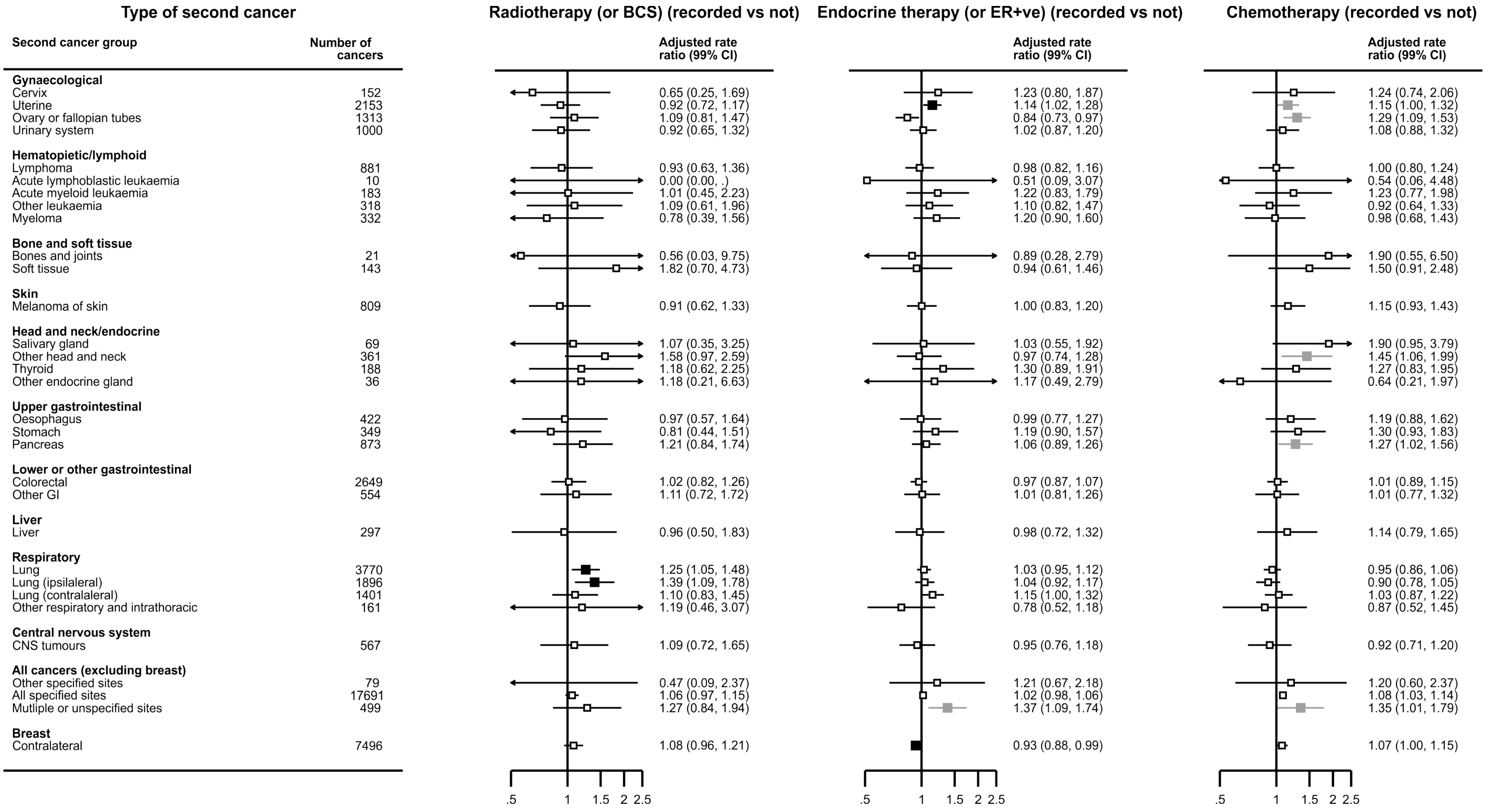

Type of second cancer: Second cancer group	Number of cancers	Radiotherapy (or BCS) (recorded vs not): Adjusted rate ratio (99% CI)	Endocrine therapy (or ER+ve) (recorded vs not): Adjusted rate ratio (99% CI)	Chemotherapy (recorded vs not): Adjusted rate ratio (99% CI)
**Gynaecological**				
Cervix	152	0.65 (0.25, 1.69)	1.23 (0.80, 1.87)	1.24 (0.74, 2.06)
Uterine	2153	0.92 (0.72, 1.17)	1.14 (1.02, 1.28)	1.15 (1.00, 1.32)
Ovary or fallopian tubes	1313	1.09 (0.81, 1.47)	0.84 (0.73, 0.97)	1.29 (1.09, 1.53)
Urinary system	1000	0.92 (0.65, 1.32)	1.02 (0.87, 1.20)	1.08 (0.88, 1.32)
**Hematopietic/lymphoid**				
Lymphoma	881	0.93 (0.63, 1.36)	0.98 (0.82, 1.16)	1.00 (0.80, 1.24)
Acute lymphoblastic leukaemia	10	0.00 (0.00, .)	0.51 (0.09, 3.07)	0.54 (0.06, 4.48)
Acute myeloid leukaemia	183	1.01 (0.45, 2.23)	1.22 (0.83, 1.79)	1.23 (0.77, 1.98)
Other leukaemia	318	1.09 (0.61, 1.96)	1.10 (0.82, 1.47)	0.92 (0.64, 1.33)
Myeloma	332	0.78 (0.39, 1.56)	1.20 (0.90, 1.60)	0.98 (0.68, 1.43)
**Bone and soft tissue**				
Bones and joints	21	0.56 (0.03, 9.75)	0.89 (0.28, 2.79)	1.90 (0.55, 6.50)
Soft tissue	143	1.82 (0.70, 4.73)	0.94 (0.61, 1.46)	1.50 (0.91, 2.48)
**Skin**				
Melanoma of skin	809	0.91 (0.62, 1.33)	1.00 (0.83, 1.20)	1.15 (0.93, 1.43)
**Head and neck/endocrine**				
Salivary gland	69	1.07 (0.35, 3.25)	1.03 (0.55, 1.92)	1.90 (0.95, 3.79)
Other head and neck	361	1.58 (0.97, 2.59)	0.97 (0.74, 1.28)	1.45 (1.06, 1.99)
Thyroid	188	1.18 (0.62, 2.25)	1.30 (0.89, 1.91)	1.27 (0.83, 1.95)
Other endocrine gland	36	1.18 (0.21, 6.63)	1.17 (0.49, 2.79)	0.64 (0.21, 1.97)
**Upper gastrointestinal**				
Oesophagus	422	0.97 (0.57, 1.64)	0.99 (0.77, 1.27)	1.19 (0.88, 1.62)
Stomach	349	0.81 (0.44, 1.51)	1.19 (0.90, 1.57)	1.30 (0.93, 1.83)
Pancreas	873	1.21 (0.84, 1.74)	1.06 (0.89, 1.26)	1.27 (1.02, 1.56)
**Lower or other gastrointestinal**				
Colorectal	2649	1.02 (0.82, 1.26)	0.97 (0.87, 1.07)	1.01 (0.89, 1.15)
Other GI	554	1.11 (0.72, 1.72)	1.01 (0.81, 1.26)	1.01 (0.77, 1.32)
**Liver**				
Liver	297	0.96 (0.50, 1.83)	0.98 (0.72, 1.32)	1.14 (0.79, 1.65)
**Respiratory**				
Lung	3770	1.25 (1.05, 1.48)	1.03 (0.95, 1.12)	0.95 (0.86, 1.06)
Lung (ipsilateral)	1896	1.39 (1.09, 1.78)	1.04 (0.92, 1.17)	0.90 (0.78, 1.05)
Lung (contralateral)	1401	1.10 (0.83, 1.45)	1.15 (1.00, 1.32)	1.03 (0.87, 1.22)
Other respiratory and intrathoracic	161	1.19 (0.46, 3.07)	0.78 (0.52, 1.18)	0.87 (0.52, 1.45)
**Central nervous system**				
CNS tumours	567	1.09 (0.72, 1.65)	0.95 (0.76, 1.18)	0.92 (0.71, 1.20)
**All cancers (excluding breast)**				
Other specified sites	79	0.47 (0.09, 2.37)	1.21 (0.67, 2.18)	1.20 (0.60, 2.37)
All specified sites	17691	1.06 (0.97, 1.15)	1.02 (0.98, 1.06)	1.08 (1.03, 1.14)
Mutliple or unspecified sites	499	1.27 (0.84, 1.94)	1.37 (1.09, 1.74)	1.35 (1.01, 1.79)
**Breast**				
Contralateral	7496	1.08 (0.96, 1.21)	0.93 (0.88, 0.99)	1.07 (1.00, 1.15)

Figure S18: Adjusted rate ratios (and 99% confidence intervals), in the 10-29 year period of follow-up, for the recording of the use of treatments for the index breast cancer by type of second cancer incidence. Rate ratios (RR) are stratified for age at index breast cancer diagnosis (5-year age bands), calendar year of diagnosis, time since diagnosis (5-year intervals), and quintile of deprivation. Each treatment category is adjusted for the other two, and also for surgery (BCS or Mastectomy). All patients treated with BCS are included in the radiotherapy recorded group and all women with ER+ve disease are included in the endocrine recorded group. *Boxes in grey and black indicate results with RR>1 and p-value<0.01 (or RR<1 & p-value<0.01 if contralateral after endocrine therapy). If the box is grey the result is not supported by randomised evidence, if black it is supported by randomised evidence. (See Table S2 for a summary of the evidence from randomised trials on adjuvant treatments and second cancer risks.)*
Abbreviations: BCS=breast-conserving surgery, ER=oestrogen-receptor

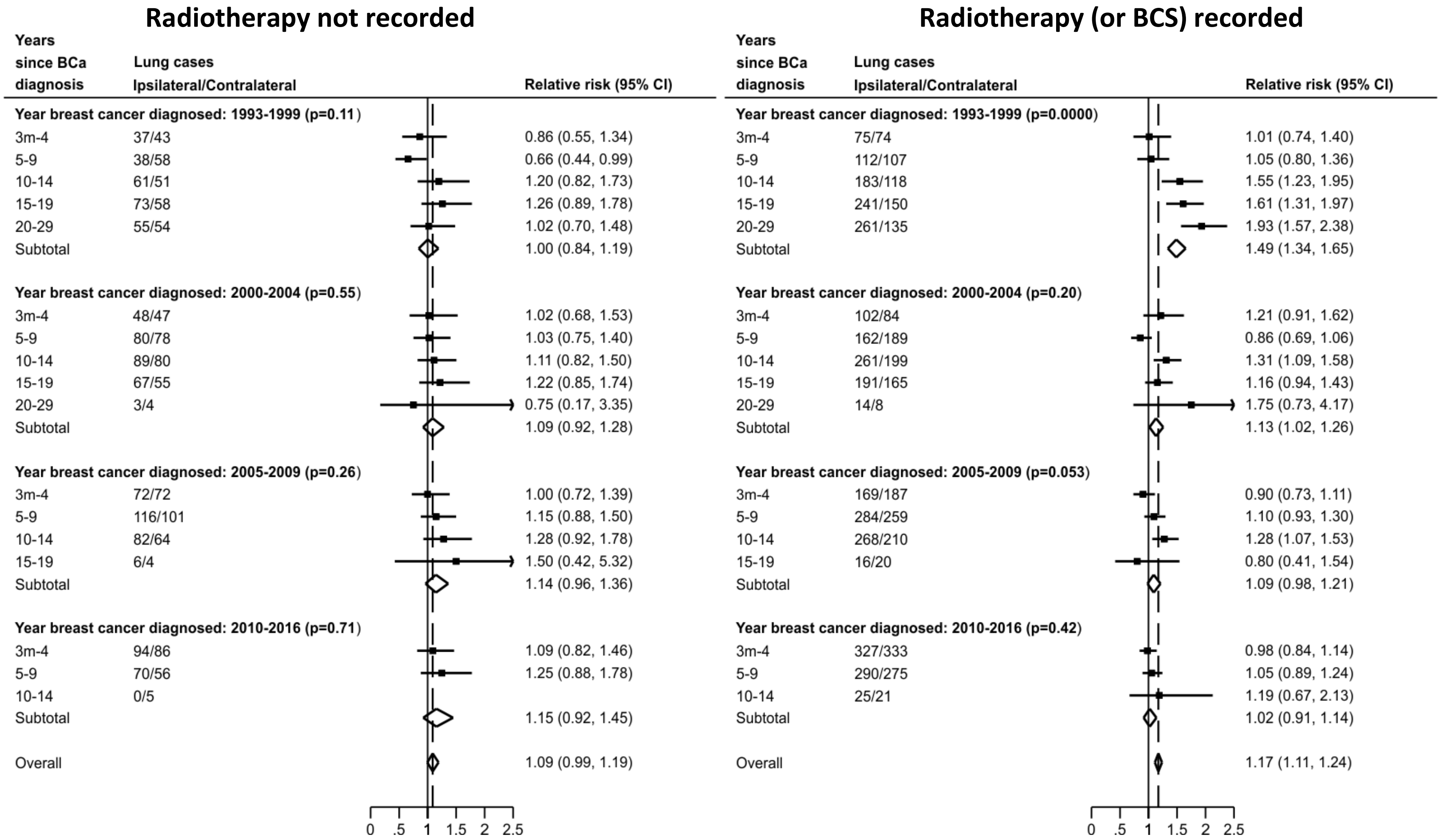

Figure S19: Relative risks (RR) for ipsilateral vs. contralateral lung cancer incidence (ie the same or opposite side to the index breast cancer), by recording of use of radiotherapy, year index breast cancer (BCa) diagnosed, and time since diagnosis. (All patients treated with BCS are included in the radiotherapy recorded group.)

Note: Considering just the period of follow-up of highest risk (10+ years) in women recorded as having radiotherapy/BCS, the (Ipsilateral/Contralateral) counts and RRs (95% CI) by lung cancer histology are; Adenocarcinoma (517/341) 1.52 (1.32,1.74), Non-small cell (100/71) 1.41 (1.04,1.91), Small cell (177/127) 1.39 (1.11,1.75), Squamous cell (239/150) 1.59 (1.30,1.95), Neoplasm (262/198) 1.32 (1.10,1.59), Other histologies (165/139) 1.19 (0.95,1.49) (p for heterogeneity = 0.40)

Abbreviations: BCS=breast-conserving surgery, m=months

ANALYSIS OF NON-INVASIVE CANCERS

Table S8: Standardised incidence ratios (SIR) and absolute excess rates (AER) by type of non-invasive second cancer. Cohort rates are compared to all England rates for the same calendar year, attained age and quintile of deprivation. To calculate the expected risk for contralateral CIS breast cancer, the national incidence rates for CIS breast cancer have been halved.

	As second cancer						At any time after index breast cancer					
**Non-invasive cancer**	**O**	**E**	**SIR**	**95% CI**	**AER**	**95% CI**	**O**	**E**	**SIR**	**95% CI**	**AER**	**95% CI**
CIS of cervix uteri	958	1042	0.92	(0.86, 0.98)	-0.16	(-0.28, -0.04)	969	1052	0.92	(0.87, 0.98)	-0.16	(-0.26, -0.04)
Myelodysplastic syndrome etc	892	873	1.02	(0.96, 1.09)	0.04	(-0.07, 0.15)	911	885	1.03	(0.96, 1.10)	0.05	(-0.07, 0.17)
CIS of digestive organs	684	635	1.08	(1.00, 1.16)	0.09	(0.00, 0.19)	704	645	1.09	(1.01, 1.17)	0.11	(0.01, 0.21)
CIS of respiratory system	50	40	1.26	(0.96, 1.66)	0.02	(-0.00, 0.05)	50	40	1.24	(0.94, 1.64)	0.02	(-0.00, 0.05)
Melanoma in situ	978	933	1.05	(0.98, 1.12)	0.09	(-0.04, 0.21)	1008	947	1.06	(1.00, 1.13)	0.12	(0.00, 0.23)
CIS of other and unspecified genital organs	344	284	1.21	(1.09, 1.35)	0.11	(0.05, 0.19)	363	288	1.26	(1.14, 1.40)	0.14	(0.08, 0.22)
CIS of other and unspecified sites	715	663	1.08	(1.00, 1.16)	0.10	(0.00, 0.20)	766	674	1.14	(1.06, 1.22)	0.17	(0.08, 0.28)
CIS of pituitary gland and craniopharyngeal duct	119	110	1.08	(0.90, 1.29)	0.02	(-0.02, 0.06)	121	112	1.08	(0.91, 1.29)	0.02	(-0.02, 0.06)
CIS of pineal gland	1	0					1	0				
UUB of oral cavity and digestive organs	203	214	0.95	(0.83, 1.09)	-0.02	(-0.07, 0.04)	208	217	0.96	(0.84, 1.10)	-0.02	(-0.07, 0.04)
UUB of middle ear and respiratory and intrathoracic organs	33	28	1.19	(0.84, 1.67)	0.01	(-0.01, 0.04)	34	28	1.20	(0.86, 1.69)	0.01	(-0.01, 0.04)
UUB of female genital organs	90	80	1.12	(0.91, 1.38)	0.02	(-0.01, 0.06)	92	81	1.13	(0.92, 1.39)	0.02	(-0.01, 0.06)
UUB of urinary organs	376	341	1.10	(1.00, 1.22)	0.07	(0.00, 0.14)	386	345	1.12	(1.01, 1.24)	0.08	(0.01, 0.16)
UUB of endocrine glands	42	40	1.05	(0.77, 1.42)	0.00	(-0.02, 0.03)	44	41	1.08	(0.81, 1.46)	0.01	(-0.01, 0.04)
Polycythemia vera	100	136	0.73	(0.60, 0.89)	-0.07	(-0.10, -0.03)	106	138	0.77	(0.63, 0.93)	-0.06	(-0.10, -0.02)
UUB of other and unspecified sites	129	129	1.00	(0.84, 1.19)	0.00	(-0.04, 0.05)	131	130	1.01	(0.85, 1.19)	0.00	(-0.04, 0.05)
CIS of breast	3485	2375	1.47	(1.42, 1.52)	2.12	(1.90, 2.36)	3548	2405	1.48	(1.43, 1.52)	2.16	(1.95, 2.36)
All non-malignant registered tumours excluding CNS	*9199*	*7922*	*1.16*	*(1.14, 1.19)*	*2.44*	*(2.12, 2.87)*	*9442*	*8026*	*1.18*	*(1.15, 1.20)*	*2.67*	*(2.27, 3.03)*
Benign cancers not fullly covered in the cancer registries	17	23	0.73	(0.45, 1.17)	-0.01	(-0.02, 0.01)	18	24	0.76	(0.48, 1.21)	-0.01	(-0.02, 0.01)
CIS of breast (contralateral)	3338	1188	2.81	(2.72, 2.91)	4.10	(3.90, 4.33)						

Note:
There were 147 ipsilateral and 3296 contralateral CIS breast cancers. The remaining 42 had laterality unknown and were grouped with the contralateral cancers totalling 3338. Of the 3338; 358 (10.7%) were lobular, 2807 (84.1%) intraductal, 151 (4.5%) other, and 22 (0.7%) unspecified.

Abbreviations: CIS=Carcinoma in situ, UUB=Neoplasm of uncertain or unknown behaviour

Non-invasive non-breast cancer

Characteristic	Number of cancers /women	Rate ratio (95% CI)
**Year breast cancer diagnosed (p=<0.00001)**		
1993-1999	1494/101825	0.81 (0.75, 0.89)
2000-2004	1470/101603	0.90 (0.84, 0.98)
2005-2009	1425/112424	1.00 (1.00, 1.00)
2010-2016	1325/157709	1.08 (0.99, 1.17)
**Age at breast cancer diagnosis (years) (p=<0.00001)**		
20-39	418/29187	0.88 (0.76, 1.01)
40-49	1112/95234	0.71 (0.63, 0.80)
50-59	1699/147534	0.76 (0.71, 0.82)
60-69	1834/144167	1.00 (1.00, 1.00)
70-75	651/57439	1.19 (1.05, 1.34)
**Cancer screen detected (p=0.10)**		
Screen detected	1609/135083	1.00 (1.00, 1.00)
Not screen detetected	1683/140198	1.06 (0.99, 1.14)
Not eligible	2422/198280	1.14 (1.01, 1.29)
**Tumour size (p=0.003)**		
1-20 mm	3139/238528	1.00 (1.00, 1.00)
21-50 mm	1381/133293	0.89 (0.83, 0.95)
>50 mm	141/14947	0.96 (0.81, 1.14)
Unknown	1053/86793	0.95 (0.87, 1.03)
**Positive nodes (p=0.83)**		
0	2005/170669	1.00 (1.00, 1.00)
1 to 3	918/79962	1.04 (0.96, 1.13)
4 to 9	212/22888	0.98 (0.85, 1.13)
10 or more	76/10023	1.00 (0.79, 1.27)
Unknown	2503/190019	1.01 (0.93, 1.09)
**Tumour grade (p=0.49)**		
Low grade	1103/84242	1.00 (1.00, 1.00)
Medium grade	2586/210392	1.04 (0.96, 1.12)
High grade	1599/144816	1.03 (0.95, 1.13)
Unknown	426/34111	0.91 (0.81, 1.03)
**Focality (p=0.76)**		
Single	1099/82520	1.00 (1.00, 1.00)
Multiple	235/21262	0.98 (0.84, 1.14)
Unknown	4380/369779	0.90 (0.81, 1.00)
**Breast quadrant (p=0.62)**		
Lateral	1819/152595	1.00 (1.00, 1.00)
Central	313/24047	1.06 (0.94, 1.20)
Medial	663/56769	1.01 (0.92, 1.10)
Overlapping	301/26600	0.95 (0.84, 1.08)
Unknown	2618/213550	0.99 (0.93, 1.06)
**Cancer type (p=0.37)**		
Carcinoma NST	4522/380185	1.00 (1.00, 1.00)
Lobular	671/52327	1.08 (0.99, 1.17)
Medullary	40/2373	1.16 (0.85, 1.59)
Metaplastic	10/1210	0.91 (0.49, 1.70)
Mucinous	83/7711	0.85 (0.68, 1.06)
Papillary	21/1726	0.96 (0.63, 1.48)
Tubular	161/11717	0.99 (0.84, 1.17)
Other/unspecified	206/16312	1.02 (0.89, 1.18)
**ER status and endocrine use (p=0.77)**		
ER-positive (or endocrine)	2990/259636	1.00 (1.00, 1.00)
ER-negative	202/22091	0.98 (0.84, 1.13)
Unknown	2522/191834	1.02 (0.95, 1.08)
**Deprivation index (p=0.003)**		
<20%, least deprived	1295/111072	1.00 (1.00, 1.00)
20-39%	1386/108327	1.11 (1.03, 1.20)
40-59%	1168/98340	1.05 (0.97, 1.13)
60-79%	1044/85395	1.13 (1.04, 1.23)
80+%, most deprived	821/70427	1.15 (1.05, 1.26)
**Time since breast cancer diagnosis (years) (p=<0.00001)**		
3m-4	2039/2093102	0.75 (0.69, 0.81)
5-9	1737/1627524	0.83 (0.77, 0.90)
10-14	1090/923307	1.00 (1.00, 1.00)
15-19	604/430554	1.31 (1.19, 1.45)
20-24	220/145961	1.54 (1.32, 1.79)
25-29	24/18843	1.36 (0.90, 2.04)

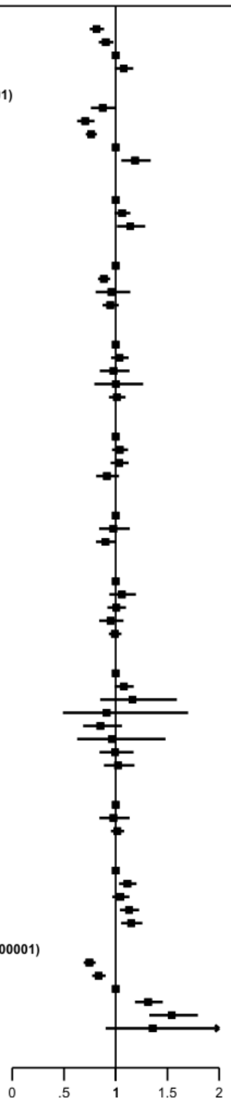

Contralateral CIS breast cancer

Characteristic	Number of cancers /women	Rate ratio (95% CI)
**Year breast cancer diagnosed (p=<0.00001)**		
1993-1999	793/101825	0.85 (0.76, 0.95)
2000-2004	912/101603	1.03 (0.93, 1.14)
2005-2009	821/112424	1.00 (1.00, 1.00)
2010-2016	812/157709	1.17 (1.05, 1.30)
**Age at breast cancer diagnosis (years) (p=<0.00001)**		
20-39	248/29187	1.55 (1.25, 1.92)
40-49	836/95234	1.57 (1.31, 1.89)
50-59	1269/147534	1.28 (1.16, 1.40)
60-69	801/144167	1.00 (1.00, 1.00)
70-75	184/57439	0.91 (0.74, 1.11)
**Cancer screen detected (p=0.06)**		
Screen detected	918/135083	1.00 (1.00, 1.00)
Not screen detetected	1069/140198	1.10 (1.00, 1.20)
Not eligible	1351/198280	0.86 (0.71, 1.05)
**Tumour size (p=0.01)**		
1-20 mm	1689/238528	1.00 (1.00, 1.00)
21-50 mm	907/133293	1.07 (0.99, 1.17)
>50 mm	105/14947	1.27 (1.04, 1.56)
Unknown	637/86793	1.08 (0.97, 1.20)
**Positive nodes (p=0.58)**		
0	1148/170669	1.00 (1.00, 1.00)
1 to 3	570/79962	1.00 (0.90, 1.11)
4 to 9	143/22888	0.97 (0.81, 1.17)
10 or more	46/10023	0.90 (0.66, 1.21)
Unknown	1431/190019	0.89 (0.80, 0.99)
**Tumour grade (p=0.55)**		
Low grade	633/84242	1.00 (1.00, 1.00)
Medium grade	1458/210392	0.95 (0.87, 1.05)
High grade	990/144816	0.96 (0.86, 1.07)
Unknown	257/34111	0.94 (0.81, 1.10)
**Focality (p=0.06)**		
Single	573/82520	1.00 (1.00, 1.00)
Multiple	168/21262	1.20 (0.99, 1.44)
Unknown	2597/369779	0.93 (0.81, 1.08)
**Breast quadrant (p=0.35)**		
Lateral	1004/152595	1.00 (1.00, 1.00)
Central	148/24047	0.95 (0.80, 1.13)
Medial	390/56769	1.10 (0.98, 1.24)
Overlapping	176/26600	1.01 (0.86, 1.19)
Unknown	1620/213550	1.12 (1.04, 1.22)
**Cancer type (p=0.04)**		
Carcinoma NST	2688/380185	1.00 (1.00, 1.00)
Lobular	394/52327	1.09 (0.97, 1.22)
Medullary	20/2373	0.92 (0.59, 1.43)
Metaplastic	6/1210	0.82 (0.37, 1.84)
Mucinous	44/7711	0.86 (0.64, 1.16)
Papillary	14/1726	1.25 (0.74, 2.12)
Tubular	70/11717	0.70 (0.55, 0.90)
Other/unspecified	102/16312	0.84 (0.68, 1.03)
**ER status and endocrine use (p=0.002)**		
ER-positive (or endocrine)	1668/259636	1.00 (1.00, 1.00)
ER-negative	155/22091	1.33 (1.11, 1.58)
Unknown	1515/191834	1.08 (0.99, 1.17)
**Deprivation index (p=0.84)**		
<20%, least deprived	801/111072	1.00 (1.00, 1.00)
20-39%	784/108327	1.03 (0.94, 1.14)
40-59%	714/98340	1.05 (0.95, 1.17)
60-79%	601/85395	1.05 (0.94, 1.17)
80+%, most deprived	438/70427	0.96 (0.85, 1.08)
**Time since breast cancer diagnosis (years) (p=<0.00001)**		
3m-4	1199/2093102	0.70 (0.64, 0.77)
5-9	1010/1627524	0.77 (0.70, 0.85)
10-14	715/923307	1.00 (1.00, 1.00)
15-19	315/430554	0.96 (0.84, 1.10)
20-24	87/145961	0.84 (0.66, 1.05)
25-29	12/18843	0.90 (0.51, 1.60)

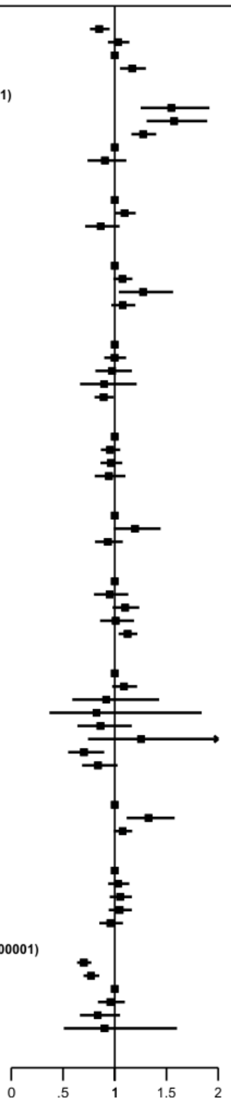

Figure S20: Adjusted rate ratios for non-invasive non-breast cancer and contralateral CIS breast cancer by patient factors and characteristics of the index invasive breast cancer. All factors are adjusted for each other as well as geographical region. The p-values exclude unknown levels and where factors are ordinal they are tests for trend, otherwise they are tests for heterogeneity. (All ER+ve patients are included in the endocrine recorded group.)
Abbreviations: ER=oestrogen-receptor, NST=no special type.

COMPARABLE STUDIES

Table S9: Other studies and reviews of second cancer after breast cancer

Search based on Embase, PubMed, Google Scholar and Medline (excluding preprints) - 2014 to 12.6.2024

Summary of keyword, title and abstract search description: - (second cancer OR new primary cancer OR subsequent cancer OR non-breast cancer OR treatment related) AND breast cancer NOT (recurrence OR recurrent OR metastatic OR secondary)

Detailed search string: PubMed Database: Embase <1974 to 2024 June 12>, Ovid MEDLINE(R) ALL <1946 to 2024 June 12,

**1** (((second cancer or new primary cancer or subsequent cancer or non-breast cancer or treatment related) and breast cancer) not (recurrence or recurrent or metastatic or secondary)).mp. [mp=tx, bt, ti, ab, hw, tn, ot, dm, mf, dv, kf, fx, dq, nm, ox, px, rx, an, ui, ds, on, sy, ux, mx] (6399)
**2** limit to English language (6213)
**3** limit to full text (967)
**4** limit to yr="2013 -Current" (614)
**5** limit to "remove preprint records" (614)
**6** limit to English language (614)
**7** limit to full text (614)
**8** limit to human/s (614)
**9** remove preprint records (560)
**10** remove duplicates (433)
**11** keep 13,27-28,41-42,45-46,63,66,69,104,124,128-129,130,151,176,184-186,193,206,263,274,283,296,329,333,373,398,418 (31)

**Original Studies (23)**	
**Publication**	**Summary of findings**
Chen T, Fallah M, Jansen L, et al Distribution and risk of the second discordant primary cancers combined after a specific first primary cancer in German and Swedish cancer registries. Cancer Letters, 01 Dec 2015 369(1):152-166	Among 1,537,004 survivors of <u>any</u> first primary cancer of any type in Germany and 588,103 in Sweden second discordant cancers were recorded. Standardised incidence ratios (SIRS) for all/any second discordant primary cancers after breast cancer were elevated. Specific discordant cancer types were not provided.
Hung M.-H., Liu C.-J., Teng C.-J., et al. Risk of second non-breast primary cancer in male and female breast cancer patients: A population-based cohort study. PLoS ONE February 2016 11(2).	Standardised incidence ratios (SIRS) for new primary cancers were higher in 578 men (2.17) than 100915 women (1.51) diagnosed with early breast cancer. Risk were higher in patients aged <40 (3.39).<br>In women the risks of uterine, thyroid and bone/soft tissue cancers were increased. In men thyroid, skin, and head/neck cancer risks were higher. In all patients male gender, older age, chemotherapy treatment and liver cirrhosis were associated with higher risk of new cancers.
Radivoyevitch T, Sachs RK, Gale RP et al. Defining AML and MDS second cancer risk dynamics after diagnoses of first cancers treated or not with radiation. Leukemia. 2016 Feb;30(2):285-94.	Patients with breast cancer had raised risks of subsequent AML and MDS which were seen to a greater extent in patients who had radiotherapy. There was a marked excess risk of acute promyelocytic leukemia following radiation therapy.
Silverman B.G., Lipshitz I., Keinan-Boker L. Second primary cancers after primary breast cancer diagnosis in Israeli women, 1992 to 2006. Journal	Standardised incidence ratios (SIRS) for second primary cancer were reported in 46090 Israeli women with a diagnosis of breast cancer from 1990-2006. The overall SIR was 1.26 for non-breast primary cancers. Significantly increased risks of colorectal, uterine, lung, ovarian, thyroid

of Global Oncology. 1 Apr 2017 3(2):135-142	cancer and leukaemia were seen. Young age at diagnosis of index cancer increased risk of second primary cancers.
Xie L., Lin C., Zhang H., et al. Second malignancy in young early-stage breast cancer patients with modern radiotherapy. Medicine, 01 Apr 2018 97(17).	There was a focus on young patients (20-44). Of 22628 patients, aged 20-44, with breast cancer diagnosed 1988-2009, 1495 developed a second primary cancer at median follow-up of 11.8 years (3.7% contralateral breast cancer, 2.9% non-breast second malignancies, and 0.7% high-dose site second malignancies) Survival following second malignancy was lower in patients who had received radiotherapy for their index breast cancer.
Park J.S., Lee Y.J., Lee S.-T., et al. Second cancers among Korean breast cancer patients with BRCA1/2 mutations. Breast April 1 2018 44 (Supplement 1):S56-S57	The incidence of second cancer was compared between 114 Korean breast cancer survivors with BRCA1/2 mutations and 661 patients without mutations. There was no significant difference in the incidence of second cancers between BRCA1/2 mutation carriers and non-carriers. In comparing risks of second cancers in breast cancer survivors and known mutations vs pts with strong family history and no mutations, there were no differences.
Liu J., Hu Z., Feng Y., et al. Problems to affect long-term survival for breast cancer patients An observational study of subsequent lung/bronchus malignancies. Medicine Sep 2018 97(39).	Of a cohort of 535941 patients with early breast cancer in the SEER database diagnosed 1973-2014, 73394 (13%) developed subsequent primary cancer (median time to new cancer 72 months) and 9398 developed lung cancer.
Zheng G, Hemminki A, Försti A et al, Second primary cancer after female breast cancer: Familial risks and cause of death. Cancer Med. 2019 Jan;8(1):400-407.	87752 female BC patients were followed for second primary cancer diagnoses and death. Relative risks (RRs) in patients who had parents or siblings affected by the same cancer were compared to the patients without family history.<br>After a median follow-up of 5 years, 14952 BC patients developed second primary cancers - 68.8% had parents or siblings diagnosed with cancer. Familial risks were significant for 14 site-specific second primary cancers - the highest was ovarian cancer (RR = 6.28). Second primary cancer was the main cause of death, from non-breast cancers and breast cancer in approximately equal proportions.
Grassmann F, He W, Eriksson et al. Interval breast cancer is associated with other types of tumors. Nat Commun. 2019 Oct 22;10(1):4648.	Patients with interval breast cancer (1772) were more likely to develop a non-BC tumour (OR 1.43) before or after (OR 1.28) breast cancer diagnosis, and more likely to report a family history of non-BC tumors. In conclusion, interval breast cancer is associated with other tumors and common cancer variants are unlikely to be responsible for this association. These findings could have implications for future screening and prevention programs.
Li S., Shen Y., Zhao X., et al. Clinicopathological features, survival and risk in breast cancer survivors with thyroid cancer: an analysis of the SEER database. BMC public health 29 Nov 2019 19(1):1592.	The risk of developing thyroid cancer after breast cancer was higher than in the general population (SIR 1.22, 95% CI [1.14, 1.31]), especially within 3 years of breast cancer diagnosis. Black race, grade and ER/PR positive expression for associated with increased risk of thyroid cancer. Other cancer types were not considered.
Journy N, Schonfeld SJ, Hauptmann M, et al. Dose-volume effects of breast cancer radiation therapy on the risk of second oesophageal cancer. Radiother Oncol. 2020 Oct;151:33-39.	In a case-control study nested within a cohort of 289,748 ≥5-year survivors of female breast cancer treated in 1943–2003, doses to the second primary cancer (DSPC) and individual dose-volume histograms (DVH) to the entire oesophagus were reconstructed for 252 oesophageal cancer cases and 488 matched controls (median follow-up time: 13 (5–37 years) to investigate the relationship between oesophagus dose-volume distribution and oesophageal cancer risk. Increased risk correlated best with median dose and V30.
Yao K.A.K., Clifford J., Li S., et al. Prevalence of germline pathogenic and likely pathogenic variants in patients	Of 75550 women with primary breast cancer 7728 (controls) developed a second primary breast cancer at a median time of 11 years. Restricting to women tested for actionable genes (n 1/4 60 310), there were 4231 (7.8%)

with second breast cancers. JNCI Cancer Spectrum. 2020 4(6).	carriers of P/LP variants in actionable genes among the controls compared with 652 (11.1%) women with second breast cancer (P < .001). This difference was seen in Caucasian, African Americana and Hispanic women. Women with P/LP variants in breast cancer predisposition genes are more likely to have second breast cancer than non-carriers suggesting genetic mechanisms in some cases of second primary and/or contralateral breast cancer.
Feigelson HS, Bodelon C, Powers et al Body Mass Index and Risk of Second Cancer Among Women With Breast Cancer. J Natl Cancer Inst. 2021 Sep 4;113(9):1156-1160	822 (12.7%) of women developed a second cancer from a cohort of 6481 breast cancer survivors within Kaiser Permanente (Colorado and Washington) at a mean follow-up was 88.0 months. BMI at the first cancer was extracted from the medical record. The mean age at initial breast cancer diagnosis was 61.2 (SD ¼ 11.8) years. Most cases were overweight (33.4%) or obese (33.8%) and diagnosed at stage I (62.0%), The risk of any second cancer diagnosis increased by 7% (RR ¼ 1.07, 95% CI ¼ 1.01 to 1.14); 13% (RR ¼ 1.13, 95% CI ¼ 1.05 to 1.21) for obesity related cancers, 11% (RR ¼ 1.11, 95% CI ¼ 1.02 to 1.21) for a second breast cancer, and 15% (RR ¼ 1.15, 95% CI ¼ 1.04 to 1.27) for a second oestrogen receptor–positive breast cancer.
Kang D, Yoon SE, Shin D et al. Risk of non-Hodgkin lymphoma in breast cancer survivors: a nationwide cohort study. Blood Cancer J. 2021 Dec 14;11 (12):200.	In a retrospective cohort study in South Korea, 84,969 women with breast cancer diagnosed between January 2002-2016 and a 1:10 sample of age-matched non-breast cancer controls (N = 1,057,674). were considered. During follow-up, 1564 incident cases of NHL occurred. The adjusted Hazard Ratio (HR) for NHL associated with breast cancer was 1.64. The adjusted HR for NHL was much higher in women aged <50 years at breast cancer diagnosis and who received endocrine therapy. Breast cancer was associated with a significantly increased risk of NHL, particularly follicular lymphoma and mature T/NK-cell lymphoma.
Chen F, Park SL, Wilkens LR, et al Genetic Risk of Second Primary Cancer in Breast Cancer Survivors: The Multiethnic Cohort Study. Cancer Res. 2022 Sep16; 82(18):3201-3208.	The Multiethnic Cohort (MEC) Study, was conducted in 3,223 female breast cancer survivors from five racial/ethnic populations (White, African American, Japanese American, Latino, and Native Hawaiian) to assess the association of rare pathogenic variants in 37 known cancer predisposition genes with risk of second primary cancer. A total of 719 (22.3%) women developed a second primary cancer , of which, 323 (10.0%) were SPBC. Germline PVs in BRCA1 (HR, 2.28; 95% CI, 1.11–4.65) and ERCC2 (HR, 3.51; 95% CI, 1.29–9.54) were significantly enriched in women with SPC. In the subtype analysis for SPBC, a significant association of ERCC2 PVs (HR, 5.09; 95% CI, 1.58–16.4) and a suggestive association of BRCA2 PVs (HR, 2.24; 95% CI, 0.91–5.55) were observed. There was also a higher risk of SPNBC in carriers of BRCA1 PVs (HR, 2.98; 95% CI, 1.21–7.36). These results provide evidence that germline PVs in BRCA1, BRCA2, and ERCC2 contribute to the development of SPC in breast cancer survivors.
Veiga L.H.S., Vo J.B., Curtis R.E., et al. Treatment-related thoracic soft tissue sarcomas in US breast cancer survivors: a retrospective cohort study. The Lancet Oncology. November 2022 23(11):1451-1464	19 (0.1%) of 15 940 eligible women in a cohort from Kaiser Permanente (1990-2016) developed thoracic soft tissue sarcomas following radiotherapy (RR 8.1). The RR for angiosarcoma was 3.6 following anthracyclines.<br>Alkylating agents were associated with other sarcomas (RR of 7.7) and thoracic sarcomas (RR 3.0) whereas radiotherapy was associated with an increased risk (RR 3.0). Radiotherapy was the strongest risk factor for sarcoma in both cohorts. Diabetes and hypertension were also associated with increased risk of sarcoma.
Deng Z, Jones MR, Wang MC, et al, Racial and ethnic disparities in mortality among breast cancer survivors after a second malignancy. J Natl Cancer Inst. 2023 Mar 9;115(3):279-287.	Mortality according to racial types and ethnicity were assessed in a cohort of 39029 female breast cancer survivors following development of a second primary cancer between 2000 and 2014 in the SEER18 database. Higher cancer mortality was seen in non-Hispanic black, or Hispanic compared to white women.

Ramin S., Liang H., Liang P et al. Predilection site and risk factor of second primary cancer: A pan-cancer analysis based on the SEER database. Chinese Medical Journal. Jun 2023 136(12):1500-1502	A large multi cancer SEER study estimated SIRs for new primary cancer following a diagnosis of breast cancer. Elevated SIRs were reported for many new primary cancers. The highest SIR (>10) seen for breast and female genital organ cancers.
Ramin C., Veiga L.H.S., Vo J.B., et al. Risk of second primary cancer among women in the Kaiser Permanente Breast Cancer Survivors Cohort. Breast Cancer Research, December 2023 25(1)	Within a cohort of 16004 patients with early breast cancer, diagnosed 1990 – 2016, the overall SIR for new primary cancer was 1.7. Significantly higher SIRs were reported for peritoneal, soft tissue, contralateral breast, acute myeloid leukaemia (or myelodysplastic syndrome) oral, colon, pancreas, lung, and uterine cancer, melanoma and non-Hodgkin lymphoma. Radiotherapy was associated with increased SIRs for all second cancers combined and soft tissue sarcoma. Chemotherapy was associated with a decreased risk for all second cancers combined but an increased risk of myelodysplastic syndrome, Endocrine therapy was associated with lower contralateral breast cancer risk.
Avatefi M., HadavandSiri F., Nazari S.S.H., et al. Risk factors of developing contralateral breast cancer after first primary breast cancer treatment. Cancer Reports. January 2024 7(1)	This was a retrospective cohort study in Iran. 5003 patients were identified with early breast cancer between 2000 and 2020. 145 patients developed contralateral breast cancer. The median time between index and subsequent contralateral breast cancer was 3.92 years. Age over 60 was negatively associated with contralateral breast cancer.
Allen I, Hassan H, Joko-Fru WY et al. Risks of second primary cancers among 584,965 female and male breast cancer survivors in England: a 25-year retrospective cohort study. Lancet Regional Health - Europe. May 2024 40 (1-14)	581,403 female and 3562 male BC survivors diagnosed between 1995 and 2019 in England were included. Second primary cancers (SPCs) were reported using standardised incidence rations (SIRs) Both genders were at elevated contralateral breast (SIR: 2.02 (95% CI: 1.99-2.06) females; 55.4 (35.5-82.4) males) and non-breast (1.10 (1.09-1.11) females, 1.10 (1.00-1.20) males) SPC risks. Non-breast SPC risks were higher for women younger at BC diagnosis (SIR: 1.34 (1.31-1.38) <50 y, 1.07 (1.06-1.09) >=50 y) and more deprived (SIR: 1.00 (0.98-1.02) vs 1.34 (1.30-1.37).<br>Enhanced SPC surveillance may benefit BC survivors. The associations between deprivation and SPC risks could provide clinical management insights.
Zhang BX, Brantley KD, Rosenberg SM et al. Second primary non-breast cancers in young breast cancer survivors. Breast Cancer Research & Treatment. June 2024, 1-11.	A study included 1230 participants of the Young Women's BC Study (YWS) diagnosed 2006 and 2016 at age 40 or younger at diagnosis (N = 1,230). Second primary non-breast cancers (SPNBCs) were estimated.<br>47 patients (4%) developed an SPNBC. Five and 10-year cumulative incidence were 1.4% and 3.2%. No patient factors, primary tumour characteristics, or treatments were associated with SPNBC.<br>This highlights the importance of long-term surveillance for new non-breast cancers in young adult BC survivors.
Brantley KD, Rosenberg SM, Collins LC, et al. Second primary breast cancer in young breast cancer survivors. JAMA oncology. 2024 Apr 11.	Among 685 breast cancer survivors aged 40 years or younger at primary BC diagnosis who underwent lumpectomy or unilateral mastectomy, the 10-year risk of developing a second primary BC was approximately 2% among women who did not carry a germline pathogenic variant compared with approximately 9% among women who carried such a variant, suggesting that young BC survivors without a germline pathogenic variant have a low risk of developing a second primary breast cancer in the first 10 years after diagnosis

**Reviews (8)**	
**Publication**	**Summary of findings**
Marcu L.G., Santos A., Bezak E. Risk of second primary cancer after breast	Common sites with increased second primary cancer occurrence were identified (such as lung, oesophagus, myeloid leukaemia, and soft tissue).

cancer treatment. European Journal of Cancer Care. Jan 2014 23(1):51-64.	However, for several other anatomical locations results were inconclusive. Given the large number of genetic, environmental and life-style factors, it would be erroneous to assume that radiotherapy is solely responsible for the incidence of second primary cancer. Thus the excess risk in ovarian cancers could be due to the same predisposing genes as the primary breast cancer, the excess risk in uterine cancer can be attributed to shared hormonal risk factors, whereas the excess in blood/lymphatic cancers could be due to chemotherapy-related toxicities.
Molina-Montes E., Perez-Nevot B., Pollan M et al. Cumulative risk of second primary contralateral breast cancer in BRCA1/BRCA2 mutation carriers with a first breast cancer: A systematic review and meta-analysis. Breast, Dec 2014, 23(6):721-742.	Twenty articles were retrieved. The cumulative 5-years risk of contralateral breast cancers in BRCA1 & 2 mutation carriers was 15% and 9% respectively at 10-years the risks were 27% and 19%, respectively. The risk was lower in non-BRCA carriers (3%) and remained so over subsequent years. In conclusion, risk of contralateral breast cancers increases with length of time after the first breast cancer diagnosis in BRCA1/2 mutation carriers. Studies addressing the impact of treatment-related factors and clinical characteristics of the first breast cancer on this risk are warranted.
Molina-Montes E., Requena M., Sanchez-Cantalejo E. et al Risk of second cancers cancer after a first primary breast cancer: a systematic review and meta-analysis. Gynecologic oncology, 1 Jan 2015 136(1):158-171.	15 articles were identified reporting retrospective cohort studies. SIRs reported in these studies for all cancers combined varied from 1.0 to 1.4. The pooled SIR estimate for second cancer risk was 1.17. By age groups, SIR estimates were 1.51 for women younger than 50 years and 1.11) for older women. Women with breast cancer are at risk of second cancers within the first 10 years after the first breast cancer diagnosis
Grantzau T, Overgaard J. Risk of second non-breast cancer among patients treated with and without postoperative radiotherapy for primary breast cancer: A systematic review and meta-analysis of population-based studies including 522,739 patients. Radiother Oncol. 2016 Dec;121(3):402-413.<br>.	Of 22 cohort studies comprising 245,575 irradiated and 277,164 non-irradiated women comparing their risk of second primary cancer to the risk of the general female population Irradiated patients had an overall increased risk of second non-breast cancer, with a SIR of 1.23 (95% confidence interval [CI] 1.12–1.36). For non-irradiated patients the SIR was 1.08 (95% CI, 1.03–1.13). For irradiated patients the incidence of second cancers included the lung, esophagus, thyroid and connective tissues progressively increased over time, peaking at 10–15 years following breast cancer diagnosis.<br>Non-irradiated patients had no increased risk of second lung or esophagus cancer, neither overall nor<br>over time but did have increased risks for thyroid cancer (SIR 1.21) and sarcomas (SIR 1.42)<br>Conclusion: Radiotherapy for breast cancer is associated with an excess risk of second non-breast cancer, overall and in organs adjacent to the previous treatment fields highlighting the need for an improved individualized approach toward identifying patients with an expected benefit from radiation and patients with no added radiation-benefit.
Pan B, Xu Y, Zhou YD, et al. The prognostic comparison among unilateral, bilateral, synchronous bilateral, and metachronous bilateral breast cancer: A meta-analysis of studies from recent decade (2008-2018). Cancer Med. 2019 Jun;8(6):2908-2918.	A meta-analysis of 15 studies from 2008-2018, considering survival for unilateral, bilateral synchronous and bilateral metachronous early breast cancer was conducted<br>The summary HR of survival comparison between bilateral vs unilateral was 1.68, for bilateral synchronous vs unilateral was 2.01, and for bilateral metachronous vs unilateral was 3.22. When 3, 6, 12 months were used as the interval time, the summary HR of the survival comparison between bilateral synchronous vs bilateral metachronous were 0.64, 1.17, and 1.45) respectively.
Wang KY, Newman J, Lee CS, Seetharamu N. Epidemiology and clinicopathological features of lung cancer in patients with prior history of breast cancer. SAGE Open Medicine.2021,May; 9:20503121211017757.	Breast cancer is the most common malignancy in women, and lung cancer, the leading cause of cancer-related mortality in the United States, is the most common subsequent primary cancer among breast cancer survivors. A review was carried out of studies reporting lung cancer risk in breast cancer survivors. Some studies indicate that LC occurs more frequently in patients with prior history of BC compared to the general population. Smoking and radiotherapy are likely risk factors. Treatment with

	antiestrogen therapy in appropriate settings may serve as primary prevention in post-menopausal women with a high risk of developing LC. BC patients with high-risk characteristics, such as diagnosis at age less than 50, previous RT, triple-negative subtype, and history of breast implants, should also be more closely monitored for subsequent development of primary LC. A family history of cancer can also prompt testing for mutations such as TP53, BRCA, and EGFR
Parhizgar P, Bahadori Monfared A, Mohseny M et al. Risk of second primary cancer among breast cancer patients: A systematic review and meta-analysis. Frontiers in Oncology. 2023 Jan ;12.	30 articles published up to Oct 2022 were reviewed. The estimated SIR for second primary cancer for men and women was 1.28 and 1.27 respectively. Women diagnosed with breast cancer before menopause [1.52) were at higher risk than post-menopausal women {1.21 } as well as women after 10 years since their breast cancer diagnosis [1.33) vs. 1.24) were at a higher risk of developing second primary cancer. Among men, there were no differences in risk based on age. There is an extra risk of second primary cancer among breast cancer patients. The extra risk should be considered for further screening and preventive measures.
Allen I, Hend H, Sofianopoulou E, et al. Risks of second non-breast primaries following breast cancer in women: a systematic review and meta-analysis. Breast Cancer Research, 2023 Feb: 25: 18.	28 articles were reviewed. The summary SIR estimate was 1.24. This was higher in patients with a younger age at index cancer diagnosis and in Asian (SIR 1.47) as opposed to European (SIR 1.16) studies. There were higher SIRs for thyroid, uterine, ovarian, renal, oesophageal, lung, bladder, stomach and skin cancers as well as acute leukaemia.
