# Supplementary material for: Second cancers in 475 000 women with early invasive breast cancer diagnosed in England during 1993-2016: population based observational cohort study
Source: BMJ. 2025 Aug 27;390:e083975. doi: 10.1136/bmj-2024-083975 (PMC12381949; doi:10.1136/bmj-2024-083975)
Supplement: Supplementary file 2 — Web appendix: Analytical code [file mcgp083975.ww2.pdf]

Second cancers in 475 000 women with early invasive breast cancer diagnosed in England during 1993-2016: Population based observational cohort study

Supplementary Appendix 2 - Analytic Code (Stata v17)

Name of Stata procedure to produce main figures for paper **Page**

Name	Description	Page
Figures_1_2	Produced cumulative incidence plot for non-breast second cancers (combined) and contralateral cancers: - over all ages and by age group	2
Figure_3	Computes cumulative incidence for all available cancer groupings	16
Figure_4	Regressions to investigate associations between breast cancer treatments and second cancer risk	27

Names of Stata procedure subroutines called by main figure procedures

Name	Description	Page
var_cif	Calculates the variances of the cumulative incidence estimates	43
two_sigf	Displays a number to 2 sig figs or as <0.0001	45

The procedures run under Stata v17. De-personalised study data may be made available on request to accredited researchers who submit a proposal that is approved by NHS England's Data Access Request Service (DARS). Therefore, the data upon which these procedures operate has not been made publicly available.

```
* Procedure: Figures_1_2.do <factor> <time point>
*
* Produce a cumulative incidence plot for non-breast second cancers (combined) and contralateral
cancers
* competing risks are any second cancer event and death
*
* Rather than estimate the CIF from subhazards this program uses the probability of
* the event of interest
*
* If factor is not follow-up (FU) then the program tries to works out the 10-year cumulative
risks by the
* factor specified by the user
*
* Allowable factors
*      factor: 0=FU (doesn't work for this factor yet when tp=1)
*             1=Year of BCa diagnosis
*             2=Age at BCa diagnosis
*             3=IMD
*
* Time point to estimate the risk
*   time point: 0 = end of FU
*               1 = upto year 10 only
*
* User input: "0 0" produces figure 1
*           : "2 0" produces figure 2
*
* Calls var_cif.do (written by PMcG) to calculate variances of CIF estimates
* Calls two_sigf.do (written by PMcG) to display numbers to two significant figures
*
* PMcG: Mar 2024

*************
* Constants *
*************
* 95% CI
       local lvl=invnorm(1-(1-95/100)/2)

* Censoring date (set this up so can use most of 2021, in the cohort 2nd ca FU ends 31st Oct
2021)
       local cendat=date("31/10/2021","DMY")

* What calendar period to drop out
       local drp_yr=2022

**************
* User input *
**************

* Which factor (indicates which variable in data)
       local fac "`1'"
       if ~("`fac'"=="0" | "`fac'"=="1" | "`fac'"=="2" | "`fac'"=="3") local fac=0
       if `fac'==0 local facv=""
       if `fac'==1 local facv="yr_diag"
       if `fac'==2 local facv="cage"
       if `fac'==3 local facv="imd"

* Time point
       local tp "`2'"
       if `tp'==0 & `fac'==2 local facv="cage5"

       if `fac'==0 & `tp'==1 {
              no di "NOTE: Doesn't work for this FUxTP combination yet, EXITING"
              exit
       }

**************************************
* Breast cancer cohort analysis file *
**************************************
clear
use <<BCa Cohort file>>

noi di
noi di "**************************************************************************************"
noi di "******* MAKE SURE USING CORRECT CA_DG* FOR, NON-BCA,  BCA (cont, ips, lat?) & UNSPEC.*"
noi di "**************************************************************************************"
```

```
local nbcvar=""
local contvar=""
local ipsvar=""
local unspvar=""
* Unknown now added into contralaterals
foreach var of varlist ca_dg* {
* Note correct ones
	if `"`:variable label `var''"' == "Malignant neoplasms of all specified sites (excl breast & non-melanoma skin) and" local nbcvar="`var'"
	if `"`:variable label `var''"' == "Invasive breast (cont./unk.)" local contvar="`var'"
	if `"`:variable label `var''"' == "Invasive breast (ipsi.)" local ipsvar="`var'"
	if `"`:variable label `var''"' == "Malignant neoplasm of unspecified sites or multiple sites" local unspvar="`var'"
}

noi di "Using: `nbcvar', `contvar', `ipsvar', `unspvar'"
noi di


* Some labels
lab var yr_diag "Year breast cancer diagnosed"
lab var cage "Age at breast cancer diagnosis (years)"
lab var cage5 "Age at breast cancer diagnosis (years)"
lab def quintile 1 "<20%" 5 "80+%",modify

* Sort out deaths in relation to 2nd cancer so can use as competing risk ie ignore death
* after 2nd cancer
* Cancers only go to `cendat'
replace dead=0 if dead==1 & type_2ca<15
replace dead=0 if dead==1 & datlsn>`cendat'

* Use date of second cancer (index 1)
gen datdiag_2ca=datdiag_2ca1
replace datdiag_2ca=datlsn if datdiag_2ca==.
gen datexit=datdiag_2ca
replace datexit=`cendat' if datdiag_2ca>`cendat'


* Setup flag for events (nonBCa, cont BCa) and for competing risks (death, ipsi BCa, and
* other ill-spec non-BCa)
* For BCa, interested in contralateral as outcome
capture drop failtyp
gen failtyp=0
* All cancers excl. breast
replace failtyp=1 if `nbcvar'==1
* Cont. breast cancer
replace failtyp=2 if `contvar'==1
* Death, ispi breast, unspec
*replace failtyp=3 if dead==1 | `unspvar'==1  | `ipsvar'==1
replace failtyp=3 if dead==1 | `unspvar'==1  | `ipsvar'==1
lab var failtyp "Failure type"
lab def failtyp 0 "Censored" 1 "Non-BCa" 2 "Cont. BCa" 3 "Death/other", modify
lab val failtyp failtyp

gen nonbc=(failtyp==1)
gen cont=(failtyp==2)
gen other=(failtyp==3)
gen any=(failtyp>0)
lab var nonbc "New non-BCa"
lab var cont "Contralateral"
lab var other "Other cancer or death"
lab var any "Any event"

stset datdiag_2ca, id(anon_patient) ori(time datdiag) enter(datatrisk) exit(time datexit) fail(any) scale(365.25)
drop if _st==0

stsplit yrsdiag, at(0(1)max)
lab val yrsdiag yrsdiag
*lab var yrsdiag "Time since breast cancer diagnosis (years)"
lab var yrsdiag "Time since diagnosis of index breast cancer (years)"

*Note numbers at risk
preserve
if `fac'==0 {
```

```
* Save a version with annual numbers
	gen atrisk=1
	collapse (sum) atrisk, by(yrsdiag)
	sort yrsdiag
	save tmp_nums_annual,replace
	restore, preserve
	keep if yrsdiag==0 | yrsdiag==5 | yrsdiag==10 |  yrsdiag==20
	rena yrsdiag cyrsdiag
	recode cyrsdiag 5=1 10=2 20=3
	gen pop=1
	collapse (sum) pop, by(cyrsdiag)
	sort cyrsdiag
	save tmp_nums,replace
}
else {
* Save a version with annual numbers
	gen atrisk=1
	collapse (sum) atrisk, by(`facv' yrsdiag)
	sort `facv' yrsdiag
	save tmp_nums_annual,replace
	restore, preserve
	keep if yrsdiag==0
	gen pop=1
	collapse (sum) pop, by(`facv')
	sort `facv'
	save tmp_nums,replace
}
restore

* Split by period, age, FU, so can get expecteds
stsplit year=-21914, at(93(1)123)
replace year=year+1900
stsplit age=datob, at(0(5)90)

* Note bin width (sensitive to age)
* Get actual bin width
egen min_t0=min(_t0), by(yrsdiag `facv')
egen max_t=max(_t), by(yrsdiag `facv')
gen w=max_t-min_t0

* Save widths to a file (possible could clash with another file so
* give an exotic name).  Make sensitive to age.
preserve
keep yrsdiag w `facv'
bysort yrsdiag `facv' : keep if _n==1
compress
sort `facv' yrsdiag
save crcaa_fu_width,replace
restore

* Drop events after specific calendar period
drop if year>=`drp_yr'

* Popln data group ages after 85, drop out age>=85
drop if age>=85

* Update w-yrs and outcomes
gen pyrs=_t-_t0
replace any = _d

foreach var of varlist nonbc cont other {
	replace `var'=0 if any==0
}


* Match codes to pop. rates file
replace age=age+4
lab var age "Attained age (years)"
lab def agefmt  4 "0-4" 9 "5-9" 14 "10-14" 19 "15-19" 24 "20-24" 29 "25-29" 34 "30-34" 39 "35-
39" 44 "40-44" 49 "45-49" 54 "50-54" 59 "55-59" 64 "60-64" 69 "65-69" 74 "70-74" 79 "75-79" 84
"80-84" 89 "85-89" 90 "90+", modify
lab val age agefmt
* Drop out ages <20 and ages >84
drop if age<24 | age>84
```

```
lab var year "Current calendar year"


save tmp,replace

* Prepare to merge in pop counts
use tmp,clear

collapse (sum) nonbc cont other any pyrs, by(year age imd yrsdiag `facv')

* Rectangularise data for merge
if `fac' ==1 | `fac' ==2 fillin year age imd yrsdiag `facv'
if `fac' ==0 | `fac' ==3 fillin year age imd yrsdiag
replace nonbc=0 if _fillin==1
replace cont=0 if _fillin==1
replace other=0 if _fillin==1
replace any=0 if _fillin==1
replace pyrs=0 if _fillin==1
drop _fillin
sort year age imd
* All non-bca
merge m:1 year age imd using <<population non-breast-cancer rates file>>
drop if _merge==2
drop _merge
* All BCa (for cont, eventually need to mutliply by 0.5)
merge m:1 year age imd using <<population breast cancer rates file>>
drop if _merge==2
drop if age<24 | age>84
drop if year<1993 | year>=`drp_yr'

* Group up younger ages
*recode age 4 9 14 19 24 29 34 =39
*lab def age 39 "<40", modify

collapse (sum)  nonbc cont other any pop_`nbcvar' pop_`contvar' pyrs pop, by(year age imd
yrsdiag `facv')

gen pop_`nbcvar'_rate=pop_`nbcvar'/pop
recode pop_`nbcvar'_rate .=0
gen pop_`contvar'_rate=pop_`contvar'/pop
recode pop_`contvar'_rate .=0
drop pop pop_`nbcvar' pop_`contvar'

* Calculate expecteds
gen exp_nonbc=pyrs*pop_`nbcvar'_rate
lab var exp_nonbc "Expected non-breast cancers"

* For contralateral, just assume its half the all breast as L & R about 50:50
gen exp_cont=pyrs*pop_`contvar'_rate
replace exp_cont = 0.5*exp_cont
lab var exp_cont "Expected contralateral cancers"

* Now sum by follow-up year and event type
collapse (sum) nonbc cont other any exp_nonbc exp_cont pyrs, by(yrsdiag `facv')

* Merge in widths of FU windows
sort `facv' yrsdiag
sort yrsdiag
merge m:1 `facv' yrsdiag using crcaa_fu_width
drop if _merge==2
noi assert _merge==3 | (_merge==1 & pyrs==0)
drop _merge

* Now start to work on cumulative risks

* First need to get S for any event and then lag by 1 year so get get KM adjusted
* for competing risks
if `fac'==0 {
	sort yrsdiag
	gen crate=sum(w*any/pyrs)
	gen S_any=exp(-crate)
	gen F_any=1-S_any
	sort yrsdiag
	gen S_any_lag=S_any[_n-1]
}
```

```
else {
	bysort `facv' (yrsdiag) : gen crate=sum(w*any/pyrs)
	gen S_any=exp(-crate)
	gen F_any=1-S_any
	bysort `facv' (yrsdiag) : gen S_any_lag=S_any[_n-1]
}
replace S_any_lag=1 if yrsdiag==0
drop crate

* Work out KM (& SE) for each failure type
foreach var of varlist nonbc cont other exp_nonbc exp_cont {

	if `fac'==0 {
		sort yrsdiag
* KM
		gen cif_`var'=sum(S_any_lag*(1-exp(-w*`var'/pyrs)))
* SE
* Also do PMCGs method just as a check
		sort yrsdiag
		gen t_se_cif_`var'=sum(S_any_lag*S_any_lag*(exp(-w*`var'/pyrs)*exp(-
w*`var'/pyrs))*w*w*`var'/(pyrs*pyrs))
		replace t_se_cif_`var'=100*sqrt(t_se_cif_`var')
		noi run var_cif yrsdiag w "`var'" any pyrs "se_cif_`var'"
* CIs
		gen cif_`var'_ll =100*(cif_`var'^(exp(-
`lvl'*se_cif_`var'/(cif_`var'*log(cif_`var')))))
		gen cif_`var'_ul
=100*(cif_`var'^(exp(`lvl'*se_cif_`var'/(cif_`var'*log(cif_`var')))))
		replace cif_`var'=100*cif_`var'
		replace se_cif_`var'=100*se_cif_`var'
	}
	else {
* MK
		bysort `facv' (yrsdiag) : gen cif_`var'=sum(S_any_lag*(1-exp(-w*`var'/pyrs)))
* SE
* Also do PMCGs method just as a check
		bysort `facv' (yrsdiag) : gen t_se_cif_`var'=sum(S_any_lag*S_any_lag*(exp(-
w*`var'/pyrs)*exp(-w*`var'/pyrs))*w*w*`var'/(pyrs*pyrs))
		replace t_se_cif_`var'=100*sqrt(t_se_cif_`var')

* Have to loop round factor groups and then merge in SE
		local i=1
		levelsof `facv', local(levels)
		foreach lev of local levels {
			preserve
			keep if `facv' == `lev'
			noi run var_cif yrsdiag w "`var'" any pyrs "se_cif_`var'"
			keep `facv' yrsdiag se_cif_`var'
			if `i'==1 {
				sort `facv' yrsdiag
				save tmp_se,replace
			}
			else {
				append using tmp_se
				sort `facv' yrsdiag
				save tmp_se,replace
			}
			restore
			local i = `i' + 1
		}
* Merge in SE (for expects, one should really treat the SE as 0)
		sort `facv' yrsdiag
		merge 1:1 `facv' yrsdiag using tmp_se
		assert _merge==3
		drop _merge
* CIs
		gen cif_`var'_ll =100*(cif_`var'^(exp(-
`lvl'*se_cif_`var'/(cif_`var'*log(cif_`var')))))
		gen cif_`var'_ul
=100*(cif_`var'^(exp(`lvl'*se_cif_`var'/(cif_`var'*log(cif_`var')))))
		replace cif_`var'=100*cif_`var'
		replace se_cif_`var'=100*se_cif_`var'

	}
}
```

```
noi list `facv' yrsdiag t_se_cif_nonbc se_cif_nonbc t_se_cif_cont se_cif_cont t_se_cif_other
se_cif_other if mod(yrsdiag,5)==0, noobs clean

*P for difference in risk
*foreach var of varlist nonbc cont  {
*       gen diff_`var'=abs(cif_`var'-cif_exp_`var')
*       gen z_`var'=diff_`var'/se_cif_`var'
*       gen p_`var'=2*(1-normprob(z_`var'))
*}

* Use SIR to represent difference
* For time since, get SIR for each period
if `fac'==0 {
       local facv="cyrsdiag"
       capture drop cyrsdiag
       gen cyrsdiag=0 if yrsdiag>=0 & yrsdiag<=4
       replace cyrsdiag=1 if yrsdiag>=5 & yrsdiag<=9
       replace cyrsdiag=2 if yrsdiag>=10 & yrsdiag<=19
       replace cyrsdiag=3 if yrsdiag>=20 & yrsdiag~=.
       lab var cyrsdiag "Time since breast cancer diagnosis (years)"
       lab def cyrsdiag 0 "3m-4" 1 "5-9" 2 "10-19" 3 "20-29",modify
       lab val cyrsdiag cyrsdiag
}
* Add in numbers at risk
* If time since analysis add in annual numbers at risk
if `fac'==0 {
       sort yrsdiag
       merge m:1 yrsdiag using tmp_nums_annual
       drop if _merge==2
       drop _merge
}
else {
       sort `facv' yrsdiag
       merge m:1 `facv' yrsdiag using tmp_nums_annual
       drop if _merge==2
       drop _merge
}

sort `facv'
merge m:1 `facv' using tmp_nums
drop if _merge==2
drop _merge
* For diag year and age, proper SIR comparison is at 10 years
local y10=""
if `fac'==1 | `fac'==2 local y10="& yrsdiag<=9"
levelsof `facv', local(levels)
foreach var of varlist nonbc cont  {
       gen sir_`var'=.
       gen sir_`var'_ll=.
       gen sir_`var'_ul=.
       gen p_`var'=.
       gen aer_`var'=.
       gen aer_`var'_ll=.
       gen aer_`var'_ul=.
       if `fac'==1 | `fac'==2 egen t_`var'=sum(`var')  if yrsdiag<=9, by(`facv')
       if `fac'==0 | `fac'==3 egen t_`var'=sum(`var'), by(`facv')
       local i=1
       if `fac'==0  local r_`var'0="Time  N  n  SIR (95% CI)"
       if `fac'==1  local r_`var'0="Year  N  n  SIR (95% CI) CIF (95% CI)"
       if `fac'==2  local r_`var'0="Age   N  n  SIR (95% CI) CIF (95% CI)"
       if `fac'==3  local r_`var'0="IMD   N  n  SIR (95% CI) CIF (95% CI)"
*      if `fac'==0  local r_`var'0="Time  N  n  SIR (95% CI) AER (95% CI)"
*      if `fac'==1  local r_`var'0="Year  N  n  SIR (95% CI) CIF (95% CI) AER (95% CI)"
*      if `fac'==2  local r_`var'0="Age   N  n  SIR (95% CI) CIF (95% CI) AER (95% CI)"
*      if `fac'==3  local r_`var'0="IMD   N  n  SIR (95% CI) CIF (95% CI) AER (95% CI)"
*      if `fac'==0 & "`var'"=="cont" local r_`var'0="n  SIR (95% CI) AER (95% CI)"
*      if `fac'==1 & "`var'"=="cont" local r_`var'0="n  SIR (95% CI) CIF (95%) AER (95% CI)"
*      if `fac'==2 & "`var'"=="cont" local r_`var'0="n  SIR (95% CI) CIF (95%) AER (95% CI)"
*      if `fac'==3 & "`var'"=="cont" local r_`var'0="n  SIR (95% CI) CIF (95%) AER (95% CI)"
       local  r_`var'99=" "
       if `fac'==1 | `fac'==2 local r_`var'99="By 10 years since diagnosis:"
       if `fac'==3            local r_`var'99="All years"
       foreach lev of local levels {
```

```
* Setup a record to use as the origin on the plots
             if "`var'"=="nonbc" {
                    local nobs=_N
                    local nobs=`nobs'+1
                    if `fac'==0 & `i'==1 {
                           set obs `nobs'
                           replace yrsdiag=-1 if _n==`nobs'
                    }
                    else if `fac'>0  {
                           set obs `nobs'
                           replace yrsdiag=-1 if _n==`nobs'
                           replace `facv'=`lev' if _n==`nobs'
                    }
             }

             local r_`var'`i'=""
             local pop=""
             local tcan=""
             local sir=""
             local sir_ll=""
             local sir_ul=""
             local aer=""
             local aer_ll=""
             local aer_ul=""
             local lv_lab : label (`facv') `lev'
             poisson `var' if exp_`var'>0 & `facv'==`lev' `y10', exp(exp_`var')
             replace sir_`var'=exp(_b[_cons]) if `facv'==`lev' `y10'
             replace sir_`var'_ll=exp(_b[_cons]-`lvl'*_se[_cons]) if `facv'==`lev' `y10'
             replace sir_`var'_ul=exp(_b[_cons]+`lvl'*_se[_cons]) if `facv'==`lev' `y10'
             replace p_`var'=2*(1-normprob(abs(_b[_cons]/_se[_cons]))) if `facv'==`lev' `y10'
             summ pyrs if `facv'==`lev' `y10'
             local spyrs=r(sum)
             summ `var' if `facv'==`lev' `y10'
             local O=r(sum)
             summ exp_`var' if `facv'==`lev' `y10'
             local E=r(sum)
             replace aer_`var'= 1e4*(`O'-`E')/`spyrs' if `facv'==`lev' `y10'
             replace aer_`var'_ll= 1e4*`E'*(sir_`var'_ll-1)/`spyrs' if `facv'==`lev' `y10'
             replace aer_`var'_ul= 1e4*`E'*(sir_`var'_ul-1)/`spyrs' if `facv'==`lev' `y10'

* Hold summary result for plots
             summ t_`var' if `facv'==`lev' `y10'
             local tcan=r(mean)
             summ pop if `facv'==`lev'
             local pop=r(mean)
             summ sir_`var' if `facv'==`lev' `y10'
             local sir=r(mean)
             summ sir_`var'_ll if `facv'==`lev' `y10'
             local sir_ll=r(mean)
             summ sir_`var'_ul if `facv'==`lev' `y10'
             local sir_ul=r(mean)
             summ cif_`var' if `facv'==`lev' & yrsdiag==9
             local cif=r(mean)
             summ cif_`var'_ll if `facv'==`lev' & yrsdiag==9
             local cif_ll=r(mean)
             summ cif_`var'_ul if `facv'==`lev' & yrsdiag==9
             local cif_ul=r(mean)
             summ aer_`var' if `facv'==`lev'  `y10'
             local aer=r(mean)
             summ aer_`var'_ll if `facv'==`lev' `y10'
             local aer_ll=r(mean)
             summ aer_`var'_ul if `facv'==`lev' `y10'
             local aer_ul=r(mean)

             if `fac'==0  local r_`var'`i'="`lv_lab'  `pop'  `tcan' "+string(`sir',"%5.2f")+"
("+string(`sir_ll',"%5.2f")+","+string(`sir_ul',"%5.2f")
             if `fac'>0   local r_`var'`i'="`lv_lab'  `pop'  `tcan' "+string(`sir',"%5.2f")+"
("+string(`sir_ll',"%5.2f")+","+string(`sir_ul',"%5.2f")+") " + string(`cif',"%4.1f")+"
("+string(`cif_ll',"%4.1f")+","+string(`cif_ul',"%4.1f")+")"
*            if `fac'==0  local r_`var'`i'="`lv_lab'  `pop'  `tcan' "+string(`sir',"%5.2f")+"
("+string(`sir_ll',"%5.2f")+","+string(`sir_ul',"%5.2f")+") "  + string(`aer',"%5.2f")+"
("+string(`aer_ll',"%5.2f")+","+string(`aer_ul',"%5.2f")+")"
*            if `fac'>0   local r_`var'`i'="`lv_lab'  `pop'  `tcan' "+string(`sir',"%5.2f")+"
("+string(`sir_ll',"%5.2f")+","+string(`sir_ul',"%5.2f")+") " + string(`cif',"%4.1f")+"
```

```
("+string(`cif_ll',"%4.1f")+","+string(`cif_ul',"%4.1f")+")" +string(`aer',"%5.2f")+"
("+string(`aer_ll',"%5.2f")+","+string(`aer_ul',"%5.2f")+")"
*           if `fac'==0 & "`var'"=="cont" local r_`var'`i'="`tcan' "+string(`sir',"%5.2f")+"
("+string(`sir_ll',"%5.2f")+","+string(`sir_ul',"%5.2f")+")"
*           if `fac'>0 & "`var'"=="cont"   local r_`var'`i'="`tcan' "+string(`sir',"%5.2f")+"
("+string(`sir_ll',"%5.2f")+","+string(`sir_ul',"%5.2f")+") " + string(`cif',"%4.1f")+"
("+string(`cif_ll',"%4.1f")+","+string(`cif_ul',"%4.1f")+")"

* For yrs since, add an overal total
            if `fac'==0 & `lev'==3 {
                  poisson `var' if exp_`var'>0 , exp(exp_`var')
                  replace sir_`var'=exp(_b[_cons])
                  replace sir_`var'_ll=exp(_b[_cons]-`lvl'*_se[_cons])
                  replace sir_`var'_ul=exp(_b[_cons]+`lvl'*_se[_cons])
                  replace p_`var'=2*(1-normprob(abs(_b[_cons]/_se[_cons])))
                  summ pyrs
                  local spyrs=r(sum)
                  summ `var'
                  local O=r(sum)
                  summ exp_`var'
                  local E=r(sum)
                  replace aer_`var'= 1e4*(`O'-`E')/`spyrs'
                  replace aer_`var'_ll= 1e4*`E'*(sir_`var'_ll-1)/`spyrs'
                  replace aer_`var'_ul= 1e4*`E'*(sir_`var'_ul-1)/`spyrs'

* Hold summary result for plots
                  summ `var'
                  local tcan=r(sum)
                  summ pop if yrsdiag==0
                  local pop=r(mean)
                  local r_`var'5="All  `pop'  `tcan' "+string(sir_`var',"%5.2f")+"
("+string(sir_`var'_ll,"%5.2f")+","+string(sir_`var'_ul,"%5.2f")+")"
*                 local r_`var'5="All  `pop'  `tcan' "+string(sir_`var',"%5.2f")+"
("+string(sir_`var'_ll,"%5.2f")+","+string(sir_`var'_ul,"%5.2f")+") " +
string(aer_`var',"%5.2f")+" ("+string(aer_`var'_ll,"%5.2f")+","+string(aer_`var'_ul,"%5.2f")+")"
*                 if "`var'"=="cont"  local r_`var'5="`tcan' "+string(sir_`var',"%5.2f")+"
("+string(sir_`var'_ll,"%5.2f")+","+string(sir_`var'_ul,"%5.2f")+")"
            }

* Next level
            local i=`i'+1
      }
}


* Don't plot estimates with pyrs=0
drop if pyrs==0


if `tp'==0 {
* Over all FU years
      drop if se_cif_nonbc==. & yrsdiag>=0
      drop if cif_nonbc>25 & yrsdiag>=0

* Setup vars for origin
      replace yrsdiag=yrsdiag+1
      replace yrsdiag=0.25 if yrsdiag==0
      foreach var of varlist cif* {
            recode `var' .=0 if yrsdiag==.25
      }
      capture drop idx
      bysort `facv' (yrsdiag) : gen idx=_n
      format sir* %5.2f
      noi list `facv' pop sir_nonbc sir_nonbc_ll sir_nonbc_ul sir_cont sir_cont_ll sir_cont_ul
if idx==2,clean noobs
      sort yrsdiag
      if `fac'==0 {
* Time since diagnosis factor

* Some % points to plot
            local pvar "y_nbc y_enbc pnbc penbc y_cont y_econt pcont pecont"
            foreach v of local pvar {
                  capture drop `v'
                  if substr("`v'",1,1)=="p" {
                        gen `v'=""
```

```
                }
                else {
                        gen `v'=.
                }
        }
        capture drop scale
        gen scale=1.2
        replace scale=1.1 if yrsdiag>10
        forvalues x=5 10 to 25 {

* Obsvd
                summ cif_nonbc if yrsdiag==`x'
                local nbc=round(r(mean),.1)
                replace y_nbc=`nbc'*scale if yrsdiag==`x'
                replace pnbc=string(`nbc',"%3.1f") if yrsdiag==`x'
                summ cif_cont if yrsdiag==`x'
                local cont=round(r(mean),.1)
                replace y_cont=`cont'*scale*scale if yrsdiag==`x'
                replace pcont=string(`cont',"%3.1f") if yrsdiag==`x'

* Exp
                summ cif_exp_nonbc if yrsdiag==`x'
                local enbc=round(r(mean),.1)
                replace y_enbc=`enbc'*1/scale if yrsdiag==`x'
                replace penbc=string(`enbc',"%3.1f") if yrsdiag==`x'
                summ cif_exp_cont if yrsdiag==`x'
                local econt=round(r(mean),.1)
                replace y_econt=`econt'*1/(scale*scale) if yrsdiag==`x'
                replace pecont=string(`econt',"%3.1f") if yrsdiag==`x'
        }

* Make new text to appear to plot
        foreach var of varlist nonbc cont {
                summ `var'
                local n_`var'=r(sum)
                local r_`var'0="`n_`var'' cancers"
                local r_`var'1="Time      risk (%)    risk (%) (95% CI)"
                local i=2
                forval t= 5(5)25 {
                        summ cif_`var' if yrsdiag==`t'
                        local o_cif=r(sum)
                        summ se_cif_`var' if yrsdiag==`t'
                        local se_o_cif=r(sum)
                        summ cif_exp_`var' if yrsdiag==`t'
                        local e_cif=r(sum)
                        local ex_cif =  `o_cif' -  `e_cif'
                        local ex_cif_ll =  `ex_cif' - `lvl'*`se_o_cif'
                        local ex_cif_ul =  `ex_cif' + `lvl'*`se_o_cif'
                        local r_`var'`i' ="`t'        " + string(`o_cif',"%4.2f")+ "         "
+string(`ex_cif',"%4.2f")+  " ("+string(`ex_cif_ll',"%4.2f")+","+string(`ex_cif_ul',"%4.2f")+")"
                        local i = `i'+1
                }
        }
        twoway rarea cif_nonbc_ll cif_nonbc_ul yrsdiag, col(navy) || scatter y_nbc y_enbc
yrsdiag, ms(i i) mlabpos(0 0) mlabsiz(*.9 *.9) mla(pnbc penbc) mlabc(navy maroon)   || scatter
cif_nonbc cif_exp_nonbc yrsdiag, recast(line) lp(solid dash) lc(navy maroon) ylab(0(5)20,nogrid
ang(0)) xlab(0(10)30) yt("Cumulative risk % (95% CI)", siz(*1)) legend(cols(1) ring(0) pos(9)
symx(*.3) size(*.5) keygap(*.1) region(style(none)))  text(18 2 "{bf:`r_nonbc0'}"
"{bf:`r_nonbc1'}" "`r_nonbc2'" "`r_nonbc3'" "`r_nonbc4'" "`r_nonbc5'" "`r_nonbc6'",
placement(east) justification(left) siz(*.8)) graphr(color(white) lcolor(white))
saving(gr_a,replace)
        twoway rarea cif_cont_ll cif_cont_ul yrsdiag, col(navy)   || scatter y_cont y_econt
yrsdiag, ms(i i) mlabpos(0 0) mlabsiz(*.9 *.9) mla(pcont pecont) mlabc(navy maroon)  || scatter
cif_cont cif_exp_cont yrsdiag, recast(line) lp(solid dash) lc(navy maroon) ylab(0(5)20,nogrid
ang(0)) xlab(0(10)30) yt("Cumulative risk % (95% CI)", siz(*1)) legend(cols(1) ring(0) pos(9)
symx(*.3) size(*.5) keygap(*.1) region(style(none))) text(18 2 "{bf:`r_cont0'}"
"{bf:`r_cont1'}"  "`r_cont2'" "`r_cont3'" "`r_cont4'" "`r_cont5'" "`r_cont6'",  placement(east)
justification(left) siz(*.8)) graphr(color(white) lcolor(white)) saving(gr_b,replace)

* Save a version of the datafor BMJ dynamic plot
        save bmj_fig1_data,replace

    }
    else {
* All other factors
```

```
            sepscatter cif_nonbc yrsdiag, sep(`facv') recast(line) ylab(0(5)20,nogrid ang(0))
xlab(0(10)30) yt("Cumulative risk %", siz(*1)) legend(cols(1) ring(0) pos(9) symx(*.3) size(*.5)
keygap(*.1) region(style(none)))  text(19 2  "`r_nonbc99'" "{bf:`r_nonbc0'}"  "`r_nonbc1'"
"`r_nonbc2'" "`r_nonbc3'" "`r_nonbc4'" "`r_nonbc5'",  placement(east) justification(left)
siz(*.8)) graphr(color(white) lcolor(white)) saving(gr_a,replace)
            sepscatter cif_cont  yrsdiag, sep(`facv') recast(line) ylab(0(5)20,nogrid ang(0))
xlab(0(10)30) yt("Cumulative risk %", siz(*1)) legend(cols(1) ring(0) pos(9) symx(*.3) size(*.5)
keygap(*.1) region(style(none))) text(19 2  "`r_cont99'" "{bf:`r_cont0'}"  "`r_cont1'"
"`r_cont2'" "`r_cont3'" "`r_cont4'" "`r_cont5'",  placement(east) justification(left) siz(*.8))
graphr(color(white) lcolor(white)) saving(gr_b,replace)
* With CIs
            local col_array "navy maroon forest_green dkorange teal cranberry lavender khaki
sienna emidblue emerald brown erose gold bluishgray"
            local i=1
            local nbc_plt=""
            local cnt_plt=""
            local clrs=""
            foreach lev of local levels {
* Which colour for line
                  local lc`i' : word `i' of `col_array'
                  local clrs="`clrs' `lc`i''"
                  if `i'==1 {
                        local nbc_plt="twoway rarea cif_nonbc_ll cif_nonbc_ul yrsdiag if
`facv'==`lev', col(`lc`i'')"
                        local cnt_plt="twoway rarea cif_cont_ll cif_cont_ul yrsdiag if
`facv'==`lev', col(`lc`i'')"
                  }
                  else {
                        local nbc_plt="`nbc_plt' || rarea cif_nonbc_ll cif_nonbc_ul yrsdiag if
`facv'==`lev',  col(`lc`i'')"
                        local cnt_plt="`cnt_plt' || rarea cif_cont_ll cif_cont_ul yrsdiag if
`facv'==`lev',  col(`lc`i'')"
                  }
* Next level
                  local i=`i'+1
            }
            gen cifa=cif_nonbc
            gen cifb=cif_cont
            separate cifa, by(`facv')
            separate cifb, by(`facv')
            drop cifa cifb
            `nbc_plt' || scatter cifa* yrsdiag, recast(line) lc(`clrs') ylab(0(5)20,nogrid
ang(0)) xlab(0(10)30) yt("Cumulative risk % (95% CI)", siz(*1)) legend(cols(1) ring(0) pos(9)
symx(*.3) size(*.5) keygap(*.1) region(style(none))) text(19 2 "`r_nonbc99'" "{bf:`r_nonbc0'}"
"`r_nonbc1'" "`r_nonbc2'" "`r_nonbc3'" "`r_nonbc4'" "`r_nonbc5'",  placement(east)
justification(left) siz(*.8)) graphr(color(white) lcolor(white)) saving(gr_a,replace)
            `cnt_plt' || scatter cifb* yrsdiag, recast(line) lc(`clrs') ylab(0(5)20,nogrid
ang(0)) xlab(0(10)30) yt("Cumulative risk % (95% CI)", siz(*1)) legend(cols(1) ring(0) pos(9)
symx(*.3) size(*.5) keygap(*.1) region(style(none))) text(19 2 "`r_cont99'" "{bf:`r_cont0'}"
"`r_cont1'" "`r_cont2'" "`r_cont3'" "`r_cont4'" "`r_cont5'",  placement(east)
justification(left) siz(*.8)) graphr(color(white) lcolor(white)) saving(gr_b,replace)

      }
      gr combine gr_a.gph gr_b.gph,  xsize(11.5) ysize(5) cols(2) imargin(zero)
graphr(color(white) lcolor(white)) saving(gr_obs_v_pop_comp_risk_`facv'_tp`tp',replace)
      gr export gr_obs_v_pop_comp_risk_`facv'_tp`tp'.pdf,replace

}
else if `tp'==1 {
* Plot 10 year risk by age (and event type)
      keep if yrsdiag==9

* Max values (for plotting labels)
      local i=1
      foreach var of varlist cif_nonbc cif_exp_nonbc cif_cont cif_exp_cont {
            sort `var'
            local max`i'=`var'[_N]
            local i= `i'+1
*           local max=`var'[_N]
*           local pies  `"  "blueberry cake" "'
*           local olab "`olab' text(`max' 24 "  `"`pies'"' ")"
      }


* Setup plotting relevant to factor variable
```

```
* Factor label
	local facv_lab : variable label `facv'
	if `fac'==1 {
* Diag yr
		local xrng="1993,2015"
		local xtxt=2013
		local xl="1995(5)2010,value nogrid"
		local max1 = `max1' +1
		local max2 = `max2' - 0.5
		local max3 = `max3' + .5
		local max4 = `max4' - .5
	}
	else if `fac'==2 {
* Age
		local xrng="35,85"
		local xtxt=80
		local xl="39(5)74,value nogrid"
		local max1 = `max1' +0.8
		local max2 = `max2' - 0.7
		local max3 = `max3' -.2
		local max4 = `max4' - .4
	}
	else if `fac'==3 {
* IMD
		local xrng="0.5,6.2"
		local xtxt=5.7
		local xl="1(1)5,value nogrid"
		local max1 = `max1' +.8
		local max2 = `max2' - 0.3
		local max3 = `max3' + 0.3
		local max4 = `max4' - .5
	}

	capture drop cen_`facv'
	gen cen_`facv'=`facv'-2.5
	sort `facv'

*	twoway rarea cif_nonbc_ll cif_nonbc_ul `facv', lc(navy) lw(*1.2) || rarea cif_cont_ll
cif_cont_ul `facv', lc(maroon) lw(*1.2) || scatter cif_nonbc cif_exp_nonbc cif_cont cif_exp_cont
`facv', lw(*1.2) c(l l l l) lc(navy navy maroon maroon) msym(i i i i) lp(solid dash solid dash)
legend(off) yt("10 year cumulative risk % (95% CI)", siz(*1.2))  ylab(0(2)12,ang(0)
labsiz(*1.2)) xt("`facv_lab'",siz(*1.2)) xlab(,grid) xscale(range(`xrng')) text(`max1' `xtxt'
"Non-breast" "cancer", c(navy) j(left) siz(*1.2)) text(`max2' `xtxt' "Population" "expected",
c(navy) j(left) siz(*1.2)) text(`max5' `xtxt' "Contralateral" "breast cancer", c(maroon) j(left)
siz(*1.2)) text(`max4' `xtxt' "Population" "expected", c(maroon) j(left) siz(*1.2))
graphr(color(white) lcolor(white)) saving(gr_obs_v_pop_comp_risk_`facv',replace)
* No grid
	twoway rarea cif_nonbc_ll cif_nonbc_ul `facv', lc(navy) lw(*1.2) || rarea cif_cont_ll
cif_cont_ul `facv', lc(maroon) lw(*1.2) || scatter cif_nonbc cif_exp_nonbc cif_cont cif_exp_cont
`facv', lw(*1.2) c(l l l l) lc(navy navy maroon maroon) msym(i i i i) lp(solid dash solid dash)
legend(off) yt("10 year cumulative risk % (95% CI)", siz(*1.2))  ylab(0(5)20, nogrid ang(0)
labsiz(*1.2)) xlab(`xl') xt("`facv_lab'",siz(*1.2)) xlab(,nogrid) xscale(range(`xrng'))
text(`max1' `xtxt' "Non-breast" "cancer", c(navy) j(left) siz(*1.2)) text(`max2' `xtxt'
"Population" "expected", c(navy) j(left) siz(*1.2)) text(`max3' `xtxt' "Contralateral" "breast
cancer", c(maroon) j(left) siz(*1.2)) text(`max4' `xtxt' "Population" "expected", c(maroon)
j(left) siz(*1.2))   graphr(color(white) lcolor(white))
saving(gr_obs_v_pop_comp_risk_`facv'_tp`tp',replace)
	gr export gr_obs_v_pop_comp_risk_`facv'_tp`tp'.pdf,replace
}


* If age do a version with individual plots for age group
* Get max value
local mcif=0
foreach var of varlist cif*nonbc*ul cif*cont*ul {
	summ `var'
	if r(max) > `mcif' local mcif= r(max)
}
local mcif=int(`mcif'+1)
if `fac'==2 {

* Save a version of the data for BMJ dynamic plot
	save bmj_fig2_data,replace

	levelsof `facv', local(levels)
```

```
      foreach l of local levels {
* Keep relevant age
             preserve
             keep if `facv'==`l'
* Get SIRs
             poisson  nonbc if exp_nonbc>0, exp(exp_nonbc)
             local sir=exp(_b[_cons])
             local sir_ll=exp(_b[_cons]-`lvl'*_se[_cons])
             local sir_ul=exp(_b[_cons]+`lvl'*_se[_cons])
             local p=2*(1-normprob(abs(_b[_cons]/_se[_cons])))
             local sir_nbc=string(`sir',"%4.2f") + " (" + string(`sir_ll',"%4.2f") + ", " +
string(`sir_ul',"%4.2f")+")"
             run two_sigf `p'
             local p_nbc  : di  "$osign" " " "$onum"
             poisson  cont if exp_cont>0, exp(exp_cont)
             local sir=exp(_b[_cons])
             local sir_ll=exp(_b[_cons]-`lvl'*_se[_cons])
             local sir_ul=exp(_b[_cons]+`lvl'*_se[_cons])
             local p=2*(1-normprob(abs(_b[_cons]/_se[_cons])))
             local sir_cnt=string(`sir',"%4.2f") + " (" + string(`sir_ll',"%4.2f") + ", " +
string(`sir_ul',"%4.2f")+")"
             run two_sigf `p'
             local p_cnt  : di  "$osign" " " "$onum"
             sum pop
             local N=r(mean)
             sum nonbc
             local n_nbc=r(sum)
             sum cont
             local n_cnt=r(sum)

             local lval : label (`facv') `l'

* Some % points to plot
             local pvar "y_nbc y_enbc pnbc penbc y_cont y_econt pcont pecont"
             foreach v of local pvar {
                    capture drop `v'
                    if substr("`v'",1,1)=="p" {
                           gen `v'=""
                    }
                    else {
                           gen `v'=.
                    }
             }
             capture drop scale
             gen scale=1.2
             replace scale=1.1 if yrsdiag>10
             forvalues x=5 10 to 25 {

* Obsvd
                    summ cif_nonbc if yrsdiag==`x'
                    local nbc=round(r(mean),.1)
                    replace y_nbc=`nbc'*scale if yrsdiag==`x'
                    replace pnbc=string(`nbc',"%3.1f") if yrsdiag==`x'
                    summ cif_cont if yrsdiag==`x'
                    local cont=round(r(mean),.1)
                    replace y_cont=`cont'*scale*scale if yrsdiag==`x'
                    replace pcont=string(`cont',"%3.1f") if yrsdiag==`x'

* Exp
                    summ cif_exp_nonbc if yrsdiag==`x'
                    local enbc=round(r(mean),.1)
                    replace y_enbc=`enbc'*1/scale if yrsdiag==`x'
                    replace penbc=string(`enbc',"%3.1f") if yrsdiag==`x'
                    summ cif_exp_cont if yrsdiag==`x'
                    local econt=round(r(mean),.1)
                    replace y_econt=`econt'*1/(scale*scale) if yrsdiag==`x'
                    replace pecont=string(`econt',"%3.1f") if yrsdiag==`x'
             }
*            twoway rarea cif_nonbc_ll cif_nonbc_ul yrsdiag, lc(navy) || scatter y_nbc y_enbc
yrsdiag, ms(i i) mlabpos(0 0) mlabsiz(*.9 *.9) mla(pnbc penbc) mlabc(navy navy)    || scatter
cif_nonbc cif_exp_nonbc yrsdiag, recast(line) lp(solid dash) lc(navy navy) ylab(0(5)20,nogrid
ang(0)) xlab(0(10)30) yt("Cumulative risk % (95% CI)", siz(*1)) legend(cols(1) ring(0) pos(9)
symx(*.3) size(*.5) keygap(*.1) region(style(none)))  text(19 10 "{bf:`r_nonbc0'}"  "`r_nonbc1'"
"`r_nonbc2'" "`r_nonbc3'" "`r_nonbc4'" "`r_nonbc5'", j(left) siz(*.8)) graphr(color(white)
lcolor(white)) saving(gr_a,replace)
```

```
*            twoway rarea cif_cont_ll cif_cont_ul yrsdiag, lc(navy)   || scatter y_cont y_econt
yrsdiag, ms(i i) mlabpos(0 0) mlabsiz(*.9 *.9) mla(pcont pecont) mlabc(navy navy)  || scatter
cif_cont cif_exp_cont yrsdiag, recast(line) lp(solid dash) lc(navy navy) ylab(0(5)20,nogrid
ang(0)) xlab(0(10)30) yt("Cumulative risk % (95% CI)", siz(*1)) legend(cols(1) ring(0) pos(9)
symx(*.3) size(*.5) keygap(*.1) region(style(none))) text(19 9 "{bf:`r_cont0'}"  "`r_cont1'"
"`r_cont2'" "`r_cont3'" "`r_cont4'" "`r_cont5'", j(left) siz(*.8)) graphr(color(white)
lcolor(white)) saving(gr_b,replace)
*            twoway rarea cif_nonbc_ll cif_nonbc_ul yrsdiag, col(maroon) || scatter y_nbc y_enbc
yrsdiag, ms(i i) mlabpos(0 0) mlabsiz(*.9 *.9) mla(pnbc penbc) mlabc(maroon navy)   || scatter
cif_nonbc cif_exp_nonbc yrsdiag, recast(line) lp(solid dash) lc(maroon navy) ylab(0(5)20,nogrid
ang(0)) xlab(0(10)30) yscale(range(0 `mcif')) yt("Cumulative risk % (95% CI)", siz(*1)) text(20
0 "{bf: Age `lval' at diagnosis}" "`N' women, `n_nbc' cancers" "SIR `sir_nbc'; p `p_nbc'",
placement(east) justification(left) size(*1.1)) legend(off) graphr(color(white) lcolor(white))
saving(gr_a,replace)
*            twoway rarea cif_cont_ll cif_cont_ul yrsdiag, col(maroon)   || scatter y_cont
y_econt yrsdiag, ms(i i) mlabpos(0 0) mlabsiz(*.9 *.9) mla(pcont pecont) mlabc(maroon navy)  ||
scatter cif_cont cif_exp_cont yrsdiag, recast(line) lp(solid dash) lc(maroon navy)
ylab(0(5)20,nogrid ang(0)) xlab(0(10)30) yscale(range(0 `mcif')) yt("Cumulative risk % (95%
CI)", siz(*1)) text(20 0 "{bf: Age `lval' at diagnosis}" "`N' women, `n_cnt' cancers"  "SIR
`sir_cnt'; p `p_cnt'", placement(east) justification(left) size(*1.1)) legend(off)
graphr(color(white) lcolor(white)) saving(gr_b,replace)
             twoway rarea cif_nonbc_ll cif_nonbc_ul yrsdiag, col(navy) || scatter y_nbc y_enbc
yrsdiag, ms(i i) mlabpos(0 0) mlabsiz(*.9 *.9) mla(pnbc penbc) mlabc(navy maroon)   || scatter
cif_nonbc cif_exp_nonbc yrsdiag, recast(line) lp(solid dash) lc(navy maroon) ylab(0(5)20,nogrid
ang(0)) xlab(0(10)30) yscale(range(0 `mcif')) yt("Cumulative risk % (95% CI)", siz(*1)) text(20
0 "{bf: Age `lval' at diagnosis}" "`N' women, `n_nbc' cancers" "SIR `sir_nbc'", placement(east)
justification(left) size(*1.1)) legend(off) graphr(color(white) lcolor(white))
saving(gr_a,replace)
             twoway rarea cif_cont_ll cif_cont_ul yrsdiag, col(navy)   || scatter y_cont y_econt
yrsdiag, ms(i i) mlabpos(0 0) mlabsiz(*.9 *.9) mla(pcont pecont) mlabc(navy maroon)  || scatter
cif_cont cif_exp_cont yrsdiag, recast(line) lp(solid dash) lc(navy maroon) ylab(0(5)20,nogrid
ang(0)) xlab(0(10)30) yscale(range(0 `mcif')) yt("Cumulative risk % (95% CI)", siz(*1)) text(20
0 "{bf: Age `lval' at diagnosis}" "`N' women, `n_cnt' cancers" "SIR `sir_cnt'", placement(east)
justification(left) size(*1.1)) legend(off)  graphr(color(white) lcolor(white))
saving(gr_b,replace)
             gr combine gr_a.gph gr_b.gph, imargin(zero) graphr(color(white) lcolor(white))
saving(gr_age_`l',replace)

* Next age group
             restore
       }
       gr combine gr_age_39.gph gr_age_49.gph gr_age_59.gph gr_age_69.gph gr_age_70.gph, cols(1)
imargin(zero) graphr(color(white) lcolor(white)) xsize(8.5) ysize(11.5) scale(*.7)
saving(gr_age_cif,replace)

* Get SIR by year x
       foreach var of varlist nonbc cont {
             foreach l of local levels {
                    replace sir_`var'=. if `facv'==`l'
                    replace sir_`var'_ll=. if `facv'==`l'
                    replace sir_`var'_ul=. if `facv'==`l'
                    replace p_`var'=. if `facv'==`l'
                    forval t=2/29 {
                          qui poisson `var' if exp_`var'>0 & `facv'==`l' & yrsdiag <=`t',
exp(exp_`var')
                          replace sir_`var'=exp(_b[_cons]) if `facv'==`l' & yrsdiag ==`t'
                          replace sir_`var'_ll=exp(_b[_cons]-`lvl'*_se[_cons]) if `facv'==`l' &
yrsdiag ==`t'
                          replace sir_`var'_ul=exp(_b[_cons]+`lvl'*_se[_cons]) if `facv'==`l' &
yrsdiag ==`t'
                          replace p_`var'=2*(1-normprob(abs(_b[_cons]/_se[_cons]))) if
`facv'==`l' & yrsdiag ==`t'
                    }
             }
       }
* Display summary, first workout excess
       capture drop nnt* ex_sir*
       gen nnt_nonbc=.
       gen nnt_cont=.
       gen ex_sir_nonbc=.
       gen ex_sir_cont=.
       foreach var of varlist nonbc cont {
             replace nnt_`var'=100/(cif_`var'-cif_exp_`var')
             replace nnt_`var'=round(nnt_`var',1)
             replace ex_sir_`var' = sir_`var'-1
```

```
		replace ex_sir_`var'=round(ex_sir_`var',.01)
	}
	format cif* %4.1f
	sort cage yrsdiag
	noi list `facv' yrsdiag cif_nonbc nnt_nonbc sir_nonbc cif_cont nnt_cont sir_cont if
mod(yrsdiag,5)==0 & yrsdiag>5,  noobs clean

}


***************
* Logical end *
***************
exit

*********************************
* Some number checking routines *
*********************************
* Do a listing
keep `facv' cif_nonbc cif_exp_nonbc cif_cont cif_exp_cont p_nonbc p_cont sir_nonbc sir_cont
rena cif_nonbc  risk1
rena cif_exp_nonbc  risk2
rena sir_nonbc risk3
rena p_nonbc risk4
rena cif_cont   risk5
rena cif_exp_cont   risk6
rena sir_cont risk7
rena p_cont risk8
foreach var of varlist risk1 risk2 risk3 risk5 risk6 risk7 {
	if "`var'"=="risk3" | "`var'"=="risk7" {
		replace `var'=round(`var',0.01)
	}
	else {
		replace `var'=round(`var',0.1)
	}
}



reshape long risk, i(`facv') j(v)
lab def v 1 "NonBCa" 2 "Popln" 3 "SIR" 4 "p-diff" 5 "Contra" 6 "Popln" 7 "SIR" 8 "p-diff",modify
lab val v v
reshape wide risk, i(v) j(`facv')
sort v
noi list v risk*, clean noobs
```

```
* Procedure: Figure_3.do <agegrp>
*
* Computes cumulative incidence for all available cancer groupings
* Competing risks are second cancer event and death
*
*      agegrp: 0 = all ages
*              1 = <60 years at BCa diagnosis
*             2 = 60+ years at BCa diagnosis
*
* User input: "0" produces figure 3
*
* Calls var_cif.do (written by PMcG) to calculate variances of CIF estimates
*
* Uses Stata user written routine to export graphs to Powerpoint: graphs2ppt.ado
* Available here: https://www.statalist.org/forums/forum/general-stata-
discussion/general/1424046-exporting-to-power-point
*
* PMcG: May 2024

*************
* Constants *
*************
* 95% CI
	local lvl=invnorm(1-(1-95/100)/2)

* Censoring date (set this up so can use most of 2021, in the cohort 2nd ca FU ends 31st Oct
2021)
	local cendat=date("31/10/2021","DMY")

* What calendar period to drop out
	local drp_yr=2022

**************
* User input *
**************

* Age at BCa diagnosis to analyse
	local agegrp "`1'"
	if ~("`agegrp'"=="1" | "`agegrp'"=="2")  local agegrp=0



set li 255
**************************************
* Breast cancer cohort analysis file *
**************************************
clear
use <<BCa Cohort file>>


capture drop rt
*gen rt=(surg_rt==1 | surg_rt==3)
*lab def rt 0 "No RT" 1 "RT", modify
gen rt=(surg_rt==1  | surg_rt==2 | surg_rt==3)
lab def rt 0 "No RT" 1 "RT/BCS", modify
lab val rt rt
lab var rt "Radiotherapy"
gen bcs=(surg_rt==1 | surg_rt==2)
lab def bcs 0 "Mast" 1 "BCS", modify
lab val bcs bcs
lab var bcs "Surgery (BCS vs Mast)"
*keep if bcs==0

* Age range to analyse
if `agegrp'==1 drop if age>=60
if `agegrp'==2 keep if age>=60

* Minimise file
drop *nmal* morph* cod*

* Drop out some cancers
foreach var of varlist ca_dg* {
	local lbl : variable label `var'
	if "`lbl'" == "Lung cancer (ipsi.)" drop `var'
	if "`lbl'" == "Lung cancer (cont.)" drop `var'
```

```
       if "`lbl'" == "Lung cancer (unk.)" drop `var'
       if "`lbl'" == "Lung (ipsi.)" drop `var'
       if "`lbl'" == "Lung (cont.)" drop `var'
       if "`lbl'" == "Lung (unk.)" drop `var'
*      if "`lbl'" == "Invasive breast (ipsi.)" drop `var'
* For contralaterals use the cont./unk. variable
       if "`lbl'" == "Invasive breast (cont.)" drop `var'
       if "`lbl'" == "Invasive breast (unk.)" drop `var'

*      if "`lbl'" == "Carcinoma in situ of breast (ipsi.)" drop `var'
*      if "`lbl'" == "Carcinoma in situ of breast (cont.)" drop `var'
*      if "`lbl'" == "Carcinoma in situ of breast (unk.)" drop `var'

}

* Note if had second cancer
capture drop had2ca
gen had2ca=0
lab var had2ca "Had a second cancer"
foreach var of varlist ca_dg* {
       replace had2ca =1 if `var'==1
}

* Sort out deaths in relation to 2nd cancer so can use as competing risk ie ignore death
* after 2nd cancer
* Cancers only go to `cendat'
replace dead=0 if dead==1 & type_2ca<15
replace dead=0 if dead==1 & datlsn>`cendat'

* Use date of second cancer (index 1)
gen datdiag_2ca=datdiag_2ca1
replace datdiag_2ca=datlsn if datdiag_2ca==.
gen datexit=datdiag_2ca
replace datexit=`cendat' if datdiag_2ca>`cendat'

* Setup flag for events cancer and for competing risks (death)
capture drop failtyp
gen failtyp=0
* All cancers
replace failtyp=1 if had2ca==1
* Death
replace failtyp=2 if dead==1
lab var failtyp "Failure type"
lab def failtyp 0 "Censored" 1 "Cancer" 2 "Death", modify
lab val failtyp failtyp

gen canc=(failtyp==1)
gen other=(failtyp==2)
gen any=(failtyp>0)
lab var canc "Second cancer"
lab var other "Death"
lab var any "Any event"

stset datdiag_2ca, id(anon_patient) ori(time datdiag) enter(datatrisk) exit(time datexit)
fail(any) scale(365.25)
drop if _st==0

stsplit yrsdiag, at(0(1)max)
lab val yrsdiag yrsdiag
lab var yrsdiag "Time since breast cancer diagnosis (years)"

* Split by period, age, FU, so can get expecteds
stsplit year=-21914, at(93(1)123)
replace year=year+1900
stsplit age=datob, at(0(5)90)

* Note bin width (sensitive to age)
* Get actual bin width
egen min_t0=min(_t0), by(yrsdiag)
egen max_t=max(_t), by(yrsdiag)
gen w=max_t-min_t0

* Save widths to a file (possible could clash with another do file so
* give an exotic name).  Make sensitive to age.
preserve
```

```
keep yrsdiag w
bysort yrsdiag: keep if _n==1
compress
sort  yrsdiag
save crcaa_fu_width,replace
restore

* Drop events after specific calendar period
drop if year>=`drp_yr'

* Popln data group ages after 85, drop out age>=85
drop if age>=85

* Update w-yrs and outcomes
gen pyrs=_t-_t0
replace any = _d

foreach var of varlist ca_dg* had2ca canc other {
      replace `var'=0 if any==0
}


* Match codes to pop. rates file
replace age=age+4
lab var age "Attained age (years)"
lab def agefmt  4 "0-4" 9 "5-9" 14 "10-14" 19 "15-19" 24 "20-24" 29 "25-29" 34 "30-34" 39 "35-
39" 44 "40-44" 49 "45-49" 54 "50-54" 59 "55-59" 64 "60-64" 69 "65-69" 74 "70-74" 79 "75-79" 84
"80-84" 89 "85-89" 90 "90+", modify
lab val age agefmt
* Drop out ages <20 and ages >84
drop if age<24 | age>84

lab var year "Current calendar year"
compress
save tmp,replace

* Prepare for CIF analysis
use tmp,clear

noi di "Now doing CIFs"
local glist=""
graph drop _all
local j=1
foreach var of varlist ca_dg*  {
* Check got some data for cancer
      summ `var'
      if r(sum) ==0 continue
      preserve
      replace `var'=0 if had2ca==0
      local lbl : variable label `var'
      local dg_num=substr("`var'", 6,.)

* If looking at contralateral etc, multiply the expecteds by 0.5
      local mult=1
      if "`lbl'" == "Breast cancer (ipsi.)" local mult=0.5
      if "`lbl'" == "Breast cancer (cont.)" local mult=0.5
      if "`lbl'" == "Invasive breast (ipsi.)" local mult=0.5
      if "`lbl'" == "Invasive breast (cont.)" local mult=0.5
      if "`lbl'" == "Invasive breast (cont./unk.)" local mult=0.5
      if "`lbl'" == "Carcinoma in situ of breast (ipsi.)" local mult=0.5
      if "`lbl'" == "Carcinoma in situ of breast (cont.)" local mult=0.5
      if "`lbl'" == "Carcinoma in situ of breast (cont./unk.)" local mult=0.5

* For plotting range, 0-5 for all except all cancers & breast
      local ymax=5
      if index("`lbl'", "Malignant neoplasms")>0 local ymax=20
      if index("`lbl'", "All")>0 local ymax=20
      if index("`lbl'", "breast")>0 local ymax=20


* Merge in popln counts and get expected numbers
      collapse (sum) `var' any pyrs, by(year age imd yrsdiag)

* Rectangularise data for merge
      fillin year age imd yrsdiag
```

```
       replace `var'=0 if _fillin==1
       replace any=0 if _fillin==1
       replace pyrs=0 if _fillin==1
       drop _fillin
       sort year age imd
       merge m:1 year age imd using <<population rates file for specified cancer>>
       drop if age<24 | age>84
       drop if year<1993 | year>=`drp_yr'
       drop if _merge==2

* Group up younger ages
*      recode age 4 9 14 19 24 29 34 =39
*      lab def age 39 "<40", modify

       collapse (sum) `var' any pop_`var' pyrs pop, by(year age imd yrsdiag)
       gen pop_`var'_rate=pop_`var'/pop
       recode pop_`var'_rate .=0
       drop pop pop_`var'

* Calculate expecteds (might need to multiply by 1/2 if contralateral)
       gen exp =`mult'*pyrs*pop_`var'_rate
       lab var exp  "Expected cancers"

* Now sum by follow-up year and event type
       collapse (sum)      `var' any exp pyrs, by(yrsdiag)

* Merge in widths of FU windows
       sort yrsdiag
       merge m:1 yrsdiag using crcaa_fu_width
       drop if _merge==2
       noi assert _merge==3 | (_merge==1 & pyrs==0)
       drop _merge

* Now start to work on cumulative risks

* First need to get S for any event and then lag by 1 year so get get KM adjusted
* for competing risks
       sort yrsdiag
       gen crate=sum(w*any/pyrs)
       gen S_any=exp(-crate)
       gen F_any=1-S_any
       sort yrsdiag
       gen S_any_lag=S_any[_n-1]
       replace S_any_lag=1 if yrsdiag==0
       drop crate

* Work out KM (& SE) for each failure type
       foreach kmvar of varlist `var' exp {
              sort yrsdiag
* KM
              gen cif_`kmvar'=sum(S_any_lag*(1-exp(-w*`kmvar'/pyrs)))
* SE
* Also do PMCGs method just as a check
              sort yrsdiag
              gen t_se_cif_`kmvar'=sum(S_any_lag*S_any_lag*(exp(-w*`kmvar'/pyrs)*exp(-
w*`kmvar'/pyrs))*w*w*`kmvar'/(pyrs*pyrs))
              replace t_se_cif_`kmvar'=100*sqrt(t_se_cif_`kmvar')
              noi run var_cif yrsdiag w "`kmvar'" any pyrs "se_cif_`kmvar'"
* CIs
              gen cif_`kmvar'_ll =100*(cif_`kmvar'^(exp(-
`lvl'*se_cif_`kmvar'/(cif_`kmvar'*log(cif_`kmvar')))))
              gen cif_`kmvar'_ul
=100*(cif_`kmvar'^(exp(`lvl'*se_cif_`kmvar'/(cif_`kmvar'*log(cif_`kmvar')))))
              replace cif_`kmvar'=100*cif_`kmvar'
              replace se_cif_`kmvar'=100*se_cif_`kmvar'
       }

*noi list yrsdiag t_se_cif_`var' se_cif_`var'  if mod(yrsdiag,5)==0, noobs clean

*P for difference in risk (%'s)
       gen cif_diff=(cif_`var'-cif_exp)
*      gen se_cif_diff=sqrt((se_cif_`var')^2 + (se_cif_exp)^2)
* Take expected to be known without error
       gen se_cif_diff=se_cif_`var'
       gen cif_diff_ll=cif_diff - `lvl'*se_cif_diff
```

```
      gen cif_diff_ul=cif_diff + `lvl'*se_cif_diff
      gen z=abs(cif_diff)/se_cif_diff
      gen p=2*(1-normprob(z))

* Use SIR to represent difference, get SIR for each period
      local facv="cyrsdiag"
      capture drop cyrsdiag
      gen cyrsdiag=0 if yrsdiag>=0 & yrsdiag<=4
      replace cyrsdiag=1 if yrsdiag>=5 & yrsdiag<=9
      replace cyrsdiag=2 if yrsdiag>=10 & yrsdiag<=19
      replace cyrsdiag=3 if yrsdiag>=20 & yrsdiag~=.
      lab var cyrsdiag "Time since breast cancer diagnosis (years)"
      lab def cyrsdiag 0 "3m-4" 1 "5-9" 2 "10-19" 3 "20-29",modify
      lab val cyrsdiag cyrsdiag

      levelsof `facv', local(levels)
      gen sir_`var'=.
      gen sir_`var'_ll=.
      gen sir_`var'_ul=.
      gen p_`var'=.
      egen t_`var'=sum(`var'), by(`facv')
      local i=1
      local r_`var'0="Time  SIR (95% CI)"
      local  r_`var'99=" "
      foreach lev of local levels {
* Setup a record to use as the origin on the plots
            local nobs=_N
            local nobs=`nobs'+1
            if `i'==1 {
                  set obs `nobs'
                  replace yrsdiag=-1 if _n==`nobs'
            }
            local r_`var'`i'=""
            local sir=""
            local sir_ll=""
            local sir_ul=""
            local lv_lab : label (`facv') `lev'
            capture poisson `var' if exp>0 & `facv'==`lev', exp(exp)
            if _rc==0 {
                  replace sir_`var'=exp(_b[_cons]) if `facv'==`lev'
                  replace sir_`var'_ll=exp(_b[_cons]-`lvl'*_se[_cons]) if `facv'==`lev'
                  replace sir_`var'_ul=exp(_b[_cons]+`lvl'*_se[_cons]) if `facv'==`lev'
                  replace p_`var'=2*(1-normprob(abs(_b[_cons]/_se[_cons]))) if `facv'==`lev'
            }
* Hold summary result for plots
            summ sir_`var' if `facv'==`lev'
            local sir=r(mean)
            summ sir_`var'_ll if `facv'==`lev'
            local sir_ll=r(mean)
            summ sir_`var'_ul if `facv'==`lev'
            local sir_ul=r(mean)
            summ cif_`var' if `facv'==`lev' & yrsdiag==9
            local cif=r(mean)
            summ cif_`var'_ll if `facv'==`lev' & yrsdiag==9
            local cif_ll=r(mean)
            summ cif_`var'_ul if `facv'==`lev' & yrsdiag==9
            local cif_ul=r(mean)
            local r_`var'`i'="`lv_lab' "+string(`sir',"%5.2f")+"
("+string(`sir_ll',"%5.2f")+","+string(`sir_ul',"%5.2f")+")"

* Add an overal total
            if `lev'==3 {
                  poisson `var' if exp>0 , exp(exp)
                  replace sir_`var'=exp(_b[_cons])
                  replace sir_`var'_ll=exp(_b[_cons]-`lvl'*_se[_cons])
                  replace sir_`var'_ul=exp(_b[_cons]+`lvl'*_se[_cons])
                  replace p_`var'=2*(1-normprob(abs(_b[_cons]/_se[_cons])))
* For AER
                  local sir_aer_ll=.
                  local sir_aer_ul=.
                  local sir_aer_ll=exp(_b[_cons]-`lvl'*_se[_cons])
                  local sir_aer_ul=exp(_b[_cons]+`lvl'*_se[_cons])
                  local sir_aer_ul :  display  %5.2f `sir_aer_ul'
                  local sir_aer_ll :  display  %5.2f `sir_aer_ll'
```

```
* Hold summary result for plots
                    if "`var'"=="canc" local r_`var'5="All "+string(sir_`var',"%5.2f")+"
("+string(sir_`var'_ll,"%5.2f")+","+string(sir_`var'_ul,"%5.2f")+")"
              }

* Next level
              local i=`i'+1
       }


* For listing later on, create O & E and AER
       egen spyr=sum(pyrs)
       summ spyr
       replace spyr=r(mean)
       egen O=sum(`var')
       summ O
       replace O=r(mean)
       egen E=sum(exp)
       summ E
       replace E=r(mean)
       gen aer_`var'=1e4*(O-E)/spyr
       gen aer_`var'_ll=1e4*E*(`sir_aer_ll'-1)/spyr
       gen aer_`var'_ul=1e4*E*(`sir_aer_ul'-1)/spyr
       drop spyr

* Don't plot estimates with pyrs=0
       drop if pyrs==0

       drop if se_cif_`var'==. & yrsdiag>=0
*      drop if cif_`var'>25 & yrsdiag>=0

* Setup vars for origin
       replace yrsdiag=yrsdiag+1
       replace yrsdiag=0.25 if yrsdiag==0
       foreach cvar of varlist cif* {
              recode `cvar' .=0 if yrsdiag==.25
       }
       capture drop idx
       bysort `facv' (yrsdiag) : gen idx=_n
       format sir* %5.2f
*      noi list `facv' sir_`var' sir_`var'_ll sir_`var'_ul if idx==2,clean noobs
       sort yrsdiag
* Some % points to plot
       local pvar "y_can y_ecan pcan pecan "
       foreach v of local pvar {
              capture drop `v'
              if substr("`v'",1,1)=="p" {
                     gen `v'=""
              }
              else {
                     gen `v'=.
              }
       }
       capture drop scale
       gen scale=1.2
       replace scale=1.1 if yrsdiag>10
       forvalues x=5 10 to 25 {

* Obsvd
              summ cif_`var' if yrsdiag==`x'
              local can=round(r(mean),.1)
              replace y_can=`can'*scale if yrsdiag==`x'
              replace pcan=string(`can',"%3.1f") if yrsdiag==`x'

* Exp
              summ cif_exp if yrsdiag==`x'
              local ecan=round(r(mean),.1)
              replace y_ecan=`ecan'*1/scale if yrsdiag==`x'
              replace pecan=string(`ecan',"%3.1f") if yrsdiag==`x'
       }
       sort yrsdiag
       if `ymax'==5 twoway rarea cif_`var'_ll cif_`var'_ul yrsdiag, lc(navy) || scatter y_can
y_ecan   yrsdiag, ms(i i) mlabpos(0 0) mlabsiz(*.9 *.9) mla(pcan pecan) mlabc(navy navy)   ||
scatter cif_`var' cif_exp yrsdiag, recast(line) lp(solid dash) lc(navy navy) ylab(0(1)5,nogrid
ang(0)) xlab(0(10)30) yt("Cumulative risk % (95% CI)", siz(*1)) legend(cols(1) ring(0) pos(9)
```

```
symx(*.3) size(*.5) keygap(*.1) region(style(none)))  text(4 5 "{bf:`r_`var'0'}"  "`r_`var'1'"
"`r_`var'2'" "`r_`var'3'" "`r_`var'4'" "`r_`var'5'", j(left) siz(*.8)) ti("`lbl'") legend(off)
graphr(color(white) lcolor(white)) name(`var')
       if `ymax'==20 twoway rarea cif_`var'_ll cif_`var'_ul yrsdiag, lc(navy) || scatter y_can
y_ecan   yrsdiag, ms(i i) mlabpos(0 0) mlabsiz(*.9 *.9) mla(pcan pecan) mlabc(navy navy)   ||
scatter cif_`var' cif_exp yrsdiag, recast(line) lp(solid dash) lc(navy navy) ylab(0(5)20,nogrid
ang(0)) xlab(0(10)30) yt("Cumulative risk % (95% CI)", siz(*1)) legend(cols(1) ring(0) pos(9)
symx(*.3) size(*.5) keygap(*.1) region(style(none)))  text(19 5 "{bf:`r_`var'0'}"  "`r_`var'1'"
"`r_`var'2'" "`r_`var'3'" "`r_`var'4'" "`r_`var'5'", j(left) siz(*.8)) ti("`lbl'") legend(off)
graphr(color(white) lcolor(white)) name(`var')
       local glist = "`glist' `var'"


* For listing
       keep if mod(yrsdiag,10)==0
       gen dg_num=`dg_num'
       keep dg_num O E sir* cif* yrsdiag aer*
       gen sir=string(sir_`var',"%5.2f")+"
("+string(sir_`var'_ll,"%5.2f")+","+string(sir_`var'_ul,"%5.2f")+")"
       gen aer=string(aer_`var',"%5.2f")+"
("+string(aer_`var'_ll,"%5.2f")+","+string(aer_`var'_ul,"%5.2f")+")"
       gen cif_O=string(cif_`var',"%5.2f")+"
("+string(cif_`var'_ll,"%5.2f")+","+string(cif_`var'_ul,"%5.2f")+")"
* Take for expected to be known without error
*      gen cif_E=string(cif_exp,"%5.2f")+"
("+string(cif_exp_ll,"%5.2f")+","+string(cif_exp_ul,"%5.2f")+")"
       gen cif_E=string(cif_exp,"%5.2f")
       gen cif_d=string(cif_diff,"%5.2f")+"
("+string(cif_diff_ll,"%5.2f")+","+string(cif_diff_ul,"%5.2f")+")"
       drop sir*`var'* cif*`var'* cif*exp* cif*diff* aer*`var'*
       reshape wide cif*, i(dg_num) j(yrsdiag)
       gen can="`lbl'"
       order dg_num can O E sir aer cif_O10 cif_E10 cif_d10 cif_O20 cif_E20 cif_d20
       noi list, clean noobs nohead
       if `j'==1 {
             save ca_incid_cif_age`agegrp',replace
       }
       else {
             append using ca_incid_cif_age`agegrp'
             compress
             sort dg_num
             save ca_incid_cif_age`agegrp',replace
       }


* Next cancer
       restore
       local j=`j'+1
}

* Export to powerpoint
noi di "Exporting to powerpoint"
graphs2ppt `glist' using comp_risk_ca_incid_by_cancer_slides_age`agegrp', replace

* Export to Excel
noi di "Listing to Excel"
use ca_incid_cif_age`agegrp',clear
*replace can="Contralateral" if can=="Breast (cont.)"
replace can="Contralateral" if can=="Invasive breast (cont./unk.)"
replace can="Ipsilateral" if can=="Invasive breast (ipsi.)"
replace can = subinstr(can,"cont.","contralaleral",.)
replace can = subinstr(can,"ipsi.","ipsilaleral",.)
replace can = subinstr(can,"gastrointestinal","GI",.)
*replace can = subinstr(can,"respiratory and intrathoracic","resp. or IT",.)
replace can = subinstr(can,"Tumours of central nervous system (CNS)","CNS tumours",.)
replace can = subinstr(can,"Malignant neoplasm of unspecified sites or multiple sites","Mutliple
or unspecified sites",.)
replace can = subinstr(can,"Malignant neoplasms of all specified sites (excl breast & non-
melanoma skin) and","All specified sites",.)
*replace can = subinstr(can,"Melanoma of skin","Skin",.)

* To make things group on plot move other GI
replace dg_num=99 if dg_num==10

* Super-group cancers
```

```
capture drop grp
gen grp=1 if dg_num>=14 & dg_num<=17
replace grp=2 if dg_num>=21 & dg_num<=25
replace grp=3 if dg_num==11 | dg_num==13
replace grp=4 if dg_num==12
replace grp=5 if (dg_num>=1 & dg_num<=2) | dg_num==19 | dg_num==20
replace grp=6 if (dg_num>=3 & dg_num<=4) | dg_num==7
replace grp=7 if dg_num==5 | dg_num==8
replace grp=8 if dg_num==6
replace grp=9 if dg_num==9  | (dg_num>=35 & dg_num<=36) |  dg_num==99
replace grp=10 if dg_num==18
replace grp=11 if dg_num==26 | dg_num==27 | dg_num==29
replace grp=12 if dg_num==31 | dg_num==34
recode grp .=13

lab def grp 1 "Gynaecological" 2 "Hematopietic/lymphoid" 3 "Bone and soft tissue" 4 "Skin" 5
"Head and neck/endocrine" 6 "Upper gastrointestinal" 7 "Lower or other gastrointestinal" 8
"Liver" 9 "Respiratory" 10 "Central nervous system" 11 "All cancers (excluding breast)" 12
"Breast" 13 "Unclassified",modify
lab val grp grp
lab var grp "Second cancer group"
compress

bysort grp (dg_num) : gen idx=_n
expand 2, gen(cop)
drop if cop==1 & idx>1
replace idx=0 if cop==1
sort grp idx
foreach var of varlist can-cif_d20 {
       capture replace `var'="" if idx==0
       capture replace `var'=. if idx==0
}
replace grp=. if idx>0
export excel grp can O E sir aer cif_O10 cif_E10 cif_d10 cif_O20 cif_E20 cif_d20 using
comp_risk_ca_incid_by_cancer_age`agegrp'.xlsx, replace firstr(varl)
noi list grp can O E sir aer cif_O10 cif_E10 cif_d10 cif_O20 cif_E20 cif_d20, clean noobs

* Scatter plot of CIFs by cancer
noi di "Scatter plot of CIFs by cancer"
use ca_incid_cif_age`agegrp',clear
*replace can="Contralateral" if can=="Breast (cont.)"
replace can="Contralateral" if can=="Invasive breast (cont./unk.)"
replace can="Ipsilateral" if can=="Invasive breast (ipsi.)"
replace can = subinstr(can,"cont.","contralaleral",.)
replace can = subinstr(can,"ipsi.","ipsilaleral",.)
replace can = subinstr(can,"gastrointestinal","GI",.)
*replace can = subinstr(can,"respiratory and intrathoracic","resp. or IT",.)
replace can = subinstr(can,"Tumours of central nervous system (CNS)","CNS tumours",.)
replace can = subinstr(can,"Malignant neoplasm of unspecified sites or multiple sites","Mutliple
or unspecified sites",.)
replace can = subinstr(can,"Malignant neoplasms of all specified sites (excl breast & non-
melanoma skin) and","All specified sites",.)
*replace can = subinstr(can,"Melanoma of skin","Skin",.)

*drop if dg_num==27 | dg_num==28 | dg_num>=30

replace can ="Other respiratory" if can=="Other respiratory and intrathoracic"
replace can="Unspecified" if can=="Mutliple or unspecified sites"
replace can="Other specified" if can=="Other specified sites"
replace can="ALL" if can=="Acute lymphoblastic leukaemia"
replace can="AML" if can=="Acute myeloid leukaemia"
replace can="Other head/neck" if can=="Other head and neck"
replace can="Ovarian" if can=="Ovary or fallopian tubes"
replace can="Other endocrine" if can=="Other endocrine gland"


gen c10o=real(substr(cif_O10,1,4))
gen c10ol=real(substr(cif_O10,7,4))
gen c10ou=real(substr(cif_O10,12,4))
gen c10e=real(substr(cif_E10,1,4))
* Take expected to be known without error
*gen c10el=real(substr(cif_E10,7,4))
*gen c10eu=real(substr(cif_E10,12,4))
gen c10el=c10e
gen c10eu=c10e
```

```
gen c20o=real(substr(cif_O20,1,4))
gen c20ol=real(substr(cif_O20,7,4))
gen c20ou=real(substr(cif_O20,12,4))
gen c20e=real(substr(cif_E20,1,4))
* Take expected to be known without error
*gen c20el=real(substr(cif_E20,7,4))
*gen c20eu=real(substr(cif_E20,12,4))
gen c20el=c20e
gen c20eu=c20e

sum c10ou
local ymx10=round(r(max),.01)
sum c20ou
local ymx20=round(r(max),.1)


*sort dg_num
gsort -c20o
gen dnum=_n
labmask dnum, val(can)
lab var dnum "Type of second cancer"
gen sir_pe=substr(sir,1,4)

* Force y-axis
local ymx20=3.8
if `agegrp'==0 local ymx20=2.8

*twoway rcap c20ol c20ou dnum, lc(navy) || rcap c20el c20eu dnum, lc(maroon) || scatter c20o
c20e dnum, msym(o o) msiz(*.5 *.5) mcol(navy maroon) legend(order(3 "Observed" 4 "Expected")
cols(1) ring(0) pos(1)) xlab(1(1)27, grid ang(-45) val labs(*.5)) ylab(0(0.2)`ymx20',ang(0)
format(%3.1f))  xsize(11.5) ysize(8.5) graphr(color(white) lcolor(white)) yt("Cumulative risk %
(95% CI) at 20-years") saving(ca_incid_cif_yr20_age`agegrp'_v1,replace)
*twoway rcap c20ol c20ou dnum, lc(navy) || rcap c20el c20eu dnum, lc(maroon) || scatter c20o
c20e dnum, msym(o |) msang(0 90) msiz(*.5 *.5) mcol(navy maroon) legend(order(3 "Observed" 4
"Expected") cols(1) ring(0) pos(1)) xlab(1(1)27, grid ang(-30) val labs(*.5))
ylab(0(0.2)`ymx20',ang(0) format(%3.1f))  xsize(11.5) ysize(8.5) graphr(color(white)
lcolor(white)) yt("Cumulative risk % (95% CI) at 20-years")
saving(ca_incid_cif_yr20_age`agegrp'_v1,replace)
twoway rcap c20ol c20ou dnum, lc(navy) || scatter c20o c20e dnum, msym(o |) msang(0 90) msiz(*.5
*1.1) mcol(navy maroon) legend(order(2 "Observed" 3 "Expected") cols(1) ring(0) pos(1))
xlab(1(1)27, grid ang(-30) val labs(*.5)) ylab(0(0.2)`ymx20',ang(0) format(%3.1f))  xsize(11.5)
ysize(8.5) graphr(color(white) lcolor(white)) yt("Cumulative risk % (95% CI) at 20-years")
saving(ca_incid_cif_yr20_age`agegrp'_v1,replace)
gr export ca_incid_cif_yr20_age`agegrp'_v1.pdf,replace
twoway rcap c20ol c20ou dnum, lc(navy) || rcap c20el c20eu dnum, lc(maroon) || scatter c20o c20e
dnum, msym(o o) msiz(*.5 *.5) mcol(navy maroon) mlab(sir_pe) mlabp(12) mlabs(*.5) mlabc(black)
mlabg(*2) legend(order(3 "Observed" 4 "Expected") cols(1) ring(0) pos(1)) xlab(1(1)27, grid
ang(-45) val labs(*.5)) ylab(0(0.2)`ymx20',ang(0) format(%3.1f))  xsize(11.5) ysize(8.5)
graphr(color(white) lcolor(white)) yt("Cumulative risk % (95% CI) at 20-years")
saving(ca_incid_cif_yr20_age`agegrp'_v2,replace)
gr export ca_incid_cif_yr20_age`agegrp'_v2.pdf,replace

* Vertical orientation
twoway rcap c20ol c20ou dnum, lc(navy) horizontal || rcap c20el c20eu dnum, lc(maroon)
horizontal || scatter dnum c20o , msym(o) msiz(*.5) mcol(navy) || scatter dnum c20e , msym(|)
msiz(*.5) mcol(maroon) legend(order(3 "Observed" 4 "Expected") cols(1) ring(0) pos(3))
xlab(0(0.2)`ymx20',labs(*.7) grid ang(0) format(%3.1f)) ylab(1(1)27, grid ang(0) val labs(*.5))
graphr(color(white) lcolor(white)) b1t("Cumulative risk % (95% CI) at 20-years") yscale(reverse)
xsize(9) ysize(9) saving(ca_incid_cif_yr20_age`agegrp'_v3,replace)
twoway rcap c20ol c20ou dnum, lc(navy) horizontal || rcap c20el c20eu dnum, lc(maroon)
horizontal || scatter dnum c20o , msym(o) msiz(*.5) mcol(navy) || scatter dnum c20e , msym(|)
msiz(*.5) mcol(maroon) legend(order(3 "Observed" 4 "Expected") cols(1) ring(0) pos(3))
xlab(0(0.2)`ymx20',labs(*.7) grid ang(0) format(%3.1f)) ylab(1(1)27, grid ang(0) val labs(*.5))
graphr(color(white) lcolor(white)) b1t("Cumulative risk % (95% CI) at 20-years") yscale(reverse)
saving(ca_incid_cif_yr20_age`agegrp'_v3,replace)
gr export ca_incid_cif_yr20_age`agegrp'_v3.pdf,replace


*twoway rcap c10ol c10ou dnum, lc(navy) || rcap c10el c10eu dnum, lc(maroon) || scatter c10o
c10e dnum, msym(o o) msiz(*.5 *.5) mcol(navy maroon) legend(order(3 "Observed" 3 "Expected")
cols(1) ring(0) pos(1)) xlab(1(1)27, grid ang(-45) val labs(*.5)) ylab(0(0.1)`ymx10',ang(0)
format(%3.1f)) graphr(color(white) lcolor(white)) yt("Cumulative risk % (95% CI) at 10-years")
saving(ca_incid_cif_yr10_age`agegrp',replace)
```

```
*gr combine ca_incid_cif_yr10_age`agegrp'.gph ca_incid_cif_yr20_age`agegrp'.gph, cols(1)
xsize(8.5) ysize(11.5) imargin(zero)  graphr(color(white) lcolor(white))

* Square root plt
foreach var of varlist c20* {
       gen sqrt_`var'=sqrt(`var')
}

* Labels
local label=""
foreach n of num 0(0.2)2.8 {
       local label `label' `= sqrt(`n')' "`n'"
}
*twoway rcap sqrt_c20ol sqrt_c20ou dnum, lc(navy) || rcap sqrt_c20el sqrt_c20eu dnum, lc(maroon)
|| scatter sqrt_c20o sqrt_c20e dnum, msym(o o) msiz(*.5 *.5) mcol(navy maroon) legend(order(3
"Observed" 4 "Expected") cols(1) ring(0) pos(1)) xlab(1(1)27, grid ang(-45) val labs(*.5))
ylab(`label',ang(0) format(%3.1f)) graphr(color(white) lcolor(white)) yt("Cumulative risk % (95%
CI) at 20-years") saving(ca_incid_cif_yr20_sqrt_age`agegrp',replace)

* Save a version of the data for BMJ
save bmj_fig3_data.dta,replace

***************
* Logical end *
***************
exit

*****************************************************
* Optional extra code to plot scatter graphs of CIFs *
*****************************************************

* Plot CIFs at 20 years for <60 & 60+
noi di "Scatter plot of CIFs
use ca_incid_cif_age1,clear
gen age=1
append using ca_incid_cif_age2
recode age .=2


* Drop totals cats
drop if dg_num==27 | dg_num==28 | dg_num>=30


*replace can="Contralateral" if can=="Breast (cont.)"
replace can="Contralateral" if can=="Invasive breast (cont./unk.)"
replace can = subinstr(can,"cont.","contralaleral",.)
replace can = subinstr(can,"ipsi.","ipsilaleral",.)
replace can = subinstr(can,"gastrointestinal","GI",.)
*replace can = subinstr(can,"respiratory and intrathoracic","resp. or IT",.)
replace can = subinstr(can,"Tumours of central nervous system (CNS)","CNS tumours",.)
replace can = subinstr(can,"Malignant neoplasm of unspecified sites or multiple sites","Mutliple
or unspecified sites",.)
replace can = subinstr(can,"Malignant neoplasms of all specified sites (excl breast & non-
melanoma skin) and","All specified sites",.)
*replace can = subinstr(can,"Melanoma of skin","Skin",.)
replace can ="Other respiratory" if can=="Other respiratory and intrathoracic"
replace can="Unspecified" if can=="Mutliple or unspecified sites"
replace can="Other specified" if can=="Other specified sites"
replace can="ALL" if can=="Acute lymphoblastic leukaemia"
replace can="AML" if can=="Acute myeloid leukaemia"
replace can="Other head/neck" if can=="Other head and neck"
replace can="Ovarian" if can=="Ovary or fallopian tubes"
replace can="Other endocrine" if can=="Other endocrine gland"


gen c20o=real(substr(cif_O20,1,4))
gen c20ol=real(substr(cif_O20,7,4))
gen c20ou=real(substr(cif_O20,12,4))
gen c20e=real(substr(cif_E20,1,4))
gen c20el=real(substr(cif_E20,7,4))
gen c20eu=real(substr(cif_E20,12,4))

drop O E sir sir aer cif_O10 cif_E10 cif_O20 cif_E20

reshape wide c20*, i(dg_num) j(age)
```

```
sum c20ou2
local ymx=round(r(max),.1)
gsort -c20o2
gen dnum=_n
labmask dnum, val(can)
lab var dnum "Type of second cancer"
reshape long
sort dnum

lab var age "Age at BCa diagnosis"
lab def age 1 "Age 18-59 at breast cancer diagnosis" 2 "Age 60-75 at breast cancer
diagnosis",modify
lab val age age

twoway rcap c20ol c20ou dnum, lc(navy) || rcap c20el c20eu dnum, lc(maroon) || scatter c20o c20e
dnum, by(age, ix cols(1) graphr(color(white) lcolor(white))) msym(o o) msiz(*.5 *.5) mcol(navy
maroon)  xlab(1(1)27, grid ang(-45) val labs(*.6)) ylab(0(0.2)`ymx',ang(0) format(%3.1f)
labs(*.8)) legend(order(3 "Observed" 4 "Expected") cols(2) ring(1) pos(11) siz(*.8))
graphr(color(white) lcolor(white)) yt("Cumulative risk % (95% CI) at 20-years") xsize(8)
ysize(13.5)  subtitle(,nobox fc(white)) saving(ca_incid_cif_yr20_age12,replace)
gr export ca_incid_cif_yr20_age12.pdf,replace
```

```
* Procedure: Figure_4.do
*
* Regressions to investigate associations between breast cancer treatments and
* second cancer risk
*
* Uses Stata user written command admetan.ado to produce forest plots
* Available: https://ideas.repec.org/p/boc/usug18/06.html
* Uses Stata user written command labmask.ado for labelling variable values
* Available: http://fmwww.bc.edu/repec/bocode/l/labmask.ado
*
* PMcG: Feb 2024 -first version
* PMcG: Aug 2024: Use colours on plots to compare with expected result from RCTs (needs manual
*                 intervention to always make sure its correct admetan doesn't always handle the
plot
*                 order correctly either, so needs double checking)
* PMcG: Nov 2024: for excess cancers use number in treated groups

* Levels for CI (for admetan to work as expected with CIs one needs the level option set
*                in admetan and also the global macro set)
       local wci=99
       set level `wci'

* Censoring date (set this up so can use most of 2021, in the cohort 2nd ca FU ends 31st Oct
2021)
       local cendat=date("31/10/2021","DMY")

* Arrays for adjusted regressions
* Do adjustments based on model testing of death outcomes
* Strata so can do stratified analysis via xtpoisson regression
       local array "bcs rt sys_hormer sys_chem cage imd yr_diag yrsdiag"
       local adj_array "bcs rt sys_hormer sys_chem ib69.cage i.imd i.yr_diag i.yrsdiag"
       local strata_array "cage imd yr_diag yrsdiag"

* For endocrine as many missclassed as no endo when likely did need to add an
* adjustment factor (apply to uterine and conts only as they have the signif results)
* Factor based RR(3 good cancer registry regions)/RR(all registries)
* Only required for all years as excess cancers not otherwise shown
       local cf_oth=1
       local cf_ut=1.046
       local cf_cont=0.962

**************************************
* Breast cancer cohort analysis file *
**************************************
clear
use <<BCa Cohort file>>

* Drop out uninteresting (ALWAYS CHECK CODES ARE VALID AS CAN CHANGE!!!!)
drop ca_dg28 ca_dg30 ca_dg31 ca_dg32 ca_dg33 ca_dg37

lab def agefmt 39 "20-39", modify

* Make RT BCS vs Mast-RT
capture drop rt
*gen rt=(surg_rt==1 | surg_rt==3)
gen rt=(surg_rt==1  | surg_rt==2 | surg_rt==3)
lab def rt 0 "No RT" 1 "RT/BCS", modify
lab val rt rt
lab var rt "Radiotherapy"
gen bcs=(surg_rt==1 | surg_rt==2)
lab def bcs 0 "Mast" 1 "BCS", modify
lab val bcs bcs
lab var bcs "Surgery (BCS vs Mast)"

lab def sys_horm 0 "NoEndo" 1 "Endo", modify
lab def sys_chem 0 "NoChem" 1 "Chem", modify
egen horm_chem=group(sys_horm sys_chem), lab(horm_chem, replace)

gen sys_hormer=(er_endo==1)
lab def sys_hormer 0 "NoEndo" 1 "Endo/ER+", modify
lab val sys_hormer sys_hormer
lab var sys_hormer "Endocrine therapy"

* Note registries with decent endo recording (defined as 70+%)
gen freq=1
```

```
egen sum_h=sum(sys_hormer), by(creg_name)
egen ptot_reg=sum(freq), by(creg_name)
gen horm_rec_reg=100*sum_h/ptot_reg
gen horm_70pct=horm_rec_reg>=70
drop freq sum_h ptot_reg
* Analyse just the 3 "good" treatment recording registires
local good=0
if `good'==1 {
	noi di "NOTE: analysis running on just the 3 good endo recording registries"
	keep if horm_70pct==1
}

lab def escrn_det 0 "Scrn det" 1 "Not scrn det" 2 "Not elig", modify
lab def er_status_lbl 2 "ER-ve" 3 "ER+ve" 5 "ER?", modify

gen nodpos=(nod_stat>=2 & nod_stat<=4)
replace nodpos=2 if nod_stat==5
lab def nodpos 0 "pN0" 1 "pN+" 2 "pN?"
lab val nodpos nodpos

* For cancer type, group up if less than total number of events <100
* this is manual!!
recode ca_type 4=0
lab def ca_type 0 "Carcinoma NST", modify
recode ca_type 1 2 3 5 6 12 14 17 =19


* Note if had second cancer
replace had2ca=0
lab var had2ca "Had a second primary"
foreach var of varlist ca_dg* {
	replace had2ca =1 if `var'==1
}

* Use date of second cancer (index 1)
gen datdiag_2ca=datdiag_2ca1
replace datdiag_2ca=datlsn if datdiag_2ca==.
gen datexit=datdiag_2ca
replace datexit=`cendat' if datdiag_2ca>`cendat'
stset datdiag_2ca, id(anon_patient) ori(time datdiag) enter(datatrisk) exit(time datexit)
fail(had2ca) scale(365.25)
drop if _st==0

* Use the 70% endo registries to get treatment prevalences
sum bcs if horm_70pct==1
local p_bcs=r(mean)
sum rt if horm_70pct==1
local p_rt=r(mean)
sum sys_hormer if horm_70pct==1
local p_et=r(mean)
sum sys_chem if horm_70pct==1
local p_ct=r(mean)
* Need RT/ET combination for contralateral excess estimates
gen rt_noet=(rt==1 & sys_hormer==0)
sum rt_noet if horm_70pct==1
local p_rt_noet=r(mean)
gen nort_et=(rt==0 & sys_hormer==1)
sum nort_et if horm_70pct==1
local p_nort_et=r(mean)
capture drop rt_noet nort_et

stsplit yrsdiag, at(0(5)25)
lab def yrsdiag 0 "<1-4" 5 "5-9" 10 "10-14" 15 "15-19" 20 "20-24" 25 "25-29", modify
lab val yrsdiag yrsdiag
lab var yrsdiag "Time since diagnosis (years)"

* Update outcome & pyrs
gen pyrs=_t-_t0
replace had2ca=_d

* Update cancer flags
foreach var of varlist ca_dg* {
	replace `var'=0 if had2ca==0
}
*replace diag_2ca_dth=0 if had2ca==0
```

```
replace type_2ca = 15 if had2ca==0
save tmp,replace


local rater=1
if `rater'==1 {
***************
* Rate ratios *
***************

*****Unadjusted rate ratios
use tmp,clear
capture postclose temp
postfile temp str20 trt seccatype str50 calab ncases prev cf logrr selogrr using ca_rr_unadj,
replace
foreach ca of varlist ca_dg* {
	local ca_num=substr("`ca'", 6,.)
	local calab: variable label `ca'
	noi di "ca: `ca' [`calab']"
	summ `ca' if `ca'==1
	local ncases=r(N)
	preserve
	collapse (sum) `ca' pyrs, by(rt sys_hormer sys_chem)
	foreach var of varlist rt sys_hormer sys_chem {
		noi poisson `ca' `var', nolog exp(pyrs)
		local logrr=_coef[`var']
		local selogrr=_se[`var']
* Setup treatment prevlances
		if "`var'"=="bcs" local prev=`p_bcs'
		if "`var'"=="rt" local prev=`p_rt'
		if "`var'"=="sys_hormer" local prev=`p_et'
		if "`var'"=="sys_chem" local prev=`p_ct'
* For contralaterals
		if "`var'"=="rt" & trim("`calab'")=="Invasive breast (cont./unk.)" local
prev=`p_rt_noet'
		if "`var'"=="sys_hormer" & trim("`calab'")=="Invasive breast (cont./unk.)" local
prev=`p_nort_et'
* Correction factor for missclassification
		local cf=`cf_oth'
		if "`var'"=="sys_hormer" & trim("`calab'")=="Uterine" local cf=`cf_ut'
		if "`var'"=="sys_hormer" & trim("`calab'")=="Invasive breast (cont./unk.)" local
cf=`cf_cont'
		post temp ("`var'") (`ca_num') ("`calab'") (`ncases') (`prev') (`cf') (`logrr')
(`selogrr')
	}
	restore
}
capture postclose temp

* Plot
use ca_rr_unadj, clear
compress

gen p=2*(1-normprob(abs(logrr/selogrr)))
gen sig="*" if p<0.01 & logrr>0
replace sig="*" if p<0.01 & trim(calab)=="Invasive breast (cont./unk.)" &
trim(trt)=="sys_hormer"
replace sig="" if trim(calab)=="Malignant neoplasms of all specified sites (excl b"
lab var sig "RR>1 & p<0.01"
gen rr=cf*exp(logrr)
gen rrlt1=rr<1 & sig=="*"
replace rr=1/rr if sig=="*" & rr<1
gen tmp=ncases*prev*(rr-1)/rr if sig=="*"
replace tmp=round(tmp,5)
gen ex_ca=string(tmp)
replace ex_ca="-"+ex_ca if rrlt1==1
replace ex_ca="" if ex_ca=="."
lab var ex_ca "Excess cancers"

lab var calab " "
*replace calab="Contralateral" if calab=="Breast (cont.)"
replace calab="Contralateral" if calab=="Invasive breast (cont./unk.)"
replace calab = subinstr(calab,"cont.","contralaleral",.)
replace calab = subinstr(calab,"ipsi.","ipsilaleral",.)
replace calab = subinstr(calab,"gastrointestinal","GI",.)
```

```
*replace calab = subinstr(calab,"respiratory and intrathoracic","resp. or IT",.)
replace calab = subinstr(calab,"Tumours of central nervous system (CNS)","CNS tumours",.)
replace calab = subinstr(calab,"Malignant neoplasm of unspecified sites or multipl","Mutliple or unspecified sites",.)
replace calab = subinstr(calab,"Malignant neoplasms of all specified sites (excl b","All specified sites",.)
*replace calab = subinstr(calab,"Melanoma of skin","Skin",.)

* To make things group on plot move other GI
replace seccatype=99 if seccatype==10

lab var ncases "# cancers"
lab var seccatyp "Type of second cancer"
labmask seccatyp, value(calab)

* Setup colours for points noting which endpoints have signif RR from RCTs
gen rand_ex = 1  if (trt=="rt" & calab=="Contralateral")
replace rand_ex = 1  if (trt=="rt" & index(upper(calab),"LEUKAEMIA"))
replace rand_ex = 1  if (trt=="rt" & calab=="Lung")
replace rand_ex = 1  if (trt=="rt" & calab=="Lung (ipsilaleral)")
replace rand_ex = 1  if (trt=="rt" & calab=="Oesophagus")
replace rand_ex = 1  if (trt=="sys_hormer" & calab=="Contralateral")
replace rand_ex = 1  if (trt=="sys_hormer" & calab=="Uterine")
replace rand_ex = 1  if (trt=="sys_chem" & calab=="Acute myeloid leukaemia")
gen plt=1
replace plt=2 if sig=="*"
replace plt=3 if plt==2 & rand_ex==1

lab def plt 1 "Uninteresting" 2 "Signif, no RCT" 3 "Signif, RCT", modify
lab val plt plt
lab var plt "Plot colour"

* White for unintersting, grey for signif but not seen in RCTS, black for signif and seen in RCTs
* pc1 is colour of the CI for plt=1
local pc1="gs0"
local pc2="gs10"
local pc3="gs0"

noi tab trt plt, miss
* admetan can't handle gaps in "plt", so need to do some setting up
* RT has plt vals 1 & 2, horm has 1 & 3, CT has 1, 2, 3
*local rt_opt=" ci1opt(lc(`pc1') lw(vthin) msize(vsmall)) point1opt(msymbol(S) msize(vsmall) mfcol(gs16) mcol(gs16) mlcol(gs0) mlw(vthin)) ci2opt(lc(`pc2') lw(vthin) msize(vsmall)) point2opt(msymbol(S) msize(vsmall) mcol(`pc2')) ci3opt(lc(`pc3') lw(vthin) msize(vsmall)) point3opt(msymbol(S) msize(vsmall) mcol(`pc3'))"
local rt_opt=" ci1opt(lc(`pc1') lw(vthin) msize(vsmall)) point1opt(msymbol(S) msize(vsmall) mfcol(gs16) mcol(gs16) mlcol(gs0) mlw(vthin)) ci2opt(lc(`pc2') lw(vthin) msize(vsmall)) point2opt(msymbol(S) msize(vsmall) mcol(`pc2'))"
*local et_opt=" ci1opt(lc(`pc1') lw(vthin) msize(vsmall)) point1opt(msymbol(S) msize(vsmall) mfcol(gs16) mcol(gs16) mlcol(gs0) mlw(vthin)) ci2opt(lc(`pc2') lw(vthin) msize(vsmall)) point2opt(msymbol(S) msize(vsmall) mcol(`pc2')) ci3opt(lc(`pc3') lw(vthin) msize(vsmall)) point3opt(msymbol(S) msize(vsmall) mcol(`pc3'))"
* For some reason admetan won't accept the plot order endo, so kludge it here
local et_opt=" ci1opt(lc(`pc1') lw(vthin) msize(vsmall)) point1opt(msymbol(S) msize(vsmall) mfcol(gs16) mcol(gs16) mlcol(gs0) mlw(vthin)) ci2opt(lc(`pc3') lw(vthin) msize(vsmall)) point2opt(msymbol(S) msize(vsmall) mcol(`pc3')) ci3opt(lc(`pc2') lw(vthin) msize(vsmall)) point3opt(msymbol(S) msize(vsmall) mcol(`pc2'))"
local ct_opt=" ci1opt(lc(`pc1') lw(vthin) msize(vsmall)) point1opt(msymbol(S) msize(vsmall) mfcol(gs16) mcol(gs16) mlcol(gs0) mlw(vthin)) ci2opt(lc(`pc2') lw(vthin) msize(vsmall)) point2opt(msymbol(S) msize(vsmall) mcol(`pc2')) ci3opt(lc(`pc3') lw(vthin) msize(vsmall)) point3opt(msymbol(S) msize(vsmall) mcol(`pc3'))"

* Super-group cancers (ALWAYS CHECK CODES ARE VALID AS CAN CHANGE!!!!)
capture drop grp
gen grp=1 if seccatyp>=14 & seccatyp<=17
replace grp=2 if seccatyp>=21 & seccatyp<=25
replace grp=3 if seccatyp==11 | seccatyp==13
replace grp=4 if seccatyp==12
replace grp=5 if (seccatyp>=1 & seccatyp<=2) | seccatyp==19 | seccatyp==20
replace grp=6 if (seccatyp>=3 & seccatyp<=4) | seccatyp==7
replace grp=7 if seccatyp==5 | seccatyp==8
replace grp=8 if seccatyp==6
replace grp=9 if seccatyp==9  | (seccatyp>=35 & seccatyp<=36) |  seccatyp==99
replace grp=10 if seccatyp==18
```

```
replace grp=11 if seccatyp==26 | seccatyp==27 | seccatyp==29
replace grp=12 if seccatyp==34
recode grp .=13

lab def grp 1 "{bf:Gynaecological}" 2 "{bf:Hematopietic/lymphoid}" 3 "{bf:Bone and soft tissue}"
4 "{bf:Skin}" 5 "{bf:Head and neck/endocrine}" 6 "{bf:Upper gastrointestinal}" 7 "{bf:Lower or
other gastrointestinal}" 8 "{bf:Liver}" 9 "{bf:Respiratory}" 10 "{bf:Central nervous system}" 11
"{bf:All cancers (excluding breast)}" 12 "{bf:Breast}" 13 "{bf:Unclassified}",modify
lab val grp grp
lab var grp "Second cancer group"
compress
format ncases %-5.0f
gen sncases=string(ncases,"%-5.0f")
lab var ncases "Number_of cancers"
lab var sncases "Number_of cancers"


* Forest
* Do a version in admetan that will place the RRs beside each other and just have the cancer
description once
* Buildup multiplot
* Don't show excess # cancers on these plots
* Ca names & # cases
local scale = "0.5 1.0 1.5 2.0 2.5"
admetan logrr selogrr if trt=="rt", by(grp) sortby(seccatype) study(calab) lcols(sncases) eform
level(`wci') nowt keepa keepord forestplot( title("Type of second cancer", size(tiny) c(black))
graphr(color(white)) nooverall nosubgroup nostats nowt nohet nowarn colsonly
name(cancers,replace) savedims(A))
* RRs for RT
admetan logrr selogrr if trt=="rt",   by(grp)    sortby(seccatype) effect(Unadjusted rate ratio)
study(calab) eform level(`wci') nowt keepa keepord forestplot(xlab(`scale', force)
title("Radiotherapy (or BCS) (recorded vs not)", size(tiny) c(black))        plotid(plt)
`rt_opt' diamopt(lc(black) lw(thin)) olineopt(lc(black) lp(dash) lw(thin)) astext(50)
graphr(color(white)) null(1) leftj nobox nooverall nosubgroup nonames nowarn name(rt,replace)
usedims(A) )
* RRs for endocrine
admetan logrr selogrr if trt=="sys_hormer", by(grp) sortby(seccatype) effect(Unadjusted rate
ratio) study(calab) eform level(`wci') nowt keepa keepord forestplot(xlab(`scale', force)
title("Endocrine therapy (or ER+ve) (recorded vs not)", size(tiny) c(black)) plotid(plt)
`et_opt' diamopt(lc(black) lw(thin)) olineopt(lc(black) lp(dash) lw(thin))astext(50)
graphr(color(white)) null(1) leftj nobox nooverall nosubgroup nonames nowarn name(endo,replace)
usedims(A) )
* RRs for chemo
admetan logrr selogrr if trt=="sys_chem", by(grp) sortby(seccatype) effect(Unadjusted rate
ratio) study(calab) eform level(`wci') nowt keepa keepord forestplot(xlab(`scale', force)
title("Chemotherapy (recorded vs not)", size(tiny) c(black))     plotid(plt)  `ct_opt'
diamopt(lc(black) lw(thin)) olineopt(lc(black) lp(dash) lw(thin)) astext(50)
graphr(color(white)) null(1) leftj nobox nooverall nosubgroup nonames nowarn name(chemo,replace)
usedims(A) )
* Combine
graph combine cancers rt endo chemo, rows(1) scale(*1.9) imargin(zero) graphr(color(white)
margin(zero) lcolor(white)) xsize(11.5) ysize(6)
saving(gr_trt_rater_2ndca_unadj_fp_comb,replace)
gr export gr_trt_rater_2ndca_unadj_fp_comb.pdf,replace

*****adjusted rate ratios
use tmp,clear
capture postclose temp
postfile temp str20 trt seccatype str50 calab ncases prev cf logrr selogrr using ca_rr_adj,
replace
foreach ca of varlist ca_dg* {
       local ca_num=substr("`ca'", 6,.)
       local calab: variable label `ca'
       noi di "ca: `ca' [`calab']"
       summ `ca' if `ca'==1
       local ncases=r(N)
       local ca_trt="bcs rt sys_hormer sys_chem"
       preserve
       collapse (sum) `ca' pyrs, by(`array')
*      noi poisson `ca'  `adj_array', nolog exp(pyrs)
       egen strata=group(`strata_array'),lab
       xtset strata
       noi xtpoisson `ca' `ca_trt', nolog exp(pyrs) irr fe
       foreach var of local ca_trt {
              local logrr=_coef[`var']
```

```
              local selogrr=_se[`var']
* Setup treatment prevlances
              if "`var'"=="bcs" local prev=`p_bcs'
              if "`var'"=="rt" local prev=`p_rt'
              if "`var'"=="sys_hormer" local prev=`p_et'
              if "`var'"=="sys_chem" local prev=`p_ct'
* For contralaterals
              if "`var'"=="rt" & trim("`calab'")=="Invasive breast (cont./unk.)" local
prev=`p_rt_noet'
              if "`var'"=="sys_hormer" & trim("`calab'")=="Invasive breast (cont./unk.)" local
prev=`p_nort_et'
* Correction factor for missclassification
              local cf=`cf_oth'
              if "`var'"=="sys_hormer" & trim("`calab'")=="Uterine" local cf=`cf_ut'
              if "`var'"=="sys_hormer" & trim("`calab'")=="Invasive breast (cont./unk.)" local
cf=`cf_cont'
              post temp ("`var'") (`ca_num') ("`calab'") (`ncases') (`prev') (`cf') (`logrr')
(`selogrr')
       }
       restore
}
capture postclose temp

* Plot
use ca_rr_adj, clear
compress

gen p=2*(1-normprob(abs(logrr/selogrr)))
gen sig="*" if p<0.01 & logrr>0
replace sig="*" if p<0.01 & trim(calab)=="Invasive breast (cont./unk.)" &
trim(trt)=="sys_hormer"
replace sig="" if trim(calab)=="Malignant neoplasms of all specified sites (excl b"
lab var sig "RR>1 & p<0.01"
gen rr=cf*exp(logrr)
gen rrlt1=rr<1 & sig=="*"
replace rr=1/rr if sig=="*" & rr<1
gen tmp=ncases*prev*(rr-1)/rr if sig=="*"
replace tmp=round(tmp,5)
gen ex_ca=string(tmp)
replace ex_ca="-"+ex_ca if rrlt1==1
replace ex_ca="" if ex_ca=="."
lab var ex_ca "Excess cancers"


lab var calab " "
*replace calab="Contralateral" if calab=="Breast (cont.)"
replace calab="Contralateral" if calab=="Invasive breast (cont./unk.)"
replace calab = subinstr(calab,"cont.","contralaleral",.)
replace calab = subinstr(calab,"ipsi.","ipsilaleral",.)
replace calab = subinstr(calab,"gastrointestinal","GI",.)
*replace calab = subinstr(calab,"respiratory and intrathoracic","resp. or IT",.)
replace calab = subinstr(calab,"Tumours of central nervous system (CNS)","CNS tumours",.)
replace calab = subinstr(calab,"Malignant neoplasm of unspecified sites or multipl","Mutliple or
unspecified sites",.)
replace calab = subinstr(calab,"Malignant neoplasms of all specified sites (excl b","All
specified sites",.)
*replace calab = subinstr(calab,"Melanoma of skin","Skin",.)

* To make things group on plot move other GI
replace seccatype=99 if seccatype==10

lab var ncases "# cancers"
lab var seccatyp "Type of second cancer"
labmask seccatyp, value(calab)

* Setup colours for points noting which endpoints have signif RR from RCTs
gen rand_ex = 1  if (trt=="rt" & calab=="Contralateral")
replace rand_ex = 1  if (trt=="rt" & index(upper(calab),"LEUKAEMIA"))
replace rand_ex = 1  if (trt=="rt" & calab=="Lung")
replace rand_ex = 1  if (trt=="rt" & calab=="Lung (ipsilaleral)")
replace rand_ex = 1  if (trt=="rt" & calab=="Oesophagus")
replace rand_ex = 1  if (trt=="sys_hormer" & calab=="Contralateral")
replace rand_ex = 1  if (trt=="sys_hormer" & calab=="Uterine")
replace rand_ex = 1  if (trt=="sys_chem" & calab=="Acute myeloid leukaemia")
gen plt=1
```

```
replace plt=2 if sig=="*"
replace plt=3 if plt==2 & rand_ex==1

lab def plt 1 "Uninteresting" 2 "Signif, no RCT" 3 "Signif, RCT", modify
lab val plt plt
lab var plt "Plot colour"

* White for unintersting, grey for signif but not seen in RCTS, black for signif and seen in RCTs
* pc1 is colour of the CI for plt=1
local pc1="gs0"
local pc2="gs10"
local pc3="gs0"

noi tab trt plt, miss
* admetan can't handle gaps in "plt", so need to do some setting up
* RT has plt vals 1, 2, & 3, horm has 1 & 3, CT has 1, 2, 3
local rt_opt=" ci1opt(lc(`pc1') lw(vthin) msize(vsmall)) point1opt(msymbol(S) msize(vsmall) mfcol(gs16) mcol(gs16) mlcol(gs0) mlw(vthin)) ci2opt(lc(`pc2') lw(vthin) msize(vsmall)) point2opt(msymbol(S) msize(vsmall) mcol(`pc2')) ci3opt(lc(`pc3') lw(vthin) msize(vsmall)) point3opt(msymbol(S) msize(vsmall) mcol(`pc3'))"
*local et_opt=" ci1opt(lc(`pc1') lw(vthin) msize(vsmall)) point1opt(msymbol(S) msize(vsmall) mfcol(gs16) mcol(gs16) mlcol(gs0) mlw(vthin)) ci2opt(lc(`pc2') lw(vthin) msize(vsmall)) point2opt(msymbol(S) msize(vsmall) mcol(`pc2')) ci3opt(lc(`pc3') lw(vthin) msize(vsmall)) point3opt(msymbol(S) msize(vsmall) mcol(`pc3'))"
local et_opt=" ci1opt(lc(`pc1') lw(vthin) msize(vsmall)) point1opt(msymbol(S) msize(vsmall) mfcol(gs16) mcol(gs16) mlcol(gs0) mlw(vthin)) ci2opt(lc(`pc3') lw(vthin) msize(vsmall)) point2opt(msymbol(S) msize(vsmall) mcol(`pc3'))"
local ct_opt=" ci1opt(lc(`pc1') lw(vthin) msize(vsmall)) point1opt(msymbol(S) msize(vsmall) mfcol(gs16) mcol(gs16) mlcol(gs0) mlw(vthin)) ci2opt(lc(`pc2') lw(vthin) msize(vsmall)) point2opt(msymbol(S) msize(vsmall) mcol(`pc2')) ci3opt(lc(`pc3') lw(vthin) msize(vsmall)) point3opt(msymbol(S) msize(vsmall) mcol(`pc3'))"

* Super-group cancers (ALWAYS CHECK CODES ARE VALID AS CAN CHANGE!!!!)
capture drop grp
gen grp=1 if seccatyp>=14 & seccatyp<=17
replace grp=2 if seccatyp>=21 & seccatyp<=25
replace grp=3 if seccatyp==11 | seccatyp==13
replace grp=4 if seccatyp==12
replace grp=5 if (seccatyp>=1 & seccatyp<=2) | seccatyp==19 | seccatyp==20
replace grp=6 if (seccatyp>=3 & seccatyp<=4) | seccatyp==7
replace grp=7 if seccatyp==5 | seccatyp==8
replace grp=8 if seccatyp==6
replace grp=9 if seccatyp==9  | (seccatyp>=35 & seccatyp<=36) |  seccatyp==99
replace grp=10 if seccatyp==18
replace grp=11 if seccatyp==26 | seccatyp==27 | seccatyp==29
replace grp=12 if seccatyp==34
recode grp .=13

lab def grp 1 "{bf:Gynaecological}" 2 "{bf:Hematopietic/lymphoid}" 3 "{bf:Bone and soft tissue}" 4 "{bf:Skin}" 5 "{bf:Head and neck/endocrine}" 6 "{bf:Upper gastrointestinal}" 7 "{bf:Lower or other gastrointestinal}" 8 "{bf:Liver}" 9 "{bf:Respiratory}" 10 "{bf:Central nervous system}" 11 "{bf:All cancers (excluding breast)}" 12 "{bf:Breast}" 13 "{bf:Unclassified}",modify
lab val grp grp
lab var grp "Second cancer group"
compress
format ncases %-5.0f
gen sncases=string(ncases,"%-5.0f")
lab var ncases "Number_of cancers"
lab var sncases "Number_of cancers"


* Forest
* Do a version in admetan that will place the RRs beside each other and just have the cancer description once
* Buildup multiplot
* Ca names & # cases
local scale = "0.5 1.0 1.5 2.0 2.5"
admetan logrr selogrr if trt=="rt", by(grp) sortby(seccatype) study(calab) lcols(sncases) eform level(`wci') nowt keepa keepord forestplot( title("Type of second cancer", size(tiny) c(black)) graphr(color(white)) nooverall nosubgroup nostats nowt nohet nowarn colsonly name(cancers,replace) savedims(A))
* RRs for RT
admetan logrr selogrr if trt=="rt",   by(grp)    sortby(seccatype) effect(Adjusted rate ratio) study(calab) eform level(`wci') nowt keepa keepord forestplot(xlab(`scale', force)
```

```
title("Radiotherapy (or BCS) (recorded vs not)", size(tiny) c(black))         plotid(plt)
`rt_opt' diamopt(lc(black) lw(thin)) olineopt(lc(black) lp(dash) lw(thin)) rcols(ex_ca)
astext(50)  graphr(color(white)) null(1) leftj nobox nooverall nosubgroup nonames nowarn
name(rt,replace) usedims(A) )
* RRs for endocrine
admetan logrr selogrr if trt=="sys_hormer", by(grp) sortby(seccatype) effect(Adjusted rate
ratio) study(calab) eform level(`wci') nowt keepa keepord forestplot(xlab(`scale', force)
title("Endocrine therapy (or ER+ve) (recorded vs not)", size(tiny) c(black)) plotid(plt)
`et_opt' diamopt(lc(black) lw(thin)) olineopt(lc(black) lp(dash) lw(thin)) rcols(ex_ca)
astext(50) graphr(color(white)) null(1) leftj nobox nooverall nosubgroup nonames nowarn
name(endo,replace) usedims(A) )
* RRs for chemo
admetan logrr selogrr if trt=="sys_chem", by(grp) sortby(seccatype) effect(Adjusted rate ratio)
study(calab) eform level(`wci') nowt keepa keepord forestplot(xlab(`scale', force)
title("Chemotherapy (recorded vs not)", size(tiny) c(black))     plotid(plt)  `ct_opt'
diamopt(lc(black) lw(thin)) olineopt(lc(black) lp(dash) lw(thin)) rcols(ex_ca) astext(50)
graphr(color(white)) null(1) leftj nobox nooverall nosubgroup nonames nowarn name(chemo,replace)
usedims(A) )
* Combine
graph combine cancers rt endo chemo, rows(1) scale(*1.9) imargin(zero) graphr(color(white)
margin(zero) lcolor(white)) xsize(11.5) ysize(6) saving(gr_trt_rater_2ndca_adj_fp_comb,replace)
gr export gr_trt_rater_2ndca_adj_fp_comb.pdf,replace

*****adjusted rate ratios, years 0-9 only
use tmp,clear
keep if yrsdiag<10
capture postclose temp
postfile temp str20 trt seccatype str50 calab ncases prev cf logrr selogrr using ca_rr_adj_09,
replace
foreach ca of varlist ca_dg* {
       local ca_num=substr("`ca'", 6,.)
       local calab: variable label `ca'
       noi di "ca: `ca' [`calab']"
       summ `ca' if `ca'==1
       local ncases=r(N)
       local ca_trt="bcs rt sys_hormer sys_chem"
       preserve
       collapse (sum) `ca' pyrs, by(`array')
*      noi poisson `ca' `adj_array', nolog exp(pyrs)
       egen strata=group(`strata_array'),lab
       xtset strata
       noi xtpoisson `ca' `ca_trt', nolog exp(pyrs) irr fe
       foreach var of local ca_trt {
              local logrr=_coef[`var']
              local selogrr=_se[`var']
* Setup treatment prevlances
              if "`var'"=="bcs" local prev=`p_bcs'
              if "`var'"=="rt" local prev=`p_rt'
              if "`var'"=="sys_hormer" local prev=`p_et'
              if "`var'"=="sys_chem" local prev=`p_ct'
* For contralaterals
              if "`var'"=="rt" & trim("`calab'")=="Invasive breast (cont./unk.)" local
prev=`p_rt_noet'
              if "`var'"=="sys_hormer" & trim("`calab'")=="Invasive breast (cont./unk.)" local
prev=`p_nort_et'
* Correction factor for missclassification
              local cf=`cf_oth'
              if "`var'"=="sys_hormer" & trim("`calab'")=="Uterine" local cf=`cf_ut'
              if "`var'"=="sys_hormer" & trim("`calab'")=="Invasive breast (cont./unk.)" local
cf=`cf_cont'
              post temp ("`var'") (`ca_num') ("`calab'") (`ncases') (`prev') (`cf') (`logrr')
(`selogrr')
       }
       restore
}
capture postclose temp

* Plot
use ca_rr_adj_09, clear
compress

gen p=2*(1-normprob(abs(logrr/selogrr)))
gen sig="*" if p<0.01 & logrr>0
replace sig="*" if p<0.01 & trim(calab)=="Invasive breast (cont./unk.)" &
trim(trt)=="sys_hormer"
```

```
replace sig="" if trim(calab)=="Malignant neoplasms of all specified sites (excl b"
lab var sig "RR>1 & p<0.01"
gen rr=cf*exp(logrr)
gen rrlt1=rr<1 & sig=="*"
replace rr=1/rr if sig=="*" & rr<1
gen tmp=ncases*prev*(rr-1)/rr if sig=="*"
replace tmp=round(tmp,5)
gen ex_ca=string(tmp)
replace ex_ca="-"+ex_ca if rrlt1==1
replace ex_ca="" if ex_ca=="."
lab var ex_ca "Excess cancers"


lab var calab " "
*replace calab="Contralateral" if calab=="Breast (cont.)"
replace calab="Contralateral" if calab=="Invasive breast (cont./unk.)"
replace calab = subinstr(calab,"cont.","contralaleral",.)
replace calab = subinstr(calab,"ipsi.","ipsilaleral",.)
replace calab = subinstr(calab,"gastrointestinal","GI",.)
*replace calab = subinstr(calab,"respiratory and intrathoracic","resp. or IT",.)
replace calab = subinstr(calab,"Tumours of central nervous system (CNS)","CNS tumours",.)
replace calab = subinstr(calab,"Malignant neoplasm of unspecified sites or multipl","Mutliple or
unspecified sites",.)
replace calab = subinstr(calab,"Malignant neoplasms of all specified sites (excl b","All
specified sites",.)
*replace calab = subinstr(calab,"Melanoma of skin","Skin",.)

* To make things group on plot move other GI
replace seccatype=99 if seccatype==10

lab var ncases "# cancers"
lab var seccatyp "Type of second cancer"
labmask seccatyp, value(calab)

* Setup colours for points noting which endpoints have signif RR from RCTs
gen rand_ex = 1  if (trt=="rt" & calab=="Contralateral")
replace rand_ex = 1  if (trt=="rt" & index(upper(calab),"LEUKAEMIA"))
replace rand_ex = 1  if (trt=="rt" & calab=="Lung")
replace rand_ex = 1  if (trt=="rt" & calab=="Lung (ipsilaleral)")
replace rand_ex = 1  if (trt=="rt" & calab=="Oesophagus")
replace rand_ex = 1  if (trt=="sys_hormer" & calab=="Contralateral")
replace rand_ex = 1  if (trt=="sys_hormer" & calab=="Uterine")
replace rand_ex = 1  if (trt=="sys_chem" & calab=="Acute myeloid leukaemia")
gen plt=1
replace plt=2 if sig=="*"
replace plt=3 if plt==2 & rand_ex==1

lab def plt 1 "Uninteresting" 2 "Signif, no RCT" 3 "Signif, RCT", modify
lab val plt plt
lab var plt "Plot colour"

* White for unintersting, grey for signif but not seen in RCTS, black for signif and seen in
RCTs
* pc1 is colour of the CI for plt=1
 local pc1="gs0"
local pc2="gs10"
local pc3="gs0"

noi tab trt plt, miss
* admetan can't handle gaps in "plt", so need to do some setting up
* RT has plt vals 1 & 3, horm has 1 & 3, CT has 1, 2, & 3
*local rt_opt=" ci1opt(lc(`pc1') lw(vthin) msize(vsmall)) point1opt(msymbol(S) msize(vsmall)
mfcol(gs16) mcol(gs16) mlcol(gs0) mlw(vthin)) ci2opt(lc(`pc2') lw(vthin) msize(vsmall))
point2opt(msymbol(S) msize(vsmall) mcol(`pc2')) ci3opt(lc(`pc3') lw(vthin) msize(vsmall))
point3opt(msymbol(S) msize(vsmall) mcol(`pc3'))"
local rt_opt=" ci1opt(lc(`pc1') lw(vthin) msize(vsmall)) point1opt(msymbol(S) msize(vsmall)
mfcol(gs16) mcol(gs16) mlcol(gs0) mlw(vthin)) ci2opt(lc(`pc3') lw(vthin) msize(vsmall))
point2opt(msymbol(S) msize(vsmall) mcol(`pc3'))"
*local et_opt=" ci1opt(lc(`pc1') lw(vthin) msize(vsmall)) point1opt(msymbol(S) msize(vsmall)
mfcol(gs16) mcol(gs16) mlcol(gs0) mlw(vthin)) ci2opt(lc(`pc2') lw(vthin) msize(vsmall))
point2opt(msymbol(S) msize(vsmall) mcol(`pc2')) ci3opt(lc(`pc3') lw(vthin) msize(vsmall))
point3opt(msymbol(S) msize(vsmall) mcol(`pc3'))"
local et_opt=" ci1opt(lc(`pc1') lw(vthin) msize(vsmall)) point1opt(msymbol(S) msize(vsmall)
mfcol(gs16) mcol(gs16) mlcol(gs0) mlw(vthin)) ci2opt(lc(`pc3') lw(vthin) msize(vsmall))
point2opt(msymbol(S) msize(vsmall) mcol(`pc3'))"
```

```
local ct_opt=" ci1opt(lc(`pc1') lw(vthin) msize(vsmall)) point1opt(msymbol(S) msize(vsmall)
mfcol(gs16) mcol(gs16) mlcol(gs0) mlw(vthin)) ci2opt(lc(`pc2') lw(vthin) msize(vsmall))
point2opt(msymbol(S) msize(vsmall) mcol(`pc2')) ci3opt(lc(`pc3') lw(vthin) msize(vsmall))
point3opt(msymbol(S) msize(vsmall) mcol(`pc3'))"

* Super-group cancers (ALWAYS CHECK CODES ARE VALID AS CAN CHANGE!!!!)
capture drop grp
gen grp=1 if seccatyp>=14 & seccatyp<=17
replace grp=2 if seccatyp>=21 & seccatyp<=25
replace grp=3 if seccatyp==11 | seccatyp==13
replace grp=4 if seccatyp==12
replace grp=5 if (seccatyp>=1 & seccatyp<=2) | seccatyp==19 | seccatyp==20
replace grp=6 if (seccatyp>=3 & seccatyp<=4) | seccatyp==7
replace grp=7 if seccatyp==5 | seccatyp==8
replace grp=8 if seccatyp==6
replace grp=9 if seccatyp==9  | (seccatyp>=35 & seccatyp<=36) |  seccatyp==99
replace grp=10 if seccatyp==18
replace grp=11 if seccatyp==26 | seccatyp==27 | seccatyp==29
replace grp=12 if seccatyp==34
recode grp .=13

lab def grp 1 "{bf:Gynaecological}" 2 "{bf:Hematopietic/lymphoid}" 3 "{bf:Bone and soft tissue}"
4 "{bf:Skin}" 5 "{bf:Head and neck/endocrine}" 6 "{bf:Upper gastrointestinal}" 7 "{bf:Lower or
other gastrointestinal}" 8 "{bf:Liver}" 9 "{bf:Respiratory}" 10 "{bf:Central nervous system}" 11
"{bf:All cancers (excluding breast)}" 12 "{bf:Breast}" 13 "{bf:Unclassified}",modify
lab val grp grp
lab var grp "Second cancer group (RRs for 0-9 yrs of FU)"

compress
format ncases %-5.0f
gen sncases=string(ncases,"%-5.0f")
lab var ncases "Number_of cancers"
lab var sncases "Number_of cancers"


* Forest
* Do a version in admetan that will place the RRs beside each other and just have the cancer
description once
* Buildup multiplot
* Ca names & # cases
local scale = "0.5 1.0 1.5 2.0 2.5"
admetan logrr selogrr if trt=="rt", by(grp) sortby(seccatype) study(calab) lcols(sncases) eform
level(`wci') nowt keepa keepord forestplot( title("Type of second cancer", size(tiny) c(black))
graphr(color(white)) nooverall nosubgroup nostats nowt nohet nowarn colsonly
name(cancers,replace) savedims(A))
* RRs for RT
admetan logrr selogrr if trt=="rt",   by(grp)    sortby(seccatype) effect(Adjusted rate ratio)
study(calab) eform level(`wci') nowt keepa keepord forestplot(xlab(`scale', force)
title("Radiotherapy (or BCS) (recorded vs not)", size(tiny) c(black))         plotid(plt)
`rt_opt' diamopt(lc(black) lw(thin)) olineopt(lc(black) lp(dash) lw(thin)) astext(50)
graphr(color(white)) null(1) leftj nobox nooverall nosubgroup nonames nowarn name(rt,replace)
usedims(A) )
* RRs for endocrine
admetan logrr selogrr if trt=="sys_hormer", by(grp) sortby(seccatype) effect(Adjusted rate
ratio) study(calab) eform level(`wci') nowt keepa keepord forestplot(xlab(`scale', force)
title("Endocrine therapy (or ER+ve) (recorded vs not)", size(tiny) c(black)) plotid(plt)
`et_opt' diamopt(lc(black) lw(thin)) olineopt(lc(black) lp(dash) lw(thin)) astext(50)
graphr(color(white)) null(1) leftj nobox nooverall nosubgroup nonames nowarn name(endo,replace)
usedims(A) )
* RRs for chemo
admetan logrr selogrr if trt=="sys_chem", by(grp) sortby(seccatype) effect(Adjusted rate ratio)
study(calab) eform level(`wci') nowt keepa keepord forestplot(xlab(`scale', force)
title("Chemotherapy (recorded vs not)", size(tiny) c(black))     plotid(plt)  `ct_opt'
diamopt(lc(black) lw(thin)) olineopt(lc(black) lp(dash) lw(thin))  astext(50)
graphr(color(white)) null(1) leftj nobox nooverall nosubgroup nonames nowarn name(chemo,replace)
usedims(A) )
* Combine
graph combine cancers rt endo chemo, rows(1) scale(*1.9) imargin(zero) graphr(color(white)
margin(zero) lcolor(white)) xsize(11.5) ysize(6)
saving(gr_trt_rater_2ndca_adj_09_fp_comb,replace)
gr export gr_trt_rater_2ndca_adj_09_fp_comb.pdf,replace

*****adjusted rate ratios, years 10+ only
use tmp,clear
drop if yrsdiag<10
```

```
capture postclose temp
postfile temp str20 trt seccatype str50 calab ncases prev cf logrr selogrr using ca_rr_adj_10p,
replace
foreach ca of varlist ca_dg* {
	local ca_num=substr("`ca'", 6,.)
	local calab: variable label `ca'
	noi di "ca: `ca' [`calab']"
	summ `ca' if `ca'==1
	local ncases=r(N)
	local ca_trt="bcs rt sys_hormer sys_chem"
	preserve
	collapse (sum) `ca' pyrs, by(`array')
*	noi poisson `ca' `adj_array', nolog exp(pyrs)
	egen strata=group(`strata_array'),lab
	xtset strata
	noi xtpoisson `ca' `ca_trt', nolog exp(pyrs) irr fe
	foreach var of local ca_trt {
		local logrr=_coef[`var']
		local selogrr=_se[`var']
* Setup treatment prevlances
		if "`var'"=="bcs" local prev=`p_bcs'
		if "`var'"=="rt" local prev=`p_rt'
		if "`var'"=="sys_hormer" local prev=`p_et'
		if "`var'"=="sys_chem" local prev=`p_ct'
* For contralaterals
		if "`var'"=="rt" & trim("`calab'")=="Invasive breast (cont./unk.)" local
prev=`p_rt_noet'
		if "`var'"=="sys_hormer" & trim("`calab'")=="Invasive breast (cont./unk.)" local
prev=`p_nort_et'
* Correction factor for missclassification
		local cf=`cf_oth'
		if "`var'"=="sys_hormer" & trim("`calab'")=="Uterine" local cf=`cf_ut'
		if "`var'"=="sys_hormer" & trim("`calab'")=="Invasive breast (cont./unk.)" local
cf=`cf_cont'
		post temp ("`var'") (`ca_num') ("`calab'") (`ncases') (`prev') (`cf') (`logrr')
(`selogrr')
	}
	restore
}
capture postclose temp

* Plot
use ca_rr_adj_10p, clear
compress

gen p=2*(1-normprob(abs(logrr/selogrr)))
gen sig="*" if p<0.01 & logrr>0
replace sig="*" if p<0.01 & trim(calab)=="Invasive breast (cont./unk.)" &
trim(trt)=="sys_hormer"
replace sig="" if trim(calab)=="Malignant neoplasms of all specified sites (excl b"
lab var sig "RR>1 & p<0.01"
gen rr=cf*exp(logrr)
gen rrlt1=rr<1 & sig=="*"
replace rr=1/rr if sig=="*" & rr<1
gen tmp=ncases*prev*(rr-1)/rr if sig=="*"
replace tmp=round(tmp,5)
gen ex_ca=string(tmp)
replace ex_ca="-"+ex_ca if rrlt1==1
replace ex_ca="" if ex_ca=="."
lab var ex_ca "Excess cancers"

lab var calab " "
*replace calab="Contralateral" if calab=="Breast (cont.)"
replace calab="Contralateral" if calab=="Invasive breast (cont./unk.)"
replace calab = subinstr(calab,"cont.","contralaleral",.)
replace calab = subinstr(calab,"ipsi.","ipsilaleral",.)
replace calab = subinstr(calab,"gastrointestinal","GI",.)
*replace calab = subinstr(calab,"respiratory and intrathoracic","resp. or IT",.)
replace calab = subinstr(calab,"Tumours of central nervous system (CNS)","CNS tumours",.)
replace calab = subinstr(calab,"Malignant neoplasm of unspecified sites or multipl","Mutliple or
unspecified sites",.)
replace calab = subinstr(calab,"Malignant neoplasms of all specified sites (excl b","All
specified sites",.)
*replace calab = subinstr(calab,"Melanoma of skin","Skin",.)
```

```
* To make things group on plot move other GI
replace seccatype=99 if seccatype==10

lab var ncases "# cancers"
lab var seccatyp "Type of second cancer"
labmask seccatyp, value(calab)

* Setup colours for points noting which endpoints have signif RR from RCTs
gen rand_ex = 1  if (trt=="rt" & calab=="Contralateral")
replace rand_ex = 1  if (trt=="rt" & index(upper(calab),"LEUKAEMIA"))
replace rand_ex = 1  if (trt=="rt" & calab=="Lung")
replace rand_ex = 1  if (trt=="rt" & calab=="Lung (ipsilaleral)")
replace rand_ex = 1  if (trt=="rt" & calab=="Oesophagus")
replace rand_ex = 1  if (trt=="sys_hormer" & calab=="Contralateral")
replace rand_ex = 1  if (trt=="sys_hormer" & calab=="Uterine")
replace rand_ex = 1  if (trt=="sys_chem" & calab=="Acute myeloid leukaemia")
gen plt=1
replace plt=2 if sig=="*"
replace plt=3 if plt==2 & rand_ex==1

lab def plt 1 "Uninteresting" 2 "Signif, no RCT" 3 "Signif, RCT", modify
lab val plt plt
lab var plt "Plot colour"

* White for unintersting, grey for signif but not seen in RCTS, black for signif and seen in
RCTs
local pc1="gs0"
local pc2="gs10"
local pc3="gs0"

noi tab trt plt, miss
* admetan can't handle gaps in "plt", so need to do some setting up
* RT has all plt vals 1, 3, horm has vals 1, 2, & 3, CT has vals 1, & 2
*local rt_opt=" ci1opt(lc(`pc1') lw(vthin) msize(vsmall)) point1opt(msymbol(S) msize(vsmall)
mfcol(gs16) mcol(gs16) mlcol(gs0) mlw(vthin)) ci2opt(lc(`pc2') lw(vthin) msize(vsmall))
point2opt(msymbol(S) msize(vsmall) mcol(`pc2')) ci3opt(lc(`pc3') lw(vthin) msize(vsmall))
point3opt(msymbol(S) msize(vsmall) mcol(`pc3'))"
local rt_opt=" ci1opt(lc(`pc1') lw(vthin) msize(vsmall)) point1opt(msymbol(S) msize(vsmall)
mfcol(gs16) mcol(gs16) mlcol(gs0) mlw(vthin)) ci2opt(lc(`pc3') lw(vthin) msize(vsmall))
point2opt(msymbol(S) msize(vsmall) mcol(`pc3'))"
local et_opt=" ci1opt(lc(`pc1') lw(vthin) msize(vsmall)) point1opt(msymbol(S) msize(vsmall)
mfcol(gs16) mcol(gs16) mlcol(gs0) mlw(vthin)) ci2opt(lc(`pc2') lw(vthin) msize(vsmall))
point2opt(msymbol(S) msize(vsmall) mcol(`pc2')) ci3opt(lc(`pc3') lw(vthin) msize(vsmall))
point3opt(msymbol(S) msize(vsmall) mcol(`pc3'))"
* For some reason admetan won't accept the plot order endo, so kludge it here
local et_opt=" ci1opt(lc(`pc1') lw(vthin) msize(vsmall)) point1opt(msymbol(S) msize(vsmall)
mfcol(gs16) mcol(gs16) mlcol(gs0) mlw(vthin)) ci2opt(lc(`pc3') lw(vthin) msize(vsmall))
point2opt(msymbol(S) msize(vsmall) mcol(`pc3')) ci3opt(lc(`pc2') lw(vthin) msize(vsmall))
point3opt(msymbol(S) msize(vsmall) mcol(`pc2'))"
*local ct_opt=" ci1opt(lc(`pc1') lw(vthin) msize(vsmall)) point1opt(msymbol(S) msize(vsmall)
mfcol(gs16) mcol(gs16) mlcol(gs0) mlw(vthin)) ci2opt(lc(`pc2') lw(vthin) msize(vsmall))
point2opt(msymbol(S) msize(vsmall) mcol(`pc2')) ci3opt(lc(`pc3') lw(vthin) msize(vsmall))
point3opt(msymbol(S) msize(vsmall) mcol(`pc3'))"
local ct_opt=" ci1opt(lc(`pc1') lw(vthin) msize(vsmall)) point1opt(msymbol(S) msize(vsmall)
mfcol(gs16) mcol(gs16) mlcol(gs0) mlw(vthin)) ci2opt(lc(`pc2') lw(vthin) msize(vsmall))
point2opt(msymbol(S) msize(vsmall) mcol(`pc2'))"

* Super-group cancers (ALWAYS CHECK CODES ARE VALID AS CAN CHANGE!!!!)
capture drop grp
gen grp=1 if seccatyp>=14 & seccatyp<=17
replace grp=2 if seccatyp>=21 & seccatyp<=25
replace grp=3 if seccatyp==11 | seccatyp==13
replace grp=4 if seccatyp==12
replace grp=5 if (seccatyp>=1 & seccatyp<=2) | seccatyp==19 | seccatyp==20
replace grp=6 if (seccatyp>=3 & seccatyp<=4) | seccatyp==7
replace grp=7 if seccatyp==5 | seccatyp==8
replace grp=8 if seccatyp==6
replace grp=9 if seccatyp==9  | (seccatyp>=35 & seccatyp<=36) |  seccatyp==99
replace grp=10 if seccatyp==18
replace grp=11 if seccatyp==26 | seccatyp==27 | seccatyp==29
replace grp=12 if seccatyp==34
recode grp .=13

lab def grp 1 "{bf:Gynaecological}" 2 "{bf:Hematopietic/lymphoid}" 3 "{bf:Bone and soft tissue}"
4 "{bf:Skin}" 5 "{bf:Head and neck/endocrine}" 6 "{bf:Upper gastrointestinal}" 7 "{bf:Lower or
```

```
other gastrointestinal}" 8 "{bf:Liver}" 9 "{bf:Respiratory}" 10 "{bf:Central nervous system}" 11
"{bf:All cancers (excluding breast)}" 12 "{bf:Breast}" 13 "{bf:Unclassified}",modify
lab val grp grp
lab var grp "Second cancer group (RRs for 10+ yrs of FU)"

compress
format ncases %-5.0f
gen sncases=string(ncases,"%-5.0f")
lab var ncases "Number_of cancers"
lab var sncases "Number_of cancers"

* Forest
* Do a version in admetan that will place the RRs beside each other and just have the cancer
description once
* Buildup multiplot
* Ca names & # cases
local scale = "0.5 1.0 1.5 2.0 2.5"
admetan logrr selogrr if trt=="rt", by(grp) sortby(seccatype) study(calab) lcols(sncases) eform
level(`wci') nowt keepa keepord forestplot( title("Type of second cancer", size(tiny) c(black))
graphr(color(white)) nooverall nosubgroup nostats nowt nohet nowarn colsonly
name(cancers,replace) savedims(A))
* RRs for RT
admetan logrr selogrr if trt=="rt",   by(grp)    sortby(seccatype) effect(Adjusted rate ratio)
study(calab) eform level(`wci') nowt keepa keepord forestplot(xlab(`scale', force)
title("Radiotherapy (or BCS) (recorded vs not)", size(tiny) c(black))        plotid(plt)
`rt_opt' diamopt(lc(black) lw(thin)) olineopt(lc(black) lp(dash) lw(thin)) astext(50)
graphr(color(white)) null(1) leftj nobox nooverall nosubgroup nonames nowarn name(rt,replace)
usedims(A) )
* RRs for endocrine
admetan logrr selogrr if trt=="sys_hormer", by(grp) sortby(seccatype) effect(Adjusted rate
ratio) study(calab) eform level(`wci') nowt keepa keepord forestplot(xlab(`scale', force)
title("Endocrine therapy (or ER+ve) (recorded vs not)", size(tiny) c(black)) plotid(plt)
`et_opt' diamopt(lc(black) lw(thin)) olineopt(lc(black) lp(dash) lw(thin))astext(50)
graphr(color(white)) null(1) leftj nobox nooverall nosubgroup nonames nowarn name(endo,replace)
usedims(A) )
* RRs for chemo
admetan logrr selogrr if trt=="sys_chem", by(grp) sortby(seccatype) effect(Adjusted rate ratio)
study(calab) eform level(`wci') nowt keepa keepord forestplot(xlab(`scale', force)
title("Chemotherapy (recorded vs not)", size(tiny) c(black))     plotid(plt)  `ct_opt'
diamopt(lc(black) lw(thin)) olineopt(lc(black) lp(dash) lw(thin))  astext(50)
graphr(color(white)) null(1) leftj nobox nooverall nosubgroup nonames nowarn name(chemo,replace)
usedims(A) )
* Combine
graph combine cancers rt endo chemo, rows(1) scale(*1.9) imargin(zero) graphr(color(white)
margin(zero) lcolor(white)) xsize(11.5) ysize(6)
saving(gr_trt_rater_2ndca_adj_10p_fp_comb,replace)
gr export gr_trt_rater_2ndca_adj_10p_fp_comb.pdf,replace


******* plot an all years version with ipsi lung split into 0-9 & 10+
use ca_rr_adj_09, clear
gen yr=0
append using ca_rr_adj_10p
recode yr .=10
keep if calab=="Lung (ipsi.)"
local lu_num=seccatype[1]
*replace seccatype = seccatype + 1 if yr==10
save tmp_lung,replace

* All years result
use ca_rr_adj, clear
compress

* Drop all years lung and replace with lung split by FU
*replace seccatype = seccatype + 1 if seccatype>`lu_num'
drop if calab=="Lung (ipsi.)"
append using tmp_lung

gen p=2*(1-normprob(abs(logrr/selogrr)))
gen sig="*" if p<0.01 & logrr>0
replace sig="*" if p<0.01 & trim(calab)=="Invasive breast (cont./unk.)" &
trim(trt)=="sys_hormer"
replace sig="" if trim(calab)=="Malignant neoplasms of all specified sites (excl b"
lab var sig "RR>1 & p<0.01"
gen rr=cf*exp(logrr)
```

```
gen rrlt1=rr<1 & sig=="*"
replace rr=1/rr if sig=="*" & rr<1
gen tmp=ncases*prev*(rr-1)/rr if sig=="*"
replace tmp=round(tmp,5)
gen ex_ca=string(tmp)
replace ex_ca="-"+ex_ca if rrlt1==1
replace ex_ca="" if ex_ca=="."
lab var ex_ca "Excess cancers"

* To make things group on plot move other GI
replace seccatype=99 if seccatype==10

* Super-group cancers (ALWAYS CHECK CODES ARE VALID AS CAN CHANGE!!!!)
capture drop grp
gen grp=1 if seccatyp>=14 & seccatyp<=17
replace grp=2 if seccatyp>=21 & seccatyp<=25
replace grp=3 if seccatyp==11 | seccatyp==13
replace grp=4 if seccatyp==12
replace grp=5 if (seccatyp>=1 & seccatyp<=2) | seccatyp==19 | seccatyp==20
replace grp=6 if (seccatyp>=3 & seccatyp<=4) | seccatyp==7
replace grp=7 if seccatyp==5 | seccatyp==8
replace grp=8 if seccatyp==6
replace grp=9 if seccatyp==9  | (seccatyp>=35 & seccatyp<=36) |  seccatyp==99
replace grp=10 if seccatyp==18
replace grp=11 if seccatyp==26 | seccatyp==27 | seccatyp==29
replace grp=12 if seccatyp==34
recode grp .=13

lab def grp 1 "{bf:Gynaecological}" 2 "{bf:Hematopietic/lymphoid}" 3 "{bf:Bone and soft tissue}"
4 "{bf:Skin}" 5 "{bf:Head and neck/endocrine}" 6 "{bf:Upper gastrointestinal}" 7 "{bf:Lower or
other gastrointestinal}" 8 "{bf:Liver}" 9 "{bf:Respiratory}" 10 "{bf:Central nervous system}" 11
"{bf:All cancers (excluding breast)}" 12 "{bf:Breast}" 13 "{bf:Unclassified}",modify
lab val grp grp
lab var grp "Second cancer group"
compress
format ncases %-5.0f
gen sncases=string(ncases,"%-5.0f")
lab var ncases "Number_of cancers"
lab var sncases "Number_of cancers"

* Sort out ipsi lung
replace seccatype = seccatype + 1 if seccatype>`lu_num'
replace seccatype = seccatype + 1 if seccatype==`lu_num' & yr==10

replace calab = "Lung (ipsi.) [follow-up years 0-9]"  if calab=="Lung (ipsi.)" &
seccatype==`lu_num'
replace calab = "Lung (ipsi.) [follow-up years 10-29]"  if calab=="Lung (ipsi.)" &
seccatype==`lu_num'+1

lab var calab " "
*replace calab="Contralateral" if calab=="Breast (cont.)"
replace calab="Contralateral" if calab=="Invasive breast (cont./unk.)"
replace calab = subinstr(calab,"cont.","contralaleral",.)
replace calab = subinstr(calab,"ipsi.","ipsilaleral",.)
replace calab = subinstr(calab,"gastrointestinal","GI",.)
*replace calab = subinstr(calab,"respiratory and intrathoracic","resp. or IT",.)
replace calab = subinstr(calab,"Tumours of central nervous system (CNS)","CNS tumours",.)
replace calab = subinstr(calab,"Malignant neoplasm of unspecified sites or multipl","Mutliple or
unspecified sites",.)
replace calab = subinstr(calab,"Malignant neoplasms of all specified sites (excl b","All
specified sites",.)
*replace calab = subinstr(calab,"Melanoma of skin","Skin",.)
compress

lab var ncases "# cancers"
lab var seccatyp "Type of second cancer"
labmask seccatyp, value(calab)

* Setup colours for points noting which endpoints have signif RR from RCTs
gen rand_ex = 1  if (trt=="rt" & calab=="Contralateral")
replace rand_ex = 1  if (trt=="rt" & index(upper(calab),"LEUKAEMIA"))
*replace rand_ex = 1  if (trt=="rt" & calab=="Lung")
replace rand_ex = 1  if (trt=="rt" & calab=="Lung (ipsilaleral) [follow-up years 10-29]")
replace rand_ex = 1  if (trt=="rt" & calab=="Oesophagus")
replace rand_ex = 1  if (trt=="sys_hormer" & calab=="Contralateral")
```

```
replace rand_ex = 1  if (trt=="sys_hormer" & calab=="Uterine")
replace rand_ex = 1  if (trt=="sys_chem" & calab=="Acute myeloid leukaemia")
gen plt=1
replace plt=2 if sig=="*"
replace plt=3 if plt==2 & rand_ex==1

lab def plt 1 "Uninteresting" 2 "Signif, no RCT" 3 "Signif, RCT", modify
lab val plt plt
lab var plt "Plot colour"

* White for unintersting, grey for signif but not seen in RCTS, black for signif and seen in
RCTs
* pc1 is colour of the CI for plt=1
local pc1="gs0"
local pc2="gs10"
local pc3="gs0"

noi tab trt plt, miss
* admetan can't handle gaps in "plt", so need to do some setting up
* RT has plt vals 1, 2, & 3, horm has 1 & 3, CT has 1, 2, 3
local rt_opt=" ci1opt(lc(`pc1') lw(vthin) msize(vsmall)) point1opt(msymbol(S) msize(vsmall)
mfcol(gs16) mcol(gs16) mlcol(gs0) mlw(vthin)) ci2opt(lc(`pc2') lw(vthin) msize(vsmall))
point2opt(msymbol(S) msize(vsmall) mcol(`pc2')) ci3opt(lc(`pc3') lw(vthin) msize(vsmall))
point3opt(msymbol(S) msize(vsmall) mcol(`pc3'))"
*local et_opt=" ci1opt(lc(`pc1') lw(vthin) msize(vsmall)) point1opt(msymbol(S) msize(vsmall)
mfcol(gs16) mcol(gs16) mlcol(gs0) mlw(vthin)) ci2opt(lc(`pc2') lw(vthin) msize(vsmall))
point2opt(msymbol(S) msize(vsmall) mcol(`pc2')) ci3opt(lc(`pc3') lw(vthin) msize(vsmall))
point3opt(msymbol(S) msize(vsmall) mcol(`pc3'))"
local et_opt=" ci1opt(lc(`pc1') lw(vthin) msize(vsmall)) point1opt(msymbol(S) msize(vsmall)
mfcol(gs16) mcol(gs16) mlcol(gs0) mlw(vthin)) ci2opt(lc(`pc3') lw(vthin) msize(vsmall))
point2opt(msymbol(S) msize(vsmall) mcol(`pc3'))"
local ct_opt=" ci1opt(lc(`pc1') lw(vthin) msize(vsmall)) point1opt(msymbol(S) msize(vsmall)
mfcol(gs16) mcol(gs16) mlcol(gs0) mlw(vthin)) ci2opt(lc(`pc2') lw(vthin) msize(vsmall))
point2opt(msymbol(S) msize(vsmall) mcol(`pc2')) ci3opt(lc(`pc3') lw(vthin) msize(vsmall))
point3opt(msymbol(S) msize(vsmall) mcol(`pc3'))"


* Forest
* Do a version in admetan that will place the RRs beside each other and just have the cancer
description once
* Buildup multiplot
* Ca names & # cases
local scale = "0.5 1.0 1.5 2.0 2.5"
admetan logrr selogrr if trt=="rt", by(grp) sortby(seccatype) study(calab) lcols(sncases) eform
level(`wci') nowt keepa keepord forestplot( title("Type of second cancer", size(tiny) c(black))
graphr(color(white)) nooverall nosubgroup nostats nowt nohet nowarn colsonly
name(cancers,replace) savedims(A))
* RRs for RT
admetan logrr selogrr if trt=="rt",   by(grp)    sortby(seccatype) effect(Adjusted rate ratio)
study(calab) eform level(`wci') nowt keepa keepord forestplot(xlab(`scale', force)
title("Radiotherapy (or BCS) (recorded vs not)", size(tiny) c(black))        plotid(plt)
`rt_opt' diamopt(lc(black) lw(thin)) olineopt(lc(black) lp(dash) lw(thin)) rcols(ex_ca)
astext(50)  graphr(color(white)) null(1) leftj nobox nooverall nosubgroup nonames nowarn
name(rt,replace) usedims(A) )
* RRs for endocrine
admetan logrr selogrr if trt=="sys_hormer", by(grp) sortby(seccatype) effect(Adjusted rate
ratio) study(calab) eform level(`wci') nowt keepa keepord forestplot(xlab(`scale', force)
title("Endocrine therapy (or ER+ve) (recorded vs not)", size(tiny) c(black)) plotid(plt)
`et_opt' diamopt(lc(black) lw(thin)) olineopt(lc(black) lp(dash) lw(thin)) rcols(ex_ca)
astext(50) graphr(color(white)) null(1) leftj nobox nooverall nosubgroup nonames nowarn
name(endo,replace) usedims(A) )
* RRs for chemo
admetan logrr selogrr if trt=="sys_chem", by(grp) sortby(seccatype) effect(Adjusted rate ratio)
study(calab) eform level(`wci') nowt keepa keepord forestplot(xlab(`scale', force)
title("Chemotherapy (recorded vs not)", size(tiny) c(black))     plotid(plt)  `ct_opt'
diamopt(lc(black) lw(thin)) olineopt(lc(black) lp(dash) lw(thin)) rcols(ex_ca) astext(50)
graphr(color(white)) null(1) leftj nobox nooverall nosubgroup nonames nowarn name(chemo,replace)
usedims(A) )
* Combine
graph combine cancers rt endo chemo, rows(1) scale(*1.9) imargin(zero) graphr(color(white)
margin(zero) lcolor(white)) xsize(11.5) ysize(6)
saving(gr_trt_rater_2ndca_adj_fp_comb_v2,replace)
gr export gr_trt_rater_2ndca_adj_fp_comb_v2.pdf,replace

* Save datafile for BMJ fig 4
```

```
save bmj_fig4_data.dta, replace

}

***************************************
* RESET GLOBAL CI LEVEL TO 95 ON EXIT *
***************************************
set level 95

***************
* Logical end *
***************
```

```
* Procedure: var_cif.do <fu> <w> <vint> <vtot> <py> <se_vint>
*
* Works out the variance of the cumulative incidence
* Based on Tom Fearn's method - double precision required as involves very small quantities
* scalar gives more precision than local
*
*      fu  = name of time interval variable
*      w  = width of time interval variable
*      vint = name of outcome of interest variable
*      vtot = name of any event variable
*      py = person-years
*      se_vint = name to use to hold the SE of vint
*
* PMcG: Jun 2023

* User input
      local fu "`1'"
      local w "`2'"
      local vint "`3'"
      local vtot "`4'"
      local py "`5'"
      local se_vint "`6'"

* These variables are going to be created during the run, brutally ensure they don't already
exist
      local array "f eai eai2 easqi ebi ebi2 ebsqi vcr eabi pebi2 pebi2_l pebsqi pebsqi_l p1
p2b"
      foreach var of local array {
            capture drop `var'
      }

*************************************
* Setup components for expectations *
*************************************
      gen double f=`w'/`py'
* For `vint' expectations
      gen double eai=1-exp(-`vint'*(1-exp(-f)))
      gen double eai2=eai*eai
      gen double easqi=1-2*exp(-`vint'*(1-exp(-f))) + exp(-`vint'*(1-exp(-2*f)))

* For vtot expectations
      gen double ebi=exp(-`vtot'*(1-exp(-f)))
      gen double ebi2=ebi*ebi
      gen double ebsqi=exp(-`vtot'*(1-exp(-2*f)))

* For `vint' & `vtot' combination expectations (need competing risk events here)
      gen double vcr=`vtot'-`vint'
      gen double eabi =(exp(-`vint'*(1-exp(-f))) - exp(-`vint'*(1-exp(-2*f)))) *  exp(-vcr*(1-
exp(-f)))

* Variance part, form product of bi and then lag as requires multiplication only upto time=i-1
      sort `fu'
      gen double pebi2=exp(sum(log(ebi2)))
      gen double pebi2_l=pebi2[_n-1]
      replace pebi2_l=1 if _n==1
      gen double pebsqi=exp(sum(log(ebsqi)))
      gen double pebsqi_l=pebsqi[_n-1]
      replace pebsqi_l=1 if _n==1
      gen double p1=sum(easqi*pebsqi_l - eai2*pebi2_l)

* For initial interval, no covariance so, variance is easy
      capture drop `se_vint'
      gen double `se_vint' = sqrt(p1) if _n==1

* Covariance part
* Easy bit!
      gen double p2b = (eabi*pebsqi_l - eai*ebi*pebi2_l)

* Need to loop round FU intervals to get the sum of the covariance
      summ `fu'
      local nrows=r(N)
      local i=2
      while `i' <= `nrows' {
            local k = 1
*           local ti=0
```

```
            scalar ti=0
            while `k' < `i' {

* Product of eb
                    local j = `k' + 1
*                   local pebi=1
                    scalar pebi=1
                    while `j' < `i' {
*                           local pebi = `pebi'*ebi[`j']
                            scalar pebi = pebi*ebi[`j']
* Next interval
                            local j=`j'+1
                    }

*                   local ti = `ti' + eai[`i']*`pebi'*p2b[`k']
                    scalar ti = ti + eai[`i']*pebi*p2b[`k']
*                   noi di "i=`i', k = `k', eai: " eai[`i'] " pebi: `pebi' p2b: " p2b[`k']
* Next inner sum
                    local k = `k' + 1
            }

* Sum variance components
*           local p1=p1[`i']
*           noi di "p1: `p1' ti: `ti'"
*           replace `se_vint' = sqrt(p1 + 2*`ti') if _n==`i'
            replace `se_vint' = sqrt(p1 + 2*ti) if _n==`i'

* Next outer sum
            local i = `i' + 1

      }

* Tidy up
      foreach var of local array {
            capture drop `var'
      }
      scalar drop _all
```

```
* Procedure: two_sigf.do <inum>
*
* Displays a number to 2 sig figs or as <0.0001
* (Lancet rules)
*
* Will output the number as a global variable and also give an "<" or "=" sign
*
*  inum = local variable holding number
*
* PMcG: Nov 2023

************
* Constant *
************
	local dp=2
	local w=4

*************
* User input*
*************
	local inum "`1'"

*************
* Output    *
*************
	global onum 0
	global osign "="


	if `inum' < 0.0001 {
		global onum "0.0001"
		global osign "<"
	}
	else {
		if `inum' <0.1 {
			local w=5
			local dp=3
		}
		if `inum' <0.01 {
			local w=6
			local dp=4
		}
		if `inum' <0.001 {
			local w=7
			local dp=5
		}
		global onum : display %`w'.`dp'f `inum'
	}
```
